# Isolation of *Streptococcus agalactiae* in a female llama (*Lama glama*) in South Tyrol (Italy)

**DOI:** 10.1186/s12917-018-1676-9

**Published:** 2018-11-13

**Authors:** Alexander Tavella, Astrid Bettini, Monia Cocchi, Ilda Idrizi, Stefano Colorio, Laura Viel, Claudia Zanardello, Patrik Zanolari

**Affiliations:** 1 Istituto Zooprofilattico Sperimentale delle Venezie, Viale dell’Università 10, 35020 Legnaro, Italy; 2 Clinic for Ruminants, Vetsuisse-Faculty, University of Bern, Bremgartenstrasse 109A, 3012 Bern, Switzerland

**Keywords:** *Streptococcus agalactiae*, *Lama glama*, South American camelids, Lancefield group B *Streptococcus*

## Abstract

**Background:**

*Streptococcus agalactiae* is pathogenic for both animals and humans. In dairy cattle it commonly causes mastitis, with great economic losses, and there is scientific evidence of mastitis, caseous lymphadenitis, contagious skin necrosis and purulent infections associated with *S. agalactiae* in camels (*Camelus dromedarius*) as well. In humans, it is a common component of the respiratory and gastrointestinal microflora, but it can also act as a pathogen, especially in elderly people and immunocompromised patients, as well as in pregrant women and newborns.

**Case presentation:**

A 10-year old non-pregnant female llama (*Lama glama*) was conferred to the Institute for Animal Health Control, in Bolzano for necropsy after sudden death. The animal had not shown unusual behaviour and had a low to normal nutritional condition (body condition score 2/5). The breeder had reported a chronic suppurative subcutaneous infection in the intermandibular area, resistant to therapy (therapy unknown). After necropsy, several samples were processed for histological, bacteriological and parasitological examinations.

**Conclusions:**

This report describes, to the best of our knowledge, the first isolation of *S. agalactiae* in llamas (*Lama glama*). The animal came from a herd that counts approximately 200 South American camelids (llamas, alpacas) along with several horses, chicken, rabbits, cats and dogs; this farm offers services, such as trekking and pet therapy activities.

**Electronic supplementary material:**

The online version of this article (10.1186/s12917-018-1676-9) contains supplementary material, which is available to authorized users.

## Background

Llamas and Alpacas have gained increasing interest in the last 10 years and are now frequently held as farm animals and for hobby purposes (trekking, pet therapy) in the alpine regions of Northern Italy. In the Autonomous Province of Bolzano – South Tyrol (Italy), the South American camelids (SACs) population has grown lately and currently counts 800 individuals (583 llamas and 217 alpacas – informations from the local Veterinary Service 2017-databank), representing an important *niche product* in local livestock breeding. In fact, SACs are often held in multispecies farming systems with sheep and horses, and could come into close contact with humans for trekking and pet therapy reasons.

*Streptococcus agalactiae (S. agalactiae)*, a Lancefield Group B *Streptococcus* (GBS), is an important pathogen affecting both animals and humans [1]. In dairy cattle, it is a major cause of mastitis and an important source of economic loss [2]. GBS have also been found in many other animals, such as camels, dogs, cats, crocodiles, seals, fish and dolphins [3–5]. More in detail, *S. agalactiae* has been frequently observed in camels (*Camelus dromedarius*)*,* as a causative agent of mastitis, caseous lymphadenitis, contagious skin necrosis and purulent infections [6–8]. Moreover, authors described that the camel strains are different from the bovine strains, and that they resemble much more the human strains [3]. To our knowledge, no *S. agalactiae* strains have been isolated from llamas (*Lama glama*).

*S. agalactiae* has also been observed in humans. It is a common component of the microflora of the respiratory and gastrointestinal tracts, approximately isolated in 30% of healthy humans. In the elderly and in immunocompromised patients, this bacterium can be associated with urinary tract and skin and soft-tissue infections, bacteremia, osteomyelitis, meningitis and endocarditis [9, 10]. Moreover, in pregnant women, invasive maternal infection is associated with abortion, preterm delivery [11], sepsis and meningitis in newborns [12]. In humans, invasive infections caused by *S. agalactiae* are reported increasingly worldwide [13]. In the pathogenetic mechanism, the adherence to host epithelial cells is the first critical step of the infectious process, leading to the formation of microbial biofilms. These consist of microcolonies encased in extracellular polysaccharide material. Bacteria inside biofilms have increased resistance to antimicrobial agents and disinfectants [14]. Furthermore, the production of biofilms is correlated with both pathogenicity and virulence of the bacteria.

In this report, we describe the case of a 10-year-old non-pregnant female llama (*Lama glama*), conferred for necropsy to the Institute for Animal Health Control (*Istituto Zooprofilattico Sperimentale delle Venezie*, IZSVe) in Bolzano (Italy) after sudden death.

## Case presentation

### Clinical history

The animal had been kept all summer with other six llamas on an alpine pasture, but it originated from a larger llama breeding farm where no *S. agalactiae*-cases were ever recorded. The affected llama had not shown an unusual behavior. The breeder reported a chronic suppurative subcutaneous infection in the intermandibular area, that had persisted for several months. The nutritional condition of the individual was low to normal (body condition score 2/5) and was evaluated through adspection and palpation of the thorax, the abdomen and the back [15]. The animal was found dead by the breeder on the alpine pasture.

### Necropsy

The physical exam revealed a 2–3 cm fistulating lump in the intermandibular area. After removing both hair and skin surfaces, a delimited suppurative infection site was observed in the subcutaneous tissue, in form of an abscess. Inspection of the anatomic area did not reveal a connection to the animal’s oral cavity. A diffused subcutaneous oedema was noted in the intermandibular area, as well as in the neck and the caudal portion of the head. The retropharyngeal lymph nodes were enlarged and oedematous. After opening the lump, a yellowish creamy content was observed.

The exam of the abdominal cavity showed a severe sero-fibrinous ascites. The C3 intestinal compartment showed a catarrhal-haemorrhagic inflammation of the mucous membrane, while the proximal part of the bowel portion was hyperaemic; furthermore, a catarrhal duodenojejunitis was recognized. A severe nematode larvae infestation was identified in the intestinal content.

The liver had a light brown colour and a brittle consistency and showed a diffused necrosis.

In the thoracic cavity, a high-grade of sero-fibrinogenous pleural effusion was identified. The lung showed a pronounced oedema, as well as emphysematous areas; furthermore, an interstitial pneumonia was observed.

The pericardium and heart base were oedematous. No other pathological lesions were observed in any other organ.

Selected samples from liver and lung tissues were routinely processed for histopathological examination. Moreover, bacteriological examinations from the intermandibular abscess, the liver and the lungs were performed by routine laboratory tests, using blood agar (in-house protocol) at 37 ± 1 °C in aerobiosis for 24 h. The flotation procedure was performed, using a solution with specific gravity of 1.3.

### Histology

**Fig. 1:**
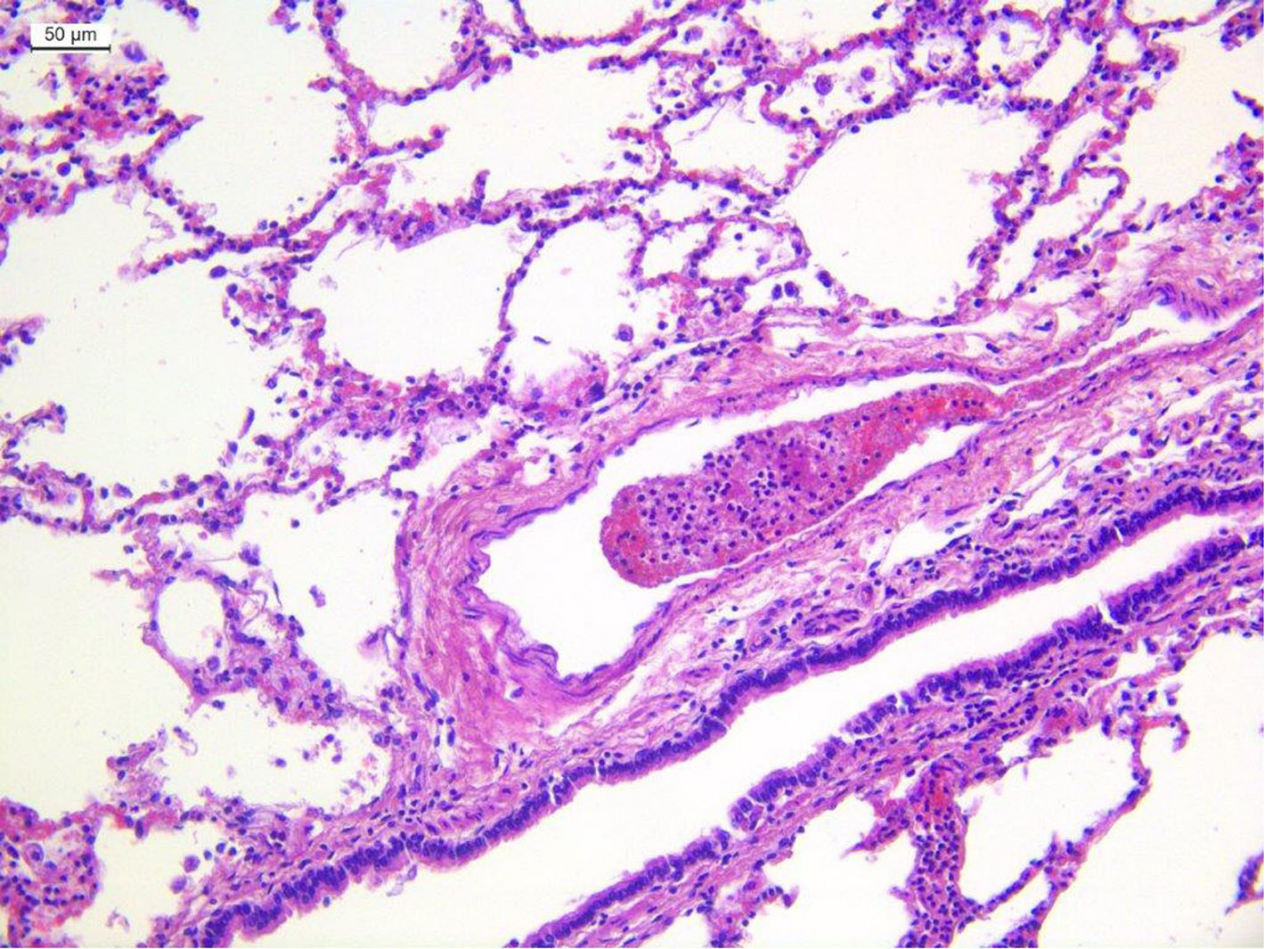
Lung - Mild to moderate neutrophilic and lymphocytic interstitial infiltration with alveolar emphysema. Presence of a thrombus in the blood vessel. HE, 20X

Samples of lung and liver parenchyma were first collected and fixed in 10% neutral buffered formalin, then processed, paraffin embedded, stained with Haematoxylin and Eosin (HE) and observed by standard light microscopy for histological examination. A diffuse mild to moderate neutrophilic and lymphocytic interstitial infiltration with alveolar emphysema was observed in the lung parenchyma (Fig. 1).

**Fig. 2:**
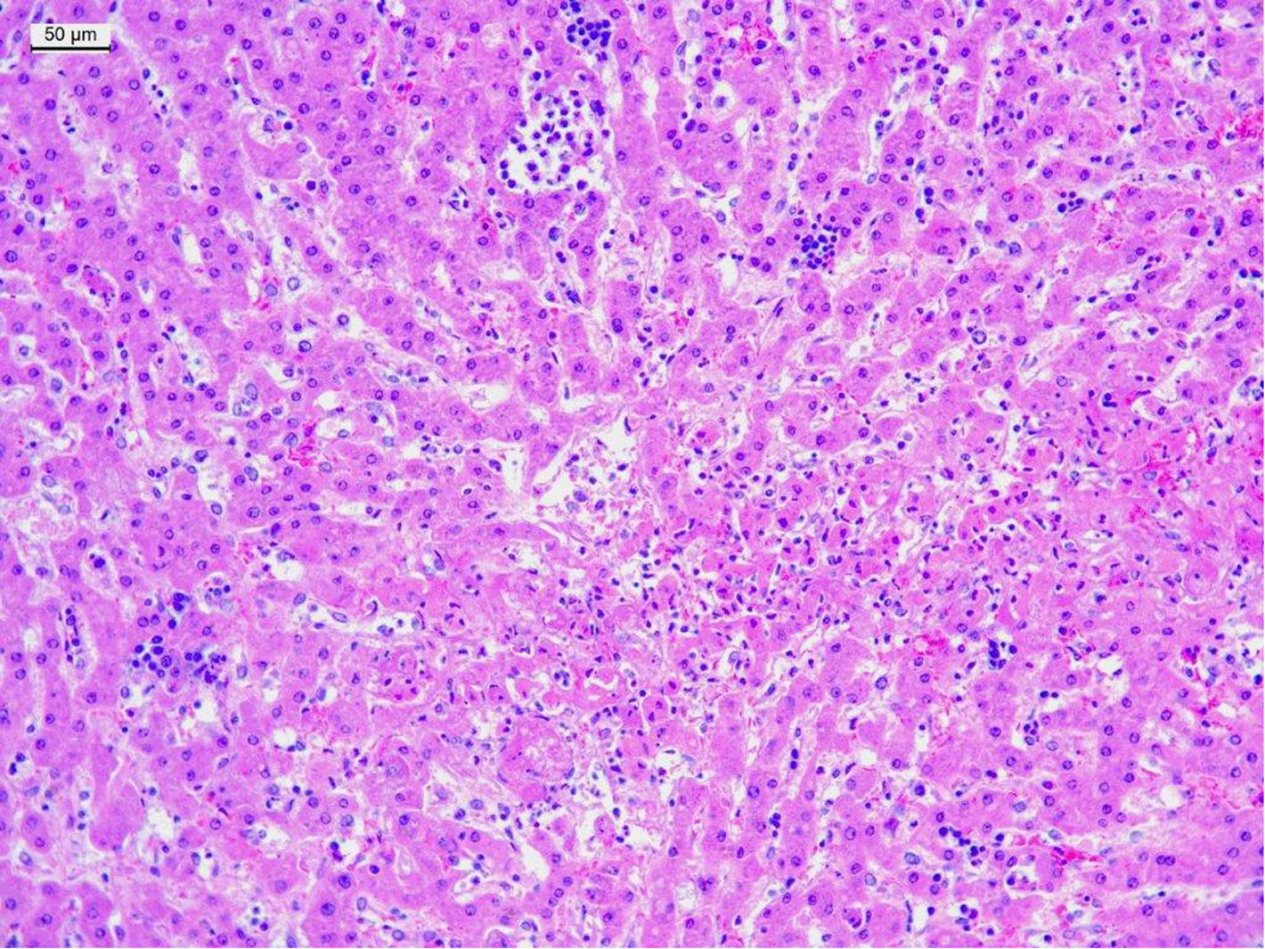
Liver – Foci of necrosis of hepatocytes with neutrophilic infiltration. HE, 20X

While examining the liver parenchyma, a multifocal necrosis of hepatocytes with foci of neutrophilic and lymphocytic infiltration and diffuse hydropic degeneration of hepatocytes were detected (Fig. 2).

### Bacterial examination

After incubation, a pure culture of translucid grey colonies, with a complete ß hemolysis was submitted for identification. Gram staining revealed Gram positive cocci in linear chains, catalase negative, and esculin negative as well.

To confirm the presence of ß hemolytic *Streptococci*, the CAMP test was performed, indicating a positive reaction, the serological assay (Streptococcal^EN^ Grouping Kit, Oixoid, Wade road, Basingstoke, Hampshire, UK) was performed as well, showing group B antigens. Biochemical identification of isolates (api® 32 Strep, bioMéerieux, Marcy-L’Etoile, France), revealed *S. agalactiae* (profile: 16122051110; %ID = 81.2; *T* = 0.77), with an excellent level of genus identification.

*S. agalactiae* was isolated from the purulent material of the intermandibular abscess and from the pulmonary parenchyma.

Biofilm formation was evaluated using micro titer plates as described by Stepanovic and coworkers [14].

In order to confirm the identification of the isolated strains obtained from the abscess and from the lung, Matrix-Assisted Laser Desorption/Ionization Time-Of-Flight Mass-Spectrometry (MALDI TOF MS) (Maldi Biotyper, Bruker Daltonics) was performed. This technique allows the identification of a bacterium by determining the molecular mass of fingerprint peptides (mainly ribosomal proteins), and comparing the mass fingerprint of the unknown strains to a database containing reference mass fingerprints. As specified by the manufacturer, a score value of < 1.7 indicates that identification is not reliable, scores between 1.7 and 2.0 demonstrate that identification is reliable at the genus level, scores between 2.0 and 2.3 evidence that identification is reliable at the genus level and probable at the species level. Scores higher than 2.3 indicate highly probable species identification.

*S. agalactiae* strains were recognized with a score of 2.454 (abscess isolate), and a score of 2.465 (lung isolate), indicating a highly probable species identification.

The strains formed biofilms under in vitro conditions, and were identified as moderate biofilm producers.

### Determination of the minimum inhibitory concentration (MIC)

Minimum Inhibitory Concentration (MIC) of the lung and abscess isolates was evaluated in accordance with the guidelines of the Clinical and Laboratory Standards Institute [16] for microwell dilution testing, using a commercial plate (Micronaut-S, Merlin, Kleinstrasse 14, Bornheim, Hersel, Germany). The antimicrobials tested were ampicillin, ceftiofur, enrofloxacin, florphenycol, spectinomycin, tetracycline, tilmicosin, trimethoprim/sulphamethoxazole. The MIC results are shown in Table 1.

**Table 1:** Inhibitory concentrations of the isolated *S. agalactiae* strain

Strain	Ampicillin μg/ml	Ceftiofur μg/ml	Enrofloxacin μg/ml	Florfenicol μg/ml	Spectinomycin μg/ml	Tetracycline μg/ml	Tilmicosin μg/ml	Trimethprim/Sulfamethoxazole μg/ml
Abscess	0.125	0.125	0.5	1	128	0.25	4	0.125/2.375
Lung	0.125	0.125	0.5	1	128	0.25	4	0.125/2.375

### Parasitology

Parasitology of the feces reported an elevated number of oval eggs with a thin single wall, measuring approximately 45x30μm, attributable to *Strongyloides spp*.. Several eggs had transformed to the larval stage. Furthermore, yellow-brownish eggs (approximately 70 × 30 μm) showing a “lemon” shape with evident stoppers at the poles referred to *Trichuris spp*. were observed. The elevated number of eggs observed at the microscope and the evidence of the severe enteral parasitaemia allowed the indirect evaluation of the infestation intensity.

### Molecular characterization

Both isolates were submitted for 16S sequencing. The alignment using the NCBI GenBank BLAST function (Additional files 1 and 2) and the Clustal Omega application (Additional file 3) confirmed the identification of *S. agalactiae*.

## Discussion and conclusions

*S. agalactiae* has already been isolated from skin lesions, periarticular abscesses and mastitic udder in camelids (*Camelus dromedarius*) [3, 6, 17]. Furthermore, it has been described as an opportunistic pathogen of the upper respiratory tract [18]. *S. agalactiae* has been isolated from caseus lymphadenitis, contagious skin necrosis and purulent infections in camels [3]; it has also been observed in wounds caused by *Hyalomma* spp. in dromedaries in Kenya [19]. So far, no *S. agalactiae* have been described in llamas, instead *S. zooepidemicus, S. pyogenes, S. faecalis* and *S. uberis* have been isolated from alpacas in Peru [20]. Therefore, to the best of our knowledge, this represents the first isolation of *S. agalactiae* from llamas (*Lama glama*).

In this report, the identification of *S. agalactiae* was performed in an abscess and in the lung parenchima in an animal presenting a parasitic infestation.

Parasitism in SACs has become a major health concern, as several parasites, in particular gastrointestinal nematodes, are believed to cause mild-to-severe clinical diseases and economic losses. Although death from parasitic gastroenteritis associated with gastrointestinal nematodes may occur, infections tend to be more insidious, often presenting nonspecific clinical signs (i.e. diarrhoea, anorexia and poor growth) or asymptomatic conditions [21]. Domesticated SACs, comprising llamas, often share pasture with other livestock species and are often farmed under more intensive grazing conditions as in their native countries, factors which may significantly increase the risk of nematode infections [22]. In the present case, an elevated number of eggs attributable to *Strongyloides spp.* and *Trichuris spp.* were observed: this has been identified in the large intestine of SACs in previous studies as well [21–23]. Infections with Trichuris spp. can lead to severe anemia [15], even though many clinical signs are often absent at the early stages of infection [24]. The abdominal cavity lesions observed can be ascribed to the intense infestation by gastrointesintal nematodes [23, 25]. Moreover, this could act as a predisposing factor for septicemic streptococcosis.

*S. agalactiae* is a component of the microflora of the respiratory, genital and gastrointestinal tracts in both humans and ruminants. In humans, GBS is also an important cause of morbidity and mortality in newborns, in the elderly and in immunocompromised adults [26]. Primary manifestations include bacteremia, skin and soft tissue infections, while other signs are referred to pneumonia, osteomyelitis and urinary tract infections [27, 28]. Furthermore, invasive infections are increasingly reported worldwide: Takahashi and coworkers [13] have highlighted significant differences in clinical aspects, including prognosis, between disease-entities caused by *S. agalactiae* and other Streptococci in human diseases. In veterinary medicine, *S. agalactiae* is the main cause of subclinical mastitis in cattle. To our knowledge, no report of septicemic conditions have been observed in SACs. In other species, the bacterial colonization and infection requires the capacity of the bacterium to adhere and to persist. The formation of biofilm-like communities could facilitate microbial survival and proliferation by enhancing resistance to host defenses and nutrient deprivation [29]. In our report, the isolates were indeed biofilm producers. The production of biofilm was correlated with pathogenicity and virulence of some bacteria [30]. In fact, microorganisms inside biofilms have increased resistance to antimicrobial agents and disinfectants, indicating the pathogenicity potential in the isolated strains. The analysis of virulence factors production was not performed in this report, though previous reports have highlighted the potential of virulence factors production by *S. agalactiae* in bovine mastitis [31]. Furthermore, biofilm formation appears to be a prerequisite for colonization of the bovine mammary gland. *S. agalactiae* isolates recovered from bovine subclinical mastitis produced different pH-dependent biofilm levels, suggesting that biofilm production is modulated by environmental factors [28, 32]. No data are available for isolates from llamas.

Identification of the bacteria was performed by a commerical kit and by MALDI TOF MS. This last procedure is routinely used in many laboratories, and has shown to be a rapid and reliable technique for the identification of veterinary bacteria [33]. Furthermore, Lartigue and coworkers [34] have identified over 100 *S. agalactiae* isolates, further charachterized by serotyping and multilocus sequence typing, with the use of MALDI TOF MS. Based on the previously described evaluation scores, a score higher than 2.3 allows the highly probable species identification. In this case report, *S. agalactiae* strains were identified with a score of 2.454 (abscess isolate), and a score of 2.465 (lung isolate).

The determination of MIC was performed in order to evaluate potential antimicrobial resistances. Since no CLSI official breakpoints are available for llamas, and in order to provide a laboratory result, the MIC results (Table 1) were evaluated using the bovine clinical breakpoints, even though several authors have observed that llamas, but also other SACs, and bovines present different pharmacokinetics and pharmacodynamics [35, 36]. The evaluation, with bovine breakpoints, highlights sensitivity to all antimicrobials tested. While no official breakpoints are available for SACs, non official breakpoints have been proposed for ampicillin, ceftiofur and enrofloxacin [15]. Based on these data, the MIC results would suggest ampicillin and ceftiofur sensitivity by IV and IM administration, and an intermediate resistance to enrofloxacin by IV administration.

The breeding of SACs in the Autonomous Province of Bolzano – South Tyrol (Italy) has grown considerably in the last 10 years, conveying our Province a leading position in this field. These animals have gained great fame especially as hobby animals in zoos, private hotel gardens, as trekking animals and for pet therapy purposes, leading to a close contact between animals and humans, pointing out the possible risk of the transmission to humans. In fact, transmission of *S. agalactiae* from animals to humans has been briefly described in cattle [37, 38]; the authors describe a probable linkage between cattle exposure and detection of GBS in humans, stating that *S. agalactiae* can be transmitted between bovines and humans in a farm environment and that increased cattle exposure is associated with higher risk of infection. To this regard, no data are available for SACs, and molecular characterization of the isolates should be performed in order to establish the zoonotic potential of *S. agalactiae*.

## Additional files

Additional file 1: BLAST allignment of the abscess isolate. (PDF 387 kb)

Additional file 2: BLAST allignment of the lung isolate. (PDF 397 kb)

Additional file 3: Clustal Omega allignment. (PDF 62 kb)

## Abbreviations

CAMP test: Christie Atkins Munch Petersen test
CLSI: Clinical and Laboratory Standards Institute
GBS: Lancefield Group B *Streptococcus*
HE: Haematoxylin and Eosin
IM: Intramuscolar inoculation
IV: Intravenous inoculation
IZSVe: Istituto Zooprofilattico delle Venezie
m/z: Mass to charge ratio
MALDI TOF MS: Matrix- Assisted Laser Desorption/Ionization Time-Of-Flight Mass Spectrometry
MIC: Minimum Inhibitory Concentration
*S. agalactiae*: *Streptococcus agalactiae*
SACs: South American camelids
