# Supplementary material for: Isolation of Streptococcus agalactiae in a female llama (Lama glama) in South Tyrol (Italy)
Source: BMC Vet Res. 2018 Nov 13;14:343. doi: 10.1186/s12917-018-1676-9 (PMC6234556; doi:10.1186/s12917-018-1676-9)
Supplement: Supplementary file 1 — BLAST allignment of the abscess isolate. (PDF 387 kb) [file 12917_2018_1676_MOESM1_ESM.pdf]

BLAST ® » blastn suite » RID-RDAWS5SX015

BLAST Results

Questions/comments

Job title: Consensus for segment MG386600 for specimen...

**RID**	RDAWS5SX015 (Expires on 08-18 19:03 pm)		
**Query ID**	lcl|Query_38841	**Database Name**	nr
**Description**	Consensus for segment MG386600 for specimen 18RS-1924-7	**Description**	Nucleotide collection (nt)
**Molecule type**	nucleic acid	**Program**	BLASTN 2.8.0+
**Query Length**	820		

Graphic Summary

Distribution of the top 200 Blast Hits on 100 subject sequences

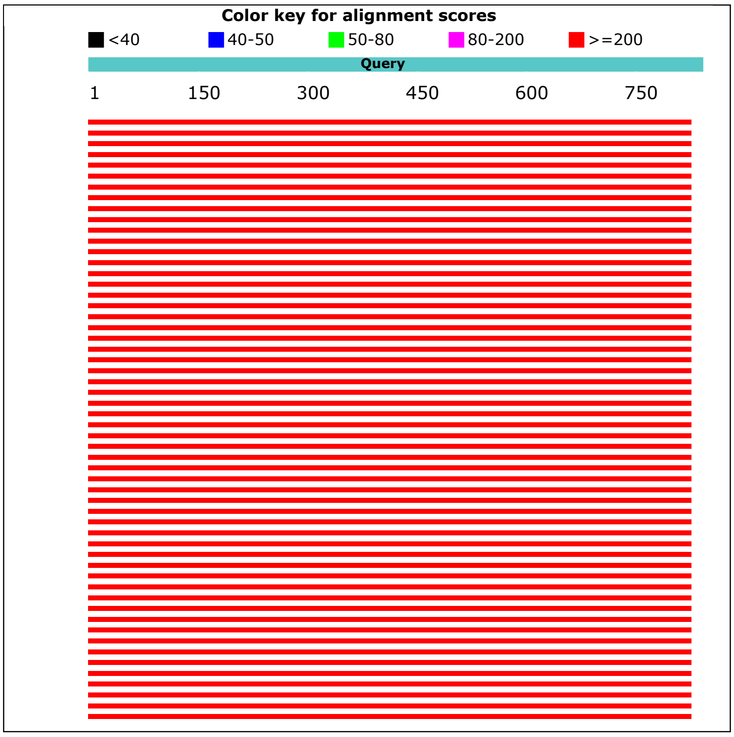

Descriptions

Sequences producing significant alignments:

Description	Max score	Total score	Query cover	E value	Ident	Accession
Streptococcus agalactiae strain GZ120 16S ribosomal RNA gene, partial sequence	1515	1515	100%	0.0	100%	MG386600.1
Streptococcus agalactiae strain B111 chromosome, complete genome	1515	10607	100%	0.0	100%	CP021772.1
Streptococcus agalactiae strain B507 chromosome, complete genome	1515	10590	100%	0.0	100%	CP021771.1
Streptococcus agalactiae strain B509 chromosome, complete genome	1515	10596	100%	0.0	100%	CP021769.1
Streptococcus agalactiae strain NCTC11930 genome assembly, chromosome: 1	1515	10585	100%	0.0	100%	LS483342.1
Streptococcus agalactiae strain SGEHI2015-25 chromosome, complete genome	1515	10579	100%	0.0	100%	CP025029.1
Streptococcus agalactiae strain SGEHI2015-107 chromosome, complete genome	1515	10574	100%	0.0	100%	CP025027.1
Streptococcus agalactiae strain QX1503 16S ribosomal RNA gene, partial sequence	1515	1515	100%	0.0	100%	MF113268.1
Streptococcus agalactiae strain HN1517 16S ribosomal RNA gene, partial sequence	1515	1515	100%	0.0	100%	MF113266.1
Streptococcus agalactiae strain HN1503 16S ribosomal RNA gene, partial sequence	1515	1515	100%	0.0	100%	MF113265.1
Streptococcus agalactiae strain GXN1417 16S ribosomal RNA gene, partial sequence	1515	1515	100%	0.0	100%	MF113264.1
Streptococcus agalactiae strain GXN1402 16S ribosomal RNA gene, partial sequence	1515	1515	100%	0.0	100%	MF113263.1
Streptococcus agalactiae strain GD1221 16S ribosomal RNA gene, partial sequence	1515	1515	100%	0.0	100%	MF113262.1
Streptococcus agalactiae strain GD1212 16S ribosomal RNA gene, partial sequence	1515	1515	100%	0.0	100%	MF113261.1
Streptococcus agalactiae strain NYD007 16S ribosomal RNA gene, partial sequence	1515	1515	100%	0.0	100%	MF113259.1
Streptococcus agalactiae strain NYD001 16S ribosomal RNA gene, partial sequence	1515	1515	100%	0.0	100%	MF113258.1
Streptococcus agalactiae strain BJ1411 16S ribosomal RNA gene, partial sequence	1515	1515	100%	0.0	100%	MF113257.1
Streptococcus agalactiae strain QX1501 16S ribosomal RNA gene, partial sequence	1515	1515	100%	0.0	100%	MF037804.1
Streptococcus agalactiae strain PK1601 16S ribosomal RNA gene, partial sequence	1515	1515	100%	0.0	100%	MF037803.1
Streptococcus agalactiae strain HN1506 16S ribosomal RNA gene, partial sequence	1515	1515	100%	0.0	100%	MF037798.1
Streptococcus agalactiae strain GXN1411 16S ribosomal RNA gene, partial sequence	1515	1515	100%	0.0	100%	MF037796.1
Streptococcus agalactiae strain NYD03 16S ribosomal RNA gene, partial sequence	1515	1515	100%	0.0	100%	MF037794.1
Streptococcus agalactiae strain NZY014 16S ribosomal RNA gene, partial sequence	1515	1515	100%	0.0	100%	MF037793.1
Streptococcus agalactiae strain BJ1402 16S ribosomal RNA gene, partial sequence	1515	1515	100%	0.0	100%	MF037792.1
Streptococcus agalactiae strain ZJRZ1623 16S ribosomal RNA gene, partial sequence	1515	1515	100%	0.0	100%	MF037790.1
Streptococcus agalactiae strain ZJRZ1620 16S ribosomal RNA gene, partial sequence	1515	1515	100%	0.0	100%	MF037789.1
Streptococcus agalactiae strain ZJRZ1619 16S ribosomal RNA gene, partial sequence	1515	1515	100%	0.0	100%	MF037788.1
Streptococcus agalactiae strain ZJRZ1604 16S ribosomal RNA gene, partial sequence	1515	1515	100%	0.0	100%	MF037786.1
Streptococcus agalactiae strain ZJRZ1603 16S ribosomal RNA gene, partial sequence	1515	1515	100%	0.0	100%	MF037785.1
Streptococcus agalactiae strain NJJNB1619 16S ribosomal RNA gene, partial sequence	1515	1515	100%	0.0	100%	MF037784.1
Streptococcus agalactiae strain NJJNB1612 16S ribosomal RNA gene, partial sequence	1515	1515	100%	0.0	100%	MF037783.1
Streptococcus agalactiae strain NJJNB1608 16S ribosomal RNA gene, partial sequence	1515	1515	100%	0.0	100%	MF037782.1
Streptococcus agalactiae strain NJJNB1606 16S ribosomal RNA gene, partial sequence	1515	1515	100%	0.0	100%	MF037781.1
Streptococcus agalactiae strain NJJNB1602 16S ribosomal RNA gene, partial sequence	1515	1515	100%	0.0	100%	MF037780.1
Streptococcus agalactiae strain HAHY1335 16S ribosomal RNA gene, partial sequence	1515	1515	100%	0.0	100%	MF037778.1
Streptococcus agalactiae strain HAHY1332 16S ribosomal RNA gene, partial sequence	1515	1515	100%	0.0	100%	MF037776.1
Streptococcus agalactiae strain HAHY1322 16S ribosomal RNA gene, partial sequence	1515	1515	100%	0.0	100%	MF037775.1
Streptococcus agalactiae strain HAHY1315 16S ribosomal RNA gene, partial sequence	1515	1515	100%	0.0	100%	MF037774.1
Streptococcus agalactiae strain HAQH1336 16S ribosomal RNA gene, partial sequence	1515	1515	100%	0.0	100%	MF037772.1
Streptococcus agalactiae strain HAQH1335 16S ribosomal RNA gene, partial sequence	1515	1515	100%	0.0	100%	MF037771.1
Streptococcus agalactiae strain HAQH1312 16S ribosomal RNA gene, partial sequence	1515	1515	100%	0.0	100%	MF037768.1
Streptococcus agalactiae strain HAQH1309 16S ribosomal RNA gene, partial sequence	1515	1515	100%	0.0	100%	MF037767.1
Streptococcus agalactiae strain NJJNA1632 16S ribosomal RNA gene, partial sequence	1515	1515	100%	0.0	100%	MF037766.1
Streptococcus agalactiae strain NJJNA1619 16S ribosomal RNA gene, partial sequence	1515	1515	100%	0.0	100%	MF037764.1
Streptococcus agalactiae strain NJJNA1613 16S ribosomal RNA gene, partial sequence	1515	1515	100%	0.0	100%	MF037763.1
Streptococcus agalactiae strain NJJNA1612 16S ribosomal RNA gene, partial sequence	1515	1515	100%	0.0	100%	MF037762.1
Streptococcus agalactiae strain NJJNA1608 16S ribosomal RNA gene, partial sequence	1515	1515	100%	0.0	100%	MF037761.1
Streptococcus agalactiae strain NJPK1419 16S ribosomal RNA gene, partial sequence	1515	1515	100%	0.0	100%	MF037759.1
Streptococcus agalactiae strain NJPK1407 16S ribosomal RNA gene, partial sequence	1515	1515	100%	0.0	100%	MF037757.1
Streptococcus agalactiae strain NJPK1406 16S ribosomal RNA gene, partial sequence	1515	1515	100%	0.0	100%	MF037756.1
Streptococcus agalactiae strain HZJG1236 16S ribosomal RNA gene, partial sequence	1515	1515	100%	0.0	100%	MF037754.1
Streptococcus agalactiae strain HZJG1228 16S ribosomal RNA gene, partial sequence	1515	1515	100%	0.0	100%	MF037753.1
Streptococcus agalactiae strain HZJG1216 16S ribosomal RNA gene, partial sequence	1515	1515	100%	0.0	100%	MF037751.1
Streptococcus agalactiae strain HZJG1211 16S ribosomal RNA gene, partial sequence	1515	1515	100%	0.0	100%	MF037750.1
Streptococcus agalactiae strain CZFY1231 16S ribosomal RNA gene, partial sequence	1515	1515	100%	0.0	100%	MF037748.1
Streptococcus agalactiae strain CZFY1227 16S ribosomal RNA gene, partial sequence	1515	1515	100%	0.0	100%	MF037747.1
Streptococcus agalactiae strain CZFY1214 16S ribosomal RNA gene, partial sequence	1515	1515	100%	0.0	100%	MF037746.1
Streptococcus agalactiae strain CZFY1209 16S ribosomal RNA gene, partial sequence	1515	1515	100%	0.0	100%	MF037744.1
Streptococcus agalactiae strain CZFY1207 16S ribosomal RNA gene, partial sequence	1515	1515	100%	0.0	100%	MF037743.1
Streptococcus agalactiae strain SHFX1111 16S ribosomal RNA gene, partial sequence	1515	1515	100%	0.0	100%	MF037741.1
Streptococcus agalactiae strain SHFX1106 16S ribosomal RNA gene, partial sequence	1515	1515	100%	0.0	100%	MF037740.1
Streptococcus agalactiae strain SHFX1102 16S ribosomal RNA gene, partial sequence	1515	1515	100%	0.0	100%	MF037738.1

Description	Max score	Total score	Query cover	E value	Ident	Accession
Streptococcus agalactiae strain SHFX1101 16S ribosomal RNA gene, partial sequence	1515	1515	100%	0.0	100%	MF037737.1
Streptococcus agalactiae strain 3X23 16S ribosomal RNA gene, partial sequence	1515	1515	100%	0.0	100%	KY765027.1
Streptococcus agalactiae strain C001, complete genome	1515	9092	100%	0.0	100%	CP008813.1
Streptococcus agalactiae strain SG-M8, complete genome	1515	10607	100%	0.0	100%	CP021868.1
Streptococcus agalactiae strain CUGBS591, complete genome	1515	10596	100%	0.0	100%	CP021862.1
Streptococcus agalactiae strain Sag37, complete genome	1515	10576	100%	0.0	100%	CP019978.1
Streptococcus agalactiae strain GBS-M002, complete genome	1515	10607	100%	0.0	100%	CP013908.1
Uncultured bacterium clone gao21 16S ribosomal RNA gene, partial sequence	1515	1515	100%	0.0	100%	KU363876.1
Streptococcus agalactiae strain WC1535, complete genome	1515	10563	100%	0.0	100%	CP016501.1
Streptococcus agalactiae strain CU_GBS_98, complete genome	1515	7554	100%	0.0	100%	CP010875.1
Streptococcus agalactiae strain CU_GBS_08 chromosome, complete genome	1515	7554	100%	0.0	100%	CP010874.1
Streptococcus agalactiae strain GBS85147, complete genome	1515	9062	100%	0.0	100%	CP010319.1
Streptococcus agalactiae strain GX064 genome	1515	9064	100%	0.0	100%	CP011327.1
Streptococcus agalactiae strain YM001 genome	1515	9064	100%	0.0	100%	CP011326.1
Streptococcus agalactiae strain HN016, complete genome	1515	10574	100%	0.0	100%	CP011325.1
Streptococcus agalactiae strain HZ10 16S ribosomal RNA gene, partial sequence	1515	1515	100%	0.0	100%	KF111311.1
Uncultured Streptococcus sp. clone M52 16S ribosomal RNA gene, partial sequence	1515	1515	100%	0.0	100%	JX841329.1
Uncultured Streptococcus sp. clone M11 16S ribosomal RNA gene, partial sequence	1515	1515	100%	0.0	100%	JX841322.1
Streptococcus agalactiae GD201008-001, complete genome	1515	10542	100%	0.0	100%	CP003810.1
Streptococcus agalactiae strain B7 16S ribosomal RNA gene, partial sequence	1515	1515	100%	0.0	100%	JN176347.1
Uncultured bacterium clone GoC_Bac_17_D1_C0_M0 16S ribosomal RNA gene, partial sequence	1515	1515	100%	0.0	100%	FJ813519.1
Streptococcus agalactiae strain 14-ninetytwomp 16S ribosomal RNA gene, partial sequence	1515	1515	100%	0.0	100%	EU075069.1
Streptococcus agalactiae A909, complete genome	1515	10579	100%	0.0	100%	CP000114.1
Streptococcus agalactiae strain GZ2058 16S ribosomal RNA gene, partial sequence	1509	1509	100%	0.0	99%	MG386601.1
Streptococcus agalactiae strain S73 chromosome, complete genome	1509	9185	100%	0.0	99%	CP030845.1
Streptococcus agalactiae strain B105 chromosome, complete genome	1509	10568	100%	0.0	99%	CP021773.1
Streptococcus agalactiae strain B508 chromosome, complete genome	1509	10568	100%	0.0	99%	CP021770.1
Streptococcus agalactiae strain NCTC8187 genome assembly, chromosome: 1	1509	9058	100%	0.0	99%	LS483387.1
Streptococcus agalactiae strain L0023-06 16S ribosomal RNA gene, partial sequence	1509	1509	100%	0.0	99%	MH463668.1
Streptococcus agalactiae strain L0023-02F 16S ribosomal RNA gene, partial sequence	1509	1509	100%	0.0	99%	MH447034.1
Streptococcus agalactiae strain NQ-2018-5 16S ribosomal RNA gene, partial sequence	1509	1509	100%	0.0	99%	MH423900.1
Streptococcus agalactiae strain QMA0271 chromosome, complete genome	1509	3019	100%	0.0	99%	CP029632.1
Streptococcus agalactiae strain 16CS3 16S ribosomal RNA gene, partial sequence	1509	1509	100%	0.0	99%	MH158279.1
Streptococcus agalactiae strain 7PS3 16S ribosomal RNA gene, partial sequence	1509	1509	100%	0.0	99%	MH109009.1
Streptococcus agalactiae strain YUC18-1 MCC 3038 16S ribosomal RNA gene, partial sequence	1509	1509	100%	0.0	99%	MH021639.1
Streptococcus agalactiae strain FDAARGOS_254 chromosome, complete genome	1509	10568	100%	0.0	99%	CP020449.2
Streptococcus sp. 'group B' strain FDAARGOS_229 chromosome, complete genome	1509	10546	100%	0.0	99%	CP020432.2
Streptococcus agalactiae strain SGEHI2015-95 chromosome, complete genome	1509	10563	100%	0.0	99%	CP025028.1

Alignments

Streptococcus agalactiae strain GZ120 16S ribosomal RNA gene, partial sequence
Sequence ID: **MG386600.1** Length: 1406 Number of Matches: 1
Range 1: 2 to 821

Score	Expect	Identities	Gaps	Strand	Frame
1515 bits(820)	0.0()	820/820(100%)	0/820(0%)	Plus/Plus	

Features:

```
Query  1    CGCTGATGTTTGGTGTTTACACTAGACTGATGAGTTGCGAACGGGTGAGTAACGCGTAGG  60
            ||||||||||||||||||||||||||||||||||||||||||||||||||||||||||||
Sbjct  2    CGCTGATGTTTGGTGTTTACACTAGACTGATGAGTTGCGAACGGGTGAGTAACGCGTAGG  61

Query  61   TAACCTGCCTCATAGCGGGGGATAACTATTGGAAACGATAGCTAATACCGCATAAGAGTA  120
            ||||||||||||||||||||||||||||||||||||||||||||||||||||||||||||
Sbjct  62   TAACCTGCCTCATAGCGGGGGATAACTATTGGAAACGATAGCTAATACCGCATAAGAGTA  121

Query  121  ATTAACACATGTTAGTTATTTAAAAGGAGCAATTGCTTCACTGTGAGATGGACCTGCGTT  180
            ||||||||||||||||||||||||||||||||||||||||||||||||||||||||||||
Sbjct  122  ATTAACACATGTTAGTTATTTAAAAGGAGCAATTGCTTCACTGTGAGATGGACCTGCGTT  181

Query  181  GTATTAGCTAGTTGGTGAGGTAAAGGCTCACCAAGGCGACGATACATAGCCGACCTGAGA  240
            ||||||||||||||||||||||||||||||||||||||||||||||||||||||||||||
Sbjct  182  GTATTAGCTAGTTGGTGAGGTAAAGGCTCACCAAGGCGACGATACATAGCCGACCTGAGA  241

Query  241  GGGTGATCGGCCACACTGGGACTGAGACACGGCCCAGACTCCTACGGGAGGCAGCAGTAG  300
            ||||||||||||||||||||||||||||||||||||||||||||||||||||||||||||
Sbjct  242  GGGTGATCGGCCACACTGGGACTGAGACACGGCCCAGACTCCTACGGGAGGCAGCAGTAG  301

Query  301  GGAATCTTCGGCAATGGACGGAAGTCTGACCGAGCAACGCCGCGTGAGTGAAGAAGGTTT  360
            ||||||||||||||||||||||||||||||||||||||||||||||||||||||||||||
Sbjct  302  GGAATCTTCGGCAATGGACGGAAGTCTGACCGAGCAACGCCGCGTGAGTGAAGAAGGTTT  361

Query  361  TCGGATCGTAAAGCTCTGTTGTTAGAGAAGAACGTTGGTAGGAGTGGAAAATCTACCAAG  420
            ||||||||||||||||||||||||||||||||||||||||||||||||||||||||||||
Sbjct  362  TCGGATCGTAAAGCTCTGTTGTTAGAGAAGAACGTTGGTAGGAGTGGAAAATCTACCAAG  421

Query  421  TGACGGTAACTAACCAGAAAGGGACGGCTAACTACGTGCCAGCAGCCGCGGTAATACGTA  480
            ||||||||||||||||||||||||||||||||||||||||||||||||||||||||||||
Sbjct  422  TGACGGTAACTAACCAGAAAGGGACGGCTAACTACGTGCCAGCAGCCGCGGTAATACGTA  481

Query  481  GGTCCCGAGCGTTGTCCGGATTTATTGGGCGTAAAGCGAGCGCAGGCGGTTCTTTAAGTC  540
            ||||||||||||||||||||||||||||||||||||||||||||||||||||||||||||
Sbjct  482  GGTCCCGAGCGTTGTCCGGATTTATTGGGCGTAAAGCGAGCGCAGGCGGTTCTTTAAGTC  541

Query  541  TGAAGTTAAAGGCAGTGGCTTAACCATTGTACGCTTTGGAAACTGGAGGACTTGAGTGCA  600
            ||||||||||||||||||||||||||||||||||||||||||||||||||||||||||||
Sbjct  542  TGAAGTTAAAGGCAGTGGCTTAACCATTGTACGCTTTGGAAACTGGAGGACTTGAGTGCA  601

Query  601  GAAGGGGAGAGTGGAATTCCATGTGTAGCGGTGAAATGCGTAGATATATGGAGGAACACC  660
            ||||||||||||||||||||||||||||||||||||||||||||||||||||||||||||
Sbjct  602  GAAGGGGAGAGTGGAATTCCATGTGTAGCGGTGAAATGCGTAGATATATGGAGGAACACC  661

Query  661  GGTGGCGAAAGCGGCTCTCTGGTCTGTAACTGACGCTGAGGCTCGAAAGCGTGGGGAGCA  720
            ||||||||||||||||||||||||||||||||||||||||||||||||||||||||||||
Sbjct  662  GGTGGCGAAAGCGGCTCTCTGGTCTGTAACTGACGCTGAGGCTCGAAAGCGTGGGGAGCA  721
```

```
Query  721   AACAGGATTAGATACCCTGGTAGTCCACGCCGTAAACGATGAGTGCTAGGTGTTAGGCCC  780
             ||||||||||||||||||||||||||||||||||||||||||||||||||||||||||||
Sbjct  722   AACAGGATTAGATACCCTGGTAGTCCACGCCGTAAACGATGAGTGCTAGGTGTTAGGCCC  781

Query  781   TTTCCGGGGCTTAGTGCCGCAGCTAACGCATTAAGCACTC  820
             ||||||||||||||||||||||||||||||||||||||||
Sbjct  782   TTTCCGGGGCTTAGTGCCGCAGCTAACGCATTAAGCACTC  821
```

Streptococcus agalactiae strain B111 chromosome, complete genome
Sequence ID: **CP021772.1** Length: 2150631 Number of Matches: 7
Range 1: 1414830 to 1415649

Score	Expect	Identities	Gaps	Strand	Frame
1515 bits(820)	0.0()	820/820(100%)	0/820(0%)	Plus/Plus	

Features:

```
Query  1        CGCTGATGTTTGGTGTTTACACTAGACTGATGAGTTGCGAACGGGTGAGTAACGCGTAGG  60
                ||||||||||||||||||||||||||||||||||||||||||||||||||||||||||||
Sbjct  1414830  CGCTGATGTTTGGTGTTTACACTAGACTGATGAGTTGCGAACGGGTGAGTAACGCGTAGG  1414889

Query  61       TAACCTGCCTCATAGCGGGGGATAACTATTGGAAACGATAGCTAATACCGCATAAGAGTA  120
                ||||||||||||||||||||||||||||||||||||||||||||||||||||||||||||
Sbjct  1414890  TAACCTGCCTCATAGCGGGGGATAACTATTGGAAACGATAGCTAATACCGCATAAGAGTA  1414949

Query  121      ATTAACACATGTTAGTTATTTAAAAGGAGCAATTGCTTCACTGTGAGATGGACCTGCGTT  180
                ||||||||||||||||||||||||||||||||||||||||||||||||||||||||||||
Sbjct  1414950  ATTAACACATGTTAGTTATTTAAAAGGAGCAATTGCTTCACTGTGAGATGGACCTGCGTT  1415009

Query  181      GTATTAGCTAGTTGGTGAGGTAAAGGCTCACCAAGGCGACGATACATAGCCGACCTGAGA  240
                ||||||||||||||||||||||||||||||||||||||||||||||||||||||||||||
Sbjct  1415010  GTATTAGCTAGTTGGTGAGGTAAAGGCTCACCAAGGCGACGATACATAGCCGACCTGAGA  1415069

Query  241      GGGTGATCGGCCACACTGGGACTGAGACACGGCCCAGACTCCTACGGGAGGCAGCAGTAG  300
                ||||||||||||||||||||||||||||||||||||||||||||||||||||||||||||
Sbjct  1415070  GGGTGATCGGCCACACTGGGACTGAGACACGGCCCAGACTCCTACGGGAGGCAGCAGTAG  1415129

Query  301      GGAATCTTCGGCAATGGACGGAAGTCTGACCGAGCAACGCCGCGTGAGTGAAGAAGGTTT  360
                ||||||||||||||||||||||||||||||||||||||||||||||||||||||||||||
Sbjct  1415130  GGAATCTTCGGCAATGGACGGAAGTCTGACCGAGCAACGCCGCGTGAGTGAAGAAGGTTT  1415189

Query  361      TCGGATCGTAAAGCTCTGTTGTTAGAGAAGAACGTTGGTAGGAGTGGAAAATCTACCAAG  420
                ||||||||||||||||||||||||||||||||||||||||||||||||||||||||||||
Sbjct  1415190  TCGGATCGTAAAGCTCTGTTGTTAGAGAAGAACGTTGGTAGGAGTGGAAAATCTACCAAG  1415249

Query  421      TGACGGTAACTAACCAGAAAGGGACGGCTAACTACGTGCCAGCAGCCGCGGTAATACGTA  480
                ||||||||||||||||||||||||||||||||||||||||||||||||||||||||||||
Sbjct  1415250  TGACGGTAACTAACCAGAAAGGGACGGCTAACTACGTGCCAGCAGCCGCGGTAATACGTA  1415309

Query  481      GGTCCCGAGCGTTGTCCGGATTTATTGGGCGTAAAGCGAGCGCAGGCGGTTCTTTAAGTC  540
                ||||||||||||||||||||||||||||||||||||||||||||||||||||||||||||
Sbjct  1415310  GGTCCCGAGCGTTGTCCGGATTTATTGGGCGTAAAGCGAGCGCAGGCGGTTCTTTAAGTC  1415369

Query  541      TGAAGTTAAAGGCAGTGGCTTAACCATTGTACGCTTTGGAAACTGGAGGACTTGAGTGCA  600
                ||||||||||||||||||||||||||||||||||||||||||||||||||||||||||||
Sbjct  1415370  TGAAGTTAAAGGCAGTGGCTTAACCATTGTACGCTTTGGAAACTGGAGGACTTGAGTGCA  1415429

Query  601      GAAGGGGAGAGTGGAATTCCATGTGTAGCGGTGAAATGCGTAGATATATGGAGGAACACC  660
                ||||||||||||||||||||||||||||||||||||||||||||||||||||||||||||
Sbjct  1415430  GAAGGGGAGAGTGGAATTCCATGTGTAGCGGTGAAATGCGTAGATATATGGAGGAACACC  1415489

Query  661      GGTGGCGAAAGCGGCTCTCTGGTCTGTAACTGACGCTGAGGCTCGAAAGCGTGGGGAGCA  720
                ||||||||||||||||||||||||||||||||||||||||||||||||||||||||||||
Sbjct  1415490  GGTGGCGAAAGCGGCTCTCTGGTCTGTAACTGACGCTGAGGCTCGAAAGCGTGGGGAGCA  1415549

Query  721      AACAGGATTAGATACCCTGGTAGTCCACGCCGTAAACGATGAGTGCTAGGTGTTAGGCCC  780
                ||||||||||||||||||||||||||||||||||||||||||||||||||||||||||||
Sbjct  1415550  AACAGGATTAGATACCCTGGTAGTCCACGCCGTAAACGATGAGTGCTAGGTGTTAGGCCC  1415609

Query  781      TTTCCGGGGCTTAGTGCCGCAGCTAACGCATTAAGCACTC  820
                ||||||||||||||||||||||||||||||||||||||||
Sbjct  1415610  TTTCCGGGGCTTAGTGCCGCAGCTAACGCATTAAGCACTC  1415649
```

Range 2: 1420664 to 1421483

Score	Expect	Identities	Gaps	Strand	Frame
1515 bits(820)	0.0()	820/820(100%)	0/820(0%)	Plus/Plus	

Features:

```
Query  1        CGCTGATGTTTGGTGTTTACACTAGACTGATGAGTTGCGAACGGGTGAGTAACGCGTAGG  60
                ||||||||||||||||||||||||||||||||||||||||||||||||||||||||||||
Sbjct  1420664  CGCTGATGTTTGGTGTTTACACTAGACTGATGAGTTGCGAACGGGTGAGTAACGCGTAGG  1420723

Query  61       TAACCTGCCTCATAGCGGGGGATAACTATTGGAAACGATAGCTAATACCGCATAAGAGTA  120
                ||||||||||||||||||||||||||||||||||||||||||||||||||||||||||||
Sbjct  1420724  TAACCTGCCTCATAGCGGGGGATAACTATTGGAAACGATAGCTAATACCGCATAAGAGTA  1420783

Query  121      ATTAACACATGTTAGTTATTTAAAAGGAGCAATTGCTTCACTGTGAGATGGACCTGCGTT  180
                ||||||||||||||||||||||||||||||||||||||||||||||||||||||||||||
Sbjct  1420784  ATTAACACATGTTAGTTATTTAAAAGGAGCAATTGCTTCACTGTGAGATGGACCTGCGTT  1420843

Query  181      GTATTAGCTAGTTGGTGAGGTAAAGGCTCACCAAGGCGACGATACATAGCCGACCTGAGA  240
                ||||||||||||||||||||||||||||||||||||||||||||||||||||||||||||
Sbjct  1420844  GTATTAGCTAGTTGGTGAGGTAAAGGCTCACCAAGGCGACGATACATAGCCGACCTGAGA  1420903

Query  241      GGGTGATCGGCCACACTGGGACTGAGACACGGCCCAGACTCCTACGGGAGGCAGCAGTAG  300
                ||||||||||||||||||||||||||||||||||||||||||||||||||||||||||||
Sbjct  1420904  GGGTGATCGGCCACACTGGGACTGAGACACGGCCCAGACTCCTACGGGAGGCAGCAGTAG  1420963

Query  301      GGAATCTTCGGCAATGGACGGAAGTCTGACCGAGCAACGCCGCGTGAGTGAAGAAGGTTT  360
                ||||||||||||||||||||||||||||||||||||||||||||||||||||||||||||
Sbjct  1420964  GGAATCTTCGGCAATGGACGGAAGTCTGACCGAGCAACGCCGCGTGAGTGAAGAAGGTTT  1421023

Query  361      TCGGATCGTAAAGCTCTGTTGTTAGAGAAGAACGTTGGTAGGAGTGGAAAATCTACCAAG  420
                ||||||||||||||||||||||||||||||||||||||||||||||||||||||||||||
Sbjct  1421024  TCGGATCGTAAAGCTCTGTTGTTAGAGAAGAACGTTGGTAGGAGTGGAAAATCTACCAAG  1421083

Query  421      TGACGGTAACTAACCAGAAAGGGACGGCTAACTACGTGCCAGCAGCCGCGGTAATACGTA  480
                ||||||||||||||||||||||||||||||||||||||||||||||||||||||||||||
Sbjct  1421084  TGACGGTAACTAACCAGAAAGGGACGGCTAACTACGTGCCAGCAGCCGCGGTAATACGTA  1421143

Query  481      GGTCCCGAGCGTTGTCCGGATTTATTGGGCGTAAAGCGAGCGCAGGCGGTTCTTTAAGTC  540
                ||||||||||||||||||||||||||||||||||||||||||||||||||||||||||||
Sbjct  1421144  GGTCCCGAGCGTTGTCCGGATTTATTGGGCGTAAAGCGAGCGCAGGCGGTTCTTTAAGTC  1421203

Query  541      TGAAGTTAAAGGCAGTGGCTTAACCATTGTACGCTTTGGAAACTGGAGGACTTGAGTGCA  600
                ||||||||||||||||||||||||||||||||||||||||||||||||||||||||||||
Sbjct  1421204  TGAAGTTAAAGGCAGTGGCTTAACCATTGTACGCTTTGGAAACTGGAGGACTTGAGTGCA  1421263

Query  601      GAAGGGGAGAGTGGAATTCCATGTGTAGCGGTGAAATGCGTAGATATATGGAGGAACACC  660
                ||||||||||||||||||||||||||||||||||||||||||||||||||||||||||||
Sbjct  1421264  GAAGGGGAGAGTGGAATTCCATGTGTAGCGGTGAAATGCGTAGATATATGGAGGAACACC  1421323

Query  661      GGTGGCGAAAGCGGCTCTCTGGTCTGTAACTGACGCTGAGGCTCGAAAGCGTGGGGAGCA  720
                ||||||||||||||||||||||||||||||||||||||||||||||||||||||||||||
Sbjct  1421324  GGTGGCGAAAGCGGCTCTCTGGTCTGTAACTGACGCTGAGGCTCGAAAGCGTGGGGAGCA  1421383

Query  721      AACAGGATTAGATACCCTGGTAGTCCACGCCGTAAACGATGAGTGCTAGGTGTTAGGCCC  780
                ||||||||||||||||||||||||||||||||||||||||||||||||||||||||||||
Sbjct  1421384  AACAGGATTAGATACCCTGGTAGTCCACGCCGTAAACGATGAGTGCTAGGTGTTAGGCCC  1421443

Query  781      TTTCCGGGGCTTAGTGCCGCAGCTAACGCATTAAGCACTC  820
                ||||||||||||||||||||||||||||||||||||||||
Sbjct  1421444  TTTCCGGGGCTTAGTGCCGCAGCTAACGCATTAAGCACTC  1421483
```

Range 3: 1489468 to 1490287

Score	Expect	Identities	Gaps	Strand	Frame
1515 bits(820)	0.0()	820/820(100%)	0/820(0%)	Plus/Plus	

Features:

```
Query  1        CGCTGATGTTTGGTGTTTACACTAGACTGATGAGTTGCGAACGGGTGAGTAACGCGTAGG  60
                ||||||||||||||||||||||||||||||||||||||||||||||||||||||||||||
Sbjct  1489468  CGCTGATGTTTGGTGTTTACACTAGACTGATGAGTTGCGAACGGGTGAGTAACGCGTAGG  1489527
Query  61       TAACCTGCCTCATAGCGGGGGATAACTATTGGAAACGATAGCTAATACCGCATAAGAGTA  120
                ||||||||||||||||||||||||||||||||||||||||||||||||||||||||||||
Sbjct  1489528  TAACCTGCCTCATAGCGGGGGATAACTATTGGAAACGATAGCTAATACCGCATAAGAGTA  1489587
Query  121      ATTAACACATGTTAGTTATTTAAAAGGAGCAATTGCTTCACTGTGAGATGGACCTGCGTT  180
                ||||||||||||||||||||||||||||||||||||||||||||||||||||||||||||
Sbjct  1489588  ATTAACACATGTTAGTTATTTAAAAGGAGCAATTGCTTCACTGTGAGATGGACCTGCGTT  1489647
Query  181      GTATTAGCTAGTTGGTGAGGTAAAGGCTCACCAAGGCGACGATACATAGCCGACCTGAGA  240
                ||||||||||||||||||||||||||||||||||||||||||||||||||||||||||||
Sbjct  1489648  GTATTAGCTAGTTGGTGAGGTAAAGGCTCACCAAGGCGACGATACATAGCCGACCTGAGA  1489707
Query  241      GGGTGATCGGCCACACTGGGACTGAGACACGGCCCAGACTCCTACGGGAGGCAGCAGTAG  300
                ||||||||||||||||||||||||||||||||||||||||||||||||||||||||||||
Sbjct  1489708  GGGTGATCGGCCACACTGGGACTGAGACACGGCCCAGACTCCTACGGGAGGCAGCAGTAG  1489767
Query  301      GGAATCTTCGGCAATGGACGGAAGTCTGACCGAGCAACGCCGCGTGAGTGAAGAAGGTTT  360
                ||||||||||||||||||||||||||||||||||||||||||||||||||||||||||||
Sbjct  1489768  GGAATCTTCGGCAATGGACGGAAGTCTGACCGAGCAACGCCGCGTGAGTGAAGAAGGTTT  1489827
Query  361      TCGGATCGTAAAGCTCTGTTGTTAGAGAAGAACGTTGGTAGGAGTGGAAAATCTACCAAG  420
                ||||||||||||||||||||||||||||||||||||||||||||||||||||||||||||
Sbjct  1489828  TCGGATCGTAAAGCTCTGTTGTTAGAGAAGAACGTTGGTAGGAGTGGAAAATCTACCAAG  1489887
Query  421      TGACGGTAACTAACCAGAAAGGGACGGCTAACTACGTGCCAGCAGCCGCGGTAATACGTA  480
                ||||||||||||||||||||||||||||||||||||||||||||||||||||||||||||
Sbjct  1489888  TGACGGTAACTAACCAGAAAGGGACGGCTAACTACGTGCCAGCAGCCGCGGTAATACGTA  1489947
Query  481      GGTCCCGAGCGTTGTCCGGATTTATTGGGCGTAAAGCGAGCGCAGGCGGTTCTTTAAGTC  540
                ||||||||||||||||||||||||||||||||||||||||||||||||||||||||||||
Sbjct  1489948  GGTCCCGAGCGTTGTCCGGATTTATTGGGCGTAAAGCGAGCGCAGGCGGTTCTTTAAGTC  1490007
Query  541      TGAAGTTAAAGGCAGTGGCTTAACCATTGTACGCTTTGGAAACTGGAGGACTTGAGTGCA  600
                ||||||||||||||||||||||||||||||||||||||||||||||||||||||||||||
Sbjct  1490008  TGAAGTTAAAGGCAGTGGCTTAACCATTGTACGCTTTGGAAACTGGAGGACTTGAGTGCA  1490067
Query  601      GAAGGGGAGAGTGGAATTCCATGTGTAGCGGTGAAATGCGTAGATATATGGAGGAACACC  660
                ||||||||||||||||||||||||||||||||||||||||||||||||||||||||||||
Sbjct  1490068  GAAGGGGAGAGTGGAATTCCATGTGTAGCGGTGAAATGCGTAGATATATGGAGGAACACC  1490127
Query  661      GGTGGCGAAAGCGGCTCTCTGGTCTGTAACTGACGCTGAGGCTCGAAAGCGTGGGGAGCA  720
                ||||||||||||||||||||||||||||||||||||||||||||||||||||||||||||
Sbjct  1490128  GGTGGCGAAAGCGGCTCTCTGGTCTGTAACTGACGCTGAGGCTCGAAAGCGTGGGGAGCA  1490187
Query  721      AACAGGATTAGATACCCTGGTAGTCCACGCCGTAAACGATGAGTGCTAGGTGTTAGGCCC  780
                ||||||||||||||||||||||||||||||||||||||||||||||||||||||||||||
Sbjct  1490188  AACAGGATTAGATACCCTGGTAGTCCACGCCGTAAACGATGAGTGCTAGGTGTTAGGCCC  1490247
Query  781      TTTCCGGGGCTTAGTGCCGCAGCTAACGCATTAAGCACTC  820
                ||||||||||||||||||||||||||||||||||||||||
Sbjct  1490248  TTTCCGGGGCTTAGTGCCGCAGCTAACGCATTAAGCACTC  1490287
```

Range 4: 1563498 to 1564317

Score	Expect	Identities	Gaps	Strand	Frame
1515 bits(820)	0.0()	820/820(100%)	0/820(0%)	Plus/Plus	

Features:

```
Query  1        CGCTGATGTTTGGTGTTTACACTAGACTGATGAGTTGCGAACGGGTGAGTAACGCGTAGG  60
                ||||||||||||||||||||||||||||||||||||||||||||||||||||||||||||
Sbjct  1563498  CGCTGATGTTTGGTGTTTACACTAGACTGATGAGTTGCGAACGGGTGAGTAACGCGTAGG  1563557
Query  61       TAACCTGCCTCATAGCGGGGGATAACTATTGGAAACGATAGCTAATACCGCATAAGAGTA  120
                ||||||||||||||||||||||||||||||||||||||||||||||||||||||||||||
Sbjct  1563558  TAACCTGCCTCATAGCGGGGGATAACTATTGGAAACGATAGCTAATACCGCATAAGAGTA  1563617
Query  121      ATTAACACATGTTAGTTATTTAAAAGGAGCAATTGCTTCACTGTGAGATGGACCTGCGTT  180
                ||||||||||||||||||||||||||||||||||||||||||||||||||||||||||||
Sbjct  1563618  ATTAACACATGTTAGTTATTTAAAAGGAGCAATTGCTTCACTGTGAGATGGACCTGCGTT  1563677
Query  181      GTATTAGCTAGTTGGTGAGGTAAAGGCTCACCAAGGCGACGATACATAGCCGACCTGAGA  240
                ||||||||||||||||||||||||||||||||||||||||||||||||||||||||||||
Sbjct  1563678  GTATTAGCTAGTTGGTGAGGTAAAGGCTCACCAAGGCGACGATACATAGCCGACCTGAGA  1563737
Query  241      GGGTGATCGGCCACACTGGGACTGAGACACGGCCCAGACTCCTACGGGAGGCAGCAGTAG  300
                ||||||||||||||||||||||||||||||||||||||||||||||||||||||||||||
Sbjct  1563738  GGGTGATCGGCCACACTGGGACTGAGACACGGCCCAGACTCCTACGGGAGGCAGCAGTAG  1563797
Query  301      GGAATCTTCGGCAATGGACGGAAGTCTGACCGAGCAACGCCGCGTGAGTGAAGAAGGTTT  360
                ||||||||||||||||||||||||||||||||||||||||||||||||||||||||||||
Sbjct  1563798  GGAATCTTCGGCAATGGACGGAAGTCTGACCGAGCAACGCCGCGTGAGTGAAGAAGGTTT  1563857
Query  361      TCGGATCGTAAAGCTCTGTTGTTAGAGAAGAACGTTGGTAGGAGTGGAAAATCTACCAAG  420
                ||||||||||||||||||||||||||||||||||||||||||||||||||||||||||||
Sbjct  1563858  TCGGATCGTAAAGCTCTGTTGTTAGAGAAGAACGTTGGTAGGAGTGGAAAATCTACCAAG  1563917
Query  421      TGACGGTAACTAACCAGAAAGGGACGGCTAACTACGTGCCAGCAGCCGCGGTAATACGTA  480
                ||||||||||||||||||||||||||||||||||||||||||||||||||||||||||||
Sbjct  1563918  TGACGGTAACTAACCAGAAAGGGACGGCTAACTACGTGCCAGCAGCCGCGGTAATACGTA  1563977
Query  481      GGTCCCGAGCGTTGTCCGGATTTATTGGGCGTAAAGCGAGCGCAGGCGGTTCTTTAAGTC  540
                ||||||||||||||||||||||||||||||||||||||||||||||||||||||||||||
Sbjct  1563978  GGTCCCGAGCGTTGTCCGGATTTATTGGGCGTAAAGCGAGCGCAGGCGGTTCTTTAAGTC  1564037
Query  541      TGAAGTTAAAGGCAGTGGCTTAACCATTGTACGCTTTGGAAACTGGAGGACTTGAGTGCA  600
                ||||||||||||||||||||||||||||||||||||||||||||||||||||||||||||
Sbjct  1564038  TGAAGTTAAAGGCAGTGGCTTAACCATTGTACGCTTTGGAAACTGGAGGACTTGAGTGCA  1564097
Query  601      GAAGGGGAGAGTGGAATTCCATGTGTAGCGGTGAAATGCGTAGATATATGGAGGAACACC  660
                ||||||||||||||||||||||||||||||||||||||||||||||||||||||||||||
Sbjct  1564098  GAAGGGGAGAGTGGAATTCCATGTGTAGCGGTGAAATGCGTAGATATATGGAGGAACACC  1564157
Query  661      GGTGGCGAAAGCGGCTCTCTGGTCTGTAACTGACGCTGAGGCTCGAAAGCGTGGGGAGCA  720
                ||||||||||||||||||||||||||||||||||||||||||||||||||||||||||||
Sbjct  1564158  GGTGGCGAAAGCGGCTCTCTGGTCTGTAACTGACGCTGAGGCTCGAAAGCGTGGGGAGCA  1564217
Query  721      AACAGGATTAGATACCCTGGTAGTCCACGCCGTAAACGATGAGTGCTAGGTGTTAGGCCC  780
                ||||||||||||||||||||||||||||||||||||||||||||||||||||||||||||
Sbjct  1564218  AACAGGATTAGATACCCTGGTAGTCCACGCCGTAAACGATGAGTGCTAGGTGTTAGGCCC  1564277
Query  781      TTTCCGGGGCTTAGTGCCGCAGCTAACGCATTAAGCACTC  820
                ||||||||||||||||||||||||||||||||||||||||
Sbjct  1564278  TTTCCGGGGCTTAGTGCCGCAGCTAACGCATTAAGCACTC  1564317
```

Range 5: 1671609 to 1672428

Score	Expect	Identities	Gaps	Strand	Frame
1515 bits(820)	0.0()	820/820(100%)	0/820(0%)	Plus/Plus	

Features:

```
Query  1        CGCTGATGTTTGGTGTTTACACTAGACTGATGAGTTGCGAACGGGTGAGTAACGCGTAGG  60
                ||||||||||||||||||||||||||||||||||||||||||||||||||||||||||||
Sbjct  1671609  CGCTGATGTTTGGTGTTTACACTAGACTGATGAGTTGCGAACGGGTGAGTAACGCGTAGG  1671668
Query  61       TAACCTGCCTCATAGCGGGGGATAACTATTGGAAACGATAGCTAATACCGCATAAGAGTA  120
                ||||||||||||||||||||||||||||||||||||||||||||||||||||||||||||
Sbjct  1671669  TAACCTGCCTCATAGCGGGGGATAACTATTGGAAACGATAGCTAATACCGCATAAGAGTA  1671728
Query  121      ATTAACACATGTTAGTTATTTAAAAGGAGCAATTGCTTCACTGTGAGATGGACCTGCGTT  180
                ||||||||||||||||||||||||||||||||||||||||||||||||||||||||||||
Sbjct  1671729  ATTAACACATGTTAGTTATTTAAAAGGAGCAATTGCTTCACTGTGAGATGGACCTGCGTT  1671788
Query  181      GTATTAGCTAGTTGGTGAGGTAAAGGCTCACCAAGGCGACGATACATAGCCGACCTGAGA  240
                ||||||||||||||||||||||||||||||||||||||||||||||||||||||||||||
Sbjct  1671789  GTATTAGCTAGTTGGTGAGGTAAAGGCTCACCAAGGCGACGATACATAGCCGACCTGAGA  1671848
Query  241      GGGTGATCGGCCACACTGGGACTGAGACACGGCCCAGACTCCTACGGGAGGCAGCAGTAG  300
                ||||||||||||||||||||||||||||||||||||||||||||||||||||||||||||
Sbjct  1671849  GGGTGATCGGCCACACTGGGACTGAGACACGGCCCAGACTCCTACGGGAGGCAGCAGTAG  1671908
Query  301      GGAATCTTCGGCAATGGACGGAAGTCTGACCGAGCAACGCCGCGTGAGTGAAGAAGGTTT  360
                ||||||||||||||||||||||||||||||||||||||||||||||||||||||||||||
Sbjct  1671909  GGAATCTTCGGCAATGGACGGAAGTCTGACCGAGCAACGCCGCGTGAGTGAAGAAGGTTT  1671968
```

```
Query  361      TCGGATCGTAAAGCTCTGTTGTTAGAGAAGAACGTTGGTAGGAGTGGAAAATCTACCAAG  420
                ||||||||||||||||||||||||||||||||||||||||||||||||||||||||||||
Sbjct  1671969  TCGGATCGTAAAGCTCTGTTGTTAGAGAAGAACGTTGGTAGGAGTGGAAAATCTACCAAG  1672028

Query  421      TGACGGTAACTAACCAGAAAGGGACGGCTAACTACGTGCCAGCAGCCGCGGTAATACGTA  480
                ||||||||||||||||||||||||||||||||||||||||||||||||||||||||||||
Sbjct  1672029  TGACGGTAACTAACCAGAAAGGGACGGCTAACTACGTGCCAGCAGCCGCGGTAATACGTA  1672088

Query  481      GGTCCCGAGCGTTGTCCGGATTTATTGGGCGTAAAGCGAGCGCAGGCGGTTCTTTAAGTC  540
                ||||||||||||||||||||||||||||||||||||||||||||||||||||||||||||
Sbjct  1672089  GGTCCCGAGCGTTGTCCGGATTTATTGGGCGTAAAGCGAGCGCAGGCGGTTCTTTAAGTC  1672148

Query  541      TGAAGTTAAAGGCAGTGGCTTAACCATTGTACGCTTTGGAAACTGGAGGACTTGAGTGCA  600
                ||||||||||||||||||||||||||||||||||||||||||||||||||||||||||||
Sbjct  1672149  TGAAGTTAAAGGCAGTGGCTTAACCATTGTACGCTTTGGAAACTGGAGGACTTGAGTGCA  1672208

Query  601      GAAGGGGAGAGTGGAATTCCATGTGTAGCGGTGAAATGCGTAGATATATGGAGGAACACC  660
                ||||||||||||||||||||||||||||||||||||||||||||||||||||||||||||
Sbjct  1672209  GAAGGGGAGAGTGGAATTCCATGTGTAGCGGTGAAATGCGTAGATATATGGAGGAACACC  1672268

Query  661      GGTGGCGAAAGCGGCTCTCTGGTCTGTAACTGACGCTGAGGCTCGAAAGCGTGGGGAGCA  720
                ||||||||||||||||||||||||||||||||||||||||||||||||||||||||||||
Sbjct  1672269  GGTGGCGAAAGCGGCTCTCTGGTCTGTAACTGACGCTGAGGCTCGAAAGCGTGGGGAGCA  1672328

Query  721      AACAGGATTAGATACCCTGGTAGTCCACGCCGTAAACGATGAGTGCTAGGTGTTAGGCCC  780
                ||||||||||||||||||||||||||||||||||||||||||||||||||||||||||||
Sbjct  1672329  AACAGGATTAGATACCCTGGTAGTCCACGCCGTAAACGATGAGTGCTAGGTGTTAGGCCC  1672388

Query  781      TTTCCGGGGCTTAGTGCCGCAGCTAACGCATTAAGCACTC  820
                ||||||||||||||||||||||||||||||||||||||||
Sbjct  1672389  TTTCCGGGGCTTAGTGCCGCAGCTAACGCATTAAGCACTC  1672428
```

Range 6: 1768948 to 1769767

Score	Expect	Identities	Gaps	Strand	Frame
1515 bits(820)	0.0()	820/820(100%)	0/820(0%)	Plus/Plus	

Features:

```
Query  1        CGCTGATGTTTGGTGTTTACACTAGACTGATGAGTTGCGAACGGGTGAGTAACGCGTAGG  60
                ||||||||||||||||||||||||||||||||||||||||||||||||||||||||||||
Sbjct  1768948  CGCTGATGTTTGGTGTTTACACTAGACTGATGAGTTGCGAACGGGTGAGTAACGCGTAGG  1769007

Query  61       TAACCTGCCTCATAGCGGGGGATAACTATTGGAAACGATAGCTAATACCGCATAAGAGTA  120
                ||||||||||||||||||||||||||||||||||||||||||||||||||||||||||||
Sbjct  1769008  TAACCTGCCTCATAGCGGGGGATAACTATTGGAAACGATAGCTAATACCGCATAAGAGTA  1769067

Query  121      ATTAACACATGTTAGTTATTTAAAAGGAGCAATTGCTTCACTGTGAGATGGACCTGCGTT  180
                ||||||||||||||||||||||||||||||||||||||||||||||||||||||||||||
Sbjct  1769068  ATTAACACATGTTAGTTATTTAAAAGGAGCAATTGCTTCACTGTGAGATGGACCTGCGTT  1769127

Query  181      GTATTAGCTAGTTGGTGAGGTAAAGGCTCACCAAGGCGACGATACATAGCCGACCTGAGA  240
                ||||||||||||||||||||||||||||||||||||||||||||||||||||||||||||
Sbjct  1769128  GTATTAGCTAGTTGGTGAGGTAAAGGCTCACCAAGGCGACGATACATAGCCGACCTGAGA  1769187

Query  241      GGGTGATCGGCCACACTGGGACTGAGACACGGCCCAGACTCCTACGGGAGGCAGCAGTAG  300
                ||||||||||||||||||||||||||||||||||||||||||||||||||||||||||||
Sbjct  1769188  GGGTGATCGGCCACACTGGGACTGAGACACGGCCCAGACTCCTACGGGAGGCAGCAGTAG  1769247

Query  301      GGAATCTTCGGCAATGGACGGAAGTCTGACCGAGCAACGCCGCGTGAGTGAAGAAGGTTT  360
                ||||||||||||||||||||||||||||||||||||||||||||||||||||||||||||
Sbjct  1769248  GGAATCTTCGGCAATGGACGGAAGTCTGACCGAGCAACGCCGCGTGAGTGAAGAAGGTTT  1769307

Query  361      TCGGATCGTAAAGCTCTGTTGTTAGAGAAGAACGTTGGTAGGAGTGGAAAATCTACCAAG  420
                ||||||||||||||||||||||||||||||||||||||||||||||||||||||||||||
Sbjct  1769308  TCGGATCGTAAAGCTCTGTTGTTAGAGAAGAACGTTGGTAGGAGTGGAAAATCTACCAAG  1769367

Query  421      TGACGGTAACTAACCAGAAAGGGACGGCTAACTACGTGCCAGCAGCCGCGGTAATACGTA  480
                ||||||||||||||||||||||||||||||||||||||||||||||||||||||||||||
Sbjct  1769368  TGACGGTAACTAACCAGAAAGGGACGGCTAACTACGTGCCAGCAGCCGCGGTAATACGTA  1769427

Query  481      GGTCCCGAGCGTTGTCCGGATTTATTGGGCGTAAAGCGAGCGCAGGCGGTTCTTTAAGTC  540
                ||||||||||||||||||||||||||||||||||||||||||||||||||||||||||||
Sbjct  1769428  GGTCCCGAGCGTTGTCCGGATTTATTGGGCGTAAAGCGAGCGCAGGCGGTTCTTTAAGTC  1769487

Query  541      TGAAGTTAAAGGCAGTGGCTTAACCATTGTACGCTTTGGAAACTGGAGGACTTGAGTGCA  600
                ||||||||||||||||||||||||||||||||||||||||||||||||||||||||||||
Sbjct  1769488  TGAAGTTAAAGGCAGTGGCTTAACCATTGTACGCTTTGGAAACTGGAGGACTTGAGTGCA  1769547

Query  601      GAAGGGGAGAGTGGAATTCCATGTGTAGCGGTGAAATGCGTAGATATATGGAGGAACACC  660
                ||||||||||||||||||||||||||||||||||||||||||||||||||||||||||||
Sbjct  1769548  GAAGGGGAGAGTGGAATTCCATGTGTAGCGGTGAAATGCGTAGATATATGGAGGAACACC  1769607

Query  661      GGTGGCGAAAGCGGCTCTCTGGTCTGTAACTGACGCTGAGGCTCGAAAGCGTGGGGAGCA  720
                ||||||||||||||||||||||||||||||||||||||||||||||||||||||||||||
Sbjct  1769608  GGTGGCGAAAGCGGCTCTCTGGTCTGTAACTGACGCTGAGGCTCGAAAGCGTGGGGAGCA  1769667

Query  721      AACAGGATTAGATACCCTGGTAGTCCACGCCGTAAACGATGAGTGCTAGGTGTTAGGCCC  780
                ||||||||||||||||||||||||||||||||||||||||||||||||||||||||||||
Sbjct  1769668  AACAGGATTAGATACCCTGGTAGTCCACGCCGTAAACGATGAGTGCTAGGTGTTAGGCCC  1769727

Query  781      TTTCCGGGGCTTAGTGCCGCAGCTAACGCATTAAGCACTC  820
                ||||||||||||||||||||||||||||||||||||||||
Sbjct  1769728  TTTCCGGGGCTTAGTGCCGCAGCTAACGCATTAAGCACTC  1769767
```

Range 7: 1838091 to 1838910

Score	Expect	Identities	Gaps	Strand	Frame
1515 bits(820)	0.0()	820/820(100%)	0/820(0%)	Plus/Plus	

Features:

```
Query  1        CGCTGATGTTTGGTGTTTACACTAGACTGATGAGTTGCGAACGGGTGAGTAACGCGTAGG  60
                ||||||||||||||||||||||||||||||||||||||||||||||||||||||||||||
Sbjct  1838091  CGCTGATGTTTGGTGTTTACACTAGACTGATGAGTTGCGAACGGGTGAGTAACGCGTAGG  1838150

Query  61       TAACCTGCCTCATAGCGGGGGATAACTATTGGAAACGATAGCTAATACCGCATAAGAGTA  120
                ||||||||||||||||||||||||||||||||||||||||||||||||||||||||||||
Sbjct  1838151  TAACCTGCCTCATAGCGGGGGATAACTATTGGAAACGATAGCTAATACCGCATAAGAGTA  1838210

Query  121      ATTAACACATGTTAGTTATTTAAAAGGAGCAATTGCTTCACTGTGAGATGGACCTGCGTT  180
                ||||||||||||||||||||||||||||||||||||||||||||||||||||||||||||
Sbjct  1838211  ATTAACACATGTTAGTTATTTAAAAGGAGCAATTGCTTCACTGTGAGATGGACCTGCGTT  1838270

Query  181      GTATTAGCTAGTTGGTGAGGTAAAGGCTCACCAAGGCGACGATACATAGCCGACCTGAGA  240
                ||||||||||||||||||||||||||||||||||||||||||||||||||||||||||||
Sbjct  1838271  GTATTAGCTAGTTGGTGAGGTAAAGGCTCACCAAGGCGACGATACATAGCCGACCTGAGA  1838330

Query  241      GGGTGATCGGCCACACTGGGACTGAGACACGGCCCAGACTCCTACGGGAGGCAGCAGTAG  300
                ||||||||||||||||||||||||||||||||||||||||||||||||||||||||||||
Sbjct  1838331  GGGTGATCGGCCACACTGGGACTGAGACACGGCCCAGACTCCTACGGGAGGCAGCAGTAG  1838390

Query  301      GGAATCTTCGGCAATGGACGGAAGTCTGACCGAGCAACGCCGCGTGAGTGAAGAAGGTTT  360
                ||||||||||||||||||||||||||||||||||||||||||||||||||||||||||||
Sbjct  1838391  GGAATCTTCGGCAATGGACGGAAGTCTGACCGAGCAACGCCGCGTGAGTGAAGAAGGTTT  1838450

Query  361      TCGGATCGTAAAGCTCTGTTGTTAGAGAAGAACGTTGGTAGGAGTGGAAAATCTACCAAG  420
                ||||||||||||||||||||||||||||||||||||||||||||||||||||||||||||
Sbjct  1838451  TCGGATCGTAAAGCTCTGTTGTTAGAGAAGAACGTTGGTAGGAGTGGAAAATCTACCAAG  1838510

Query  421      TGACGGTAACTAACCAGAAAGGGACGGCTAACTACGTGCCAGCAGCCGCGGTAATACGTA  480
                ||||||||||||||||||||||||||||||||||||||||||||||||||||||||||||
Sbjct  1838511  TGACGGTAACTAACCAGAAAGGGACGGCTAACTACGTGCCAGCAGCCGCGGTAATACGTA  1838570

Query  481      GGTCCCGAGCGTTGTCCGGATTTATTGGGCGTAAAGCGAGCGCAGGCGGTTCTTTAAGTC  540
                ||||||||||||||||||||||||||||||||||||||||||||||||||||||||||||
Sbjct  1838571  GGTCCCGAGCGTTGTCCGGATTTATTGGGCGTAAAGCGAGCGCAGGCGGTTCTTTAAGTC  1838630

Query  541      TGAAGTTAAAGGCAGTGGCTTAACCATTGTACGCTTTGGAAACTGGAGGACTTGAGTGCA  600
                ||||||||||||||||||||||||||||||||||||||||||||||||||||||||||||
Sbjct  1838631  TGAAGTTAAAGGCAGTGGCTTAACCATTGTACGCTTTGGAAACTGGAGGACTTGAGTGCA  1838690

Query  601      GAAGGGGAGAGTGGAATTCCATGTGTAGCGGTGAAATGCGTAGATATATGGAGGAACACC  660
                ||||||||||||||||||||||||||||||||||||||||||||||||||||||||||||
Sbjct  1838691  GAAGGGGAGAGTGGAATTCCATGTGTAGCGGTGAAATGCGTAGATATATGGAGGAACACC  1838750

Query  661      GGTGGCGAAAGCGGCTCTCTGGTCTGTAACTGACGCTGAGGCTCGAAAGCGTGGGGAGCA  720
                ||||||||||||||||||||||||||||||||||||||||||||||||||||||||||||
```

```
Sbjct  1838751  GGTGGCGAAAGCGGCTCTCTGGTCTGTAACTGACGCTGAGGCTCGAAAGCGTGGGGAGCA  1838810

Query  721      AACAGGATTAGATACCCTGGTAGTCCACGCCGTAAACGATGAGTGCTAGGTGTTAGGCCC  780
                ||||||||||||||||||||||||||||||||||||||||||||||||||||||||||||
Sbjct  1838811  AACAGGATTAGATACCCTGGTAGTCCACGCCGTAAACGATGAGTGCTAGGTGTTAGGCCC  1838870

Query  781      TTTCCGGGGCTTAGTGCCGCAGCTAACGCATTAAGCACTC  820
                ||||||||||||||||||||||||||||||||||||||||
Sbjct  1838871  TTTCCGGGGCTTAGTGCCGCAGCTAACGCATTAAGCACTC  1838910
```

Streptococcus agalactiae strain B507 chromosome, complete genome

Sequence ID: **CP021771.1** Length: 2082479 Number of Matches: 7

Range 1: 79703 to 80522

Score	Expect	Identities	Gaps	Strand	Frame
1515 bits(820)	0.0()	820/820(100%)	0/820(0%)	Plus/Plus	

Features:

```
Query  1      CGCTGATGTTTGGTGTTTACACTAGACTGATGAGTTGCGAACGGGTGAGTAACGCGTAGG  60
              ||||||||||||||||||||||||||||||||||||||||||||||||||||||||||||
Sbjct  79703  CGCTGATGTTTGGTGTTTACACTAGACTGATGAGTTGCGAACGGGTGAGTAACGCGTAGG  79762

Query  61     TAACCTGCCTCATAGCGGGGGATAACTATTGGAAACGATAGCTAATACCGCATAAGAGTA  120
              ||||||||||||||||||||||||||||||||||||||||||||||||||||||||||||
Sbjct  79763  TAACCTGCCTCATAGCGGGGGATAACTATTGGAAACGATAGCTAATACCGCATAAGAGTA  79822

Query  121    ATTAACACATGTTAGTTATTTAAAAGGAGCAATTGCTTCACTGTGAGATGGACCTGCGTT  180
              ||||||||||||||||||||||||||||||||||||||||||||||||||||||||||||
Sbjct  79823  ATTAACACATGTTAGTTATTTAAAAGGAGCAATTGCTTCACTGTGAGATGGACCTGCGTT  79882

Query  181    GTATTAGCTAGTTGGTGAGGTAAAGGCTCACCAAGGCGACGATACATAGCCGACCTGAGA  240
              ||||||||||||||||||||||||||||||||||||||||||||||||||||||||||||
Sbjct  79883  GTATTAGCTAGTTGGTGAGGTAAAGGCTCACCAAGGCGACGATACATAGCCGACCTGAGA  79942

Query  241    GGGTGATCGGCCACACTGGGACTGAGACACGGCCCAGACTCCTACGGGAGGCAGCAGTAG  300
              ||||||||||||||||||||||||||||||||||||||||||||||||||||||||||||
Sbjct  79943  GGGTGATCGGCCACACTGGGACTGAGACACGGCCCAGACTCCTACGGGAGGCAGCAGTAG  80002

Query  301    GGAATCTTCGGCAATGGACGGAAGTCTGACCGAGCAACGCCGCGTGAGTGAAGAAGGTTT  360
              ||||||||||||||||||||||||||||||||||||||||||||||||||||||||||||
Sbjct  80003  GGAATCTTCGGCAATGGACGGAAGTCTGACCGAGCAACGCCGCGTGAGTGAAGAAGGTTT  80062

Query  361    TCGGATCGTAAAGCTCTGTTGTTAGAGAAGAACGTTGGTAGGAGTGGAAAATCTACCAAG  420
              ||||||||||||||||||||||||||||||||||||||||||||||||||||||||||||
Sbjct  80063  TCGGATCGTAAAGCTCTGTTGTTAGAGAAGAACGTTGGTAGGAGTGGAAAATCTACCAAG  80122

Query  421    TGACGGTAACTAACCAGAAAGGGACGGCTAACTACGTGCCAGCAGCCGCGGTAATACGTA  480
              ||||||||||||||||||||||||||||||||||||||||||||||||||||||||||||
Sbjct  80123  TGACGGTAACTAACCAGAAAGGGACGGCTAACTACGTGCCAGCAGCCGCGGTAATACGTA  80182

Query  481    GGTCCCGAGCGTTGTCCGGATTTATTGGGCGTAAAGCGAGCGCAGGCGGTTCTTTAAGTC  540
              ||||||||||||||||||||||||||||||||||||||||||||||||||||||||||||
Sbjct  80183  GGTCCCGAGCGTTGTCCGGATTTATTGGGCGTAAAGCGAGCGCAGGCGGTTCTTTAAGTC  80242

Query  541    TGAAGTTAAAGGCAGTGGCTTAACCATTGTACGCTTTGGAAACTGGAGGACTTGAGTGCA  600
              ||||||||||||||||||||||||||||||||||||||||||||||||||||||||||||
Sbjct  80243  TGAAGTTAAAGGCAGTGGCTTAACCATTGTACGCTTTGGAAACTGGAGGACTTGAGTGCA  80302

Query  601    GAAGGGGAGAGTGGAATTCCATGTGTAGCGGTGAAATGCGTAGATATATGGAGGAACACC  660
              ||||||||||||||||||||||||||||||||||||||||||||||||||||||||||||
Sbjct  80303  GAAGGGGAGAGTGGAATTCCATGTGTAGCGGTGAAATGCGTAGATATATGGAGGAACACC  80362

Query  661    GGTGGCGAAAGCGGCTCTCTGGTCTGTAACTGACGCTGAGGCTCGAAAGCGTGGGGAGCA  720
              ||||||||||||||||||||||||||||||||||||||||||||||||||||||||||||
Sbjct  80363  GGTGGCGAAAGCGGCTCTCTGGTCTGTAACTGACGCTGAGGCTCGAAAGCGTGGGGAGCA  80422

Query  721    AACAGGATTAGATACCCTGGTAGTCCACGCCGTAAACGATGAGTGCTAGGTGTTAGGCCC  780
              ||||||||||||||||||||||||||||||||||||||||||||||||||||||||||||
Sbjct  80423  AACAGGATTAGATACCCTGGTAGTCCACGCCGTAAACGATGAGTGCTAGGTGTTAGGCCC  80482

Query  781    TTTCCGGGGCTTAGTGCCGCAGCTAACGCATTAAGCACTC  820
              ||||||||||||||||||||||||||||||||||||||||
Sbjct  80483  TTTCCGGGGCTTAGTGCCGCAGCTAACGCATTAAGCACTC  80522
```

Range 2: 177045 to 177864

Score	Expect	Identities	Gaps	Strand	Frame
1515 bits(820)	0.0()	820/820(100%)	0/820(0%)	Plus/Plus	

Features:

```
Query  1       CGCTGATGTTTGGTGTTTACACTAGACTGATGAGTTGCGAACGGGTGAGTAACGCGTAGG  60
               ||||||||||||||||||||||||||||||||||||||||||||||||||||||||||||
Sbjct  177045  CGCTGATGTTTGGTGTTTACACTAGACTGATGAGTTGCGAACGGGTGAGTAACGCGTAGG  177104

Query  61      TAACCTGCCTCATAGCGGGGGATAACTATTGGAAACGATAGCTAATACCGCATAAGAGTA  120
               ||||||||||||||||||||||||||||||||||||||||||||||||||||||||||||
Sbjct  177105  TAACCTGCCTCATAGCGGGGGATAACTATTGGAAACGATAGCTAATACCGCATAAGAGTA  177164

Query  121     ATTAACACATGTTAGTTATTTAAAAGGAGCAATTGCTTCACTGTGAGATGGACCTGCGTT  180
               ||||||||||||||||||||||||||||||||||||||||||||||||||||||||||||
Sbjct  177165  ATTAACACATGTTAGTTATTTAAAAGGAGCAATTGCTTCACTGTGAGATGGACCTGCGTT  177224

Query  181     GTATTAGCTAGTTGGTGAGGTAAAGGCTCACCAAGGCGACGATACATAGCCGACCTGAGA  240
               ||||||||||||||||||||||||||||||||||||||||||||||||||||||||||||
Sbjct  177225  GTATTAGCTAGTTGGTGAGGTAAAGGCTCACCAAGGCGACGATACATAGCCGACCTGAGA  177284

Query  241     GGGTGATCGGCCACACTGGGACTGAGACACGGCCCAGACTCCTACGGGAGGCAGCAGTAG  300
               ||||||||||||||||||||||||||||||||||||||||||||||||||||||||||||
Sbjct  177285  GGGTGATCGGCCACACTGGGACTGAGACACGGCCCAGACTCCTACGGGAGGCAGCAGTAG  177344

Query  301     GGAATCTTCGGCAATGGACGGAAGTCTGACCGAGCAACGCCGCGTGAGTGAAGAAGGTTT  360
               ||||||||||||||||||||||||||||||||||||||||||||||||||||||||||||
Sbjct  177345  GGAATCTTCGGCAATGGACGGAAGTCTGACCGAGCAACGCCGCGTGAGTGAAGAAGGTTT  177404

Query  361     TCGGATCGTAAAGCTCTGTTGTTAGAGAAGAACGTTGGTAGGAGTGGAAAATCTACCAAG  420
               ||||||||||||||||||||||||||||||||||||||||||||||||||||||||||||
Sbjct  177405  TCGGATCGTAAAGCTCTGTTGTTAGAGAAGAACGTTGGTAGGAGTGGAAAATCTACCAAG  177464

Query  421     TGACGGTAACTAACCAGAAAGGGACGGCTAACTACGTGCCAGCAGCCGCGGTAATACGTA  480
               ||||||||||||||||||||||||||||||||||||||||||||||||||||||||||||
Sbjct  177465  TGACGGTAACTAACCAGAAAGGGACGGCTAACTACGTGCCAGCAGCCGCGGTAATACGTA  177524

Query  481     GGTCCCGAGCGTTGTCCGGATTTATTGGGCGTAAAGCGAGCGCAGGCGGTTCTTTAAGTC  540
               ||||||||||||||||||||||||||||||||||||||||||||||||||||||||||||
Sbjct  177525  GGTCCCGAGCGTTGTCCGGATTTATTGGGCGTAAAGCGAGCGCAGGCGGTTCTTTAAGTC  177584

Query  541     TGAAGTTAAAGGCAGTGGCTTAACCATTGTACGCTTTGGAAACTGGAGGACTTGAGTGCA  600
               ||||||||||||||||||||||||||||||||||||||||||||||||||||||||||||
Sbjct  177585  TGAAGTTAAAGGCAGTGGCTTAACCATTGTACGCTTTGGAAACTGGAGGACTTGAGTGCA  177644

Query  601     GAAGGGGAGAGTGGAATTCCATGTGTAGCGGTGAAATGCGTAGATATATGGAGGAACACC  660
               ||||||||||||||||||||||||||||||||||||||||||||||||||||||||||||
Sbjct  177645  GAAGGGGAGAGTGGAATTCCATGTGTAGCGGTGAAATGCGTAGATATATGGAGGAACACC  177704

Query  661     GGTGGCGAAAGCGGCTCTCTGGTCTGTAACTGACGCTGAGGCTCGAAAGCGTGGGGAGCA  720
               ||||||||||||||||||||||||||||||||||||||||||||||||||||||||||||
Sbjct  177705  GGTGGCGAAAGCGGCTCTCTGGTCTGTAACTGACGCTGAGGCTCGAAAGCGTGGGGAGCA  177764

Query  721     AACAGGATTAGATACCCTGGTAGTCCACGCCGTAAACGATGAGTGCTAGGTGTTAGGCCC  780
               ||||||||||||||||||||||||||||||||||||||||||||||||||||||||||||
Sbjct  177765  AACAGGATTAGATACCCTGGTAGTCCACGCCGTAAACGATGAGTGCTAGGTGTTAGGCCC  177824

Query  781     TTTCCGGGGCTTAGTGCCGCAGCTAACGCATTAAGCACTC  820
               ||||||||||||||||||||||||||||||||||||||||
Sbjct  177825  TTTCCGGGGCTTAGTGCCGCAGCTAACGCATTAAGCACTC  177864
```

Range 3: 246189 to 247008

Score	Expect	Identities	Gaps	Strand	Frame
1515 bits(820)	0.0()	820/820(100%)	0/820(0%)	Plus/Plus	

Features:

```
Query  1        CGCTGATGTTTGGTGTTTACACTAGACTGATGAGTTGCGAACGGGTGAGTAACGCGTAGG  60
                ||||||||||||||||||||||||||||||||||||||||||||||||||||||||||||
Sbjct  246189   CGCTGATGTTTGGTGTTTACACTAGACTGATGAGTTGCGAACGGGTGAGTAACGCGTAGG  246248
Query  61       TAACCTGCCTCATAGCGGGGGATAACTATTGGAAACGATAGCTAATACCGCATAAGAGTA  120
                ||||||||||||||||||||||||||||||||||||||||||||||||||||||||||||
Sbjct  246249   TAACCTGCCTCATAGCGGGGGATAACTATTGGAAACGATAGCTAATACCGCATAAGAGTA  246308
Query  121      ATTAACACATGTTAGTTATTTAAAAGGAGCAATTGCTTCACTGTGAGATGGACCTGCGTT  180
                ||||||||||||||||||||||||||||||||||||||||||||||||||||||||||||
Sbjct  246309   ATTAACACATGTTAGTTATTTAAAAGGAGCAATTGCTTCACTGTGAGATGGACCTGCGTT  246368
Query  181      GTATTAGCTAGTTGGTGAGGTAAAGGCTCACCAAGGCGACGATACATAGCCGACCTGAGA  240
                ||||||||||||||||||||||||||||||||||||||||||||||||||||||||||||
Sbjct  246369   GTATTAGCTAGTTGGTGAGGTAAAGGCTCACCAAGGCGACGATACATAGCCGACCTGAGA  246428
Query  241      GGGTGATCGGCCACACTGGGACTGAGACACGGCCCAGACTCCTACGGGAGGCAGCAGTAG  300
                ||||||||||||||||||||||||||||||||||||||||||||||||||||||||||||
Sbjct  246429   GGGTGATCGGCCACACTGGGACTGAGACACGGCCCAGACTCCTACGGGAGGCAGCAGTAG  246488
Query  301      GGAATCTTCGGCAATGGACGGAAGTCTGACCGAGCAACGCCGCGTGAGTGAAGAAGGTTT  360
                ||||||||||||||||||||||||||||||||||||||||||||||||||||||||||||
Sbjct  246489   GGAATCTTCGGCAATGGACGGAAGTCTGACCGAGCAACGCCGCGTGAGTGAAGAAGGTTT  246548
Query  361      TCGGATCGTAAAGCTCTGTTGTTAGAGAAGAACGTTGGTAGGAGTGGAAAATCTACCAAG  420
                ||||||||||||||||||||||||||||||||||||||||||||||||||||||||||||
Sbjct  246549   TCGGATCGTAAAGCTCTGTTGTTAGAGAAGAACGTTGGTAGGAGTGGAAAATCTACCAAG  246608
Query  421      TGACGGTAACTAACCAGAAAGGGACGGCTAACTACGTGCCAGCAGCCGCGGTAATACGTA  480
                ||||||||||||||||||||||||||||||||||||||||||||||||||||||||||||
Sbjct  246609   TGACGGTAACTAACCAGAAAGGGACGGCTAACTACGTGCCAGCAGCCGCGGTAATACGTA  246668
Query  481      GGTCCCGAGCGTTGTCCGGATTTATTGGGCGTAAAGCGAGCGCAGGCGGTTCTTTAAGTC  540
                ||||||||||||||||||||||||||||||||||||||||||||||||||||||||||||
Sbjct  246669   GGTCCCGAGCGTTGTCCGGATTTATTGGGCGTAAAGCGAGCGCAGGCGGTTCTTTAAGTC  246728
Query  541      TGAAGTTAAAGGCAGTGGCTTAACCATTGTACGCTTTGGAAACTGGAGGACTTGAGTGCA  600
                ||||||||||||||||||||||||||||||||||||||||||||||||||||||||||||
Sbjct  246729   TGAAGTTAAAGGCAGTGGCTTAACCATTGTACGCTTTGGAAACTGGAGGACTTGAGTGCA  246788
Query  601      GAAGGGGAGAGTGGAATTCCATGTGTAGCGGTGAAATGCGTAGATATATGGAGGAACACC  660
                ||||||||||||||||||||||||||||||||||||||||||||||||||||||||||||
Sbjct  246789   GAAGGGGAGAGTGGAATTCCATGTGTAGCGGTGAAATGCGTAGATATATGGAGGAACACC  246848
Query  661      GGTGGCGAAAGCGGCTCTCTGGTCTGTAACTGACGCTGAGGCTCGAAAGCGTGGGGAGCA  720
                ||||||||||||||||||||||||||||||||||||||||||||||||||||||||||||
Sbjct  246849   GGTGGCGAAAGCGGCTCTCTGGTCTGTAACTGACGCTGAGGCTCGAAAGCGTGGGGAGCA  246908
Query  721      AACAGGATTAGATACCCTGGTAGTCCACGCCGTAAACGATGAGTGCTAGGTGTTAGGCCC  780
                ||||||||||||||||||||||||||||||||||||||||||||||||||||||||||||
Sbjct  246909   AACAGGATTAGATACCCTGGTAGTCCACGCCGTAAACGATGAGTGCTAGGTGTTAGGCCC  246968
Query  781      TTTCCGGGGCTTAGTGCCGCAGCTAACGCATTAAGCACTC  820
                ||||||||||||||||||||||||||||||||||||||||
Sbjct  246969   TTTCCGGGGCTTAGTGCCGCAGCTAACGCATTAAGCACTC  247008
```

Range 4: 2066903 to 2067722

Score	Expect	Identities	Gaps	Strand	Frame
1515 bits(820)	0.0()	820/820(100%)	0/820(0%)	Plus/Plus	

Features:

```
Query  1         CGCTGATGTTTGGTGTTTACACTAGACTGATGAGTTGCGAACGGGTGAGTAACGCGTAGG  60
                 ||||||||||||||||||||||||||||||||||||||||||||||||||||||||||||
Sbjct  2066903   CGCTGATGTTTGGTGTTTACACTAGACTGATGAGTTGCGAACGGGTGAGTAACGCGTAGG  2066962
Query  61        TAACCTGCCTCATAGCGGGGGATAACTATTGGAAACGATAGCTAATACCGCATAAGAGTA  120
                 ||||||||||||||||||||||||||||||||||||||||||||||||||||||||||||
Sbjct  2066963   TAACCTGCCTCATAGCGGGGGATAACTATTGGAAACGATAGCTAATACCGCATAAGAGTA  2067022
Query  121       ATTAACACATGTTAGTTATTTAAAAGGAGCAATTGCTTCACTGTGAGATGGACCTGCGTT  180
                 ||||||||||||||||||||||||||||||||||||||||||||||||||||||||||||
Sbjct  2067023   ATTAACACATGTTAGTTATTTAAAAGGAGCAATTGCTTCACTGTGAGATGGACCTGCGTT  2067082
Query  181       GTATTAGCTAGTTGGTGAGGTAAAGGCTCACCAAGGCGACGATACATAGCCGACCTGAGA  240
                 ||||||||||||||||||||||||||||||||||||||||||||||||||||||||||||
Sbjct  2067083   GTATTAGCTAGTTGGTGAGGTAAAGGCTCACCAAGGCGACGATACATAGCCGACCTGAGA  2067142
Query  241       GGGTGATCGGCCACACTGGGACTGAGACACGGCCCAGACTCCTACGGGAGGCAGCAGTAG  300
                 ||||||||||||||||||||||||||||||||||||||||||||||||||||||||||||
Sbjct  2067143   GGGTGATCGGCCACACTGGGACTGAGACACGGCCCAGACTCCTACGGGAGGCAGCAGTAG  2067202
Query  301       GGAATCTTCGGCAATGGACGGAAGTCTGACCGAGCAACGCCGCGTGAGTGAAGAAGGTTT  360
                 ||||||||||||||||||||||||||||||||||||||||||||||||||||||||||||
Sbjct  2067203   GGAATCTTCGGCAATGGACGGAAGTCTGACCGAGCAACGCCGCGTGAGTGAAGAAGGTTT  2067262
Query  361       TCGGATCGTAAAGCTCTGTTGTTAGAGAAGAACGTTGGTAGGAGTGGAAAATCTACCAAG  420
                 ||||||||||||||||||||||||||||||||||||||||||||||||||||||||||||
Sbjct  2067263   TCGGATCGTAAAGCTCTGTTGTTAGAGAAGAACGTTGGTAGGAGTGGAAAATCTACCAAG  2067322
Query  421       TGACGGTAACTAACCAGAAAGGGACGGCTAACTACGTGCCAGCAGCCGCGGTAATACGTA  480
                 ||||||||||||||||||||||||||||||||||||||||||||||||||||||||||||
Sbjct  2067323   TGACGGTAACTAACCAGAAAGGGACGGCTAACTACGTGCCAGCAGCCGCGGTAATACGTA  2067382
Query  481       GGTCCCGAGCGTTGTCCGGATTTATTGGGCGTAAAGCGAGCGCAGGCGGTTCTTTAAGTC  540
                 ||||||||||||||||||||||||||||||||||||||||||||||||||||||||||||
Sbjct  2067383   GGTCCCGAGCGTTGTCCGGATTTATTGGGCGTAAAGCGAGCGCAGGCGGTTCTTTAAGTC  2067442
Query  541       TGAAGTTAAAGGCAGTGGCTTAACCATTGTACGCTTTGGAAACTGGAGGACTTGAGTGCA  600
                 ||||||||||||||||||||||||||||||||||||||||||||||||||||||||||||
Sbjct  2067443   TGAAGTTAAAGGCAGTGGCTTAACCATTGTACGCTTTGGAAACTGGAGGACTTGAGTGCA  2067502
Query  601       GAAGGGGAGAGTGGAATTCCATGTGTAGCGGTGAAATGCGTAGATATATGGAGGAACACC  660
                 ||||||||||||||||||||||||||||||||||||||||||||||||||||||||||||
Sbjct  2067503   GAAGGGGAGAGTGGAATTCCATGTGTAGCGGTGAAATGCGTAGATATATGGAGGAACACC  2067562
Query  661       GGTGGCGAAAGCGGCTCTCTGGTCTGTAACTGACGCTGAGGCTCGAAAGCGTGGGGAGCA  720
                 ||||||||||||||||||||||||||||||||||||||||||||||||||||||||||||
Sbjct  2067563   GGTGGCGAAAGCGGCTCTCTGGTCTGTAACTGACGCTGAGGCTCGAAAGCGTGGGGAGCA  2067622
Query  721       AACAGGATTAGATACCCTGGTAGTCCACGCCGTAAACGATGAGTGCTAGGTGTTAGGCCC  780
                 ||||||||||||||||||||||||||||||||||||||||||||||||||||||||||||
Sbjct  2067623   AACAGGATTAGATACCCTGGTAGTCCACGCCGTAAACGATGAGTGCTAGGTGTTAGGCCC  2067682
Query  781       TTTCCGGGGCTTAGTGCCGCAGCTAACGCATTAAGCACTC  820
                 ||||||||||||||||||||||||||||||||||||||||
Sbjct  2067683   TTTCCGGGGCTTAGTGCCGCAGCTAACGCATTAAGCACTC  2067722
```

Range 5: 1899344 to 1900163

Score	Expect	Identities	Gaps	Strand	Frame
1509 bits(817)	0.0()	819/820(99%)	0/820(0%)	Plus/Plus	

Features:

```
Query  1         CGCTGATGTTTGGTGTTTACACTAGACTGATGAGTTGCGAACGGGTGAGTAACGCGTAGG  60
                 ||||||||||||||||||||||||||||||||||||||||||||||||||||||||||||
Sbjct  1899344   CGCTGATGTTTGGTGTTTACACTAGACTGATGAGTTGCGAACGGGTGAGTAACGCGTAGG  1899403
Query  61        TAACCTGCCTCATAGCGGGGGATAACTATTGGAAACGATAGCTAATACCGCATAAGAGTA  120
                 ||||||||||||||||||||||||||||||||||||||||||||||||||||||||||| 
Sbjct  1899404   TAACCTGCCTCATAGCGGGGGATAACTATTGGAAACGATAGCTAATACCGCATAAGAGTG  1899463
Query  121       ATTAACACATGTTAGTTATTTAAAAGGAGCAATTGCTTCACTGTGAGATGGACCTGCGTT  180
                 ||||||||||||||||||||||||||||||||||||||||||||||||||||||||||||
Sbjct  1899464   ATTAACACATGTTAGTTATTTAAAAGGAGCAATTGCTTCACTGTGAGATGGACCTGCGTT  1899523
Query  181       GTATTAGCTAGTTGGTGAGGTAAAGGCTCACCAAGGCGACGATACATAGCCGACCTGAGA  240
                 ||||||||||||||||||||||||||||||||||||||||||||||||||||||||||||
Sbjct  1899524   GTATTAGCTAGTTGGTGAGGTAAAGGCTCACCAAGGCGACGATACATAGCCGACCTGAGA  1899583
Query  241       GGGTGATCGGCCACACTGGGACTGAGACACGGCCCAGACTCCTACGGGAGGCAGCAGTAG  300
                 ||||||||||||||||||||||||||||||||||||||||||||||||||||||||||||
Sbjct  1899584   GGGTGATCGGCCACACTGGGACTGAGACACGGCCCAGACTCCTACGGGAGGCAGCAGTAG  1899643
Query  301       GGAATCTTCGGCAATGGACGGAAGTCTGACCGAGCAACGCCGCGTGAGTGAAGAAGGTTT  360
```

```
                     ||||||||||||||||||||||||||||||||||||||||||||||||||||||||||||
Sbjct  1899644  GGAATCTTCGGCAATGGACGGAAGTCTGACCGAGCAACGCCGCGTGAGTGAAGAAGGTTT  1899703

Query  361      TCGGATCGTAAAGCTCTGTTGTTAGAGAAGAACGTTGGTAGGAGTGGAAAATCTACCAAG  420
                ||||||||||||||||||||||||||||||||||||||||||||||||||||||||||||
Sbjct  1899704  TCGGATCGTAAAGCTCTGTTGTTAGAGAAGAACGTTGGTAGGAGTGGAAAATCTACCAAG  1899763

Query  421      TGACGGTAACTAACCAGAAAGGGACGGCTAACTACGTGCCAGCAGCCGCGGTAATACGTA  480
                ||||||||||||||||||||||||||||||||||||||||||||||||||||||||||||
Sbjct  1899764  TGACGGTAACTAACCAGAAAGGGACGGCTAACTACGTGCCAGCAGCCGCGGTAATACGTA  1899823

Query  481      GGTCCCGAGCGTTGTCCGGATTTATTGGGCGTAAAGCGAGCGCAGGCGGTTCTTTAAGTC  540
                ||||||||||||||||||||||||||||||||||||||||||||||||||||||||||||
Sbjct  1899824  GGTCCCGAGCGTTGTCCGGATTTATTGGGCGTAAAGCGAGCGCAGGCGGTTCTTTAAGTC  1899883

Query  541      TGAAGTTAAAGGCAGTGGCTTAACCATTGTACGCTTTGGAAACTGGAGGACTTGAGTGCA  600
                ||||||||||||||||||||||||||||||||||||||||||||||||||||||||||||
Sbjct  1899884  TGAAGTTAAAGGCAGTGGCTTAACCATTGTACGCTTTGGAAACTGGAGGACTTGAGTGCA  1899943

Query  601      GAAGGGGAGAGTGGAATTCCATGTGTAGCGGTGAAATGCGTAGATATATGGAGGAACACC  660
                ||||||||||||||||||||||||||||||||||||||||||||||||||||||||||||
Sbjct  1899944  GAAGGGGAGAGTGGAATTCCATGTGTAGCGGTGAAATGCGTAGATATATGGAGGAACACC  1900003

Query  661      GGTGGCGAAAGCGGCTCTCTGGTCTGTAACTGACGCTGAGGCTCGAAAGCGTGGGGAGCA  720
                ||||||||||||||||||||||||||||||||||||||||||||||||||||||||||||
Sbjct  1900004  GGTGGCGAAAGCGGCTCTCTGGTCTGTAACTGACGCTGAGGCTCGAAAGCGTGGGGAGCA  1900063

Query  721      AACAGGATTAGATACCCTGGTAGTCCACGCCGTAAACGATGAGTGCTAGGTGTTAGGCCC  780
                ||||||||||||||||||||||||||||||||||||||||||||||||||||||||||||
Sbjct  1900064  AACAGGATTAGATACCCTGGTAGTCCACGCCGTAAACGATGAGTGCTAGGTGTTAGGCCC  1900123

Query  781      TTTCCGGGGCTTAGTGCCGCAGCTAACGCATTAAGCACTC  820
                ||||||||||||||||||||||||||||||||||||||||
Sbjct  1900124  TTTCCGGGGCTTAGTGCCGCAGCTAACGCATTAAGCACTC  1900163
```

Range 6: 1905176 to 1905995

Score	Expect	Identities	Gaps	Strand	Frame
1509 bits(817)	0.0()	819/820(99%)	0/820(0%)	Plus/Plus	

Features:

```
Query  1        CGCTGATGTTTGGTGTTTACACTAGACTGATGAGTTGCGAACGGGTGAGTAACGCGTAGG  60
                ||||||||||||||||||||||||||||||||||||||||||||||||||||||||||||
Sbjct  1905176  CGCTGATGTTTGGTGTTTACACTAGACTGATGAGTTGCGAACGGGTGAGTAACGCGTAGG  1905235

Query  61       TAACCTGCCTCATAGCGGGGGATAACTATTGGAAACGATAGCTAATACCGCATAAGAGTA  120
                ||||||||||||||||||||||||||||||||||||||||||||||||||||||||||| 
Sbjct  1905236  TAACCTGCCTCATAGCGGGGGATAACTATTGGAAACGATAGCTAATACCGCATAAGAGTG  1905295

Query  121      ATTAACACATGTTAGTTATTTAAAAGGAGCAATTGCTTCACTGTGAGATGGACCTGCGTT  180
                ||||||||||||||||||||||||||||||||||||||||||||||||||||||||||||
Sbjct  1905296  ATTAACACATGTTAGTTATTTAAAAGGAGCAATTGCTTCACTGTGAGATGGACCTGCGTT  1905355

Query  181      GTATTAGCTAGTTGGTGAGGTAAAGGCTCACCAAGGCGACGATACATAGCCGACCTGAGA  240
                ||||||||||||||||||||||||||||||||||||||||||||||||||||||||||||
Sbjct  1905356  GTATTAGCTAGTTGGTGAGGTAAAGGCTCACCAAGGCGACGATACATAGCCGACCTGAGA  1905415

Query  241      GGGTGATCGGCCACACTGGGACTGAGACACGGCCCAGACTCCTACGGGAGGCAGCAGTAG  300
                ||||||||||||||||||||||||||||||||||||||||||||||||||||||||||||
Sbjct  1905416  GGGTGATCGGCCACACTGGGACTGAGACACGGCCCAGACTCCTACGGGAGGCAGCAGTAG  1905475

Query  301      GGAATCTTCGGCAATGGACGGAAGTCTGACCGAGCAACGCCGCGTGAGTGAAGAAGGTTT  360
                ||||||||||||||||||||||||||||||||||||||||||||||||||||||||||||
Sbjct  1905476  GGAATCTTCGGCAATGGACGGAAGTCTGACCGAGCAACGCCGCGTGAGTGAAGAAGGTTT  1905535

Query  361      TCGGATCGTAAAGCTCTGTTGTTAGAGAAGAACGTTGGTAGGAGTGGAAAATCTACCAAG  420
                ||||||||||||||||||||||||||||||||||||||||||||||||||||||||||||
Sbjct  1905536  TCGGATCGTAAAGCTCTGTTGTTAGAGAAGAACGTTGGTAGGAGTGGAAAATCTACCAAG  1905595

Query  421      TGACGGTAACTAACCAGAAAGGGACGGCTAACTACGTGCCAGCAGCCGCGGTAATACGTA  480
                ||||||||||||||||||||||||||||||||||||||||||||||||||||||||||||
Sbjct  1905596  TGACGGTAACTAACCAGAAAGGGACGGCTAACTACGTGCCAGCAGCCGCGGTAATACGTA  1905655

Query  481      GGTCCCGAGCGTTGTCCGGATTTATTGGGCGTAAAGCGAGCGCAGGCGGTTCTTTAAGTC  540
                ||||||||||||||||||||||||||||||||||||||||||||||||||||||||||||
Sbjct  1905656  GGTCCCGAGCGTTGTCCGGATTTATTGGGCGTAAAGCGAGCGCAGGCGGTTCTTTAAGTC  1905715

Query  541      TGAAGTTAAAGGCAGTGGCTTAACCATTGTACGCTTTGGAAACTGGAGGACTTGAGTGCA  600
                ||||||||||||||||||||||||||||||||||||||||||||||||||||||||||||
Sbjct  1905716  TGAAGTTAAAGGCAGTGGCTTAACCATTGTACGCTTTGGAAACTGGAGGACTTGAGTGCA  1905775

Query  601      GAAGGGGAGAGTGGAATTCCATGTGTAGCGGTGAAATGCGTAGATATATGGAGGAACACC  660
                ||||||||||||||||||||||||||||||||||||||||||||||||||||||||||||
Sbjct  1905776  GAAGGGGAGAGTGGAATTCCATGTGTAGCGGTGAAATGCGTAGATATATGGAGGAACACC  1905835

Query  661      GGTGGCGAAAGCGGCTCTCTGGTCTGTAACTGACGCTGAGGCTCGAAAGCGTGGGGAGCA  720
                ||||||||||||||||||||||||||||||||||||||||||||||||||||||||||||
Sbjct  1905836  GGTGGCGAAAGCGGCTCTCTGGTCTGTAACTGACGCTGAGGCTCGAAAGCGTGGGGAGCA  1905895

Query  721      AACAGGATTAGATACCCTGGTAGTCCACGCCGTAAACGATGAGTGCTAGGTGTTAGGCCC  780
                ||||||||||||||||||||||||||||||||||||||||||||||||||||||||||||
Sbjct  1905896  AACAGGATTAGATACCCTGGTAGTCCACGCCGTAAACGATGAGTGCTAGGTGTTAGGCCC  1905955

Query  781      TTTCCGGGGCTTAGTGCCGCAGCTAACGCATTAAGCACTC  820
                ||||||||||||||||||||||||||||||||||||||||
Sbjct  1905956  TTTCCGGGGCTTAGTGCCGCAGCTAACGCATTAAGCACTC  1905995
```

Range 7: 1973832 to 1974651

Score	Expect	Identities	Gaps	Strand	Frame
1509 bits(817)	0.0()	819/820(99%)	0/820(0%)	Plus/Plus	

Features:

```
Query  1        CGCTGATGTTTGGTGTTTACACTAGACTGATGAGTTGCGAACGGGTGAGTAACGCGTAGG  60
                ||||||||||||||||||||||||||||||||||||||||||||||||||||||||||||
Sbjct  1973832  CGCTGATGTTTGGTGTTTACACTAGACTGATGAGTTGCGAACGGGTGAGTAACGCGTAGG  1973891

Query  61       TAACCTGCCTCATAGCGGGGGATAACTATTGGAAACGATAGCTAATACCGCATAAGAGTA  120
                ||||||||||||||||||||||||||||||||||||||||||||||||||||||||||||
Sbjct  1973892  TAACCTGCCTCATAGCGGGGGATAACTATTGGAAACGATAGCTAATACCGCATAAGAGTA  1973951

Query  121      ATTAACACATGTTAGTTATTTAAAAGGAGCAATTGCTTCACTGTGAGATGGACCTGCGTT  180
                ||||||||||||||||||||||||||||||||||||||||||||||||||||||||||||
Sbjct  1973952  ATTAACACATGTTAGTTATTTAAAAGGAGCAATTGCTTCACTGTGAGATGGACCTGCGTT  1974011

Query  181      GTATTAGCTAGTTGGTGAGGTAAAGGCTCACCAAGGCGACGATACATAGCCGACCTGAGA  240
                ||||||||||||||||||||||||||||||||||||||||||||||||||||||||||||
Sbjct  1974012  GTATTAGCTAGTTGGTGAGGTAAAGGCTCACCAAGGCGACGATACATAGCCGACCTGAGA  1974071

Query  241      GGGTGATCGGCCACACTGGGACTGAGACACGGCCCAGACTCCTACGGGAGGCAGCAGTAG  300
                ||||||||||||||||||||||||||||||||||||||||||||||||||||||||||||
Sbjct  1974072  GGGTGATCGGCCACACTGGGACTGAGACACGGCCCAGACTCCTACGGGAGGCAGCAGTAG  1974131

Query  301      GGAATCTTCGGCAATGGACGGAAGTCTGACCGAGCAACGCCGCGTGAGTGAAGAAGGTTT  360
                ||||||||||||||||||||||||||||||||||||||||||||||||||||||||||||
Sbjct  1974132  GGAATCTTCGGCAATGGACGGAAGTCTGACCGAGCAACGCCGCGTGAGTGAAGAAGGTTT  1974191

Query  361      TCGGATCGTAAAGCTCTGTTGTTAGAGAAGAACGTTGGTAGGAGTGGAAAATCTACCAAG  420
                ||||||||||||||||||||||||||||||||||||||||||||||||||||||||||||
Sbjct  1974192  TCGGATCGTAAAGCTCTGTTGTTAGAGAAGAACGTTGGTAGGAGTGGAAAATCTACCAAG  1974251

Query  421      TGACGGTAACTAACCAGAAAGGGACGGCTAACTACGTGCCAGCAGCCGCGGTAATACGTA  480
                ||||||||||||||||||||||||||||||||||||||||||||||||||||||||||||
Sbjct  1974252  TGACGGTAACTAACCAGAAAGGGACGGCTAACTACGTGCCAGCAGCCGCGGTAATACGTA  1974311

Query  481      GGTCCCGAGCGTTGTCCGGATTTATTGGGCGTAAAGCGAGCGCAGGCGGTTCTTTAAGTC  540
                ||||||||||||||||||||||||||||||||||||||||||||||||||||||||||||
Sbjct  1974312  GGTCCCGAGCGTTGTCCGGATTTATTGGGCGTAAAGCGAGCGCAGGCGGTTCTTTAAGTC  1974371

Query  541      TGAAGTTAAAGGCAGTGGCTTAACCATTGTACGCTTTGGAAACTGGAGGACTTGAGTGCA  600
                ||||||||||||||||||||||||||||||||||||||||||||||||||||||||||||
Sbjct  1974372  TGAAGTTAAAGGCAGTGGCTTAACCATTGTACGCTTTGGAAACTGGAGGACTTGAGTGCA  1974431

Query  601      GAAGGGGAGAGTGGAATTCCATGTGTAGCGGTGAAATGCGTAGATATATGGAGGAACACC  660
                ||||||||||||||||||||||||||||||||||||||||||||||||||||||||||||
Sbjct  1974432  GAAGGGGAGAGTGGAATTCCATGTGTAGCGGTGAAATGCGTAGATATATGGAGGAACACC  1974491
```

```
Query  661      GGTGGCGAAAGCGGCTCTCTGGTCTGTAACTGACGCTGAGGCTCGAAAGCGTGGGGAGCA  720
                ||||||||||||||||||||||||||||||||||||||||||||||||||||||||||||
Sbjct  1974492  GGTGGCGAAAGCGGCTCTCTGGTCTGTAACTGACGCTGAGGCTCGAAAGCGTGGGGAGCA  1974551

Query  721      AACAGGATTAGATACCCTGGTAGTCCACGCCGTAAACGATGAGTGCTAGGTGTTAGGCCC  780
                ||||||||||||||||||||||||||||||||||||||||||||||||||||||||| ||
Sbjct  1974552  AACAGGATTAGATACCCTGGTAGTCCACGCCGTAAACGATGAGTGCTAGGTGTTAGGTCC  1974611

Query  781      TTTCCGGGGCTTAGTGCCGCAGCTAACGCATTAAGCACTC  820
                ||||||||||||||||||||||||||||||||||||||||
Sbjct  1974612  TTTCCGGGGCTTAGTGCCGCAGCTAACGCATTAAGCACTC  1974651
```

Streptococcus agalactiae strain B509 chromosome, complete genome

Sequence ID: **CP021769.1** Length: 2060637 Number of Matches: 7

Range 1: 56182 to 57001

Score	Expect	Identities	Gaps	Strand	Frame
1515 bits(820)	0.0()	820/820(100%)	0/820(0%)	Plus/Minus	

Features:

```
Query  1      CGCTGATGTTTGGTGTTTACACTAGACTGATGAGTTGCGAACGGGTGAGTAACGCGTAGG  60
              ||||||||||||||||||||||||||||||||||||||||||||||||||||||||||||
Sbjct  57001  CGCTGATGTTTGGTGTTTACACTAGACTGATGAGTTGCGAACGGGTGAGTAACGCGTAGG  56942

Query  61     TAACCTGCCTCATAGCGGGGGATAACTATTGGAAACGATAGCTAATACCGCATAAGAGTA  120
              ||||||||||||||||||||||||||||||||||||||||||||||||||||||||||||
Sbjct  56941  TAACCTGCCTCATAGCGGGGGATAACTATTGGAAACGATAGCTAATACCGCATAAGAGTA  56882

Query  121    ATTAACACATGTTAGTTATTTAAAAGGAGCAATTGCTTCACTGTGAGATGGACCTGCGTT  180
              ||||||||||||||||||||||||||||||||||||||||||||||||||||||||||||
Sbjct  56881  ATTAACACATGTTAGTTATTTAAAAGGAGCAATTGCTTCACTGTGAGATGGACCTGCGTT  56822

Query  181    GTATTAGCTAGTTGGTGAGGTAAAGGCTCACCAAGGCGACGATACATAGCCGACCTGAGA  240
              ||||||||||||||||||||||||||||||||||||||||||||||||||||||||||||
Sbjct  56821  GTATTAGCTAGTTGGTGAGGTAAAGGCTCACCAAGGCGACGATACATAGCCGACCTGAGA  56762

Query  241    GGGTGATCGGCCACACTGGGACTGAGACACGGCCCAGACTCCTACGGGAGGCAGCAGTAG  300
              ||||||||||||||||||||||||||||||||||||||||||||||||||||||||||||
Sbjct  56761  GGGTGATCGGCCACACTGGGACTGAGACACGGCCCAGACTCCTACGGGAGGCAGCAGTAG  56702

Query  301    GGAATCTTCGGCAATGGACGGAAGTCTGACCGAGCAACGCCGCGTGAGTGAAGAAGGTTT  360
              ||||||||||||||||||||||||||||||||||||||||||||||||||||||||||||
Sbjct  56701  GGAATCTTCGGCAATGGACGGAAGTCTGACCGAGCAACGCCGCGTGAGTGAAGAAGGTTT  56642

Query  361    TCGGATCGTAAAGCTCTGTTGTTAGAGAAGAACGTTGGTAGGAGTGGAAAATCTACCAAG  420
              ||||||||||||||||||||||||||||||||||||||||||||||||||||||||||||
Sbjct  56641  TCGGATCGTAAAGCTCTGTTGTTAGAGAAGAACGTTGGTAGGAGTGGAAAATCTACCAAG  56582

Query  421    TGACGGTAACTAACCAGAAAGGGACGGCTAACTACGTGCCAGCAGCCGCGGTAATACGTA  480
              ||||||||||||||||||||||||||||||||||||||||||||||||||||||||||||
Sbjct  56581  TGACGGTAACTAACCAGAAAGGGACGGCTAACTACGTGCCAGCAGCCGCGGTAATACGTA  56522

Query  481    GGTCCCGAGCGTTGTCCGGATTTATTGGGCGTAAAGCGAGCGCAGGCGGTTCTTTAAGTC  540
              ||||||||||||||||||||||||||||||||||||||||||||||||||||||||||||
Sbjct  56521  GGTCCCGAGCGTTGTCCGGATTTATTGGGCGTAAAGCGAGCGCAGGCGGTTCTTTAAGTC  56462

Query  541    TGAAGTTAAAGGCAGTGGCTTAACCATTGTACGCTTTGGAAACTGGAGGACTTGAGTGCA  600
              ||||||||||||||||||||||||||||||||||||||||||||||||||||||||||||
Sbjct  56461  TGAAGTTAAAGGCAGTGGCTTAACCATTGTACGCTTTGGAAACTGGAGGACTTGAGTGCA  56402

Query  601    GAAGGGGAGAGTGGAATTCCATGTGTAGCGGTGAAATGCGTAGATATATGGAGGAACACC  660
              ||||||||||||||||||||||||||||||||||||||||||||||||||||||||||||
Sbjct  56401  GAAGGGGAGAGTGGAATTCCATGTGTAGCGGTGAAATGCGTAGATATATGGAGGAACACC  56342

Query  661    GGTGGCGAAAGCGGCTCTCTGGTCTGTAACTGACGCTGAGGCTCGAAAGCGTGGGGAGCA  720
              ||||||||||||||||||||||||||||||||||||||||||||||||||||||||||||
Sbjct  56341  GGTGGCGAAAGCGGCTCTCTGGTCTGTAACTGACGCTGAGGCTCGAAAGCGTGGGGAGCA  56282

Query  721    AACAGGATTAGATACCCTGGTAGTCCACGCCGTAAACGATGAGTGCTAGGTGTTAGGCCC  780
              ||||||||||||||||||||||||||||||||||||||||||||||||||||||||||||
Sbjct  56281  AACAGGATTAGATACCCTGGTAGTCCACGCCGTAAACGATGAGTGCTAGGTGTTAGGCCC  56222

Query  781    TTTCCGGGGCTTAGTGCCGCAGCTAACGCATTAAGCACTC  820
              ||||||||||||||||||||||||||||||||||||||||
Sbjct  56221  TTTCCGGGGCTTAGTGCCGCAGCTAACGCATTAAGCACTC  56182
```

Range 2: 125325 to 126144

Score	Expect	Identities	Gaps	Strand	Frame
1515 bits(820)	0.0()	820/820(100%)	0/820(0%)	Plus/Minus	

Features:

```
Query  1       CGCTGATGTTTGGTGTTTACACTAGACTGATGAGTTGCGAACGGGTGAGTAACGCGTAGG  60
               ||||||||||||||||||||||||||||||||||||||||||||||||||||||||||||
Sbjct  126144  CGCTGATGTTTGGTGTTTACACTAGACTGATGAGTTGCGAACGGGTGAGTAACGCGTAGG  126085

Query  61      TAACCTGCCTCATAGCGGGGGATAACTATTGGAAACGATAGCTAATACCGCATAAGAGTA  120
               ||||||||||||||||||||||||||||||||||||||||||||||||||||||||||||
Sbjct  126084  TAACCTGCCTCATAGCGGGGGATAACTATTGGAAACGATAGCTAATACCGCATAAGAGTA  126025

Query  121     ATTAACACATGTTAGTTATTTAAAAGGAGCAATTGCTTCACTGTGAGATGGACCTGCGTT  180
               ||||||||||||||||||||||||||||||||||||||||||||||||||||||||||||
Sbjct  126024  ATTAACACATGTTAGTTATTTAAAAGGAGCAATTGCTTCACTGTGAGATGGACCTGCGTT  125965

Query  181     GTATTAGCTAGTTGGTGAGGTAAAGGCTCACCAAGGCGACGATACATAGCCGACCTGAGA  240
               ||||||||||||||||||||||||||||||||||||||||||||||||||||||||||||
Sbjct  125964  GTATTAGCTAGTTGGTGAGGTAAAGGCTCACCAAGGCGACGATACATAGCCGACCTGAGA  125905

Query  241     GGGTGATCGGCCACACTGGGACTGAGACACGGCCCAGACTCCTACGGGAGGCAGCAGTAG  300
               ||||||||||||||||||||||||||||||||||||||||||||||||||||||||||||
Sbjct  125904  GGGTGATCGGCCACACTGGGACTGAGACACGGCCCAGACTCCTACGGGAGGCAGCAGTAG  125845

Query  301     GGAATCTTCGGCAATGGACGGAAGTCTGACCGAGCAACGCCGCGTGAGTGAAGAAGGTTT  360
               ||||||||||||||||||||||||||||||||||||||||||||||||||||||||||||
Sbjct  125844  GGAATCTTCGGCAATGGACGGAAGTCTGACCGAGCAACGCCGCGTGAGTGAAGAAGGTTT  125785

Query  361     TCGGATCGTAAAGCTCTGTTGTTAGAGAAGAACGTTGGTAGGAGTGGAAAATCTACCAAG  420
               ||||||||||||||||||||||||||||||||||||||||||||||||||||||||||||
Sbjct  125784  TCGGATCGTAAAGCTCTGTTGTTAGAGAAGAACGTTGGTAGGAGTGGAAAATCTACCAAG  125725

Query  421     TGACGGTAACTAACCAGAAAGGGACGGCTAACTACGTGCCAGCAGCCGCGGTAATACGTA  480
               ||||||||||||||||||||||||||||||||||||||||||||||||||||||||||||
Sbjct  125724  TGACGGTAACTAACCAGAAAGGGACGGCTAACTACGTGCCAGCAGCCGCGGTAATACGTA  125665

Query  481     GGTCCCGAGCGTTGTCCGGATTTATTGGGCGTAAAGCGAGCGCAGGCGGTTCTTTAAGTC  540
               ||||||||||||||||||||||||||||||||||||||||||||||||||||||||||||
Sbjct  125664  GGTCCCGAGCGTTGTCCGGATTTATTGGGCGTAAAGCGAGCGCAGGCGGTTCTTTAAGTC  125605

Query  541     TGAAGTTAAAGGCAGTGGCTTAACCATTGTACGCTTTGGAAACTGGAGGACTTGAGTGCA  600
               ||||||||||||||||||||||||||||||||||||||||||||||||||||||||||||
Sbjct  125604  TGAAGTTAAAGGCAGTGGCTTAACCATTGTACGCTTTGGAAACTGGAGGACTTGAGTGCA  125545

Query  601     GAAGGGGAGAGTGGAATTCCATGTGTAGCGGTGAAATGCGTAGATATATGGAGGAACACC  660
               ||||||||||||||||||||||||||||||||||||||||||||||||||||||||||||
Sbjct  125544  GAAGGGGAGAGTGGAATTCCATGTGTAGCGGTGAAATGCGTAGATATATGGAGGAACACC  125485

Query  661     GGTGGCGAAAGCGGCTCTCTGGTCTGTAACTGACGCTGAGGCTCGAAAGCGTGGGGAGCA  720
               ||||||||||||||||||||||||||||||||||||||||||||||||||||||||||||
Sbjct  125484  GGTGGCGAAAGCGGCTCTCTGGTCTGTAACTGACGCTGAGGCTCGAAAGCGTGGGGAGCA  125425

Query  721     AACAGGATTAGATACCCTGGTAGTCCACGCCGTAAACGATGAGTGCTAGGTGTTAGGCCC  780
               ||||||||||||||||||||||||||||||||||||||||||||||||||||||||||||
Sbjct  125424  AACAGGATTAGATACCCTGGTAGTCCACGCCGTAAACGATGAGTGCTAGGTGTTAGGCCC  125365

Query  781     TTTCCGGGGCTTAGTGCCGCAGCTAACGCATTAAGCACTC  820
               ||||||||||||||||||||||||||||||||||||||||
Sbjct  125364  TTTCCGGGGCTTAGTGCCGCAGCTAACGCATTAAGCACTC  125325
```

Range 3: 222666 to 223485

Score	Expect	Identities	Gaps	Strand	Frame

1515 bits(820)	0.0()	820/820(100%)	0/820(0%)	Plus/Minus	

Features:

```
Query  1       CGCTGATGTTTGGTGTTTACACTAGACTGATGAGTTGCGAACGGGTGAGTAACGCGTAGG  60
               ||||||||||||||||||||||||||||||||||||||||||||||||||||||||||||
Sbjct  223485  CGCTGATGTTTGGTGTTTACACTAGACTGATGAGTTGCGAACGGGTGAGTAACGCGTAGG  223426

Query  61      TAACCTGCCTCATAGCGGGGGATAACTATTGGAAACGATAGCTAATACCGCATAAGAGTA  120
               ||||||||||||||||||||||||||||||||||||||||||||||||||||||||||||
Sbjct  223425  TAACCTGCCTCATAGCGGGGGATAACTATTGGAAACGATAGCTAATACCGCATAAGAGTA  223366

Query  121     ATTAACACATGTTAGTTATTTAAAAGGAGCAATTGCTTCACTGTGAGATGGACCTGCGTT  180
               ||||||||||||||||||||||||||||||||||||||||||||||||||||||||||||
Sbjct  223365  ATTAACACATGTTAGTTATTTAAAAGGAGCAATTGCTTCACTGTGAGATGGACCTGCGTT  223306

Query  181     GTATTAGCTAGTTGGTGAGGTAAAGGCTCACCAAGGCGACGATACATAGCCGACCTGAGA  240
               ||||||||||||||||||||||||||||||||||||||||||||||||||||||||||||
Sbjct  223305  GTATTAGCTAGTTGGTGAGGTAAAGGCTCACCAAGGCGACGATACATAGCCGACCTGAGA  223246

Query  241     GGGTGATCGGCCACACTGGGACTGAGACACGGCCCAGACTCCTACGGGAGGCAGCAGTAG  300
               ||||||||||||||||||||||||||||||||||||||||||||||||||||||||||||
Sbjct  223245  GGGTGATCGGCCACACTGGGACTGAGACACGGCCCAGACTCCTACGGGAGGCAGCAGTAG  223186

Query  301     GGAATCTTCGGCAATGGACGGAAGTCTGACCGAGCAACGCCGCGTGAGTGAAGAAGGTTT  360
               ||||||||||||||||||||||||||||||||||||||||||||||||||||||||||||
Sbjct  223185  GGAATCTTCGGCAATGGACGGAAGTCTGACCGAGCAACGCCGCGTGAGTGAAGAAGGTTT  223126

Query  361     TCGGATCGTAAAGCTCTGTTGTTAGAGAAGAACGTTGGTAGGAGTGGAAAATCTACCAAG  420
               ||||||||||||||||||||||||||||||||||||||||||||||||||||||||||||
Sbjct  223125  TCGGATCGTAAAGCTCTGTTGTTAGAGAAGAACGTTGGTAGGAGTGGAAAATCTACCAAG  223066

Query  421     TGACGGTAACTAACCAGAAAGGGACGGCTAACTACGTGCCAGCAGCCGCGGTAATACGTA  480
               ||||||||||||||||||||||||||||||||||||||||||||||||||||||||||||
Sbjct  223065  TGACGGTAACTAACCAGAAAGGGACGGCTAACTACGTGCCAGCAGCCGCGGTAATACGTA  223006

Query  481     GGTCCCGAGCGTTGTCCGGATTTATTGGGCGTAAAGCGAGCGCAGGCGGTTCTTTAAGTC  540
               ||||||||||||||||||||||||||||||||||||||||||||||||||||||||||||
Sbjct  223005  GGTCCCGAGCGTTGTCCGGATTTATTGGGCGTAAAGCGAGCGCAGGCGGTTCTTTAAGTC  222946

Query  541     TGAAGTTAAAGGCAGTGGCTTAACCATTGTACGCTTTGGAAACTGGAGGACTTGAGTGCA  600
               ||||||||||||||||||||||||||||||||||||||||||||||||||||||||||||
Sbjct  222945  TGAAGTTAAAGGCAGTGGCTTAACCATTGTACGCTTTGGAAACTGGAGGACTTGAGTGCA  222886

Query  601     GAAGGGGAGAGTGGAATTCCATGTGTAGCGGTGAAATGCGTAGATATATGGAGGAACACC  660
               ||||||||||||||||||||||||||||||||||||||||||||||||||||||||||||
Sbjct  222885  GAAGGGGAGAGTGGAATTCCATGTGTAGCGGTGAAATGCGTAGATATATGGAGGAACACC  222826

Query  661     GGTGGCGAAAGCGGCTCTCTGGTCTGTAACTGACGCTGAGGCTCGAAAGCGTGGGGAGCA  720
               ||||||||||||||||||||||||||||||||||||||||||||||||||||||||||||
Sbjct  222825  GGTGGCGAAAGCGGCTCTCTGGTCTGTAACTGACGCTGAGGCTCGAAAGCGTGGGGAGCA  222766

Query  721     AACAGGATTAGATACCCTGGTAGTCCACGCCGTAAACGATGAGTGCTAGGTGTTAGGCCC  780
               ||||||||||||||||||||||||||||||||||||||||||||||||||||||||||||
Sbjct  222765  AACAGGATTAGATACCCTGGTAGTCCACGCCGTAAACGATGAGTGCTAGGTGTTAGGCCC  222706

Query  781     TTTCCGGGGCTTAGTGCCGCAGCTAACGCATTAAGCACTC  820
               ||||||||||||||||||||||||||||||||||||||||
Sbjct  222705  TTTCCGGGGCTTAGTGCCGCAGCTAACGCATTAAGCACTC  222666
```

Range 4: 391508 to 392327

Score	Expect	Identities	Gaps	Strand	Frame
1515 bits(820)	0.0()	820/820(100%)	0/820(0%)	Plus/Minus	

Features:

```
Query  1       CGCTGATGTTTGGTGTTTACACTAGACTGATGAGTTGCGAACGGGTGAGTAACGCGTAGG  60
               ||||||||||||||||||||||||||||||||||||||||||||||||||||||||||||
Sbjct  392327  CGCTGATGTTTGGTGTTTACACTAGACTGATGAGTTGCGAACGGGTGAGTAACGCGTAGG  392268

Query  61      TAACCTGCCTCATAGCGGGGGATAACTATTGGAAACGATAGCTAATACCGCATAAGAGTA  120
               ||||||||||||||||||||||||||||||||||||||||||||||||||||||||||||
Sbjct  392267  TAACCTGCCTCATAGCGGGGGATAACTATTGGAAACGATAGCTAATACCGCATAAGAGTA  392208

Query  121     ATTAACACATGTTAGTTATTTAAAAGGAGCAATTGCTTCACTGTGAGATGGACCTGCGTT  180
               ||||||||||||||||||||||||||||||||||||||||||||||||||||||||||||
Sbjct  392207  ATTAACACATGTTAGTTATTTAAAAGGAGCAATTGCTTCACTGTGAGATGGACCTGCGTT  392148

Query  181     GTATTAGCTAGTTGGTGAGGTAAAGGCTCACCAAGGCGACGATACATAGCCGACCTGAGA  240
               ||||||||||||||||||||||||||||||||||||||||||||||||||||||||||||
Sbjct  392147  GTATTAGCTAGTTGGTGAGGTAAAGGCTCACCAAGGCGACGATACATAGCCGACCTGAGA  392088

Query  241     GGGTGATCGGCCACACTGGGACTGAGACACGGCCCAGACTCCTACGGGAGGCAGCAGTAG  300
               ||||||||||||||||||||||||||||||||||||||||||||||||||||||||||||
Sbjct  392087  GGGTGATCGGCCACACTGGGACTGAGACACGGCCCAGACTCCTACGGGAGGCAGCAGTAG  392028

Query  301     GGAATCTTCGGCAATGGACGGAAGTCTGACCGAGCAACGCCGCGTGAGTGAAGAAGGTTT  360
               ||||||||||||||||||||||||||||||||||||||||||||||||||||||||||||
Sbjct  392027  GGAATCTTCGGCAATGGACGGAAGTCTGACCGAGCAACGCCGCGTGAGTGAAGAAGGTTT  391968

Query  361     TCGGATCGTAAAGCTCTGTTGTTAGAGAAGAACGTTGGTAGGAGTGGAAAATCTACCAAG  420
               ||||||||||||||||||||||||||||||||||||||||||||||||||||||||||||
Sbjct  391967  TCGGATCGTAAAGCTCTGTTGTTAGAGAAGAACGTTGGTAGGAGTGGAAAATCTACCAAG  391908

Query  421     TGACGGTAACTAACCAGAAAGGGACGGCTAACTACGTGCCAGCAGCCGCGGTAATACGTA  480
               ||||||||||||||||||||||||||||||||||||||||||||||||||||||||||||
Sbjct  391907  TGACGGTAACTAACCAGAAAGGGACGGCTAACTACGTGCCAGCAGCCGCGGTAATACGTA  391848

Query  481     GGTCCCGAGCGTTGTCCGGATTTATTGGGCGTAAAGCGAGCGCAGGCGGTTCTTTAAGTC  540
               ||||||||||||||||||||||||||||||||||||||||||||||||||||||||||||
Sbjct  391847  GGTCCCGAGCGTTGTCCGGATTTATTGGGCGTAAAGCGAGCGCAGGCGGTTCTTTAAGTC  391788

Query  541     TGAAGTTAAAGGCAGTGGCTTAACCATTGTACGCTTTGGAAACTGGAGGACTTGAGTGCA  600
               ||||||||||||||||||||||||||||||||||||||||||||||||||||||||||||
Sbjct  391787  TGAAGTTAAAGGCAGTGGCTTAACCATTGTACGCTTTGGAAACTGGAGGACTTGAGTGCA  391728

Query  601     GAAGGGGAGAGTGGAATTCCATGTGTAGCGGTGAAATGCGTAGATATATGGAGGAACACC  660
               ||||||||||||||||||||||||||||||||||||||||||||||||||||||||||||
Sbjct  391727  GAAGGGGAGAGTGGAATTCCATGTGTAGCGGTGAAATGCGTAGATATATGGAGGAACACC  391668

Query  661     GGTGGCGAAAGCGGCTCTCTGGTCTGTAACTGACGCTGAGGCTCGAAAGCGTGGGGAGCA  720
               ||||||||||||||||||||||||||||||||||||||||||||||||||||||||||||
Sbjct  391667  GGTGGCGAAAGCGGCTCTCTGGTCTGTAACTGACGCTGAGGCTCGAAAGCGTGGGGAGCA  391608

Query  721     AACAGGATTAGATACCCTGGTAGTCCACGCCGTAAACGATGAGTGCTAGGTGTTAGGCCC  780
               ||||||||||||||||||||||||||||||||||||||||||||||||||||||||||||
Sbjct  391607  AACAGGATTAGATACCCTGGTAGTCCACGCCGTAAACGATGAGTGCTAGGTGTTAGGCCC  391548

Query  781     TTTCCGGGGCTTAGTGCCGCAGCTAACGCATTAAGCACTC  820
               ||||||||||||||||||||||||||||||||||||||||
Sbjct  391547  TTTCCGGGGCTTAGTGCCGCAGCTAACGCATTAAGCACTC  391508
```

Range 5: 466029 to 466848

Score	Expect	Identities	Gaps	Strand	Frame
1515 bits(820)	0.0()	820/820(100%)	0/820(0%)	Plus/Minus	

Features:

```
Query  1       CGCTGATGTTTGGTGTTTACACTAGACTGATGAGTTGCGAACGGGTGAGTAACGCGTAGG  60
               ||||||||||||||||||||||||||||||||||||||||||||||||||||||||||||
Sbjct  466848  CGCTGATGTTTGGTGTTTACACTAGACTGATGAGTTGCGAACGGGTGAGTAACGCGTAGG  466789

Query  61      TAACCTGCCTCATAGCGGGGGATAACTATTGGAAACGATAGCTAATACCGCATAAGAGTA  120
               ||||||||||||||||||||||||||||||||||||||||||||||||||||||||||||
Sbjct  466788  TAACCTGCCTCATAGCGGGGGATAACTATTGGAAACGATAGCTAATACCGCATAAGAGTA  466729

Query  121     ATTAACACATGTTAGTTATTTAAAAGGAGCAATTGCTTCACTGTGAGATGGACCTGCGTT  180
               ||||||||||||||||||||||||||||||||||||||||||||||||||||||||||||
Sbjct  466728  ATTAACACATGTTAGTTATTTAAAAGGAGCAATTGCTTCACTGTGAGATGGACCTGCGTT  466669

Query  181     GTATTAGCTAGTTGGTGAGGTAAAGGCTCACCAAGGCGACGATACATAGCCGACCTGAGA  240
               ||||||||||||||||||||||||||||||||||||||||||||||||||||||||||||
Sbjct  466668  GTATTAGCTAGTTGGTGAGGTAAAGGCTCACCAAGGCGACGATACATAGCCGACCTGAGA  466609

Query  241     GGGTGATCGGCCACACTGGGACTGAGACACGGCCCAGACTCCTACGGGAGGCAGCAGTAG  300
               ||||||||||||||||||||||||||||||||||||||||||||||||||||||||||||
```

```
Sbjct  466608  GGGTGATCGGCCACACTGGGACTGAGACACGGCCCAGACTCCTACGGGAGGCAGCAGTAG  466549
Query  301     GGAATCTTCGGCAATGGACGGAAGTCTGACCGAGCAACGCCGCGTGAGTGAAGAAGGTTT  360
               ||||||||||||||||||||||||||||||||||||||||||||||||||||||||||||
Sbjct  466548  GGAATCTTCGGCAATGGACGGAAGTCTGACCGAGCAACGCCGCGTGAGTGAAGAAGGTTT  466489
Query  361     TCGGATCGTAAAGCTCTGTTGTTAGAGAAGAACGTTGGTAGGAGTGGAAAATCTACCAAG  420
               ||||||||||||||||||||||||||||||||||||||||||||||||||||||||||||
Sbjct  466488  TCGGATCGTAAAGCTCTGTTGTTAGAGAAGAACGTTGGTAGGAGTGGAAAATCTACCAAG  466429
Query  421     TGACGGTAACTAACCAGAAAGGGACGGCTAACTACGTGCCAGCAGCCGCGGTAATACGTA  480
               ||||||||||||||||||||||||||||||||||||||||||||||||||||||||||||
Sbjct  466428  TGACGGTAACTAACCAGAAAGGGACGGCTAACTACGTGCCAGCAGCCGCGGTAATACGTA  466369
Query  481     GGTCCCGAGCGTTGTCCGGATTTATTGGGCGTAAAGCGAGCGCAGGCGGTTCTTTAAGTC  540
               ||||||||||||||||||||||||||||||||||||||||||||||||||||||||||||
Sbjct  466368  GGTCCCGAGCGTTGTCCGGATTTATTGGGCGTAAAGCGAGCGCAGGCGGTTCTTTAAGTC  466309
Query  541     TGAAGTTAAAGGCAGTGGCTTAACCATTGTACGCTTTGGAAACTGGAGGACTTGAGTGCA  600
               ||||||||||||||||||||||||||||||||||||||||||||||||||||||||||||
Sbjct  466308  TGAAGTTAAAGGCAGTGGCTTAACCATTGTACGCTTTGGAAACTGGAGGACTTGAGTGCA  466249
Query  601     GAAGGGGAGAGTGGAATTCCATGTGTAGCGGTGAAATGCGTAGATATATGGAGGAACACC  660
               ||||||||||||||||||||||||||||||||||||||||||||||||||||||||||||
Sbjct  466248  GAAGGGGAGAGTGGAATTCCATGTGTAGCGGTGAAATGCGTAGATATATGGAGGAACACC  466189
Query  661     GGTGGCGAAAGCGGCTCTCTGGTCTGTAACTGACGCTGAGGCTCGAAAGCGTGGGGAGCA  720
               ||||||||||||||||||||||||||||||||||||||||||||||||||||||||||||
Sbjct  466188  GGTGGCGAAAGCGGCTCTCTGGTCTGTAACTGACGCTGAGGCTCGAAAGCGTGGGGAGCA  466129
Query  721     AACAGGATTAGATACCCTGGTAGTCCACGCCGTAAACGATGAGTGCTAGGTGTTAGGCCC  780
               ||||||||||||||||||||||||||||||||||||||||||||||||||||||||||||
Sbjct  466128  AACAGGATTAGATACCCTGGTAGTCCACGCCGTAAACGATGAGTGCTAGGTGTTAGGCCC  466069
Query  781     TTTCCGGGGCTTAGTGCCGCAGCTAACGCATTAAGCACTC  820
               ||||||||||||||||||||||||||||||||||||||||
Sbjct  466068  TTTCCGGGGCTTAGTGCCGCAGCTAACGCATTAAGCACTC  466029
```

Range 6: 307243 to 308062

Score	Expect	Identities	Gaps	Strand	Frame
1509 bits(817)	0.0()	819/820(99%)	0/820(0%)	Plus/Minus	

Features:

```
Query  1       CGCTGATGTTTGGTGTTTACACTAGACTGATGAGTTGCGAACGGGTGAGTAACGCGTAGG  60
               ||||||||||||||||||||||||||||||||||||||||||||||||||||||||||||
Sbjct  308062  CGCTGATGTTTGGTGTTTACACTAGACTGATGAGTTGCGAACGGGTGAGTAACGCGTAGG  308003
Query  61      TAACCTGCCTCATAGCGGGGGATAACTATTGGAAACGATAGCTAATACCGCATAAGAGTA  120
               ||||||||||||||||||||||||||||||||||||||||||||||||||||||||||| 
Sbjct  308002  TAACCTGCCTCATAGCGGGGGATAACTATTGGAAACGATAGCTAATACCGCATAAGAGTG  307943
Query  121     ATTAACACATGTTAGTTATTTAAAAGGAGCAATTGCTTCACTGTGAGATGGACCTGCGTT  180
               ||||||||||||||||||||||||||||||||||||||||||||||||||||||||||||
Sbjct  307942  ATTAACACATGTTAGTTATTTAAAAGGAGCAATTGCTTCACTGTGAGATGGACCTGCGTT  307883
Query  181     GTATTAGCTAGTTGGTGAGGTAAAGGCTCACCAAGGCGACGATACATAGCCGACCTGAGA  240
               ||||||||||||||||||||||||||||||||||||||||||||||||||||||||||||
Sbjct  307882  GTATTAGCTAGTTGGTGAGGTAAAGGCTCACCAAGGCGACGATACATAGCCGACCTGAGA  307823
Query  241     GGGTGATCGGCCACACTGGGACTGAGACACGGCCCAGACTCCTACGGGAGGCAGCAGTAG  300
               ||||||||||||||||||||||||||||||||||||||||||||||||||||||||||||
Sbjct  307822  GGGTGATCGGCCACACTGGGACTGAGACACGGCCCAGACTCCTACGGGAGGCAGCAGTAG  307763
Query  301     GGAATCTTCGGCAATGGACGGAAGTCTGACCGAGCAACGCCGCGTGAGTGAAGAAGGTTT  360
               ||||||||||||||||||||||||||||||||||||||||||||||||||||||||||||
Sbjct  307762  GGAATCTTCGGCAATGGACGGAAGTCTGACCGAGCAACGCCGCGTGAGTGAAGAAGGTTT  307703
Query  361     TCGGATCGTAAAGCTCTGTTGTTAGAGAAGAACGTTGGTAGGAGTGGAAAATCTACCAAG  420
               ||||||||||||||||||||||||||||||||||||||||||||||||||||||||||||
Sbjct  307702  TCGGATCGTAAAGCTCTGTTGTTAGAGAAGAACGTTGGTAGGAGTGGAAAATCTACCAAG  307643
Query  421     TGACGGTAACTAACCAGAAAGGGACGGCTAACTACGTGCCAGCAGCCGCGGTAATACGTA  480
               ||||||||||||||||||||||||||||||||||||||||||||||||||||||||||||
Sbjct  307642  TGACGGTAACTAACCAGAAAGGGACGGCTAACTACGTGCCAGCAGCCGCGGTAATACGTA  307583
Query  481     GGTCCCGAGCGTTGTCCGGATTTATTGGGCGTAAAGCGAGCGCAGGCGGTTCTTTAAGTC  540
               ||||||||||||||||||||||||||||||||||||||||||||||||||||||||||||
Sbjct  307582  GGTCCCGAGCGTTGTCCGGATTTATTGGGCGTAAAGCGAGCGCAGGCGGTTCTTTAAGTC  307523
Query  541     TGAAGTTAAAGGCAGTGGCTTAACCATTGTACGCTTTGGAAACTGGAGGACTTGAGTGCA  600
               ||||||||||||||||||||||||||||||||||||||||||||||||||||||||||||
Sbjct  307522  TGAAGTTAAAGGCAGTGGCTTAACCATTGTACGCTTTGGAAACTGGAGGACTTGAGTGCA  307463
Query  601     GAAGGGGAGAGTGGAATTCCATGTGTAGCGGTGAAATGCGTAGATATATGGAGGAACACC  660
               ||||||||||||||||||||||||||||||||||||||||||||||||||||||||||||
Sbjct  307462  GAAGGGGAGAGTGGAATTCCATGTGTAGCGGTGAAATGCGTAGATATATGGAGGAACACC  307403
Query  661     GGTGGCGAAAGCGGCTCTCTGGTCTGTAACTGACGCTGAGGCTCGAAAGCGTGGGGAGCA  720
               ||||||||||||||||||||||||||||||||||||||||||||||||||||||||||||
Sbjct  307402  GGTGGCGAAAGCGGCTCTCTGGTCTGTAACTGACGCTGAGGCTCGAAAGCGTGGGGAGCA  307343
Query  721     AACAGGATTAGATACCCTGGTAGTCCACGCCGTAAACGATGAGTGCTAGGTGTTAGGCCC  780
               ||||||||||||||||||||||||||||||||||||||||||||||||||||||||||||
Sbjct  307342  AACAGGATTAGATACCCTGGTAGTCCACGCCGTAAACGATGAGTGCTAGGTGTTAGGCCC  307283
Query  781     TTTCCGGGGCTTAGTGCCGCAGCTAACGCATTAAGCACTC  820
               ||||||||||||||||||||||||||||||||||||||||
Sbjct  307282  TTTCCGGGGCTTAGTGCCGCAGCTAACGCATTAAGCACTC  307243
```

Range 7: 460197 to 461016

Score	Expect	Identities	Gaps	Strand	Frame
1509 bits(817)	0.0()	819/820(99%)	0/820(0%)	Plus/Minus	

Features:

```
Query  1       CGCTGATGTTTGGTGTTTACACTAGACTGATGAGTTGCGAACGGGTGAGTAACGCGTAGG  60
               ||||||||||||||||||||||||||||||||||||||||||||||||||||||||||||
Sbjct  461016  CGCTGATGTTTGGTGTTTACACTAGACTGATGAGTTGCGAACGGGTGAGTAACGCGTAGG  460957
Query  61      TAACCTGCCTCATAGCGGGGGATAACTATTGGAAACGATAGCTAATACCGCATAAGAGTA  120
               ||||||||||||||||||||||||||||||||||||||||||||||||||||||||||| 
Sbjct  460956  TAACCTGCCTCATAGCGGGGGATAACTATTGGAAACGATAGCTAATACCGCATAAGAGTG  460897
Query  121     ATTAACACATGTTAGTTATTTAAAAGGAGCAATTGCTTCACTGTGAGATGGACCTGCGTT  180
               ||||||||||||||||||||||||||||||||||||||||||||||||||||||||||||
Sbjct  460896  ATTAACACATGTTAGTTATTTAAAAGGAGCAATTGCTTCACTGTGAGATGGACCTGCGTT  460837
Query  181     GTATTAGCTAGTTGGTGAGGTAAAGGCTCACCAAGGCGACGATACATAGCCGACCTGAGA  240
               ||||||||||||||||||||||||||||||||||||||||||||||||||||||||||||
Sbjct  460836  GTATTAGCTAGTTGGTGAGGTAAAGGCTCACCAAGGCGACGATACATAGCCGACCTGAGA  460777
Query  241     GGGTGATCGGCCACACTGGGACTGAGACACGGCCCAGACTCCTACGGGAGGCAGCAGTAG  300
               ||||||||||||||||||||||||||||||||||||||||||||||||||||||||||||
Sbjct  460776  GGGTGATCGGCCACACTGGGACTGAGACACGGCCCAGACTCCTACGGGAGGCAGCAGTAG  460717
Query  301     GGAATCTTCGGCAATGGACGGAAGTCTGACCGAGCAACGCCGCGTGAGTGAAGAAGGTTT  360
               ||||||||||||||||||||||||||||||||||||||||||||||||||||||||||||
Sbjct  460716  GGAATCTTCGGCAATGGACGGAAGTCTGACCGAGCAACGCCGCGTGAGTGAAGAAGGTTT  460657
Query  361     TCGGATCGTAAAGCTCTGTTGTTAGAGAAGAACGTTGGTAGGAGTGGAAAATCTACCAAG  420
               ||||||||||||||||||||||||||||||||||||||||||||||||||||||||||||
Sbjct  460656  TCGGATCGTAAAGCTCTGTTGTTAGAGAAGAACGTTGGTAGGAGTGGAAAATCTACCAAG  460597
Query  421     TGACGGTAACTAACCAGAAAGGGACGGCTAACTACGTGCCAGCAGCCGCGGTAATACGTA  480
               ||||||||||||||||||||||||||||||||||||||||||||||||||||||||||||
Sbjct  460596  TGACGGTAACTAACCAGAAAGGGACGGCTAACTACGTGCCAGCAGCCGCGGTAATACGTA  460537
Query  481     GGTCCCGAGCGTTGTCCGGATTTATTGGGCGTAAAGCGAGCGCAGGCGGTTCTTTAAGTC  540
               ||||||||||||||||||||||||||||||||||||||||||||||||||||||||||||
Sbjct  460536  GGTCCCGAGCGTTGTCCGGATTTATTGGGCGTAAAGCGAGCGCAGGCGGTTCTTTAAGTC  460477
Query  541     TGAAGTTAAAGGCAGTGGCTTAACCATTGTACGCTTTGGAAACTGGAGGACTTGAGTGCA  600
               ||||||||||||||||||||||||||||||||||||||||||||||||||||||||||||
Sbjct  460476  TGAAGTTAAAGGCAGTGGCTTAACCATTGTACGCTTTGGAAACTGGAGGACTTGAGTGCA  460417
Query  601     GAAGGGGAGAGTGGAATTCCATGTGTAGCGGTGAAATGCGTAGATATATGGAGGAACACC  660
```

```
            ||||||||||||||||||||||||||||||||||||||||||||||||||||||||||||
Sbjct  460416  GAAGGGGAGAGTGGAATTCCATGTGTAGCGGTGAAATGCGTAGATATATGGAGGAACACC  460357

Query  661     GGTGGCGAAAGCGGCTCTCTGGTCTGTAACTGACGCTGAGGCTCGAAAGCGTGGGGAGCA  720
               ||||||||||||||||||||||||||||||||||||||||||||||||||||||||||||
Sbjct  460356  GGTGGCGAAAGCGGCTCTCTGGTCTGTAACTGACGCTGAGGCTCGAAAGCGTGGGGAGCA  460297

Query  721     AACAGGATTAGATACCCTGGTAGTCCACGCCGTAAACGATGAGTGCTAGGTGTTAGGCCC  780
               ||||||||||||||||||||||||||||||||||||||||||||||||||||||||||||
Sbjct  460296  AACAGGATTAGATACCCTGGTAGTCCACGCCGTAAACGATGAGTGCTAGGTGTTAGGCCC  460237

Query  781     TTTCCGGGGCTTAGTGCCGCAGCTAACGCATTAAGCACTC  820
               ||||||||||||||||||||||||||||||||||||||||
Sbjct  460236  TTTCCGGGGCTTAGTGCCGCAGCTAACGCATTAAGCACTC  460197
```

Streptococcus agalactiae strain B7 16S ribosomal RNA gene, partial sequence
Sequence ID: **JN176347.1** Length: 1358 Number of Matches: 1
Range 1: 48 to 867

Score	Expect	Identities	Gaps	Strand	Frame
1515 bits(820)	0.0()	820/820(100%)	0/820(0%)	Plus/Plus	

Features:

```
Query  1    CGCTGATGTTTGGTGTTTACACTAGACTGATGAGTTGCGAACGGGTGAGTAACGCGTAGG  60
            ||||||||||||||||||||||||||||||||||||||||||||||||||||||||||||
Sbjct  48   CGCTGATGTTTGGTGTTTACACTAGACTGATGAGTTGCGAACGGGTGAGTAACGCGTAGG  107

Query  61   TAACCTGCCTCATAGCGGGGGATAACTATTGGAAACGATAGCTAATACCGCATAAGAGTA  120
            ||||||||||||||||||||||||||||||||||||||||||||||||||||||||||||
Sbjct  108  TAACCTGCCTCATAGCGGGGGATAACTATTGGAAACGATAGCTAATACCGCATAAGAGTA  167

Query  121  ATTAACACATGTTAGTTATTTAAAAGGAGCAATTGCTTCACTGTGAGATGGACCTGCGTT  180
            ||||||||||||||||||||||||||||||||||||||||||||||||||||||||||||
Sbjct  168  ATTAACACATGTTAGTTATTTAAAAGGAGCAATTGCTTCACTGTGAGATGGACCTGCGTT  227

Query  181  GTATTAGCTAGTTGGTGAGGTAAAGGCTCACCAAGGCGACGATACATAGCCGACCTGAGA  240
            ||||||||||||||||||||||||||||||||||||||||||||||||||||||||||||
Sbjct  228  GTATTAGCTAGTTGGTGAGGTAAAGGCTCACCAAGGCGACGATACATAGCCGACCTGAGA  287

Query  241  GGGTGATCGGCCACACTGGGACTGAGACACGGCCCAGACTCCTACGGGAGGCAGCAGTAG  300
            ||||||||||||||||||||||||||||||||||||||||||||||||||||||||||||
Sbjct  288  GGGTGATCGGCCACACTGGGACTGAGACACGGCCCAGACTCCTACGGGAGGCAGCAGTAG  347

Query  301  GGAATCTTCGGCAATGGACGGAAGTCTGACCGAGCAACGCCGCGTGAGTGAAGAAGGTTT  360
            ||||||||||||||||||||||||||||||||||||||||||||||||||||||||||||
Sbjct  348  GGAATCTTCGGCAATGGACGGAAGTCTGACCGAGCAACGCCGCGTGAGTGAAGAAGGTTT  407

Query  361  TCGGATCGTAAAGCTCTGTTGTTAGAGAAGAACGTTGGTAGGAGTGGAAAATCTACCAAG  420
            ||||||||||||||||||||||||||||||||||||||||||||||||||||||||||||
Sbjct  408  TCGGATCGTAAAGCTCTGTTGTTAGAGAAGAACGTTGGTAGGAGTGGAAAATCTACCAAG  467

Query  421  TGACGGTAACTAACCAGAAAGGGACGGCTAACTACGTGCCAGCAGCCGCGGTAATACGTA  480
            ||||||||||||||||||||||||||||||||||||||||||||||||||||||||||||
Sbjct  468  TGACGGTAACTAACCAGAAAGGGACGGCTAACTACGTGCCAGCAGCCGCGGTAATACGTA  527

Query  481  GGTCCCGAGCGTTGTCCGGATTTATTGGGCGTAAAGCGAGCGCAGGCGGTTCTTTAAGTC  540
            ||||||||||||||||||||||||||||||||||||||||||||||||||||||||||||
Sbjct  528  GGTCCCGAGCGTTGTCCGGATTTATTGGGCGTAAAGCGAGCGCAGGCGGTTCTTTAAGTC  587

Query  541  TGAAGTTAAAGGCAGTGGCTTAACCATTGTACGCTTTGGAAACTGGAGGACTTGAGTGCA  600
            ||||||||||||||||||||||||||||||||||||||||||||||||||||||||||||
Sbjct  588  TGAAGTTAAAGGCAGTGGCTTAACCATTGTACGCTTTGGAAACTGGAGGACTTGAGTGCA  647

Query  601  GAAGGGGAGAGTGGAATTCCATGTGTAGCGGTGAAATGCGTAGATATATGGAGGAACACC  660
            ||||||||||||||||||||||||||||||||||||||||||||||||||||||||||||
Sbjct  648  GAAGGGGAGAGTGGAATTCCATGTGTAGCGGTGAAATGCGTAGATATATGGAGGAACACC  707

Query  661  GGTGGCGAAAGCGGCTCTCTGGTCTGTAACTGACGCTGAGGCTCGAAAGCGTGGGGAGCA  720
            ||||||||||||||||||||||||||||||||||||||||||||||||||||||||||||
Sbjct  708  GGTGGCGAAAGCGGCTCTCTGGTCTGTAACTGACGCTGAGGCTCGAAAGCGTGGGGAGCA  767

Query  721  AACAGGATTAGATACCCTGGTAGTCCACGCCGTAAACGATGAGTGCTAGGTGTTAGGCCC  780
            ||||||||||||||||||||||||||||||||||||||||||||||||||||||||||||
Sbjct  768  AACAGGATTAGATACCCTGGTAGTCCACGCCGTAAACGATGAGTGCTAGGTGTTAGGCCC  827

Query  781  TTTCCGGGGCTTAGTGCCGCAGCTAACGCATTAAGCACTC  820
            ||||||||||||||||||||||||||||||||||||||||
Sbjct  828  TTTCCGGGGCTTAGTGCCGCAGCTAACGCATTAAGCACTC  867
```

Uncultured bacterium clone GoC_Bac_17_D1_C0_M0 16S ribosomal RNA gene, partial sequence
Sequence ID: **FJ813519.1** Length: 1508 Number of Matches: 1
Range 1: 60 to 879

Score	Expect	Identities	Gaps	Strand	Frame
1515 bits(820)	0.0()	820/820(100%)	0/820(0%)	Plus/Plus	

Features:

```
Query  1    CGCTGATGTTTGGTGTTTACACTAGACTGATGAGTTGCGAACGGGTGAGTAACGCGTAGG  60
            ||||||||||||||||||||||||||||||||||||||||||||||||||||||||||||
Sbjct  60   CGCTGATGTTTGGTGTTTACACTAGACTGATGAGTTGCGAACGGGTGAGTAACGCGTAGG  119

Query  61   TAACCTGCCTCATAGCGGGGGATAACTATTGGAAACGATAGCTAATACCGCATAAGAGTA  120
            ||||||||||||||||||||||||||||||||||||||||||||||||||||||||||||
Sbjct  120  TAACCTGCCTCATAGCGGGGGATAACTATTGGAAACGATAGCTAATACCGCATAAGAGTA  179

Query  121  ATTAACACATGTTAGTTATTTAAAAGGAGCAATTGCTTCACTGTGAGATGGACCTGCGTT  180
            ||||||||||||||||||||||||||||||||||||||||||||||||||||||||||||
Sbjct  180  ATTAACACATGTTAGTTATTTAAAAGGAGCAATTGCTTCACTGTGAGATGGACCTGCGTT  239

Query  181  GTATTAGCTAGTTGGTGAGGTAAAGGCTCACCAAGGCGACGATACATAGCCGACCTGAGA  240
            ||||||||||||||||||||||||||||||||||||||||||||||||||||||||||||
Sbjct  240  GTATTAGCTAGTTGGTGAGGTAAAGGCTCACCAAGGCGACGATACATAGCCGACCTGAGA  299

Query  241  GGGTGATCGGCCACACTGGGACTGAGACACGGCCCAGACTCCTACGGGAGGCAGCAGTAG  300
            ||||||||||||||||||||||||||||||||||||||||||||||||||||||||||||
Sbjct  300  GGGTGATCGGCCACACTGGGACTGAGACACGGCCCAGACTCCTACGGGAGGCAGCAGTAG  359

Query  301  GGAATCTTCGGCAATGGACGGAAGTCTGACCGAGCAACGCCGCGTGAGTGAAGAAGGTTT  360
            ||||||||||||||||||||||||||||||||||||||||||||||||||||||||||||
Sbjct  360  GGAATCTTCGGCAATGGACGGAAGTCTGACCGAGCAACGCCGCGTGAGTGAAGAAGGTTT  419

Query  361  TCGGATCGTAAAGCTCTGTTGTTAGAGAAGAACGTTGGTAGGAGTGGAAAATCTACCAAG  420
            ||||||||||||||||||||||||||||||||||||||||||||||||||||||||||||
Sbjct  420  TCGGATCGTAAAGCTCTGTTGTTAGAGAAGAACGTTGGTAGGAGTGGAAAATCTACCAAG  479

Query  421  TGACGGTAACTAACCAGAAAGGGACGGCTAACTACGTGCCAGCAGCCGCGGTAATACGTA  480
            ||||||||||||||||||||||||||||||||||||||||||||||||||||||||||||
Sbjct  480  TGACGGTAACTAACCAGAAAGGGACGGCTAACTACGTGCCAGCAGCCGCGGTAATACGTA  539

Query  481  GGTCCCGAGCGTTGTCCGGATTTATTGGGCGTAAAGCGAGCGCAGGCGGTTCTTTAAGTC  540
            ||||||||||||||||||||||||||||||||||||||||||||||||||||||||||||
Sbjct  540  GGTCCCGAGCGTTGTCCGGATTTATTGGGCGTAAAGCGAGCGCAGGCGGTTCTTTAAGTC  599

Query  541  TGAAGTTAAAGGCAGTGGCTTAACCATTGTACGCTTTGGAAACTGGAGGACTTGAGTGCA  600
            ||||||||||||||||||||||||||||||||||||||||||||||||||||||||||||
Sbjct  600  TGAAGTTAAAGGCAGTGGCTTAACCATTGTACGCTTTGGAAACTGGAGGACTTGAGTGCA  659

Query  601  GAAGGGGAGAGTGGAATTCCATGTGTAGCGGTGAAATGCGTAGATATATGGAGGAACACC  660
            ||||||||||||||||||||||||||||||||||||||||||||||||||||||||||||
Sbjct  660  GAAGGGGAGAGTGGAATTCCATGTGTAGCGGTGAAATGCGTAGATATATGGAGGAACACC  719

Query  661  GGTGGCGAAAGCGGCTCTCTGGTCTGTAACTGACGCTGAGGCTCGAAAGCGTGGGGAGCA  720
            ||||||||||||||||||||||||||||||||||||||||||||||||||||||||||||
Sbjct  720  GGTGGCGAAAGCGGCTCTCTGGTCTGTAACTGACGCTGAGGCTCGAAAGCGTGGGGAGCA  779

Query  721  AACAGGATTAGATACCCTGGTAGTCCACGCCGTAAACGATGAGTGCTAGGTGTTAGGCCC  780
            ||||||||||||||||||||||||||||||||||||||||||||||||||||||||||||
Sbjct  780  AACAGGATTAGATACCCTGGTAGTCCACGCCGTAAACGATGAGTGCTAGGTGTTAGGCCC  839

Query  781  TTTCCGGGGCTTAGTGCCGCAGCTAACGCATTAAGCACTC  820
            ||||||||||||||||||||||||||||||||||||||||
Sbjct  840  TTTCCGGGGCTTAGTGCCGCAGCTAACGCATTAAGCACTC  879
```

Streptococcus agalactiae strain 14-ninetytwomp 16S ribosomal RNA gene, partial sequence
Sequence ID: **EU075069.1** Length: 1449 Number of Matches: 1
Range 1: 61 to 880

Score	Expect	Identities	Gaps	Strand	Frame
1515 bits(820)	0.0()	820/820(100%)	0/820(0%)	Plus/Plus	

Features:

```
Query  1    CGCTGATGTTTGGTGTTTACACTAGACTGATGAGTTGCGAACGGGTGAGTAACGCGTAGG  60
            ||||||||||||||||||||||||||||||||||||||||||||||||||||||||||||
Sbjct  61   CGCTGATGTTTGGTGTTTACACTAGACTGATGAGTTGCGAACGGGTGAGTAACGCGTAGG  120
Query  61   TAACCTGCCTCATAGCGGGGGATAACTATTGGAAACGATAGCTAATACCGCATAAGAGTA  120
            ||||||||||||||||||||||||||||||||||||||||||||||||||||||||||||
Sbjct  121  TAACCTGCCTCATAGCGGGGGATAACTATTGGAAACGATAGCTAATACCGCATAAGAGTA  180
Query  121  ATTAACACATGTTAGTTATTTAAAAGGAGCAATTGCTTCACTGTGAGATGGACCTGCGTT  180
            ||||||||||||||||||||||||||||||||||||||||||||||||||||||||||||
Sbjct  181  ATTAACACATGTTAGTTATTTAAAAGGAGCAATTGCTTCACTGTGAGATGGACCTGCGTT  240
Query  181  GTATTAGCTAGTTGGTGAGGTAAAGGCTCACCAAGGCGACGATACATAGCCGACCTGAGA  240
            ||||||||||||||||||||||||||||||||||||||||||||||||||||||||||||
Sbjct  241  GTATTAGCTAGTTGGTGAGGTAAAGGCTCACCAAGGCGACGATACATAGCCGACCTGAGA  300
Query  241  GGGTGATCGGCCACACTGGGACTGAGACACGGCCCAGACTCCTACGGGAGGCAGCAGTAG  300
            ||||||||||||||||||||||||||||||||||||||||||||||||||||||||||||
Sbjct  301  GGGTGATCGGCCACACTGGGACTGAGACACGGCCCAGACTCCTACGGGAGGCAGCAGTAG  360
Query  301  GGAATCTTCGGCAATGGACGGAAGTCTGACCGAGCAACGCCGCGTGAGTGAAGAAGGTTT  360
            ||||||||||||||||||||||||||||||||||||||||||||||||||||||||||||
Sbjct  361  GGAATCTTCGGCAATGGACGGAAGTCTGACCGAGCAACGCCGCGTGAGTGAAGAAGGTTT  420
Query  361  TCGGATCGTAAAGCTCTGTTGTTAGAGAAGAACGTTGGTAGGAGTGGAAAATCTACCAAG  420
            ||||||||||||||||||||||||||||||||||||||||||||||||||||||||||||
Sbjct  421  TCGGATCGTAAAGCTCTGTTGTTAGAGAAGAACGTTGGTAGGAGTGGAAAATCTACCAAG  480
Query  421  TGACGGTAACTAACCAGAAAGGGACGGCTAACTACGTGCCAGCAGCCGCGGTAATACGTA  480
            ||||||||||||||||||||||||||||||||||||||||||||||||||||||||||||
Sbjct  481  TGACGGTAACTAACCAGAAAGGGACGGCTAACTACGTGCCAGCAGCCGCGGTAATACGTA  540
Query  481  GGTCCCGAGCGTTGTCCGGATTTATTGGGCGTAAAGCGAGCGCAGGCGGTTCTTTAAGTC  540
            ||||||||||||||||||||||||||||||||||||||||||||||||||||||||||||
Sbjct  541  GGTCCCGAGCGTTGTCCGGATTTATTGGGCGTAAAGCGAGCGCAGGCGGTTCTTTAAGTC  600
Query  541  TGAAGTTAAAGGCAGTGGCTTAACCATTGTACGCTTTGGAAACTGGAGGACTTGAGTGCA  600
            ||||||||||||||||||||||||||||||||||||||||||||||||||||||||||||
Sbjct  601  TGAAGTTAAAGGCAGTGGCTTAACCATTGTACGCTTTGGAAACTGGAGGACTTGAGTGCA  660
Query  601  GAAGGGGAGAGTGGAATTCCATGTGTAGCGGTGAAATGCGTAGATATATGGAGGAACACC  660
            ||||||||||||||||||||||||||||||||||||||||||||||||||||||||||||
Sbjct  661  GAAGGGGAGAGTGGAATTCCATGTGTAGCGGTGAAATGCGTAGATATATGGAGGAACACC  720
Query  661  GGTGGCGAAAGCGGCTCTCTGGTCTGTAACTGACGCTGAGGCTCGAAAGCGTGGGGAGCA  720
            ||||||||||||||||||||||||||||||||||||||||||||||||||||||||||||
Sbjct  721  GGTGGCGAAAGCGGCTCTCTGGTCTGTAACTGACGCTGAGGCTCGAAAGCGTGGGGAGCA  780
Query  721  AACAGGATTAGATACCCTGGTAGTCCACGCCGTAAACGATGAGTGCTAGGTGTTAGGCCC  780
            ||||||||||||||||||||||||||||||||||||||||||||||||||||||||||||
Sbjct  781  AACAGGATTAGATACCCTGGTAGTCCACGCCGTAAACGATGAGTGCTAGGTGTTAGGCCC  840
Query  781  TTTCCGGGGCTTAGTGCCGCAGCTAACGCATTAAGCACTC  820
            ||||||||||||||||||||||||||||||||||||||||
Sbjct  841  TTTCCGGGGCTTAGTGCCGCAGCTAACGCATTAAGCACTC  880
```

Streptococcus agalactiae A909, complete genome
Sequence ID: **CP000114.1** Length: 2127839 Number of Matches: 7
Range 1: 358722 to 359541

Score	Expect	Identities	Gaps	Strand	Frame
1515 bits(820)	0.0()	820/820(100%)	0/820(0%)	Plus/Plus	

Features:

```
Query  1       CGCTGATGTTTGGTGTTTACACTAGACTGATGAGTTGCGAACGGGTGAGTAACGCGTAGG  60
               ||||||||||||||||||||||||||||||||||||||||||||||||||||||||||||
Sbjct  358722  CGCTGATGTTTGGTGTTTACACTAGACTGATGAGTTGCGAACGGGTGAGTAACGCGTAGG  358781
Query  61      TAACCTGCCTCATAGCGGGGGATAACTATTGGAAACGATAGCTAATACCGCATAAGAGTA  120
               ||||||||||||||||||||||||||||||||||||||||||||||||||||||||||||
Sbjct  358782  TAACCTGCCTCATAGCGGGGGATAACTATTGGAAACGATAGCTAATACCGCATAAGAGTA  358841
Query  121     ATTAACACATGTTAGTTATTTAAAAGGAGCAATTGCTTCACTGTGAGATGGACCTGCGTT  180
               ||||||||||||||||||||||||||||||||||||||||||||||||||||||||||||
Sbjct  358842  ATTAACACATGTTAGTTATTTAAAAGGAGCAATTGCTTCACTGTGAGATGGACCTGCGTT  358901
Query  181     GTATTAGCTAGTTGGTGAGGTAAAGGCTCACCAAGGCGACGATACATAGCCGACCTGAGA  240
               ||||||||||||||||||||||||||||||||||||||||||||||||||||||||||||
Sbjct  358902  GTATTAGCTAGTTGGTGAGGTAAAGGCTCACCAAGGCGACGATACATAGCCGACCTGAGA  358961
Query  241     GGGTGATCGGCCACACTGGGACTGAGACACGGCCCAGACTCCTACGGGAGGCAGCAGTAG  300
               ||||||||||||||||||||||||||||||||||||||||||||||||||||||||||||
Sbjct  358962  GGGTGATCGGCCACACTGGGACTGAGACACGGCCCAGACTCCTACGGGAGGCAGCAGTAG  359021
Query  301     GGAATCTTCGGCAATGGACGGAAGTCTGACCGAGCAACGCCGCGTGAGTGAAGAAGGTTT  360
               ||||||||||||||||||||||||||||||||||||||||||||||||||||||||||||
Sbjct  359022  GGAATCTTCGGCAATGGACGGAAGTCTGACCGAGCAACGCCGCGTGAGTGAAGAAGGTTT  359081
Query  361     TCGGATCGTAAAGCTCTGTTGTTAGAGAAGAACGTTGGTAGGAGTGGAAAATCTACCAAG  420
               ||||||||||||||||||||||||||||||||||||||||||||||||||||||||||||
Sbjct  359082  TCGGATCGTAAAGCTCTGTTGTTAGAGAAGAACGTTGGTAGGAGTGGAAAATCTACCAAG  359141
Query  421     TGACGGTAACTAACCAGAAAGGGACGGCTAACTACGTGCCAGCAGCCGCGGTAATACGTA  480
               ||||||||||||||||||||||||||||||||||||||||||||||||||||||||||||
Sbjct  359142  TGACGGTAACTAACCAGAAAGGGACGGCTAACTACGTGCCAGCAGCCGCGGTAATACGTA  359201
Query  481     GGTCCCGAGCGTTGTCCGGATTTATTGGGCGTAAAGCGAGCGCAGGCGGTTCTTTAAGTC  540
               ||||||||||||||||||||||||||||||||||||||||||||||||||||||||||||
Sbjct  359202  GGTCCCGAGCGTTGTCCGGATTTATTGGGCGTAAAGCGAGCGCAGGCGGTTCTTTAAGTC  359261
Query  541     TGAAGTTAAAGGCAGTGGCTTAACCATTGTACGCTTTGGAAACTGGAGGACTTGAGTGCA  600
               ||||||||||||||||||||||||||||||||||||||||||||||||||||||||||||
Sbjct  359262  TGAAGTTAAAGGCAGTGGCTTAACCATTGTACGCTTTGGAAACTGGAGGACTTGAGTGCA  359321
Query  601     GAAGGGGAGAGTGGAATTCCATGTGTAGCGGTGAAATGCGTAGATATATGGAGGAACACC  660
               ||||||||||||||||||||||||||||||||||||||||||||||||||||||||||||
Sbjct  359322  GAAGGGGAGAGTGGAATTCCATGTGTAGCGGTGAAATGCGTAGATATATGGAGGAACACC  359381
Query  661     GGTGGCGAAAGCGGCTCTCTGGTCTGTAACTGACGCTGAGGCTCGAAAGCGTGGGGAGCA  720
               ||||||||||||||||||||||||||||||||||||||||||||||||||||||||||||
Sbjct  359382  GGTGGCGAAAGCGGCTCTCTGGTCTGTAACTGACGCTGAGGCTCGAAAGCGTGGGGAGCA  359441
Query  721     AACAGGATTAGATACCCTGGTAGTCCACGCCGTAAACGATGAGTGCTAGGTGTTAGGCCC  780
               ||||||||||||||||||||||||||||||||||||||||||||||||||||||||||||
Sbjct  359442  AACAGGATTAGATACCCTGGTAGTCCACGCCGTAAACGATGAGTGCTAGGTGTTAGGCCC  359501
Query  781     TTTCCGGGGCTTAGTGCCGCAGCTAACGCATTAAGCACTC  820
               ||||||||||||||||||||||||||||||||||||||||
Sbjct  359502  TTTCCGGGGCTTAGTGCCGCAGCTAACGCATTAAGCACTC  359541
```

Range 2: 427865 to 428684

Score	Expect	Identities	Gaps	Strand	Frame
1515 bits(820)	0.0()	820/820(100%)	0/820(0%)	Plus/Plus	

Features:

```
Query  1       CGCTGATGTTTGGTGTTTACACTAGACTGATGAGTTGCGAACGGGTGAGTAACGCGTAGG  60
               ||||||||||||||||||||||||||||||||||||||||||||||||||||||||||||
Sbjct  427865  CGCTGATGTTTGGTGTTTACACTAGACTGATGAGTTGCGAACGGGTGAGTAACGCGTAGG  427924
Query  61      TAACCTGCCTCATAGCGGGGGATAACTATTGGAAACGATAGCTAATACCGCATAAGAGTA  120
               ||||||||||||||||||||||||||||||||||||||||||||||||||||||||||||
Sbjct  427925  TAACCTGCCTCATAGCGGGGGATAACTATTGGAAACGATAGCTAATACCGCATAAGAGTA  427984
Query  121     ATTAACACATGTTAGTTATTTAAAAGGAGCAATTGCTTCACTGTGAGATGGACCTGCGTT  180
               ||||||||||||||||||||||||||||||||||||||||||||||||||||||||||||
Sbjct  427985  ATTAACACATGTTAGTTATTTAAAAGGAGCAATTGCTTCACTGTGAGATGGACCTGCGTT  428044
Query  181     GTATTAGCTAGTTGGTGAGGTAAAGGCTCACCAAGGCGACGATACATAGCCGACCTGAGA  240
               ||||||||||||||||||||||||||||||||||||||||||||||||||||||||||||
Sbjct  428045  GTATTAGCTAGTTGGTGAGGTAAAGGCTCACCAAGGCGACGATACATAGCCGACCTGAGA  428104
Query  241     GGGTGATCGGCCACACTGGGACTGAGACACGGCCCAGACTCCTACGGGAGGCAGCAGTAG  300
               ||||||||||||||||||||||||||||||||||||||||||||||||||||||||||||
Sbjct  428105  GGGTGATCGGCCACACTGGGACTGAGACACGGCCCAGACTCCTACGGGAGGCAGCAGTAG  428164
Query  301     GGAATCTTCGGCAATGGACGGAAGTCTGACCGAGCAACGCCGCGTGAGTGAAGAAGGTTT  360
               ||||||||||||||||||||||||||||||||||||||||||||||||||||||||||||
Sbjct  428165  GGAATCTTCGGCAATGGACGGAAGTCTGACCGAGCAACGCCGCGTGAGTGAAGAAGGTTT  428224
Query  361     TCGGATCGTAAAGCTCTGTTGTTAGAGAAGAACGTTGGTAGGAGTGGAAAATCTACCAAG  420
               ||||||||||||||||||||||||||||||||||||||||||||||||||||||||||||
Sbjct  428225  TCGGATCGTAAAGCTCTGTTGTTAGAGAAGAACGTTGGTAGGAGTGGAAAATCTACCAAG  428284
Query  421     TGACGGTAACTAACCAGAAAGGGACGGCTAACTACGTGCCAGCAGCCGCGGTAATACGTA  480
               ||||||||||||||||||||||||||||||||||||||||||||||||||||||||||||
Sbjct  428285  TGACGGTAACTAACCAGAAAGGGACGGCTAACTACGTGCCAGCAGCCGCGGTAATACGTA  428344
Query  481     GGTCCCGAGCGTTGTCCGGATTTATTGGGCGTAAAGCGAGCGCAGGCGGTTCTTTAAGTC  540
               ||||||||||||||||||||||||||||||||||||||||||||||||||||||||||||
Sbjct  428345  GGTCCCGAGCGTTGTCCGGATTTATTGGGCGTAAAGCGAGCGCAGGCGGTTCTTTAAGTC  428404
Query  541     TGAAGTTAAAGGCAGTGGCTTAACCATTGTACGCTTTGGAAACTGGAGGACTTGAGTGCA  600
               ||||||||||||||||||||||||||||||||||||||||||||||||||||||||||||
Sbjct  428405  TGAAGTTAAAGGCAGTGGCTTAACCATTGTACGCTTTGGAAACTGGAGGACTTGAGTGCA  428464
Query  601     GAAGGGGAGAGTGGAATTCCATGTGTAGCGGTGAAATGCGTAGATATATGGAGGAACACC  660
               ||||||||||||||||||||||||||||||||||||||||||||||||||||||||||||
Sbjct  428465  GAAGGGGAGAGTGGAATTCCATGTGTAGCGGTGAAATGCGTAGATATATGGAGGAACACC  428524
Query  661     GGTGGCGAAAGCGGCTCTCTGGTCTGTAACTGACGCTGAGGCTCGAAAGCGTGGGGAGCA  720
               ||||||||||||||||||||||||||||||||||||||||||||||||||||||||||||
Sbjct  428525  GGTGGCGAAAGCGGCTCTCTGGTCTGTAACTGACGCTGAGGCTCGAAAGCGTGGGGAGCA  428584
Query  721     AACAGGATTAGATACCCTGGTAGTCCACGCCGTAAACGATGAGTGCTAGGTGTTAGGCCC  780
               ||||||||||||||||||||||||||||||||||||||||||||||||||||||||||||
Sbjct  428585  AACAGGATTAGATACCCTGGTAGTCCACGCCGTAAACGATGAGTGCTAGGTGTTAGGCCC  428644
Query  781     TTTCCGGGGCTTAGTGCCGCAGCTAACGCATTAAGCACTC  820
               ||||||||||||||||||||||||||||||||||||||||
Sbjct  428645  TTTCCGGGGCTTAGTGCCGCAGCTAACGCATTAAGCACTC  428684
```

Range 3: 16465 to 17284

Score	Expect	Identities	Gaps	Strand	Frame
1509 bits(817)	0.0()	819/820(99%)	0/820(0%)	Plus/Plus	

Features:

```
Query  1       CGCTGATGTTTGGTGTTTACACTAGACTGATGAGTTGCGAACGGGTGAGTAACGCGTAGG  60
               ||||||||||||||||||||||||||||||||||||||||||||||||||||||||||||
Sbjct  16465   CGCTGATGTTTGGTGTTTACACTAGACTGATGAGTTGCGAACGGGTGAGTAACGCGTAGG  16524
Query  61      TAACCTGCCTCATAGCGGGGGATAACTATTGGAAACGATAGCTAATACCGCATAAGAGTA  120
               ||||||||||||||||||||||||||||||||||||||||||||||||||||||||||| 
Sbjct  16525   TAACCTGCCTCATAGCGGGGGATAACTATTGGAAACGATAGCTAATACCGCATAAGAGTG  16584
Query  121     ATTAACACATGTTAGTTATTTAAAAGGAGCAATTGCTTCACTGTGAGATGGACCTGCGTT  180
               ||||||||||||||||||||||||||||||||||||||||||||||||||||||||||||
Sbjct  16585   ATTAACACATGTTAGTTATTTAAAAGGAGCAATTGCTTCACTGTGAGATGGACCTGCGTT  16644
Query  181     GTATTAGCTAGTTGGTGAGGTAAAGGCTCACCAAGGCGACGATACATAGCCGACCTGAGA  240
               ||||||||||||||||||||||||||||||||||||||||||||||||||||||||||||
Sbjct  16645   GTATTAGCTAGTTGGTGAGGTAAAGGCTCACCAAGGCGACGATACATAGCCGACCTGAGA  16704
Query  241     GGGTGATCGGCCACACTGGGACTGAGACACGGCCCAGACTCCTACGGGAGGCAGCAGTAG  300
               ||||||||||||||||||||||||||||||||||||||||||||||||||||||||||||
Sbjct  16705   GGGTGATCGGCCACACTGGGACTGAGACACGGCCCAGACTCCTACGGGAGGCAGCAGTAG  16764
Query  301     GGAATCTTCGGCAATGGACGGAAGTCTGACCGAGCAACGCCGCGTGAGTGAAGAAGGTTT  360
               ||||||||||||||||||||||||||||||||||||||||||||||||||||||||||||
Sbjct  16765   GGAATCTTCGGCAATGGACGGAAGTCTGACCGAGCAACGCCGCGTGAGTGAAGAAGGTTT  16824
Query  361     TCGGATCGTAAAGCTCTGTTGTTAGAGAAGAACGTTGGTAGGAGTGGAAAATCTACCAAG  420
               ||||||||||||||||||||||||||||||||||||||||||||||||||||||||||||
Sbjct  16825   TCGGATCGTAAAGCTCTGTTGTTAGAGAAGAACGTTGGTAGGAGTGGAAAATCTACCAAG  16884
Query  421     TGACGGTAACTAACCAGAAAGGGACGGCTAACTACGTGCCAGCAGCCGCGGTAATACGTA  480
               ||||||||||||||||||||||||||||||||||||||||||||||||||||||||||||
Sbjct  16885   TGACGGTAACTAACCAGAAAGGGACGGCTAACTACGTGCCAGCAGCCGCGGTAATACGTA  16944
Query  481     GGTCCCGAGCGTTGTCCGGATTTATTGGGCGTAAAGCGAGCGCAGGCGGTTCTTTAAGTC  540
               ||||||||||||||||||||||||||||||||||||||||||||||||||||||||||||
Sbjct  16945   GGTCCCGAGCGTTGTCCGGATTTATTGGGCGTAAAGCGAGCGCAGGCGGTTCTTTAAGTC  17004
Query  541     TGAAGTTAAAGGCAGTGGCTTAACCATTGTACGCTTTGGAAACTGGAGGACTTGAGTGCA  600
               ||||||||||||||||||||||||||||||||||||||||||||||||||||||||||||
Sbjct  17005   TGAAGTTAAAGGCAGTGGCTTAACCATTGTACGCTTTGGAAACTGGAGGACTTGAGTGCA  17064
Query  601     GAAGGGGAGAGTGGAATTCCATGTGTAGCGGTGAAATGCGTAGATATATGGAGGAACACC  660
               ||||||||||||||||||||||||||||||||||||||||||||||||||||||||||||
Sbjct  17065   GAAGGGGAGAGTGGAATTCCATGTGTAGCGGTGAAATGCGTAGATATATGGAGGAACACC  17124
Query  661     GGTGGCGAAAGCGGCTCTCTGGTCTGTAACTGACGCTGAGGCTCGAAAGCGTGGGGAGCA  720
               ||||||||||||||||||||||||||||||||||||||||||||||||||||||||||||
Sbjct  17125   GGTGGCGAAAGCGGCTCTCTGGTCTGTAACTGACGCTGAGGCTCGAAAGCGTGGGGAGCA  17184
Query  721     AACAGGATTAGATACCCTGGTAGTCCACGCCGTAAACGATGAGTGCTAGGTGTTAGGCCC  780
               ||||||||||||||||||||||||||||||||||||||||||||||||||||||||||||
Sbjct  17185   AACAGGATTAGATACCCTGGTAGTCCACGCCGTAAACGATGAGTGCTAGGTGTTAGGCCC  17244
Query  781     TTTCCGGGGCTTAGTGCCGCAGCTAACGCATTAAGCACTC  820
               ||||||||||||||||||||||||||||||||||||||||
Sbjct  17245   TTTCCGGGGCTTAGTGCCGCAGCTAACGCATTAAGCACTC  17284
```

Range 4: 22297 to 23116

Score	Expect	Identities	Gaps	Strand	Frame
1509 bits(817)	0.0()	819/820(99%)	0/820(0%)	Plus/Plus	

Features:

```
Query  1       CGCTGATGTTTGGTGTTTACACTAGACTGATGAGTTGCGAACGGGTGAGTAACGCGTAGG  60
               ||||||||||||||||||||||||||||||||||||||||||||||||||||||||||||
Sbjct  22297   CGCTGATGTTTGGTGTTTACACTAGACTGATGAGTTGCGAACGGGTGAGTAACGCGTAGG  22356
Query  61      TAACCTGCCTCATAGCGGGGGATAACTATTGGAAACGATAGCTAATACCGCATAAGAGTA  120
               ||||||||||||||||||||||||||||||||||||||||||||||||||||||||||| 
Sbjct  22357   TAACCTGCCTCATAGCGGGGGATAACTATTGGAAACGATAGCTAATACCGCATAAGAGTG  22416
Query  121     ATTAACACATGTTAGTTATTTAAAAGGAGCAATTGCTTCACTGTGAGATGGACCTGCGTT  180
               ||||||||||||||||||||||||||||||||||||||||||||||||||||||||||||
Sbjct  22417   ATTAACACATGTTAGTTATTTAAAAGGAGCAATTGCTTCACTGTGAGATGGACCTGCGTT  22476
Query  181     GTATTAGCTAGTTGGTGAGGTAAAGGCTCACCAAGGCGACGATACATAGCCGACCTGAGA  240
               ||||||||||||||||||||||||||||||||||||||||||||||||||||||||||||
Sbjct  22477   GTATTAGCTAGTTGGTGAGGTAAAGGCTCACCAAGGCGACGATACATAGCCGACCTGAGA  22536
Query  241     GGGTGATCGGCCACACTGGGACTGAGACACGGCCCAGACTCCTACGGGAGGCAGCAGTAG  300
               ||||||||||||||||||||||||||||||||||||||||||||||||||||||||||||
Sbjct  22537   GGGTGATCGGCCACACTGGGACTGAGACACGGCCCAGACTCCTACGGGAGGCAGCAGTAG  22596
Query  301     GGAATCTTCGGCAATGGACGGAAGTCTGACCGAGCAACGCCGCGTGAGTGAAGAAGGTTT  360
               ||||||||||||||||||||||||||||||||||||||||||||||||||||||||||||
Sbjct  22597   GGAATCTTCGGCAATGGACGGAAGTCTGACCGAGCAACGCCGCGTGAGTGAAGAAGGTTT  22656
```

```
Query  361    TCGGATCGTAAAGCTCTGTTGTTAGAGAAGAACGTTGGTAGGAGTGGAAAATCTACCAAG  420
Sbjct  22657  TCGGATCGTAAAGCTCTGTTGTTAGAGAAGAACGTTGGTAGGAGTGGAAAATCTACCAAG  22716
Query  421    TGACGGTAACTAACCAGAAAGGGACGGCTAACTACGTGCCAGCAGCCGCGGTAATACGTA  480
Sbjct  22717  TGACGGTAACTAACCAGAAAGGGACGGCTAACTACGTGCCAGCAGCCGCGGTAATACGTA  22776
Query  481    GGTCCCGAGCGTTGTCCGGATTTATTGGGCGTAAAGCGAGCGCAGGCGGTTCTTTAAGTC  540
Sbjct  22777  GGTCCCGAGCGTTGTCCGGATTTATTGGGCGTAAAGCGAGCGCAGGCGGTTCTTTAAGTC  22836
Query  541    TGAAGTTAAAGGCAGTGGCTTAACCATTGTACGCTTTGGAAACTGGAGGACTTGAGTGCA  600
Sbjct  22837  TGAAGTTAAAGGCAGTGGCTTAACCATTGTACGCTTTGGAAACTGGAGGACTTGAGTGCA  22896
Query  601    GAAGGGGAGAGTGGAATTCCATGTGTAGCGGTGAAATGCGTAGATATATGGAGGAACACC  660
Sbjct  22897  GAAGGGGAGAGTGGAATTCCATGTGTAGCGGTGAAATGCGTAGATATATGGAGGAACACC  22956
Query  661    GGTGGCGAAAGCGGCTCTCTGGTCTGTAACTGACGCTGAGGCTCGAAAGCGTGGGGAGCA  720
Sbjct  22957  GGTGGCGAAAGCGGCTCTCTGGTCTGTAACTGACGCTGAGGCTCGAAAGCGTGGGGAGCA  23016
Query  721    AACAGGATTAGATACCCTGGTAGTCCACGCCGTAAACGATGAGTGCTAGGTGTTAGGCCC  780
Sbjct  23017  AACAGGATTAGATACCCTGGTAGTCCACGCCGTAAACGATGAGTGCTAGGTGTTAGGCCC  23076
Query  781    TTTCCGGGGCTTAGTGCCGCAGCTAACGCATTAAGCACTC  820
Sbjct  23077  TTTCCGGGGCTTAGTGCCGCAGCTAACGCATTAAGCACTC  23116
```

Range 5: 90986 to 91805

Score	Expect	Identities	Gaps	Strand	Frame
1509 bits(817)	0.0()	819/820(99%)	0/820(0%)	Plus/Plus	

Features:

```
Query  1      CGCTGATGTTTGGTGTTTACACTAGACTGATGAGTTGCGAACGGGTGAGTAACGCGTAGG  60
Sbjct  90986  CGCTGATGTTTGGTGTTTACACTAGACTGATGAGTTGCGAACGGGTGAGTAACGCGTAGG  91045
Query  61     TAACCTGCCTCATAGCGGGGGATAACTATTGGAAACGATAGCTAATACCGCATAAGAGTA  120
Sbjct  91046  TAACCTGCCTCATAGCGGGGGATAACTATTGGAAACGATAGCTAATACCGCATAAGAGTG  91105
Query  121    ATTAACACATGTTAGTTATTTAAAAGGAGCAATTGCTTCACTGTGAGATGGACCTGCGTT  180
Sbjct  91106  ATTAACACATGTTAGTTATTTAAAAGGAGCAATTGCTTCACTGTGAGATGGACCTGCGTT  91165
Query  181    GTATTAGCTAGTTGGTGAGGTAAAGGCTCACCAAGGCGACGATACATAGCCGACCTGAGA  240
Sbjct  91166  GTATTAGCTAGTTGGTGAGGTAAAGGCTCACCAAGGCGACGATACATAGCCGACCTGAGA  91225
Query  241    GGGTGATCGGCCACACTGGGACTGAGACACGGCCCAGACTCCTACGGGAGGCAGCAGTAG  300
Sbjct  91226  GGGTGATCGGCCACACTGGGACTGAGACACGGCCCAGACTCCTACGGGAGGCAGCAGTAG  91285
Query  301    GGAATCTTCGGCAATGGACGGAAGTCTGACCGAGCAACGCCGCGTGAGTGAAGAAGGTTT  360
Sbjct  91286  GGAATCTTCGGCAATGGACGGAAGTCTGACCGAGCAACGCCGCGTGAGTGAAGAAGGTTT  91345
Query  361    TCGGATCGTAAAGCTCTGTTGTTAGAGAAGAACGTTGGTAGGAGTGGAAAATCTACCAAG  420
Sbjct  91346  TCGGATCGTAAAGCTCTGTTGTTAGAGAAGAACGTTGGTAGGAGTGGAAAATCTACCAAG  91405
Query  421    TGACGGTAACTAACCAGAAAGGGACGGCTAACTACGTGCCAGCAGCCGCGGTAATACGTA  480
Sbjct  91406  TGACGGTAACTAACCAGAAAGGGACGGCTAACTACGTGCCAGCAGCCGCGGTAATACGTA  91465
Query  481    GGTCCCGAGCGTTGTCCGGATTTATTGGGCGTAAAGCGAGCGCAGGCGGTTCTTTAAGTC  540
Sbjct  91466  GGTCCCGAGCGTTGTCCGGATTTATTGGGCGTAAAGCGAGCGCAGGCGGTTCTTTAAGTC  91525
Query  541    TGAAGTTAAAGGCAGTGGCTTAACCATTGTACGCTTTGGAAACTGGAGGACTTGAGTGCA  600
Sbjct  91526  TGAAGTTAAAGGCAGTGGCTTAACCATTGTACGCTTTGGAAACTGGAGGACTTGAGTGCA  91585
Query  601    GAAGGGGAGAGTGGAATTCCATGTGTAGCGGTGAAATGCGTAGATATATGGAGGAACACC  660
Sbjct  91586  GAAGGGGAGAGTGGAATTCCATGTGTAGCGGTGAAATGCGTAGATATATGGAGGAACACC  91645
Query  661    GGTGGCGAAAGCGGCTCTCTGGTCTGTAACTGACGCTGAGGCTCGAAAGCGTGGGGAGCA  720
Sbjct  91646  GGTGGCGAAAGCGGCTCTCTGGTCTGTAACTGACGCTGAGGCTCGAAAGCGTGGGGAGCA  91705
Query  721    AACAGGATTAGATACCCTGGTAGTCCACGCCGTAAACGATGAGTGCTAGGTGTTAGGCCC  780
Sbjct  91706  AACAGGATTAGATACCCTGGTAGTCCACGCCGTAAACGATGAGTGCTAGGTGTTAGGCCC  91765
Query  781    TTTCCGGGGCTTAGTGCCGCAGCTAACGCATTAAGCACTC  820
Sbjct  91766  TTTCCGGGGCTTAGTGCCGCAGCTAACGCATTAAGCACTC  91805
```

Range 6: 175231 to 176050

Score	Expect	Identities	Gaps	Strand	Frame
1509 bits(817)	0.0()	819/820(99%)	0/820(0%)	Plus/Plus	

Features:

```
Query  1       CGCTGATGTTTGGTGTTTACACTAGACTGATGAGTTGCGAACGGGTGAGTAACGCGTAGG  60
Sbjct  175231  CGCTGATGTTTGGTGTTTACACTAGACTGATGAGTTGCGAACGGGTGAGTAACGCGTAGG  175290
Query  61      TAACCTGCCTCATAGCGGGGGATAACTATTGGAAACGATAGCTAATACCGCATAAGAGTA  120
Sbjct  175291  TAACCTGCCTCATAGCGGGGGATAACTATTGGAAACGATAGCTAATACCGCATAAGAGTG  175350
Query  121     ATTAACACATGTTAGTTATTTAAAAGGAGCAATTGCTTCACTGTGAGATGGACCTGCGTT  180
Sbjct  175351  ATTAACACATGTTAGTTATTTAAAAGGAGCAATTGCTTCACTGTGAGATGGACCTGCGTT  175410
Query  181     GTATTAGCTAGTTGGTGAGGTAAAGGCTCACCAAGGCGACGATACATAGCCGACCTGAGA  240
Sbjct  175411  GTATTAGCTAGTTGGTGAGGTAAAGGCTCACCAAGGCGACGATACATAGCCGACCTGAGA  175470
Query  241     GGGTGATCGGCCACACTGGGACTGAGACACGGCCCAGACTCCTACGGGAGGCAGCAGTAG  300
Sbjct  175471  GGGTGATCGGCCACACTGGGACTGAGACACGGCCCAGACTCCTACGGGAGGCAGCAGTAG  175530
Query  301     GGAATCTTCGGCAATGGACGGAAGTCTGACCGAGCAACGCCGCGTGAGTGAAGAAGGTTT  360
Sbjct  175531  GGAATCTTCGGCAATGGACGGAAGTCTGACCGAGCAACGCCGCGTGAGTGAAGAAGGTTT  175590
Query  361     TCGGATCGTAAAGCTCTGTTGTTAGAGAAGAACGTTGGTAGGAGTGGAAAATCTACCAAG  420
Sbjct  175591  TCGGATCGTAAAGCTCTGTTGTTAGAGAAGAACGTTGGTAGGAGTGGAAAATCTACCAAG  175650
Query  421     TGACGGTAACTAACCAGAAAGGGACGGCTAACTACGTGCCAGCAGCCGCGGTAATACGTA  480
Sbjct  175651  TGACGGTAACTAACCAGAAAGGGACGGCTAACTACGTGCCAGCAGCCGCGGTAATACGTA  175710
Query  481     GGTCCCGAGCGTTGTCCGGATTTATTGGGCGTAAAGCGAGCGCAGGCGGTTCTTTAAGTC  540
Sbjct  175711  GGTCCCGAGCGTTGTCCGGATTTATTGGGCGTAAAGCGAGCGCAGGCGGTTCTTTAAGTC  175770
Query  541     TGAAGTTAAAGGCAGTGGCTTAACCATTGTACGCTTTGGAAACTGGAGGACTTGAGTGCA  600
Sbjct  175771  TGAAGTTAAAGGCAGTGGCTTAACCATTGTACGCTTTGGAAACTGGAGGACTTGAGTGCA  175830
Query  601     GAAGGGGAGAGTGGAATTCCATGTGTAGCGGTGAAATGCGTAGATATATGGAGGAACACC  660
Sbjct  175831  GAAGGGGAGAGTGGAATTCCATGTGTAGCGGTGAAATGCGTAGATATATGGAGGAACACC  175890
Query  661     GGTGGCGAAAGCGGCTCTCTGGTCTGTAACTGACGCTGAGGCTCGAAAGCGTGGGGAGCA  720
```

```
Sbjct  175891  GGTGGCGAAAGCGGCTCTCTGGTCTGTAACTGACGCTGAGGCTCGAAAGCGTGGGGAGCA  175950

Query  721     AACAGGATTAGATACCCTGGTAGTCCACGCCGTAAACGATGAGTGCTAGGTGTTAGGCCC  780
               ||||||||||||||||||||||||||||||||||||||||||||||||||||||||||||
Sbjct  175951  AACAGGATTAGATACCCTGGTAGTCCACGCCGTAAACGATGAGTGCTAGGTGTTAGGCCC  176010

Query  781     TTTCCGGGGCTTAGTGCCGCAGCTAACGCATTAAGCACTC  820
               ||||||||||||||||||||||||||||||||||||||||
Sbjct  176011  TTTCCGGGGCTTAGTGCCGCAGCTAACGCATTAAGCACTC  176050
```

Range 7: 259820 to 260639

Score	Expect	Identities	Gaps	Strand	Frame
1509 bits(817)	0.0()	819/820(99%)	0/820(0%)	Plus/Plus	

Features:

```
Query  1       CGCTGATGTTTGGTGTTTACACTAGACTGATGAGTTGCGAACGGGTGAGTAACGCGTAGG  60
               ||||||||||||||||||||||||||||||||||||||||||||||||||||||||||||
Sbjct  259820  CGCTGATGTTTGGTGTTTACACTAGACTGATGAGTTGCGAACGGGTGAGTAACGCGTAGG  259879

Query  61      TAACCTGCCTCATAGCGGGGGATAACTATTGGAAACGATAGCTAATACCGCATAAGAGTA  120
               ||||||||||||||||||||||||||||||||||||||||||||||||||||||||||| 
Sbjct  259880  TAACCTGCCTCATAGCGGGGGATAACTATTGGAAACGATAGCTAATACCGCATAAGAGTG  259939

Query  121     ATTAACACATGTTAGTTATTTAAAAGGAGCAATTGCTTCACTGTGAGATGGACCTGCGTT  180
               ||||||||||||||||||||||||||||||||||||||||||||||||||||||||||||
Sbjct  259940  ATTAACACATGTTAGTTATTTAAAAGGAGCAATTGCTTCACTGTGAGATGGACCTGCGTT  259999

Query  181     GTATTAGCTAGTTGGTGAGGTAAAGGCTCACCAAGGCGACGATACATAGCCGACCTGAGA  240
               ||||||||||||||||||||||||||||||||||||||||||||||||||||||||||||
Sbjct  260000  GTATTAGCTAGTTGGTGAGGTAAAGGCTCACCAAGGCGACGATACATAGCCGACCTGAGA  260059

Query  241     GGGTGATCGGCCACACTGGGACTGAGACACGGCCCAGACTCCTACGGGAGGCAGCAGTAG  300
               ||||||||||||||||||||||||||||||||||||||||||||||||||||||||||||
Sbjct  260060  GGGTGATCGGCCACACTGGGACTGAGACACGGCCCAGACTCCTACGGGAGGCAGCAGTAG  260119

Query  301     GGAATCTTCGGCAATGGACGGAAGTCTGACCGAGCAACGCCGCGTGAGTGAAGAAGGTTT  360
               ||||||||||||||||||||||||||||||||||||||||||||||||||||||||||||
Sbjct  260120  GGAATCTTCGGCAATGGACGGAAGTCTGACCGAGCAACGCCGCGTGAGTGAAGAAGGTTT  260179

Query  361     TCGGATCGTAAAGCTCTGTTGTTAGAGAAGAACGTTGGTAGGAGTGGAAAATCTACCAAG  420
               ||||||||||||||||||||||||||||||||||||||||||||||||||||||||||||
Sbjct  260180  TCGGATCGTAAAGCTCTGTTGTTAGAGAAGAACGTTGGTAGGAGTGGAAAATCTACCAAG  260239

Query  421     TGACGGTAACTAACCAGAAAGGGACGGCTAACTACGTGCCAGCAGCCGCGGTAATACGTA  480
               ||||||||||||||||||||||||||||||||||||||||||||||||||||||||||||
Sbjct  260240  TGACGGTAACTAACCAGAAAGGGACGGCTAACTACGTGCCAGCAGCCGCGGTAATACGTA  260299

Query  481     GGTCCCGAGCGTTGTCCGGATTTATTGGGCGTAAAGCGAGCGCAGGCGGTTCTTTAAGTC  540
               ||||||||||||||||||||||||||||||||||||||||||||||||||||||||||||
Sbjct  260300  GGTCCCGAGCGTTGTCCGGATTTATTGGGCGTAAAGCGAGCGCAGGCGGTTCTTTAAGTC  260359

Query  541     TGAAGTTAAAGGCAGTGGCTTAACCATTGTACGCTTTGGAAACTGGAGGACTTGAGTGCA  600
               ||||||||||||||||||||||||||||||||||||||||||||||||||||||||||||
Sbjct  260360  TGAAGTTAAAGGCAGTGGCTTAACCATTGTACGCTTTGGAAACTGGAGGACTTGAGTGCA  260419

Query  601     GAAGGGGAGAGTGGAATTCCATGTGTAGCGGTGAAATGCGTAGATATATGGAGGAACACC  660
               ||||||||||||||||||||||||||||||||||||||||||||||||||||||||||||
Sbjct  260420  GAAGGGGAGAGTGGAATTCCATGTGTAGCGGTGAAATGCGTAGATATATGGAGGAACACC  260479

Query  661     GGTGGCGAAAGCGGCTCTCTGGTCTGTAACTGACGCTGAGGCTCGAAAGCGTGGGGAGCA  720
               ||||||||||||||||||||||||||||||||||||||||||||||||||||||||||||
Sbjct  260480  GGTGGCGAAAGCGGCTCTCTGGTCTGTAACTGACGCTGAGGCTCGAAAGCGTGGGGAGCA  260539

Query  721     AACAGGATTAGATACCCTGGTAGTCCACGCCGTAAACGATGAGTGCTAGGTGTTAGGCCC  780
               ||||||||||||||||||||||||||||||||||||||||||||||||||||||||||||
Sbjct  260540  AACAGGATTAGATACCCTGGTAGTCCACGCCGTAAACGATGAGTGCTAGGTGTTAGGCCC  260599

Query  781     TTTCCGGGGCTTAGTGCCGCAGCTAACGCATTAAGCACTC  820
               ||||||||||||||||||||||||||||||||||||||||
Sbjct  260600  TTTCCGGGGCTTAGTGCCGCAGCTAACGCATTAAGCACTC  260639
```

Streptococcus agalactiae strain GZ2058 16S ribosomal RNA gene, partial sequence
Sequence ID: **MG386601.1** Length: 1403 Number of Matches: 1
Range 1: 4 to 823

Score	Expect	Identities	Gaps	Strand	Frame
1509 bits(817)	0.0()	819/820(99%)	0/820(0%)	Plus/Plus	

Features:

```
Query  1    CGCTGATGTTTGGTGTTTACACTAGACTGATGAGTTGCGAACGGGTGAGTAACGCGTAGG  60
            |||||| |||||||||||||||||||||||||||||||||||||||||||||||||||||
Sbjct  4    CGCTGAGGTTTGGTGTTTACACTAGACTGATGAGTTGCGAACGGGTGAGTAACGCGTAGG  63

Query  61   TAACCTGCCTCATAGCGGGGGATAACTATTGGAAACGATAGCTAATACCGCATAAGAGTA  120
            ||||||||||||||||||||||||||||||||||||||||||||||||||||||||||||
Sbjct  64   TAACCTGCCTCATAGCGGGGGATAACTATTGGAAACGATAGCTAATACCGCATAAGAGTA  123

Query  121  ATTAACACATGTTAGTTATTTAAAAGGAGCAATTGCTTCACTGTGAGATGGACCTGCGTT  180
            ||||||||||||||||||||||||||||||||||||||||||||||||||||||||||||
Sbjct  124  ATTAACACATGTTAGTTATTTAAAAGGAGCAATTGCTTCACTGTGAGATGGACCTGCGTT  183

Query  181  GTATTAGCTAGTTGGTGAGGTAAAGGCTCACCAAGGCGACGATACATAGCCGACCTGAGA  240
            ||||||||||||||||||||||||||||||||||||||||||||||||||||||||||||
Sbjct  184  GTATTAGCTAGTTGGTGAGGTAAAGGCTCACCAAGGCGACGATACATAGCCGACCTGAGA  243

Query  241  GGGTGATCGGCCACACTGGGACTGAGACACGGCCCAGACTCCTACGGGAGGCAGCAGTAG  300
            ||||||||||||||||||||||||||||||||||||||||||||||||||||||||||||
Sbjct  244  GGGTGATCGGCCACACTGGGACTGAGACACGGCCCAGACTCCTACGGGAGGCAGCAGTAG  303

Query  301  GGAATCTTCGGCAATGGACGGAAGTCTGACCGAGCAACGCCGCGTGAGTGAAGAAGGTTT  360
            ||||||||||||||||||||||||||||||||||||||||||||||||||||||||||||
Sbjct  304  GGAATCTTCGGCAATGGACGGAAGTCTGACCGAGCAACGCCGCGTGAGTGAAGAAGGTTT  363

Query  361  TCGGATCGTAAAGCTCTGTTGTTAGAGAAGAACGTTGGTAGGAGTGGAAAATCTACCAAG  420
            ||||||||||||||||||||||||||||||||||||||||||||||||||||||||||||
Sbjct  364  TCGGATCGTAAAGCTCTGTTGTTAGAGAAGAACGTTGGTAGGAGTGGAAAATCTACCAAG  423

Query  421  TGACGGTAACTAACCAGAAAGGGACGGCTAACTACGTGCCAGCAGCCGCGGTAATACGTA  480
            ||||||||||||||||||||||||||||||||||||||||||||||||||||||||||||
Sbjct  424  TGACGGTAACTAACCAGAAAGGGACGGCTAACTACGTGCCAGCAGCCGCGGTAATACGTA  483

Query  481  GGTCCCGAGCGTTGTCCGGATTTATTGGGCGTAAAGCGAGCGCAGGCGGTTCTTTAAGTC  540
            ||||||||||||||||||||||||||||||||||||||||||||||||||||||||||||
Sbjct  484  GGTCCCGAGCGTTGTCCGGATTTATTGGGCGTAAAGCGAGCGCAGGCGGTTCTTTAAGTC  543

Query  541  TGAAGTTAAAGGCAGTGGCTTAACCATTGTACGCTTTGGAAACTGGAGGACTTGAGTGCA  600
            ||||||||||||||||||||||||||||||||||||||||||||||||||||||||||||
Sbjct  544  TGAAGTTAAAGGCAGTGGCTTAACCATTGTACGCTTTGGAAACTGGAGGACTTGAGTGCA  603

Query  601  GAAGGGGAGAGTGGAATTCCATGTGTAGCGGTGAAATGCGTAGATATATGGAGGAACACC  660
            ||||||||||||||||||||||||||||||||||||||||||||||||||||||||||||
Sbjct  604  GAAGGGGAGAGTGGAATTCCATGTGTAGCGGTGAAATGCGTAGATATATGGAGGAACACC  663

Query  661  GGTGGCGAAAGCGGCTCTCTGGTCTGTAACTGACGCTGAGGCTCGAAAGCGTGGGGAGCA  720
            ||||||||||||||||||||||||||||||||||||||||||||||||||||||||||||
Sbjct  664  GGTGGCGAAAGCGGCTCTCTGGTCTGTAACTGACGCTGAGGCTCGAAAGCGTGGGGAGCA  723

Query  721  AACAGGATTAGATACCCTGGTAGTCCACGCCGTAAACGATGAGTGCTAGGTGTTAGGCCC  780
            ||||||||||||||||||||||||||||||||||||||||||||||||||||||||||||
Sbjct  724  AACAGGATTAGATACCCTGGTAGTCCACGCCGTAAACGATGAGTGCTAGGTGTTAGGCCC  783

Query  781  TTTCCGGGGCTTAGTGCCGCAGCTAACGCATTAAGCACTC  820
            ||||||||||||||||||||||||||||||||||||||||
Sbjct  784  TTTCCGGGGCTTAGTGCCGCAGCTAACGCATTAAGCACTC  823
```

Streptococcus agalactiae strain S73 chromosome, complete genome
Sequence ID: **CP030845.1** Length: 2059915 Number of Matches: 7
Range 1: 171 to 990

Score	Expect	Identities	Gaps	Strand	Frame
1509 bits(817)	0.0()	819/820(99%)	0/820(0%)	Plus/Plus	

Features:

```
Query  1    CGCTGATGTTTGGTGTTTACACTAGACTGATGAGTTGCGAACGGGTGAGTAACGCGTAGG  60
            ||||||||||||||||||||||||||||||||||||||||||||||||||||||||||||
Sbjct  171  CGCTGATGTTTGGTGTTTACACTAGACTGATGAGTTGCGAACGGGTGAGTAACGCGTAGG  230

Query  61   TAACCTGCCTCATAGCGGGGGATAACTATTGGAAACGATAGCTAATACCGCATAAGAGTA  120
            ||||||||||||||||||||||||||||||||||||||||||||||||||||||||||| 
Sbjct  231  TAACCTGCCTCATAGCGGGGGATAACTATTGGAAACGATAGCTAATACCGCATAAGAGTG  290

Query  121  ATTAACACATGTTAGTTATTTAAAAGGAGCAATTGCTTCACTGTGAGATGGACCTGCGTT  180
            ||||||||||||||||||||||||||||||||||||||||||||||||||||||||||||
Sbjct  291  ATTAACACATGTTAGTTATTTAAAAGGAGCAATTGCTTCACTGTGAGATGGACCTGCGTT  350

Query  181  GTATTAGCTAGTTGGTGAGGTAAAGGCTCACCAAGGCGACGATACATAGCCGACCTGAGA  240
            ||||||||||||||||||||||||||||||||||||||||||||||||||||||||||||
Sbjct  351  GTATTAGCTAGTTGGTGAGGTAAAGGCTCACCAAGGCGACGATACATAGCCGACCTGAGA  410

Query  241  GGGTGATCGGCCACACTGGGACTGAGACACGGCCCAGACTCCTACGGGAGGCAGCAGTAG  300
            ||||||||||||||||||||||||||||||||||||||||||||||||||||||||||||
Sbjct  411  GGGTGATCGGCCACACTGGGACTGAGACACGGCCCAGACTCCTACGGGAGGCAGCAGTAG  470

Query  301  GGAATCTTCGGCAATGGACGGAAGTCTGACCGAGCAACGCCGCGTGAGTGAAGAAGGTTT  360
            ||||||||||||||||||||||||||||||||||||||||||||||||||||||||||||
Sbjct  471  GGAATCTTCGGCAATGGACGGAAGTCTGACCGAGCAACGCCGCGTGAGTGAAGAAGGTTT  530

Query  361  TCGGATCGTAAAGCTCTGTTGTTAGAGAAGAACGTTGGTAGGAGTGGAAAATCTACCAAG  420
            ||||||||||||||||||||||||||||||||||||||||||||||||||||||||||||
Sbjct  531  TCGGATCGTAAAGCTCTGTTGTTAGAGAAGAACGTTGGTAGGAGTGGAAAATCTACCAAG  590

Query  421  TGACGGTAACTAACCAGAAAGGGACGGCTAACTACGTGCCAGCAGCCGCGGTAATACGTA  480
            ||||||||||||||||||||||||||||||||||||||||||||||||||||||||||||
Sbjct  591  TGACGGTAACTAACCAGAAAGGGACGGCTAACTACGTGCCAGCAGCCGCGGTAATACGTA  650

Query  481  GGTCCCGAGCGTTGTCCGGATTTATTGGGCGTAAAGCGAGCGCAGGCGGTTCTTTAAGTC  540
            ||||||||||||||||||||||||||||||||||||||||||||||||||||||||||||
Sbjct  651  GGTCCCGAGCGTTGTCCGGATTTATTGGGCGTAAAGCGAGCGCAGGCGGTTCTTTAAGTC  710

Query  541  TGAAGTTAAAGGCAGTGGCTTAACCATTGTACGCTTTGGAAACTGGAGGACTTGAGTGCA  600
            ||||||||||||||||||||||||||||||||||||||||||||||||||||||||||||
Sbjct  711  TGAAGTTAAAGGCAGTGGCTTAACCATTGTACGCTTTGGAAACTGGAGGACTTGAGTGCA  770

Query  601  GAAGGGGAGAGTGGAATTCCATGTGTAGCGGTGAAATGCGTAGATATATGGAGGAACACC  660
            ||||||||||||||||||||||||||||||||||||||||||||||||||||||||||||
Sbjct  771  GAAGGGGAGAGTGGAATTCCATGTGTAGCGGTGAAATGCGTAGATATATGGAGGAACACC  830

Query  661  GGTGGCGAAAGCGGCTCTCTGGTCTGTAACTGACGCTGAGGCTCGAAAGCGTGGGGAGCA  720
            ||||||||||||||||||||||||||||||||||||||||||||||||||||||||||||
Sbjct  831  GGTGGCGAAAGCGGCTCTCTGGTCTGTAACTGACGCTGAGGCTCGAAAGCGTGGGGAGCA  890

Query  721  AACAGGATTAGATACCCTGGTAGTCCACGCCGTAAACGATGAGTGCTAGGTGTTAGGCCC  780
            ||||||||||||||||||||||||||||||||||||||||||||||||||||||||||||
Sbjct  891  AACAGGATTAGATACCCTGGTAGTCCACGCCGTAAACGATGAGTGCTAGGTGTTAGGCCC  950

Query  781  TTTCCGGGGCTTAGTGCCGCAGCTAACGCATTAAGCACTC  820
            ||||||||||||||||||||||||||||||||||||||||
Sbjct  951  TTTCCGGGGCTTAGTGCCGCAGCTAACGCATTAAGCACTC  990
```

Range 2: 189274 to 190093

Score	Expect	Identities	Gaps	Strand	Frame
1509 bits(817)	0.0()	819/820(99%)	0/820(0%)	Plus/Plus	

Features:

```
Query  1       CGCTGATGTTTGGTGTTTACACTAGACTGATGAGTTGCGAACGGGTGAGTAACGCGTAGG  60
               ||||||||||||||||||||||||||||||||||||||||||||||||||||||||||||
Sbjct  189274  CGCTGATGTTTGGTGTTTACACTAGACTGATGAGTTGCGAACGGGTGAGTAACGCGTAGG  189333

Query  61      TAACCTGCCTCATAGCGGGGGATAACTATTGGAAACGATAGCTAATACCGCATAAGAGTA  120
               ||||||||||||||||||||||||||||||||||||||||||||||||||||||||||| 
Sbjct  189334  TAACCTGCCTCATAGCGGGGGATAACTATTGGAAACGATAGCTAATACCGCATAAGAGTG  189393

Query  121     ATTAACACATGTTAGTTATTTAAAAGGAGCAATTGCTTCACTGTGAGATGGACCTGCGTT  180
               ||||||||||||||||||||||||||||||||||||||||||||||||||||||||||||
Sbjct  189394  ATTAACACATGTTAGTTATTTAAAAGGAGCAATTGCTTCACTGTGAGATGGACCTGCGTT  189453

Query  181     GTATTAGCTAGTTGGTGAGGTAAAGGCTCACCAAGGCGACGATACATAGCCGACCTGAGA  240
               ||||||||||||||||||||||||||||||||||||||||||||||||||||||||||||
Sbjct  189454  GTATTAGCTAGTTGGTGAGGTAAAGGCTCACCAAGGCGACGATACATAGCCGACCTGAGA  189513

Query  241     GGGTGATCGGCCACACTGGGACTGAGACACGGCCCAGACTCCTACGGGAGGCAGCAGTAG  300
               ||||||||||||||||||||||||||||||||||||||||||||||||||||||||||||
Sbjct  189514  GGGTGATCGGCCACACTGGGACTGAGACACGGCCCAGACTCCTACGGGAGGCAGCAGTAG  189573

Query  301     GGAATCTTCGGCAATGGACGGAAGTCTGACCGAGCAACGCCGCGTGAGTGAAGAAGGTTT  360
               ||||||||||||||||||||||||||||||||||||||||||||||||||||||||||||
Sbjct  189574  GGAATCTTCGGCAATGGACGGAAGTCTGACCGAGCAACGCCGCGTGAGTGAAGAAGGTTT  189633

Query  361     TCGGATCGTAAAGCTCTGTTGTTAGAGAAGAACGTTGGTAGGAGTGGAAAATCTACCAAG  420
               ||||||||||||||||||||||||||||||||||||||||||||||||||||||||||||
Sbjct  189634  TCGGATCGTAAAGCTCTGTTGTTAGAGAAGAACGTTGGTAGGAGTGGAAAATCTACCAAG  189693

Query  421     TGACGGTAACTAACCAGAAAGGGACGGCTAACTACGTGCCAGCAGCCGCGGTAATACGTA  480
               ||||||||||||||||||||||||||||||||||||||||||||||||||||||||||||
Sbjct  189694  TGACGGTAACTAACCAGAAAGGGACGGCTAACTACGTGCCAGCAGCCGCGGTAATACGTA  189753

Query  481     GGTCCCGAGCGTTGTCCGGATTTATTGGGCGTAAAGCGAGCGCAGGCGGTTCTTTAAGTC  540
               ||||||||||||||||||||||||||||||||||||||||||||||||||||||||||||
Sbjct  189754  GGTCCCGAGCGTTGTCCGGATTTATTGGGCGTAAAGCGAGCGCAGGCGGTTCTTTAAGTC  189813

Query  541     TGAAGTTAAAGGCAGTGGCTTAACCATTGTACGCTTTGGAAACTGGAGGACTTGAGTGCA  600
               ||||||||||||||||||||||||||||||||||||||||||||||||||||||||||||
Sbjct  189814  TGAAGTTAAAGGCAGTGGCTTAACCATTGTACGCTTTGGAAACTGGAGGACTTGAGTGCA  189873

Query  601     GAAGGGGAGAGTGGAATTCCATGTGTAGCGGTGAAATGCGTAGATATATGGAGGAACACC  660
               ||||||||||||||||||||||||||||||||||||||||||||||||||||||||||||
Sbjct  189874  GAAGGGGAGAGTGGAATTCCATGTGTAGCGGTGAAATGCGTAGATATATGGAGGAACACC  189933

Query  661     GGTGGCGAAAGCGGCTCTCTGGTCTGTAACTGACGCTGAGGCTCGAAAGCGTGGGGAGCA  720
               ||||||||||||||||||||||||||||||||||||||||||||||||||||||||||||
Sbjct  189934  GGTGGCGAAAGCGGCTCTCTGGTCTGTAACTGACGCTGAGGCTCGAAAGCGTGGGGAGCA  189993

Query  721     AACAGGATTAGATACCCTGGTAGTCCACGCCGTAAACGATGAGTGCTAGGTGTTAGGCCC  780
               ||||||||||||||||||||||||||||||||||||||||||||||||||||||||||||
Sbjct  189994  AACAGGATTAGATACCCTGGTAGTCCACGCCGTAAACGATGAGTGCTAGGTGTTAGGCCC  190053

Query  781     TTTCCGGGGCTTAGTGCCGCAGCTAACGCATTAAGCACTC  820
               ||||||||||||||||||||||||||||||||||||||||
Sbjct  190054  TTTCCGGGGCTTAGTGCCGCAGCTAACGCATTAAGCACTC  190093
```

Range 3: 273861 to 274680

Score	Expect	Identities	Gaps	Strand	Frame
1509 bits(817)	0.0()	819/820(99%)	0/820(0%)	Plus/Plus	

Features:

```
Query  1       CGCTGATGTTTGGTGTTTACACTAGACTGATGAGTTGCGAACGGGTGAGTAACGCGTAGG  60
               ||||||||||||||||||||||||||||||||||||||||||||||||||||||||||||
Sbjct  273861  CGCTGATGTTTGGTGTTTACACTAGACTGATGAGTTGCGAACGGGTGAGTAACGCGTAGG  273920

Query  61      TAACCTGCCTCATAGCGGGGGATAACTATTGGAAACGATAGCTAATACCGCATAAGAGTA  120
               ||||||||||||||||||||||||||||||||||||||||||||||||||||||||||| 
Sbjct  273921  TAACCTGCCTCATAGCGGGGGATAACTATTGGAAACGATAGCTAATACCGCATAAGAGTG  273980

Query  121     ATTAACACATGTTAGTTATTTAAAAGGAGCAATTGCTTCACTGTGAGATGGACCTGCGTT  180
               ||||||||||||||||||||||||||||||||||||||||||||||||||||||||||||
Sbjct  273981  ATTAACACATGTTAGTTATTTAAAAGGAGCAATTGCTTCACTGTGAGATGGACCTGCGTT  274040

Query  181     GTATTAGCTAGTTGGTGAGGTAAAGGCTCACCAAGGCGACGATACATAGCCGACCTGAGA  240
               ||||||||||||||||||||||||||||||||||||||||||||||||||||||||||||
Sbjct  274041  GTATTAGCTAGTTGGTGAGGTAAAGGCTCACCAAGGCGACGATACATAGCCGACCTGAGA  274100
```

```
Query  241     GGGTGATCGGCCACACTGGGACTGAGACACGGCCCAGACTCCTACGGGAGGCAGCAGTAG  300
               ||||||||||||||||||||||||||||||||||||||||||||||||||||||||||||
Sbjct  274101  GGGTGATCGGCCACACTGGGACTGAGACACGGCCCAGACTCCTACGGGAGGCAGCAGTAG  274160
Query  301     GGAATCTTCGGCAATGGACGGAAGTCTGACCGAGCAACGCCGCGTGAGTGAAGAAGGTTT  360
               ||||||||||||||||||||||||||||||||||||||||||||||||||||||||||||
Sbjct  274161  GGAATCTTCGGCAATGGACGGAAGTCTGACCGAGCAACGCCGCGTGAGTGAAGAAGGTTT  274220
Query  361     TCGGATCGTAAAGCTCTGTTGTTAGAGAAGAACGTTGGTAGGAGTGGAAAATCTACCAAG  420
               ||||||||||||||||||||||||||||||||||||||||||||||||||||||||||||
Sbjct  274221  TCGGATCGTAAAGCTCTGTTGTTAGAGAAGAACGTTGGTAGGAGTGGAAAATCTACCAAG  274280
Query  421     TGACGGTAACTAACCAGAAAGGGACGGCTAACTACGTGCCAGCAGCCGCGGTAATACGTA  480
               ||||||||||||||||||||||||||||||||||||||||||||||||||||||||||||
Sbjct  274281  TGACGGTAACTAACCAGAAAGGGACGGCTAACTACGTGCCAGCAGCCGCGGTAATACGTA  274340
Query  481     GGTCCCGAGCGTTGTCCGGATTTATTGGGCGTAAAGCGAGCGCAGGCGGTTCTTTAAGTC  540
               ||||||||||||||||||||||||||||||||||||||||||||||||||||||||||||
Sbjct  274341  GGTCCCGAGCGTTGTCCGGATTTATTGGGCGTAAAGCGAGCGCAGGCGGTTCTTTAAGTC  274400
Query  541     TGAAGTTAAAGGCAGTGGCTTAACCATTGTACGCTTTGGAAACTGGAGGACTTGAGTGCA  600
               ||||||||||||||||||||||||||||||||||||||||||||||||||||||||||||
Sbjct  274401  TGAAGTTAAAGGCAGTGGCTTAACCATTGTACGCTTTGGAAACTGGAGGACTTGAGTGCA  274460
Query  601     GAAGGGGAGAGTGGAATTCCATGTGTAGCGGTGAAATGCGTAGATATATGGAGGAACACC  660
               ||||||||||||||||||||||||||||||||||||||||||||||||||||||||||||
Sbjct  274461  GAAGGGGAGAGTGGAATTCCATGTGTAGCGGTGAAATGCGTAGATATATGGAGGAACACC  274520
Query  661     GGTGGCGAAAGCGGCTCTCTGGTCTGTAACTGACGCTGAGGCTCGAAAGCGTGGGGAGCA  720
               ||||||||||||||||||||||||||||||||||||||||||||||||||||||||||||
Sbjct  274521  GGTGGCGAAAGCGGCTCTCTGGTCTGTAACTGACGCTGAGGCTCGAAAGCGTGGGGAGCA  274580
Query  721     AACAGGATTAGATACCCTGGTAGTCCACGCCGTAAACGATGAGTGCTAGGTGTTAGGCCC  780
               ||||||||||||||||||||||||||||||||||||||||||||||||||||||||||||
Sbjct  274581  AACAGGATTAGATACCCTGGTAGTCCACGCCGTAAACGATGAGTGCTAGGTGTTAGGCCC  274640
Query  781     TTTCCGGGGCTTAGTGCCGCAGCTAACGCATTAAGCACTC  820
               ||||||||||||||||||||||||||||||||||||||||
Sbjct  274641  TTTCCGGGGCTTAGTGCCGCAGCTAACGCATTAAGCACTC  274680
```

Range 4: 371204 to 372023

Score	Expect	Identities	Gaps	Strand	Frame
1509 bits(817)	0.0()	819/820(99%)	0/820(0%)	Plus/Plus	

Features:

```
Query  1       CGCTGATGTTTGGTGTTTACACTAGACTGATGAGTTGCGAACGGGTGAGTAACGCGTAGG  60
               ||||||||||||||||||||||||||||||||||||||||||||||||||||||||||||
Sbjct  371204  CGCTGATGTTTGGTGTTTACACTAGACTGATGAGTTGCGAACGGGTGAGTAACGCGTAGG  371263
Query  61      TAACCTGCCTCATAGCGGGGGATAACTATTGGAAACGATAGCTAATACCGCATAAGAGTA  120
               ||||||||||||||||||||||||||||||||||||||||||||||||||||||||||| 
Sbjct  371264  TAACCTGCCTCATAGCGGGGGATAACTATTGGAAACGATAGCTAATACCGCATAAGAGTG  371323
Query  121     ATTAACACATGTTAGTTATTTAAAAGGAGCAATTGCTTCACTGTGAGATGGACCTGCGTT  180
               ||||||||||||||||||||||||||||||||||||||||||||||||||||||||||||
Sbjct  371324  ATTAACACATGTTAGTTATTTAAAAGGAGCAATTGCTTCACTGTGAGATGGACCTGCGTT  371383
Query  181     GTATTAGCTAGTTGGTGAGGTAAAGGCTCACCAAGGCGACGATACATAGCCGACCTGAGA  240
               ||||||||||||||||||||||||||||||||||||||||||||||||||||||||||||
Sbjct  371384  GTATTAGCTAGTTGGTGAGGTAAAGGCTCACCAAGGCGACGATACATAGCCGACCTGAGA  371443
Query  241     GGGTGATCGGCCACACTGGGACTGAGACACGGCCCAGACTCCTACGGGAGGCAGCAGTAG  300
               ||||||||||||||||||||||||||||||||||||||||||||||||||||||||||||
Sbjct  371444  GGGTGATCGGCCACACTGGGACTGAGACACGGCCCAGACTCCTACGGGAGGCAGCAGTAG  371503
Query  301     GGAATCTTCGGCAATGGACGGAAGTCTGACCGAGCAACGCCGCGTGAGTGAAGAAGGTTT  360
               ||||||||||||||||||||||||||||||||||||||||||||||||||||||||||||
Sbjct  371504  GGAATCTTCGGCAATGGACGGAAGTCTGACCGAGCAACGCCGCGTGAGTGAAGAAGGTTT  371563
Query  361     TCGGATCGTAAAGCTCTGTTGTTAGAGAAGAACGTTGGTAGGAGTGGAAAATCTACCAAG  420
               ||||||||||||||||||||||||||||||||||||||||||||||||||||||||||||
Sbjct  371564  TCGGATCGTAAAGCTCTGTTGTTAGAGAAGAACGTTGGTAGGAGTGGAAAATCTACCAAG  371623
Query  421     TGACGGTAACTAACCAGAAAGGGACGGCTAACTACGTGCCAGCAGCCGCGGTAATACGTA  480
               ||||||||||||||||||||||||||||||||||||||||||||||||||||||||||||
Sbjct  371624  TGACGGTAACTAACCAGAAAGGGACGGCTAACTACGTGCCAGCAGCCGCGGTAATACGTA  371683
Query  481     GGTCCCGAGCGTTGTCCGGATTTATTGGGCGTAAAGCGAGCGCAGGCGGTTCTTTAAGTC  540
               ||||||||||||||||||||||||||||||||||||||||||||||||||||||||||||
Sbjct  371684  GGTCCCGAGCGTTGTCCGGATTTATTGGGCGTAAAGCGAGCGCAGGCGGTTCTTTAAGTC  371743
Query  541     TGAAGTTAAAGGCAGTGGCTTAACCATTGTACGCTTTGGAAACTGGAGGACTTGAGTGCA  600
               ||||||||||||||||||||||||||||||||||||||||||||||||||||||||||||
Sbjct  371744  TGAAGTTAAAGGCAGTGGCTTAACCATTGTACGCTTTGGAAACTGGAGGACTTGAGTGCA  371803
Query  601     GAAGGGGAGAGTGGAATTCCATGTGTAGCGGTGAAATGCGTAGATATATGGAGGAACACC  660
               ||||||||||||||||||||||||||||||||||||||||||||||||||||||||||||
Sbjct  371804  GAAGGGGAGAGTGGAATTCCATGTGTAGCGGTGAAATGCGTAGATATATGGAGGAACACC  371863
Query  661     GGTGGCGAAAGCGGCTCTCTGGTCTGTAACTGACGCTGAGGCTCGAAAGCGTGGGGAGCA  720
               ||||||||||||||||||||||||||||||||||||||||||||||||||||||||||||
Sbjct  371864  GGTGGCGAAAGCGGCTCTCTGGTCTGTAACTGACGCTGAGGCTCGAAAGCGTGGGGAGCA  371923
Query  721     AACAGGATTAGATACCCTGGTAGTCCACGCCGTAAACGATGAGTGCTAGGTGTTAGGCCC  780
               ||||||||||||||||||||||||||||||||||||||||||||||||||||||||||||
Sbjct  371924  AACAGGATTAGATACCCTGGTAGTCCACGCCGTAAACGATGAGTGCTAGGTGTTAGGCCC  371983
Query  781     TTTCCGGGGCTTAGTGCCGCAGCTAACGCATTAAGCACTC  820
               ||||||||||||||||||||||||||||||||||||||||
Sbjct  371984  TTTCCGGGGCTTAGTGCCGCAGCTAACGCATTAAGCACTC  372023
```

Range 5: 440305 to 441124

Score	Expect	Identities	Gaps	Strand	Frame
1509 bits(817)	0.0()	819/820(99%)	0/820(0%)	Plus/Plus	

Features:

```
Query  1       CGCTGATGTTTGGTGTTTACACTAGACTGATGAGTTGCGAACGGGTGAGTAACGCGTAGG  60
               ||||||||||||||||||||||||||||||||||||||||||||||||||||||||||||
Sbjct  440305  CGCTGATGTTTGGTGTTTACACTAGACTGATGAGTTGCGAACGGGTGAGTAACGCGTAGG  440364
Query  61      TAACCTGCCTCATAGCGGGGGATAACTATTGGAAACGATAGCTAATACCGCATAAGAGTA  120
               ||||||||||||||||||||||||||||||||||||||||||||||||||||||||||| 
Sbjct  440365  TAACCTGCCTCATAGCGGGGGATAACTATTGGAAACGATAGCTAATACCGCATAAGAGTG  440424
Query  121     ATTAACACATGTTAGTTATTTAAAAGGAGCAATTGCTTCACTGTGAGATGGACCTGCGTT  180
               ||||||||||||||||||||||||||||||||||||||||||||||||||||||||||||
Sbjct  440425  ATTAACACATGTTAGTTATTTAAAAGGAGCAATTGCTTCACTGTGAGATGGACCTGCGTT  440484
Query  181     GTATTAGCTAGTTGGTGAGGTAAAGGCTCACCAAGGCGACGATACATAGCCGACCTGAGA  240
               ||||||||||||||||||||||||||||||||||||||||||||||||||||||||||||
Sbjct  440485  GTATTAGCTAGTTGGTGAGGTAAAGGCTCACCAAGGCGACGATACATAGCCGACCTGAGA  440544
Query  241     GGGTGATCGGCCACACTGGGACTGAGACACGGCCCAGACTCCTACGGGAGGCAGCAGTAG  300
               ||||||||||||||||||||||||||||||||||||||||||||||||||||||||||||
Sbjct  440545  GGGTGATCGGCCACACTGGGACTGAGACACGGCCCAGACTCCTACGGGAGGCAGCAGTAG  440604
Query  301     GGAATCTTCGGCAATGGACGGAAGTCTGACCGAGCAACGCCGCGTGAGTGAAGAAGGTTT  360
               ||||||||||||||||||||||||||||||||||||||||||||||||||||||||||||
Sbjct  440605  GGAATCTTCGGCAATGGACGGAAGTCTGACCGAGCAACGCCGCGTGAGTGAAGAAGGTTT  440664
Query  361     TCGGATCGTAAAGCTCTGTTGTTAGAGAAGAACGTTGGTAGGAGTGGAAAATCTACCAAG  420
               ||||||||||||||||||||||||||||||||||||||||||||||||||||||||||||
Sbjct  440665  TCGGATCGTAAAGCTCTGTTGTTAGAGAAGAACGTTGGTAGGAGTGGAAAATCTACCAAG  440724
Query  421     TGACGGTAACTAACCAGAAAGGGACGGCTAACTACGTGCCAGCAGCCGCGGTAATACGTA  480
               ||||||||||||||||||||||||||||||||||||||||||||||||||||||||||||
Sbjct  440725  TGACGGTAACTAACCAGAAAGGGACGGCTAACTACGTGCCAGCAGCCGCGGTAATACGTA  440784
Query  481     GGTCCCGAGCGTTGTCCGGATTTATTGGGCGTAAAGCGAGCGCAGGCGGTTCTTTAAGTC  540
               ||||||||||||||||||||||||||||||||||||||||||||||||||||||||||||
Sbjct  440785  GGTCCCGAGCGTTGTCCGGATTTATTGGGCGTAAAGCGAGCGCAGGCGGTTCTTTAAGTC  440844
Query  541     TGAAGTTAAAGGCAGTGGCTTAACCATTGTACGCTTTGGAAACTGGAGGACTTGAGTGCA  600
               ||||||||||||||||||||||||||||||||||||||||||||||||||||||||||||
```

```
Sbjct  440845   TGAAGTTAAAGGCAGTGGCTTAACCATTGTACGCTTTGGAAACTGGAGGACTTGAGTGCA  440904
Query  601      GAAGGGGAGAGTGGAATTCCATGTGTAGCGGTGAAATGCGTAGATATATGGAGGAACACC  660
Sbjct  440905   GAAGGGGAGAGTGGAATTCCATGTGTAGCGGTGAAATGCGTAGATATATGGAGGAACACC  440964
Query  661      GGTGGCGAAAGCGGCTCTCTGGTCTGTAACTGACGCTGAGGCTCGAAAGCGTGGGGAGCA  720
Sbjct  440965   GGTGGCGAAAGCGGCTCTCTGGTCTGTAACTGACGCTGAGGCTCGAAAGCGTGGGGAGCA  441024
Query  721      AACAGGATTAGATACCCTGGTAGTCCACGCCGTAAACGATGAGTGCTAGGTGTTAGGCCC  780
Sbjct  441025   AACAGGATTAGATACCCTGGTAGTCCACGCCGTAAACGATGAGTGCTAGGTGTTAGGCCC  441084
Query  781      TTTCCGGGGCTTAGTGCCGCAGCTAACGCATTAAGCACTC  820
Sbjct  441085   TTTCCGGGGCTTAGTGCCGCAGCTAACGCATTAAGCACTC  441124
```

Range 6: 68894 to 69713

Score	Expect	Identities	Gaps	Strand	Frame
1504 bits(814)	0.0()	818/820(99%)	0/820(0%)	Plus/Plus	

Features:

```
Query  1        CGCTGATGTTTGGTGTTTACACTAGACTGATGAGTTGCGAACGGGTGAGTAACGCGTAGG  60
Sbjct  68894    CGCTGATGTTTGGTGTTTACACTAGACTGATGAGTTGCGAACGGGTGAGTAACGCGTAGG  68953
Query  61       TAACCTGCCTCATAGCGGGGGATAACTATTGGAAACGATAGCTAATACCGCATAAGAGTA  120
Sbjct  68954    TAACCTGCCTCATAGCGGGGGATAACTATTGGAAACGATAGCTAATACCGCATAAGAGTG  69013
Query  121      ATTAACACATGTTAGTTATTTAAAAGGAGCAATTGCTTCACTGTGAGATGGACCTGCGTT  180
Sbjct  69014    ATTAACACATGTTAGTTATTTAAAAGGAGCAATTGCTTCACTGTGAGATGGACCTGCGTT  69073
Query  181      GTATTAGCTAGTTGGTGAGGTAAAGGCTCACCAAGGCGACGATACATAGCCGACCTGAGA  240
Sbjct  69074    GTATTAGCTAGTTGGTGAGGTAAAGGCTCACCAAGGCGACGATACATAGCCGACCTGAGA  69133
Query  241      GGGTGATCGGCCACACTGGGACTGAGACACGGCCCAGACTCCTACGGGAGGCAGCAGTAG  300
Sbjct  69134    GGGTGATCGGCCACACTGGGACTGAGACACGGCCCAGACTCCTACGGGAGGCAGCAGTAG  69193
Query  301      GGAATCTTCGGCAATGGACGGAAGTCTGACCGAGCAACGCCGCGTGAGTGAAGAAGGTTT  360
Sbjct  69194    GGAATCTTCGGCAATGGACGGAAGTCTGACCGAGCAACGCCGCGTGAGTGAAGAAGGTTT  69253
Query  361      TCGGATCGTAAAGCTCTGTTGTTAGAGAAGAACGTTGGTAGGAGTGGAAAATCTACCAAG  420
Sbjct  69254    TCGGATCGTAAAGCTCTGTTGTTAGAGAAGAACGTTGGTAGGAGTGGAAAATCTACCAAG  69313
Query  421      TGACGGTAACTAACCAGAAAGGGACGGCTAACTACGTGCCAGCAGCCGCGGTAATACGTA  480
Sbjct  69314    TGACGGTAACTAACCAGAAAGGGACGGCTAACTACGTGCCAGCAGCCGCGGTAATACGTA  69373
Query  481      GGTCCCGAGCGTTGTCCGGATTTATTGGGCGTAAAGCGAGCGCAGGCGGTTCTTTAAGTC  540
Sbjct  69374    GGTCCCGAGCGTTGTCCGGATTTATTGGGCGTAAAGCGAGCGCAGGCGGTTCTTTAAGTC  69433
Query  541      TGAAGTTAAAGGCAGTGGCTTAACCATTGTACGCTTTGGAAACTGGAGGACTTGAGTGCA  600
Sbjct  69434    TGAAGTTAAAGGCAGTGGCTTAACCATTGTACGCTTTGGAAACTGGAGGACTTGAGTGCA  69493
Query  601      GAAGGGGAGAGTGGAATTCCATGTGTAGCGGTGAAATGCGTAGATATATGGAGGAACACC  660
Sbjct  69494    GAAGGGGAGAGTGGAATTCCATGTGTAGCGGTGAAATGCGTAGATATATGGAGGAACACC  69553
Query  661      GGTGGCGAAAGCGGCTCTCTGGTCTGTAACTGACGCTGAGGCTCGAAAGCGTGGGGAGCA  720
Sbjct  69554    GGTGGCGAAAGCGGCTCTCTGGTCTGTAACTGACGCTGAGGCTCGAAAGCGTGGGGAGCA  69613
Query  721      AACAGGATTAGATACCCTGGTAGTCCACGCCGTAAACGATGAGTGCTAGGTGTTAGGCCC  780
Sbjct  69614    AACAGGATTAGATACCCTGGTAGTCCACGCCGTAAACGATGAGTGCTAGGTGTTAGACCC  69673
Query  781      TTTCCGGGGCTTAGTGCCGCAGCTAACGCATTAAGCACTC  820
Sbjct  69674    TTTCCGGGGCTTAGTGCCGCAGCTAACGCATTAAGCACTC  69713
```

Range 7: 2059845 to 2059915

Score	Expect	Identities	Gaps	Strand	Frame
132 bits(71)	2e-26()	71/71(100%)	0/71(0%)	Plus/Plus	

Features:

```
Query  1        CGCTGATGTTTGGTGTTTACACTAGACTGATGAGTTGCGAACGGGTGAGTAACGCGTAGG  60
Sbjct  2059845  CGCTGATGTTTGGTGTTTACACTAGACTGATGAGTTGCGAACGGGTGAGTAACGCGTAGG  2059904
Query  61       TAACCTGCCTC  71
Sbjct  2059905  TAACCTGCCTC  2059915
```

Streptococcus agalactiae strain B105 chromosome, complete genome
Sequence ID: **CP021773.1** Length: 2273717 Number of Matches: 7
Range 1: 1662942 to 1663761

Score	Expect	Identities	Gaps	Strand	Frame
1509 bits(817)	0.0()	819/820(99%)	0/820(0%)	Plus/Minus	

Features:

```
Query  1        CGCTGATGTTTGGTGTTTACACTAGACTGATGAGTTGCGAACGGGTGAGTAACGCGTAGG  60
Sbjct  1663761  CGCTGAGGTTTGGTGTTTACACTAGACTGATGAGTTGCGAACGGGTGAGTAACGCGTAGG  1663702
Query  61       TAACCTGCCTCATAGCGGGGGATAACTATTGGAAACGATAGCTAATACCGCATAAGAGTA  120
Sbjct  1663701  TAACCTGCCTCATAGCGGGGGATAACTATTGGAAACGATAGCTAATACCGCATAAGAGTA  1663642
Query  121      ATTAACACATGTTAGTTATTTAAAAGGAGCAATTGCTTCACTGTGAGATGGACCTGCGTT  180
Sbjct  1663641  ATTAACACATGTTAGTTATTTAAAAGGAGCAATTGCTTCACTGTGAGATGGACCTGCGTT  1663582
Query  181      GTATTAGCTAGTTGGTGAGGTAAAGGCTCACCAAGGCGACGATACATAGCCGACCTGAGA  240
Sbjct  1663581  GTATTAGCTAGTTGGTGAGGTAAAGGCTCACCAAGGCGACGATACATAGCCGACCTGAGA  1663522
Query  241      GGGTGATCGGCCACACTGGGACTGAGACACGGCCCAGACTCCTACGGGAGGCAGCAGTAG  300
Sbjct  1663521  GGGTGATCGGCCACACTGGGACTGAGACACGGCCCAGACTCCTACGGGAGGCAGCAGTAG  1663462
Query  301      GGAATCTTCGGCAATGGACGGAAGTCTGACCGAGCAACGCCGCGTGAGTGAAGAAGGTTT  360
Sbjct  1663461  GGAATCTTCGGCAATGGACGGAAGTCTGACCGAGCAACGCCGCGTGAGTGAAGAAGGTTT  1663402
Query  361      TCGGATCGTAAAGCTCTGTTGTTAGAGAAGAACGTTGGTAGGAGTGGAAAATCTACCAAG  420
Sbjct  1663401  TCGGATCGTAAAGCTCTGTTGTTAGAGAAGAACGTTGGTAGGAGTGGAAAATCTACCAAG  1663342
Query  421      TGACGGTAACTAACCAGAAAGGGACGGCTAACTACGTGCCAGCAGCCGCGGTAATACGTA  480
Sbjct  1663341  TGACGGTAACTAACCAGAAAGGGACGGCTAACTACGTGCCAGCAGCCGCGGTAATACGTA  1663282
Query  481      GGTCCCGAGCGTTGTCCGGATTTATTGGGCGTAAAGCGAGCGCAGGCGGTTCTTTAAGTC  540
Sbjct  1663281  GGTCCCGAGCGTTGTCCGGATTTATTGGGCGTAAAGCGAGCGCAGGCGGTTCTTTAAGTC  1663222
Query  541      TGAAGTTAAAGGCAGTGGCTTAACCATTGTACGCTTTGGAAACTGGAGGACTTGAGTGCA  600
```

```
                ||||||||||||||||||||||||||||||||||||||||||||||||||||||||||||
Sbjct  1663221  TGAAGTTAAAGGCAGTGGCTTAACCATTGTACGCTTTGGAAACTGGAGGACTTGAGTGCA  1663162
Query  601      GAAGGGGAGAGTGGAATTCCATGTGTAGCGGTGAAATGCGTAGATATATGGAGGAACACC  660
                ||||||||||||||||||||||||||||||||||||||||||||||||||||||||||||
Sbjct  1663161  GAAGGGGAGAGTGGAATTCCATGTGTAGCGGTGAAATGCGTAGATATATGGAGGAACACC  1663102
Query  661      GGTGGCGAAAGCGGCTCTCTGGTCTGTAACTGACGCTGAGGCTCGAAAGCGTGGGGAGCA  720
                ||||||||||||||||||||||||||||||||||||||||||||||||||||||||||||
Sbjct  1663101  GGTGGCGAAAGCGGCTCTCTGGTCTGTAACTGACGCTGAGGCTCGAAAGCGTGGGGAGCA  1663042
Query  721      AACAGGATTAGATACCCTGGTAGTCCACGCCGTAAACGATGAGTGCTAGGTGTTAGGCCC  780
                ||||||||||||||||||||||||||||||||||||||||||||||||||||||||||||
Sbjct  1663041  AACAGGATTAGATACCCTGGTAGTCCACGCCGTAAACGATGAGTGCTAGGTGTTAGGCCC  1662982
Query  781      TTTCCGGGGCTTAGTGCCGCAGCTAACGCATTAAGCACTC  820
                ||||||||||||||||||||||||||||||||||||||||
Sbjct  1662981  TTTCCGGGGCTTAGTGCCGCAGCTAACGCATTAAGCACTC  1662942
```

Range 2: 1732070 to 1732889

Score	Expect	Identities	Gaps	Strand	Frame
1509 bits(817)	0.0()	819/820(99%)	0/820(0%)	Plus/Minus	

Features:

```
Query  1        CGCTGATGTTTGGTGTTTACACTAGACTGATGAGTTGCGAACGGGTGAGTAACGCGTAGG  60
                |||||| |||||||||||||||||||||||||||||||||||||||||||||||||||||
Sbjct  1732889  CGCTGAGGTTTGGTGTTTACACTAGACTGATGAGTTGCGAACGGGTGAGTAACGCGTAGG  1732830
Query  61       TAACCTGCCTCATAGCGGGGGATAACTATTGGAAACGATAGCTAATACCGCATAAGAGTA  120
                ||||||||||||||||||||||||||||||||||||||||||||||||||||||||||||
Sbjct  1732829  TAACCTGCCTCATAGCGGGGGATAACTATTGGAAACGATAGCTAATACCGCATAAGAGTA  1732770
Query  121      ATTAACACATGTTAGTTATTTAAAAGGAGCAATTGCTTCACTGTGAGATGGACCTGCGTT  180
                ||||||||||||||||||||||||||||||||||||||||||||||||||||||||||||
Sbjct  1732769  ATTAACACATGTTAGTTATTTAAAAGGAGCAATTGCTTCACTGTGAGATGGACCTGCGTT  1732710
Query  181      GTATTAGCTAGTTGGTGAGGTAAAGGCTCACCAAGGCGACGATACATAGCCGACCTGAGA  240
                ||||||||||||||||||||||||||||||||||||||||||||||||||||||||||||
Sbjct  1732709  GTATTAGCTAGTTGGTGAGGTAAAGGCTCACCAAGGCGACGATACATAGCCGACCTGAGA  1732650
Query  241      GGGTGATCGGCCACACTGGGACTGAGACACGGCCCAGACTCCTACGGGAGGCAGCAGTAG  300
                ||||||||||||||||||||||||||||||||||||||||||||||||||||||||||||
Sbjct  1732649  GGGTGATCGGCCACACTGGGACTGAGACACGGCCCAGACTCCTACGGGAGGCAGCAGTAG  1732590
Query  301      GGAATCTTCGGCAATGGACGGAAGTCTGACCGAGCAACGCCGCGTGAGTGAAGAAGGTTT  360
                ||||||||||||||||||||||||||||||||||||||||||||||||||||||||||||
Sbjct  1732589  GGAATCTTCGGCAATGGACGGAAGTCTGACCGAGCAACGCCGCGTGAGTGAAGAAGGTTT  1732530
Query  361      TCGGATCGTAAAGCTCTGTTGTTAGAGAAGAACGTTGGTAGGAGTGGAAAATCTACCAAG  420
                ||||||||||||||||||||||||||||||||||||||||||||||||||||||||||||
Sbjct  1732529  TCGGATCGTAAAGCTCTGTTGTTAGAGAAGAACGTTGGTAGGAGTGGAAAATCTACCAAG  1732470
Query  421      TGACGGTAACTAACCAGAAAGGGACGGCTAACTACGTGCCAGCAGCCGCGGTAATACGTA  480
                ||||||||||||||||||||||||||||||||||||||||||||||||||||||||||||
Sbjct  1732469  TGACGGTAACTAACCAGAAAGGGACGGCTAACTACGTGCCAGCAGCCGCGGTAATACGTA  1732410
Query  481      GGTCCCGAGCGTTGTCCGGATTTATTGGGCGTAAAGCGAGCGCAGGCGGTTCTTTAAGTC  540
                ||||||||||||||||||||||||||||||||||||||||||||||||||||||||||||
Sbjct  1732409  GGTCCCGAGCGTTGTCCGGATTTATTGGGCGTAAAGCGAGCGCAGGCGGTTCTTTAAGTC  1732350
Query  541      TGAAGTTAAAGGCAGTGGCTTAACCATTGTACGCTTTGGAAACTGGAGGACTTGAGTGCA  600
                ||||||||||||||||||||||||||||||||||||||||||||||||||||||||||||
Sbjct  1732349  TGAAGTTAAAGGCAGTGGCTTAACCATTGTACGCTTTGGAAACTGGAGGACTTGAGTGCA  1732290
Query  601      GAAGGGGAGAGTGGAATTCCATGTGTAGCGGTGAAATGCGTAGATATATGGAGGAACACC  660
                ||||||||||||||||||||||||||||||||||||||||||||||||||||||||||||
Sbjct  1732289  GAAGGGGAGAGTGGAATTCCATGTGTAGCGGTGAAATGCGTAGATATATGGAGGAACACC  1732230
Query  661      GGTGGCGAAAGCGGCTCTCTGGTCTGTAACTGACGCTGAGGCTCGAAAGCGTGGGGAGCA  720
                ||||||||||||||||||||||||||||||||||||||||||||||||||||||||||||
Sbjct  1732229  GGTGGCGAAAGCGGCTCTCTGGTCTGTAACTGACGCTGAGGCTCGAAAGCGTGGGGAGCA  1732170
Query  721      AACAGGATTAGATACCCTGGTAGTCCACGCCGTAAACGATGAGTGCTAGGTGTTAGGCCC  780
                ||||||||||||||||||||||||||||||||||||||||||||||||||||||||||||
Sbjct  1732169  AACAGGATTAGATACCCTGGTAGTCCACGCCGTAAACGATGAGTGCTAGGTGTTAGGCCC  1732110
Query  781      TTTCCGGGGCTTAGTGCCGCAGCTAACGCATTAAGCACTC  820
                ||||||||||||||||||||||||||||||||||||||||
Sbjct  1732109  TTTCCGGGGCTTAGTGCCGCAGCTAACGCATTAAGCACTC  1732070
```

Range 3: 1827642 to 1828461

Score	Expect	Identities	Gaps	Strand	Frame
1509 bits(817)	0.0()	819/820(99%)	0/820(0%)	Plus/Minus	

Features:

```
Query  1        CGCTGATGTTTGGTGTTTACACTAGACTGATGAGTTGCGAACGGGTGAGTAACGCGTAGG  60
                |||||| |||||||||||||||||||||||||||||||||||||||||||||||||||||
Sbjct  1828461  CGCTGAGGTTTGGTGTTTACACTAGACTGATGAGTTGCGAACGGGTGAGTAACGCGTAGG  1828402
Query  61       TAACCTGCCTCATAGCGGGGGATAACTATTGGAAACGATAGCTAATACCGCATAAGAGTA  120
                ||||||||||||||||||||||||||||||||||||||||||||||||||||||||||||
Sbjct  1828401  TAACCTGCCTCATAGCGGGGGATAACTATTGGAAACGATAGCTAATACCGCATAAGAGTA  1828342
Query  121      ATTAACACATGTTAGTTATTTAAAAGGAGCAATTGCTTCACTGTGAGATGGACCTGCGTT  180
                ||||||||||||||||||||||||||||||||||||||||||||||||||||||||||||
Sbjct  1828341  ATTAACACATGTTAGTTATTTAAAAGGAGCAATTGCTTCACTGTGAGATGGACCTGCGTT  1828282
Query  181      GTATTAGCTAGTTGGTGAGGTAAAGGCTCACCAAGGCGACGATACATAGCCGACCTGAGA  240
                ||||||||||||||||||||||||||||||||||||||||||||||||||||||||||||
Sbjct  1828281  GTATTAGCTAGTTGGTGAGGTAAAGGCTCACCAAGGCGACGATACATAGCCGACCTGAGA  1828222
Query  241      GGGTGATCGGCCACACTGGGACTGAGACACGGCCCAGACTCCTACGGGAGGCAGCAGTAG  300
                ||||||||||||||||||||||||||||||||||||||||||||||||||||||||||||
Sbjct  1828221  GGGTGATCGGCCACACTGGGACTGAGACACGGCCCAGACTCCTACGGGAGGCAGCAGTAG  1828162
Query  301      GGAATCTTCGGCAATGGACGGAAGTCTGACCGAGCAACGCCGCGTGAGTGAAGAAGGTTT  360
                ||||||||||||||||||||||||||||||||||||||||||||||||||||||||||||
Sbjct  1828161  GGAATCTTCGGCAATGGACGGAAGTCTGACCGAGCAACGCCGCGTGAGTGAAGAAGGTTT  1828102
Query  361      TCGGATCGTAAAGCTCTGTTGTTAGAGAAGAACGTTGGTAGGAGTGGAAAATCTACCAAG  420
                ||||||||||||||||||||||||||||||||||||||||||||||||||||||||||||
Sbjct  1828101  TCGGATCGTAAAGCTCTGTTGTTAGAGAAGAACGTTGGTAGGAGTGGAAAATCTACCAAG  1828042
Query  421      TGACGGTAACTAACCAGAAAGGGACGGCTAACTACGTGCCAGCAGCCGCGGTAATACGTA  480
                ||||||||||||||||||||||||||||||||||||||||||||||||||||||||||||
Sbjct  1828041  TGACGGTAACTAACCAGAAAGGGACGGCTAACTACGTGCCAGCAGCCGCGGTAATACGTA  1827982
Query  481      GGTCCCGAGCGTTGTCCGGATTTATTGGGCGTAAAGCGAGCGCAGGCGGTTCTTTAAGTC  540
                ||||||||||||||||||||||||||||||||||||||||||||||||||||||||||||
Sbjct  1827981  GGTCCCGAGCGTTGTCCGGATTTATTGGGCGTAAAGCGAGCGCAGGCGGTTCTTTAAGTC  1827922
Query  541      TGAAGTTAAAGGCAGTGGCTTAACCATTGTACGCTTTGGAAACTGGAGGACTTGAGTGCA  600
                ||||||||||||||||||||||||||||||||||||||||||||||||||||||||||||
Sbjct  1827921  TGAAGTTAAAGGCAGTGGCTTAACCATTGTACGCTTTGGAAACTGGAGGACTTGAGTGCA  1827862
Query  601      GAAGGGGAGAGTGGAATTCCATGTGTAGCGGTGAAATGCGTAGATATATGGAGGAACACC  660
                ||||||||||||||||||||||||||||||||||||||||||||||||||||||||||||
Sbjct  1827861  GAAGGGGAGAGTGGAATTCCATGTGTAGCGGTGAAATGCGTAGATATATGGAGGAACACC  1827802
Query  661      GGTGGCGAAAGCGGCTCTCTGGTCTGTAACTGACGCTGAGGCTCGAAAGCGTGGGGAGCA  720
                ||||||||||||||||||||||||||||||||||||||||||||||||||||||||||||
Sbjct  1827801  GGTGGCGAAAGCGGCTCTCTGGTCTGTAACTGACGCTGAGGCTCGAAAGCGTGGGGAGCA  1827742
Query  721      AACAGGATTAGATACCCTGGTAGTCCACGCCGTAAACGATGAGTGCTAGGTGTTAGGCCC  780
                ||||||||||||||||||||||||||||||||||||||||||||||||||||||||||||
Sbjct  1827741  AACAGGATTAGATACCCTGGTAGTCCACGCCGTAAACGATGAGTGCTAGGTGTTAGGCCC  1827682
Query  781      TTTCCGGGGCTTAGTGCCGCAGCTAACGCATTAAGCACTC  820
                ||||||||||||||||||||||||||||||||||||||||
Sbjct  1827681  TTTCCGGGGCTTAGTGCCGCAGCTAACGCATTAAGCACTC  1827642
```

Range 4: 1913645 to 1914464

Score	Expect	Identities	Gaps	Strand	Frame
1509 bits(817)	0.0()	819/820(99%)	0/820(0%)	Plus/Minus	

Features:

```
Query  1        CGCTGATGTTTGGTGTTTACACTAGACTGATGAGTTGCGAACGGGTGAGTAACGCGTAGG  60
                |||||| |||||||||||||||||||||||||||||||||||||||||||||||||||||
Sbjct  1914464  CGCTGAGGTTTGGTGTTTACACTAGACTGATGAGTTGCGAACGGGTGAGTAACGCGTAGG  1914405

Query  61       TAACCTGCCTCATAGCGGGGGATAACTATTGGAAACGATAGCTAATACCGCATAAGAGTA  120
                ||||||||||||||||||||||||||||||||||||||||||||||||||||||||||||
Sbjct  1914404  TAACCTGCCTCATAGCGGGGGATAACTATTGGAAACGATAGCTAATACCGCATAAGAGTA  1914345

Query  121      ATTAACACATGTTAGTTATTTAAAAGGAGCAATTGCTTCACTGTGAGATGGACCTGCGTT  180
                ||||||||||||||||||||||||||||||||||||||||||||||||||||||||||||
Sbjct  1914344  ATTAACACATGTTAGTTATTTAAAAGGAGCAATTGCTTCACTGTGAGATGGACCTGCGTT  1914285

Query  181      GTATTAGCTAGTTGGTGAGGTAAAGGCTCACCAAGGCGACGATACATAGCCGACCTGAGA  240
                ||||||||||||||||||||||||||||||||||||||||||||||||||||||||||||
Sbjct  1914284  GTATTAGCTAGTTGGTGAGGTAAAGGCTCACCAAGGCGACGATACATAGCCGACCTGAGA  1914225

Query  241      GGGTGATCGGCCACACTGGGACTGAGACACGGCCCAGACTCCTACGGGAGGCAGCAGTAG  300
                ||||||||||||||||||||||||||||||||||||||||||||||||||||||||||||
Sbjct  1914224  GGGTGATCGGCCACACTGGGACTGAGACACGGCCCAGACTCCTACGGGAGGCAGCAGTAG  1914165

Query  301      GGAATCTTCGGCAATGGACGGAAGTCTGACCGAGCAACGCCGCGTGAGTGAAGAAGGTTT  360
                ||||||||||||||||||||||||||||||||||||||||||||||||||||||||||||
Sbjct  1914164  GGAATCTTCGGCAATGGACGGAAGTCTGACCGAGCAACGCCGCGTGAGTGAAGAAGGTTT  1914105

Query  361      TCGGATCGTAAAGCTCTGTTGTTAGAGAAGAACGTTGGTAGGAGTGGAAAATCTACCAAG  420
                ||||||||||||||||||||||||||||||||||||||||||||||||||||||||||||
Sbjct  1914104  TCGGATCGTAAAGCTCTGTTGTTAGAGAAGAACGTTGGTAGGAGTGGAAAATCTACCAAG  1914045

Query  421      TGACGGTAACTAACCAGAAAGGGACGGCTAACTACGTGCCAGCAGCCGCGGTAATACGTA  480
                ||||||||||||||||||||||||||||||||||||||||||||||||||||||||||||
Sbjct  1914044  TGACGGTAACTAACCAGAAAGGGACGGCTAACTACGTGCCAGCAGCCGCGGTAATACGTA  1913985

Query  481      GGTCCCGAGCGTTGTCCGGATTTATTGGGCGTAAAGCGAGCGCAGGCGGTTCTTTAAGTC  540
                ||||||||||||||||||||||||||||||||||||||||||||||||||||||||||||
Sbjct  1913984  GGTCCCGAGCGTTGTCCGGATTTATTGGGCGTAAAGCGAGCGCAGGCGGTTCTTTAAGTC  1913925

Query  541      TGAAGTTAAAGGCAGTGGCTTAACCATTGTACGCTTTGGAAACTGGAGGACTTGAGTGCA  600
                ||||||||||||||||||||||||||||||||||||||||||||||||||||||||||||
Sbjct  1913924  TGAAGTTAAAGGCAGTGGCTTAACCATTGTACGCTTTGGAAACTGGAGGACTTGAGTGCA  1913865

Query  601      GAAGGGGAGAGTGGAATTCCATGTGTAGCGGTGAAATGCGTAGATATATGGAGGAACACC  660
                ||||||||||||||||||||||||||||||||||||||||||||||||||||||||||||
Sbjct  1913864  GAAGGGGAGAGTGGAATTCCATGTGTAGCGGTGAAATGCGTAGATATATGGAGGAACACC  1913805

Query  661      GGTGGCGAAAGCGGCTCTCTGGTCTGTAACTGACGCTGAGGCTCGAAAGCGTGGGGAGCA  720
                ||||||||||||||||||||||||||||||||||||||||||||||||||||||||||||
Sbjct  1913804  GGTGGCGAAAGCGGCTCTCTGGTCTGTAACTGACGCTGAGGCTCGAAAGCGTGGGGAGCA  1913745

Query  721      AACAGGATTAGATACCCTGGTAGTCCACGCCGTAAACGATGAGTGCTAGGTGTTAGGCCC  780
                ||||||||||||||||||||||||||||||||||||||||||||||||||||||||||||
Sbjct  1913744  AACAGGATTAGATACCCTGGTAGTCCACGCCGTAAACGATGAGTGCTAGGTGTTAGGCCC  1913685

Query  781      TTTCCGGGGCTTAGTGCCGCAGCTAACGCATTAAGCACTC  820
                ||||||||||||||||||||||||||||||||||||||||
Sbjct  1913684  TTTCCGGGGCTTAGTGCCGCAGCTAACGCATTAAGCACTC  1913645
```

Range 5: 1989643 to 1990462

Score	Expect	Identities	Gaps	Strand	Frame
1509 bits(817)	0.0()	819/820(99%)	0/820(0%)	Plus/Minus	

Features:

```
Query  1        CGCTGATGTTTGGTGTTTACACTAGACTGATGAGTTGCGAACGGGTGAGTAACGCGTAGG  60
                |||||| |||||||||||||||||||||||||||||||||||||||||||||||||||||
Sbjct  1990462  CGCTGAGGTTTGGTGTTTACACTAGACTGATGAGTTGCGAACGGGTGAGTAACGCGTAGG  1990403

Query  61       TAACCTGCCTCATAGCGGGGGATAACTATTGGAAACGATAGCTAATACCGCATAAGAGTA  120
                ||||||||||||||||||||||||||||||||||||||||||||||||||||||||||||
Sbjct  1990402  TAACCTGCCTCATAGCGGGGGATAACTATTGGAAACGATAGCTAATACCGCATAAGAGTA  1990343

Query  121      ATTAACACATGTTAGTTATTTAAAAGGAGCAATTGCTTCACTGTGAGATGGACCTGCGTT  180
                ||||||||||||||||||||||||||||||||||||||||||||||||||||||||||||
Sbjct  1990342  ATTAACACATGTTAGTTATTTAAAAGGAGCAATTGCTTCACTGTGAGATGGACCTGCGTT  1990283

Query  181      GTATTAGCTAGTTGGTGAGGTAAAGGCTCACCAAGGCGACGATACATAGCCGACCTGAGA  240
                ||||||||||||||||||||||||||||||||||||||||||||||||||||||||||||
Sbjct  1990282  GTATTAGCTAGTTGGTGAGGTAAAGGCTCACCAAGGCGACGATACATAGCCGACCTGAGA  1990223

Query  241      GGGTGATCGGCCACACTGGGACTGAGACACGGCCCAGACTCCTACGGGAGGCAGCAGTAG  300
                ||||||||||||||||||||||||||||||||||||||||||||||||||||||||||||
Sbjct  1990222  GGGTGATCGGCCACACTGGGACTGAGACACGGCCCAGACTCCTACGGGAGGCAGCAGTAG  1990163

Query  301      GGAATCTTCGGCAATGGACGGAAGTCTGACCGAGCAACGCCGCGTGAGTGAAGAAGGTTT  360
                ||||||||||||||||||||||||||||||||||||||||||||||||||||||||||||
Sbjct  1990162  GGAATCTTCGGCAATGGACGGAAGTCTGACCGAGCAACGCCGCGTGAGTGAAGAAGGTTT  1990103

Query  361      TCGGATCGTAAAGCTCTGTTGTTAGAGAAGAACGTTGGTAGGAGTGGAAAATCTACCAAG  420
                ||||||||||||||||||||||||||||||||||||||||||||||||||||||||||||
Sbjct  1990102  TCGGATCGTAAAGCTCTGTTGTTAGAGAAGAACGTTGGTAGGAGTGGAAAATCTACCAAG  1990043

Query  421      TGACGGTAACTAACCAGAAAGGGACGGCTAACTACGTGCCAGCAGCCGCGGTAATACGTA  480
                ||||||||||||||||||||||||||||||||||||||||||||||||||||||||||||
Sbjct  1990042  TGACGGTAACTAACCAGAAAGGGACGGCTAACTACGTGCCAGCAGCCGCGGTAATACGTA  1989983

Query  481      GGTCCCGAGCGTTGTCCGGATTTATTGGGCGTAAAGCGAGCGCAGGCGGTTCTTTAAGTC  540
                ||||||||||||||||||||||||||||||||||||||||||||||||||||||||||||
Sbjct  1989982  GGTCCCGAGCGTTGTCCGGATTTATTGGGCGTAAAGCGAGCGCAGGCGGTTCTTTAAGTC  1989923

Query  541      TGAAGTTAAAGGCAGTGGCTTAACCATTGTACGCTTTGGAAACTGGAGGACTTGAGTGCA  600
                ||||||||||||||||||||||||||||||||||||||||||||||||||||||||||||
Sbjct  1989922  TGAAGTTAAAGGCAGTGGCTTAACCATTGTACGCTTTGGAAACTGGAGGACTTGAGTGCA  1989863

Query  601      GAAGGGGAGAGTGGAATTCCATGTGTAGCGGTGAAATGCGTAGATATATGGAGGAACACC  660
                ||||||||||||||||||||||||||||||||||||||||||||||||||||||||||||
Sbjct  1989862  GAAGGGGAGAGTGGAATTCCATGTGTAGCGGTGAAATGCGTAGATATATGGAGGAACACC  1989803

Query  661      GGTGGCGAAAGCGGCTCTCTGGTCTGTAACTGACGCTGAGGCTCGAAAGCGTGGGGAGCA  720
                ||||||||||||||||||||||||||||||||||||||||||||||||||||||||||||
Sbjct  1989802  GGTGGCGAAAGCGGCTCTCTGGTCTGTAACTGACGCTGAGGCTCGAAAGCGTGGGGAGCA  1989743

Query  721      AACAGGATTAGATACCCTGGTAGTCCACGCCGTAAACGATGAGTGCTAGGTGTTAGGCCC  780
                ||||||||||||||||||||||||||||||||||||||||||||||||||||||||||||
Sbjct  1989742  AACAGGATTAGATACCCTGGTAGTCCACGCCGTAAACGATGAGTGCTAGGTGTTAGGCCC  1989683

Query  781      TTTCCGGGGCTTAGTGCCGCAGCTAACGCATTAAGCACTC  820
                ||||||||||||||||||||||||||||||||||||||||
Sbjct  1989682  TTTCCGGGGCTTAGTGCCGCAGCTAACGCATTAAGCACTC  1989643
```

Range 6: 2059146 to 2059965

Score	Expect	Identities	Gaps	Strand	Frame
1509 bits(817)	0.0()	819/820(99%)	0/820(0%)	Plus/Minus	

Features:

```
Query  1        CGCTGATGTTTGGTGTTTACACTAGACTGATGAGTTGCGAACGGGTGAGTAACGCGTAGG  60
                |||||| |||||||||||||||||||||||||||||||||||||||||||||||||||||
Sbjct  2059965  CGCTGAGGTTTGGTGTTTACACTAGACTGATGAGTTGCGAACGGGTGAGTAACGCGTAGG  2059906

Query  61       TAACCTGCCTCATAGCGGGGGATAACTATTGGAAACGATAGCTAATACCGCATAAGAGTA  120
                ||||||||||||||||||||||||||||||||||||||||||||||||||||||||||||
Sbjct  2059905  TAACCTGCCTCATAGCGGGGGATAACTATTGGAAACGATAGCTAATACCGCATAAGAGTA  2059846

Query  121      ATTAACACATGTTAGTTATTTAAAAGGAGCAATTGCTTCACTGTGAGATGGACCTGCGTT  180
                ||||||||||||||||||||||||||||||||||||||||||||||||||||||||||||
Sbjct  2059845  ATTAACACATGTTAGTTATTTAAAAGGAGCAATTGCTTCACTGTGAGATGGACCTGCGTT  2059786

Query  181      GTATTAGCTAGTTGGTGAGGTAAAGGCTCACCAAGGCGACGATACATAGCCGACCTGAGA  240
                ||||||||||||||||||||||||||||||||||||||||||||||||||||||||||||
Sbjct  2059785  GTATTAGCTAGTTGGTGAGGTAAAGGCTCACCAAGGCGACGATACATAGCCGACCTGAGA  2059726
```

```
Query  241      GGGTGATCGGCCACACTGGGACTGAGACACGGCCCAGACTCCTACGGGAGGCAGCAGTAG  300
                ||||||||||||||||||||||||||||||||||||||||||||||||||||||||||||
Sbjct  2059725  GGGTGATCGGCCACACTGGGACTGAGACACGGCCCAGACTCCTACGGGAGGCAGCAGTAG  2059666

Query  301      GGAATCTTCGGCAATGGACGGAAGTCTGACCGAGCAACGCCGCGTGAGTGAAGAAGGTTT  360
                ||||||||||||||||||||||||||||||||||||||||||||||||||||||||||||
Sbjct  2059665  GGAATCTTCGGCAATGGACGGAAGTCTGACCGAGCAACGCCGCGTGAGTGAAGAAGGTTT  2059606

Query  361      TCGGATCGTAAAGCTCTGTTGTTAGAGAAGAACGTTGGTAGGAGTGGAAAATCTACCAAG  420
                ||||||||||||||||||||||||||||||||||||||||||||||||||||||||||||
Sbjct  2059605  TCGGATCGTAAAGCTCTGTTGTTAGAGAAGAACGTTGGTAGGAGTGGAAAATCTACCAAG  2059546

Query  421      TGACGGTAACTAACCAGAAAGGGACGGCTAACTACGTGCCAGCAGCCGCGGTAATACGTA  480
                ||||||||||||||||||||||||||||||||||||||||||||||||||||||||||||
Sbjct  2059545  TGACGGTAACTAACCAGAAAGGGACGGCTAACTACGTGCCAGCAGCCGCGGTAATACGTA  2059486

Query  481      GGTCCCGAGCGTTGTCCGGATTTATTGGGCGTAAAGCGAGCGCAGGCGGTTCTTTAAGTC  540
                ||||||||||||||||||||||||||||||||||||||||||||||||||||||||||||
Sbjct  2059485  GGTCCCGAGCGTTGTCCGGATTTATTGGGCGTAAAGCGAGCGCAGGCGGTTCTTTAAGTC  2059426

Query  541      TGAAGTTAAAGGCAGTGGCTTAACCATTGTACGCTTTGGAAACTGGAGGACTTGAGTGCA  600
                ||||||||||||||||||||||||||||||||||||||||||||||||||||||||||||
Sbjct  2059425  TGAAGTTAAAGGCAGTGGCTTAACCATTGTACGCTTTGGAAACTGGAGGACTTGAGTGCA  2059366

Query  601      GAAGGGGAGAGTGGAATTCCATGTGTAGCGGTGAAATGCGTAGATATATGGAGGAACACC  660
                ||||||||||||||||||||||||||||||||||||||||||||||||||||||||||||
Sbjct  2059365  GAAGGGGAGAGTGGAATTCCATGTGTAGCGGTGAAATGCGTAGATATATGGAGGAACACC  2059306

Query  661      GGTGGCGAAAGCGGCTCTCTGGTCTGTAACTGACGCTGAGGCTCGAAAGCGTGGGGAGCA  720
                ||||||||||||||||||||||||||||||||||||||||||||||||||||||||||||
Sbjct  2059305  GGTGGCGAAAGCGGCTCTCTGGTCTGTAACTGACGCTGAGGCTCGAAAGCGTGGGGAGCA  2059246

Query  721      AACAGGATTAGATACCCTGGTAGTCCACGCCGTAAACGATGAGTGCTAGGTGTTAGGCCC  780
                ||||||||||||||||||||||||||||||||||||||||||||||||||||||||||||
Sbjct  2059245  AACAGGATTAGATACCCTGGTAGTCCACGCCGTAAACGATGAGTGCTAGGTGTTAGGCCC  2059186

Query  781      TTTCCGGGGCTTAGTGCCGCAGCTAACGCATTAAGCACTC  820
                ||||||||||||||||||||||||||||||||||||||||
Sbjct  2059185  TTTCCGGGGCTTAGTGCCGCAGCTAACGCATTAAGCACTC  2059146
```

Range 7: 2064977 to 2065796

Score	Expect	Identities	Gaps	Strand	Frame
1509 bits(817)	0.0()	819/820(99%)	0/820(0%)	Plus/Minus	

Features:

```
Query  1        CGCTGATGTTTGGTGTTTACACTAGACTGATGAGTTGCGAACGGGTGAGTAACGCGTAGG  60
                |||||| |||||||||||||||||||||||||||||||||||||||||||||||||||||
Sbjct  2065796  CGCTGAGGTTTGGTGTTTACACTAGACTGATGAGTTGCGAACGGGTGAGTAACGCGTAGG  2065737

Query  61       TAACCTGCCTCATAGCGGGGGATAACTATTGGAAACGATAGCTAATACCGCATAAGAGTA  120
                ||||||||||||||||||||||||||||||||||||||||||||||||||||||||||||
Sbjct  2065736  TAACCTGCCTCATAGCGGGGGATAACTATTGGAAACGATAGCTAATACCGCATAAGAGTA  2065677

Query  121      ATTAACACATGTTAGTTATTTAAAAGGAGCAATTGCTTCACTGTGAGATGGACCTGCGTT  180
                ||||||||||||||||||||||||||||||||||||||||||||||||||||||||||||
Sbjct  2065676  ATTAACACATGTTAGTTATTTAAAAGGAGCAATTGCTTCACTGTGAGATGGACCTGCGTT  2065617

Query  181      GTATTAGCTAGTTGGTGAGGTAAAGGCTCACCAAGGCGACGATACATAGCCGACCTGAGA  240
                ||||||||||||||||||||||||||||||||||||||||||||||||||||||||||||
Sbjct  2065616  GTATTAGCTAGTTGGTGAGGTAAAGGCTCACCAAGGCGACGATACATAGCCGACCTGAGA  2065557

Query  241      GGGTGATCGGCCACACTGGGACTGAGACACGGCCCAGACTCCTACGGGAGGCAGCAGTAG  300
                ||||||||||||||||||||||||||||||||||||||||||||||||||||||||||||
Sbjct  2065556  GGGTGATCGGCCACACTGGGACTGAGACACGGCCCAGACTCCTACGGGAGGCAGCAGTAG  2065497

Query  301      GGAATCTTCGGCAATGGACGGAAGTCTGACCGAGCAACGCCGCGTGAGTGAAGAAGGTTT  360
                ||||||||||||||||||||||||||||||||||||||||||||||||||||||||||||
Sbjct  2065496  GGAATCTTCGGCAATGGACGGAAGTCTGACCGAGCAACGCCGCGTGAGTGAAGAAGGTTT  2065437

Query  361      TCGGATCGTAAAGCTCTGTTGTTAGAGAAGAACGTTGGTAGGAGTGGAAAATCTACCAAG  420
                ||||||||||||||||||||||||||||||||||||||||||||||||||||||||||||
Sbjct  2065436  TCGGATCGTAAAGCTCTGTTGTTAGAGAAGAACGTTGGTAGGAGTGGAAAATCTACCAAG  2065377

Query  421      TGACGGTAACTAACCAGAAAGGGACGGCTAACTACGTGCCAGCAGCCGCGGTAATACGTA  480
                ||||||||||||||||||||||||||||||||||||||||||||||||||||||||||||
Sbjct  2065376  TGACGGTAACTAACCAGAAAGGGACGGCTAACTACGTGCCAGCAGCCGCGGTAATACGTA  2065317

Query  481      GGTCCCGAGCGTTGTCCGGATTTATTGGGCGTAAAGCGAGCGCAGGCGGTTCTTTAAGTC  540
                ||||||||||||||||||||||||||||||||||||||||||||||||||||||||||||
Sbjct  2065316  GGTCCCGAGCGTTGTCCGGATTTATTGGGCGTAAAGCGAGCGCAGGCGGTTCTTTAAGTC  2065257

Query  541      TGAAGTTAAAGGCAGTGGCTTAACCATTGTACGCTTTGGAAACTGGAGGACTTGAGTGCA  600
                ||||||||||||||||||||||||||||||||||||||||||||||||||||||||||||
Sbjct  2065256  TGAAGTTAAAGGCAGTGGCTTAACCATTGTACGCTTTGGAAACTGGAGGACTTGAGTGCA  2065197

Query  601      GAAGGGGAGAGTGGAATTCCATGTGTAGCGGTGAAATGCGTAGATATATGGAGGAACACC  660
                ||||||||||||||||||||||||||||||||||||||||||||||||||||||||||||
Sbjct  2065196  GAAGGGGAGAGTGGAATTCCATGTGTAGCGGTGAAATGCGTAGATATATGGAGGAACACC  2065137

Query  661      GGTGGCGAAAGCGGCTCTCTGGTCTGTAACTGACGCTGAGGCTCGAAAGCGTGGGGAGCA  720
                ||||||||||||||||||||||||||||||||||||||||||||||||||||||||||||
Sbjct  2065136  GGTGGCGAAAGCGGCTCTCTGGTCTGTAACTGACGCTGAGGCTCGAAAGCGTGGGGAGCA  2065077

Query  721      AACAGGATTAGATACCCTGGTAGTCCACGCCGTAAACGATGAGTGCTAGGTGTTAGGCCC  780
                ||||||||||||||||||||||||||||||||||||||||||||||||||||||||||||
Sbjct  2065076  AACAGGATTAGATACCCTGGTAGTCCACGCCGTAAACGATGAGTGCTAGGTGTTAGGCCC  2065017

Query  781      TTTCCGGGGCTTAGTGCCGCAGCTAACGCATTAAGCACTC  820
                ||||||||||||||||||||||||||||||||||||||||
Sbjct  2065016  TTTCCGGGGCTTAGTGCCGCAGCTAACGCATTAAGCACTC  2064977
```

Streptococcus agalactiae strain B508 chromosome, complete genome
Sequence ID: **CP021770.1** Length: 2199578 Number of Matches: 7
Range 1: 16979 to 17798

Score	Expect	Identities	Gaps	Strand	Frame
1509 bits(817)	0.0()	819/820(99%)	0/820(0%)	Plus/Plus	

Features:

```
Query  1      CGCTGATGTTTGGTGTTTACACTAGACTGATGAGTTGCGAACGGGTGAGTAACGCGTAGG  60
              |||||| |||||||||||||||||||||||||||||||||||||||||||||||||||||
Sbjct  16979  CGCTGAGGTTTGGTGTTTACACTAGACTGATGAGTTGCGAACGGGTGAGTAACGCGTAGG  17038

Query  61     TAACCTGCCTCATAGCGGGGGATAACTATTGGAAACGATAGCTAATACCGCATAAGAGTA  120
              ||||||||||||||||||||||||||||||||||||||||||||||||||||||||||||
Sbjct  17039  TAACCTGCCTCATAGCGGGGGATAACTATTGGAAACGATAGCTAATACCGCATAAGAGTA  17098

Query  121    ATTAACACATGTTAGTTATTTAAAAGGAGCAATTGCTTCACTGTGAGATGGACCTGCGTT  180
              ||||||||||||||||||||||||||||||||||||||||||||||||||||||||||||
Sbjct  17099  ATTAACACATGTTAGTTATTTAAAAGGAGCAATTGCTTCACTGTGAGATGGACCTGCGTT  17158

Query  181    GTATTAGCTAGTTGGTGAGGTAAAGGCTCACCAAGGCGACGATACATAGCCGACCTGAGA  240
              ||||||||||||||||||||||||||||||||||||||||||||||||||||||||||||
Sbjct  17159  GTATTAGCTAGTTGGTGAGGTAAAGGCTCACCAAGGCGACGATACATAGCCGACCTGAGA  17218

Query  241    GGGTGATCGGCCACACTGGGACTGAGACACGGCCCAGACTCCTACGGGAGGCAGCAGTAG  300
              ||||||||||||||||||||||||||||||||||||||||||||||||||||||||||||
Sbjct  17219  GGGTGATCGGCCACACTGGGACTGAGACACGGCCCAGACTCCTACGGGAGGCAGCAGTAG  17278

Query  301    GGAATCTTCGGCAATGGACGGAAGTCTGACCGAGCAACGCCGCGTGAGTGAAGAAGGTTT  360
              ||||||||||||||||||||||||||||||||||||||||||||||||||||||||||||
Sbjct  17279  GGAATCTTCGGCAATGGACGGAAGTCTGACCGAGCAACGCCGCGTGAGTGAAGAAGGTTT  17338

Query  361    TCGGATCGTAAAGCTCTGTTGTTAGAGAAGAACGTTGGTAGGAGTGGAAAATCTACCAAG  420
              ||||||||||||||||||||||||||||||||||||||||||||||||||||||||||||
Sbjct  17339  TCGGATCGTAAAGCTCTGTTGTTAGAGAAGAACGTTGGTAGGAGTGGAAAATCTACCAAG  17398

Query  421    TGACGGTAACTAACCAGAAAGGGACGGCTAACTACGTGCCAGCAGCCGCGGTAATACGTA  480
              ||||||||||||||||||||||||||||||||||||||||||||||||||||||||||||
Sbjct  17399  TGACGGTAACTAACCAGAAAGGGACGGCTAACTACGTGCCAGCAGCCGCGGTAATACGTA  17458

Query  481    GGTCCCGAGCGTTGTCCGGATTTATTGGGCGTAAAGCGAGCGCAGGCGGTTCTTTAAGTC  540
              ||||||||||||||||||||||||||||||||||||||||||||||||||||||||||||
```

```
Sbjct  17459   GGTCCCGAGCGTTGTCCGGATTTATTGGGCGTAAAGCGAGCGCAGGCGGTTCTTTAAGTC  17518

Query  541     TGAAGTTAAAGGCAGTGGCTTAACCATTGTACGCTTTGGAAACTGGAGGACTTGAGTGCA  600
               ||||||||||||||||||||||||||||||||||||||||||||||||||||||||||||
Sbjct  17519   TGAAGTTAAAGGCAGTGGCTTAACCATTGTACGCTTTGGAAACTGGAGGACTTGAGTGCA  17578

Query  601     GAAGGGGAGAGTGGAATTCCATGTGTAGCGGTGAAATGCGTAGATATATGGAGGAACACC  660
               ||||||||||||||||||||||||||||||||||||||||||||||||||||||||||||
Sbjct  17579   GAAGGGGAGAGTGGAATTCCATGTGTAGCGGTGAAATGCGTAGATATATGGAGGAACACC  17638

Query  661     GGTGGCGAAAGCGGCTCTCTGGTCTGTAACTGACGCTGAGGCTCGAAAGCGTGGGGAGCA  720
               ||||||||||||||||||||||||||||||||||||||||||||||||||||||||||||
Sbjct  17639   GGTGGCGAAAGCGGCTCTCTGGTCTGTAACTGACGCTGAGGCTCGAAAGCGTGGGGAGCA  17698

Query  721     AACAGGATTAGATACCCTGGTAGTCCACGCCGTAAACGATGAGTGCTAGGTGTTAGGCCC  780
               ||||||||||||||||||||||||||||||||||||||||||||||||||||||||||||
Sbjct  17699   AACAGGATTAGATACCCTGGTAGTCCACGCCGTAAACGATGAGTGCTAGGTGTTAGGCCC  17758

Query  781     TTTCCGGGGCTTAGTGCCGCAGCTAACGCATTAAGCACTC  820
               ||||||||||||||||||||||||||||||||||||||||
Sbjct  17759   TTTCCGGGGCTTAGTGCCGCAGCTAACGCATTAAGCACTC  17798
```

Range 2: 115180 to 115999

Score	Expect	Identities	Gaps	Strand	Frame
1509 bits(817)	0.0()	819/820(99%)	0/820(0%)	Plus/Plus	

Features:

```
Query  1       CGCTGATGTTTGGTGTTTACACTAGACTGATGAGTTGCGAACGGGTGAGTAACGCGTAGG  60
               |||||| |||||||||||||||||||||||||||||||||||||||||||||||||||||
Sbjct  115180  CGCTGAGGTTTGGTGTTTACACTAGACTGATGAGTTGCGAACGGGTGAGTAACGCGTAGG  115239

Query  61      TAACCTGCCTCATAGCGGGGGATAACTATTGGAAACGATAGCTAATACCGCATAAGAGTA  120
               ||||||||||||||||||||||||||||||||||||||||||||||||||||||||||||
Sbjct  115240  TAACCTGCCTCATAGCGGGGGATAACTATTGGAAACGATAGCTAATACCGCATAAGAGTA  115299

Query  121     ATTAACACATGTTAGTTATTTAAAAGGAGCAATTGCTTCACTGTGAGATGGACCTGCGTT  180
               ||||||||||||||||||||||||||||||||||||||||||||||||||||||||||||
Sbjct  115300  ATTAACACATGTTAGTTATTTAAAAGGAGCAATTGCTTCACTGTGAGATGGACCTGCGTT  115359

Query  181     GTATTAGCTAGTTGGTGAGGTAAAGGCTCACCAAGGCGACGATACATAGCCGACCTGAGA  240
               ||||||||||||||||||||||||||||||||||||||||||||||||||||||||||||
Sbjct  115360  GTATTAGCTAGTTGGTGAGGTAAAGGCTCACCAAGGCGACGATACATAGCCGACCTGAGA  115419

Query  241     GGGTGATCGGCCACACTGGGACTGAGACACGGCCCAGACTCCTACGGGAGGCAGCAGTAG  300
               ||||||||||||||||||||||||||||||||||||||||||||||||||||||||||||
Sbjct  115420  GGGTGATCGGCCACACTGGGACTGAGACACGGCCCAGACTCCTACGGGAGGCAGCAGTAG  115479

Query  301     GGAATCTTCGGCAATGGACGGAAGTCTGACCGAGCAACGCCGCGTGAGTGAAGAAGGTTT  360
               ||||||||||||||||||||||||||||||||||||||||||||||||||||||||||||
Sbjct  115480  GGAATCTTCGGCAATGGACGGAAGTCTGACCGAGCAACGCCGCGTGAGTGAAGAAGGTTT  115539

Query  361     TCGGATCGTAAAGCTCTGTTGTTAGAGAAGAACGTTGGTAGGAGTGGAAAATCTACCAAG  420
               ||||||||||||||||||||||||||||||||||||||||||||||||||||||||||||
Sbjct  115540  TCGGATCGTAAAGCTCTGTTGTTAGAGAAGAACGTTGGTAGGAGTGGAAAATCTACCAAG  115599

Query  421     TGACGGTAACTAACCAGAAAGGGACGGCTAACTACGTGCCAGCAGCCGCGGTAATACGTA  480
               ||||||||||||||||||||||||||||||||||||||||||||||||||||||||||||
Sbjct  115600  TGACGGTAACTAACCAGAAAGGGACGGCTAACTACGTGCCAGCAGCCGCGGTAATACGTA  115659

Query  481     GGTCCCGAGCGTTGTCCGGATTTATTGGGCGTAAAGCGAGCGCAGGCGGTTCTTTAAGTC  540
               ||||||||||||||||||||||||||||||||||||||||||||||||||||||||||||
Sbjct  115660  GGTCCCGAGCGTTGTCCGGATTTATTGGGCGTAAAGCGAGCGCAGGCGGTTCTTTAAGTC  115719

Query  541     TGAAGTTAAAGGCAGTGGCTTAACCATTGTACGCTTTGGAAACTGGAGGACTTGAGTGCA  600
               ||||||||||||||||||||||||||||||||||||||||||||||||||||||||||||
Sbjct  115720  TGAAGTTAAAGGCAGTGGCTTAACCATTGTACGCTTTGGAAACTGGAGGACTTGAGTGCA  115779

Query  601     GAAGGGGAGAGTGGAATTCCATGTGTAGCGGTGAAATGCGTAGATATATGGAGGAACACC  660
               ||||||||||||||||||||||||||||||||||||||||||||||||||||||||||||
Sbjct  115780  GAAGGGGAGAGTGGAATTCCATGTGTAGCGGTGAAATGCGTAGATATATGGAGGAACACC  115839

Query  661     GGTGGCGAAAGCGGCTCTCTGGTCTGTAACTGACGCTGAGGCTCGAAAGCGTGGGGAGCA  720
               ||||||||||||||||||||||||||||||||||||||||||||||||||||||||||||
Sbjct  115840  GGTGGCGAAAGCGGCTCTCTGGTCTGTAACTGACGCTGAGGCTCGAAAGCGTGGGGAGCA  115899

Query  721     AACAGGATTAGATACCCTGGTAGTCCACGCCGTAAACGATGAGTGCTAGGTGTTAGGCCC  780
               ||||||||||||||||||||||||||||||||||||||||||||||||||||||||||||
Sbjct  115900  AACAGGATTAGATACCCTGGTAGTCCACGCCGTAAACGATGAGTGCTAGGTGTTAGGCCC  115959

Query  781     TTTCCGGGGCTTAGTGCCGCAGCTAACGCATTAAGCACTC  820
               ||||||||||||||||||||||||||||||||||||||||
Sbjct  115960  TTTCCGGGGCTTAGTGCCGCAGCTAACGCATTAAGCACTC  115999
```

Range 3: 184322 to 185141

Score	Expect	Identities	Gaps	Strand	Frame
1509 bits(817)	0.0()	819/820(99%)	0/820(0%)	Plus/Plus	

Features:

```
Query  1       CGCTGATGTTTGGTGTTTACACTAGACTGATGAGTTGCGAACGGGTGAGTAACGCGTAGG  60
               |||||| |||||||||||||||||||||||||||||||||||||||||||||||||||||
Sbjct  184322  CGCTGAGGTTTGGTGTTTACACTAGACTGATGAGTTGCGAACGGGTGAGTAACGCGTAGG  184381

Query  61      TAACCTGCCTCATAGCGGGGGATAACTATTGGAAACGATAGCTAATACCGCATAAGAGTA  120
               ||||||||||||||||||||||||||||||||||||||||||||||||||||||||||||
Sbjct  184382  TAACCTGCCTCATAGCGGGGGATAACTATTGGAAACGATAGCTAATACCGCATAAGAGTA  184441

Query  121     ATTAACACATGTTAGTTATTTAAAAGGAGCAATTGCTTCACTGTGAGATGGACCTGCGTT  180
               ||||||||||||||||||||||||||||||||||||||||||||||||||||||||||||
Sbjct  184442  ATTAACACATGTTAGTTATTTAAAAGGAGCAATTGCTTCACTGTGAGATGGACCTGCGTT  184501

Query  181     GTATTAGCTAGTTGGTGAGGTAAAGGCTCACCAAGGCGACGATACATAGCCGACCTGAGA  240
               ||||||||||||||||||||||||||||||||||||||||||||||||||||||||||||
Sbjct  184502  GTATTAGCTAGTTGGTGAGGTAAAGGCTCACCAAGGCGACGATACATAGCCGACCTGAGA  184561

Query  241     GGGTGATCGGCCACACTGGGACTGAGACACGGCCCAGACTCCTACGGGAGGCAGCAGTAG  300
               ||||||||||||||||||||||||||||||||||||||||||||||||||||||||||||
Sbjct  184562  GGGTGATCGGCCACACTGGGACTGAGACACGGCCCAGACTCCTACGGGAGGCAGCAGTAG  184621

Query  301     GGAATCTTCGGCAATGGACGGAAGTCTGACCGAGCAACGCCGCGTGAGTGAAGAAGGTTT  360
               ||||||||||||||||||||||||||||||||||||||||||||||||||||||||||||
Sbjct  184622  GGAATCTTCGGCAATGGACGGAAGTCTGACCGAGCAACGCCGCGTGAGTGAAGAAGGTTT  184681

Query  361     TCGGATCGTAAAGCTCTGTTGTTAGAGAAGAACGTTGGTAGGAGTGGAAAATCTACCAAG  420
               ||||||||||||||||||||||||||||||||||||||||||||||||||||||||||||
Sbjct  184682  TCGGATCGTAAAGCTCTGTTGTTAGAGAAGAACGTTGGTAGGAGTGGAAAATCTACCAAG  184741

Query  421     TGACGGTAACTAACCAGAAAGGGACGGCTAACTACGTGCCAGCAGCCGCGGTAATACGTA  480
               ||||||||||||||||||||||||||||||||||||||||||||||||||||||||||||
Sbjct  184742  TGACGGTAACTAACCAGAAAGGGACGGCTAACTACGTGCCAGCAGCCGCGGTAATACGTA  184801

Query  481     GGTCCCGAGCGTTGTCCGGATTTATTGGGCGTAAAGCGAGCGCAGGCGGTTCTTTAAGTC  540
               ||||||||||||||||||||||||||||||||||||||||||||||||||||||||||||
Sbjct  184802  GGTCCCGAGCGTTGTCCGGATTTATTGGGCGTAAAGCGAGCGCAGGCGGTTCTTTAAGTC  184861

Query  541     TGAAGTTAAAGGCAGTGGCTTAACCATTGTACGCTTTGGAAACTGGAGGACTTGAGTGCA  600
               ||||||||||||||||||||||||||||||||||||||||||||||||||||||||||||
Sbjct  184862  TGAAGTTAAAGGCAGTGGCTTAACCATTGTACGCTTTGGAAACTGGAGGACTTGAGTGCA  184921

Query  601     GAAGGGGAGAGTGGAATTCCATGTGTAGCGGTGAAATGCGTAGATATATGGAGGAACACC  660
               ||||||||||||||||||||||||||||||||||||||||||||||||||||||||||||
Sbjct  184922  GAAGGGGAGAGTGGAATTCCATGTGTAGCGGTGAAATGCGTAGATATATGGAGGAACACC  184981

Query  661     GGTGGCGAAAGCGGCTCTCTGGTCTGTAACTGACGCTGAGGCTCGAAAGCGTGGGGAGCA  720
               ||||||||||||||||||||||||||||||||||||||||||||||||||||||||||||
Sbjct  184982  GGTGGCGAAAGCGGCTCTCTGGTCTGTAACTGACGCTGAGGCTCGAAAGCGTGGGGAGCA  185041

Query  721     AACAGGATTAGATACCCTGGTAGTCCACGCCGTAAACGATGAGTGCTAGGTGTTAGGCCC  780
               ||||||||||||||||||||||||||||||||||||||||||||||||||||||||||||
Sbjct  185042  AACAGGATTAGATACCCTGGTAGTCCACGCCGTAAACGATGAGTGCTAGGTGTTAGGCCC  185101

Query  781     TTTCCGGGGCTTAGTGCCGCAGCTAACGCATTAAGCACTC  820
               ||||||||||||||||||||||||||||||||||||||||
Sbjct  185102  TTTCCGGGGCTTAGTGCCGCAGCTAACGCATTAAGCACTC  185141
```

Range 4: 1984086 to 1984905

Score	Expect	Identities	Gaps	Strand	Frame
1509 bits(817)	0.0()	819/820(99%)	0/820(0%)	Plus/Plus	

Features:

```
Query  1        CGCTGATGTTTGGTGTTTACACTAGACTGATGAGTTGCGAACGGGTGAGTAACGCGTAGG  60
                |||||| |||||||||||||||||||||||||||||||||||||||||||||||||||||
Sbjct  1984086  CGCTGAGGTTTGGTGTTTACACTAGACTGATGAGTTGCGAACGGGTGAGTAACGCGTAGG  1984145
Query  61       TAACCTGCCTCATAGCGGGGGATAACTATTGGAAACGATAGCTAATACCGCATAAGAGTA  120
                ||||||||||||||||||||||||||||||||||||||||||||||||||||||||||||
Sbjct  1984146  TAACCTGCCTCATAGCGGGGGATAACTATTGGAAACGATAGCTAATACCGCATAAGAGTA  1984205
Query  121      ATTAACACATGTTAGTTATTTAAAAGGAGCAATTGCTTCACTGTGAGATGGACCTGCGTT  180
                ||||||||||||||||||||||||||||||||||||||||||||||||||||||||||||
Sbjct  1984206  ATTAACACATGTTAGTTATTTAAAAGGAGCAATTGCTTCACTGTGAGATGGACCTGCGTT  1984265
Query  181      GTATTAGCTAGTTGGTGAGGTAAAGGCTCACCAAGGCGACGATACATAGCCGACCTGAGA  240
                ||||||||||||||||||||||||||||||||||||||||||||||||||||||||||||
Sbjct  1984266  GTATTAGCTAGTTGGTGAGGTAAAGGCTCACCAAGGCGACGATACATAGCCGACCTGAGA  1984325
Query  241      GGGTGATCGGCCACACTGGGACTGAGACACGGCCCAGACTCCTACGGGAGGCAGCAGTAG  300
                ||||||||||||||||||||||||||||||||||||||||||||||||||||||||||||
Sbjct  1984326  GGGTGATCGGCCACACTGGGACTGAGACACGGCCCAGACTCCTACGGGAGGCAGCAGTAG  1984385
Query  301      GGAATCTTCGGCAATGGACGGAAGTCTGACCGAGCAACGCCGCGTGAGTGAAGAAGGTTT  360
                ||||||||||||||||||||||||||||||||||||||||||||||||||||||||||||
Sbjct  1984386  GGAATCTTCGGCAATGGACGGAAGTCTGACCGAGCAACGCCGCGTGAGTGAAGAAGGTTT  1984445
Query  361      TCGGATCGTAAAGCTCTGTTGTTAGAGAAGAACGTTGGTAGGAGTGGAAAATCTACCAAG  420
                ||||||||||||||||||||||||||||||||||||||||||||||||||||||||||||
Sbjct  1984446  TCGGATCGTAAAGCTCTGTTGTTAGAGAAGAACGTTGGTAGGAGTGGAAAATCTACCAAG  1984505
Query  421      TGACGGTAACTAACCAGAAAGGGACGGCTAACTACGTGCCAGCAGCCGCGGTAATACGTA  480
                ||||||||||||||||||||||||||||||||||||||||||||||||||||||||||||
Sbjct  1984506  TGACGGTAACTAACCAGAAAGGGACGGCTAACTACGTGCCAGCAGCCGCGGTAATACGTA  1984565
Query  481      GGTCCCGAGCGTTGTCCGGATTTATTGGGCGTAAAGCGAGCGCAGGCGGTTCTTTAAGTC  540
                ||||||||||||||||||||||||||||||||||||||||||||||||||||||||||||
Sbjct  1984566  GGTCCCGAGCGTTGTCCGGATTTATTGGGCGTAAAGCGAGCGCAGGCGGTTCTTTAAGTC  1984625
Query  541      TGAAGTTAAAGGCAGTGGCTTAACCATTGTACGCTTTGGAAACTGGAGGACTTGAGTGCA  600
                ||||||||||||||||||||||||||||||||||||||||||||||||||||||||||||
Sbjct  1984626  TGAAGTTAAAGGCAGTGGCTTAACCATTGTACGCTTTGGAAACTGGAGGACTTGAGTGCA  1984685
Query  601      GAAGGGGAGAGTGGAATTCCATGTGTAGCGGTGAAATGCGTAGATATATGGAGGAACACC  660
                ||||||||||||||||||||||||||||||||||||||||||||||||||||||||||||
Sbjct  1984686  GAAGGGGAGAGTGGAATTCCATGTGTAGCGGTGAAATGCGTAGATATATGGAGGAACACC  1984745
Query  661      GGTGGCGAAAGCGGCTCTCTGGTCTGTAACTGACGCTGAGGCTCGAAAGCGTGGGGAGCA  720
                ||||||||||||||||||||||||||||||||||||||||||||||||||||||||||||
Sbjct  1984746  GGTGGCGAAAGCGGCTCTCTGGTCTGTAACTGACGCTGAGGCTCGAAAGCGTGGGGAGCA  1984805
Query  721      AACAGGATTAGATACCCTGGTAGTCCACGCCGTAAACGATGAGTGCTAGGTGTTAGGCCC  780
                ||||||||||||||||||||||||||||||||||||||||||||||||||||||||||||
Sbjct  1984806  AACAGGATTAGATACCCTGGTAGTCCACGCCGTAAACGATGAGTGCTAGGTGTTAGGCCC  1984865
Query  781      TTTCCGGGGCTTAGTGCCGCAGCTAACGCATTAAGCACTC  820
                ||||||||||||||||||||||||||||||||||||||||
Sbjct  1984866  TTTCCGGGGCTTAGTGCCGCAGCTAACGCATTAAGCACTC  1984905
```

Range 5: 1989918 to 1990737

Score	Expect	Identities	Gaps	Strand	Frame
1509 bits(817)	0.0()	819/820(99%)	0/820(0%)	Plus/Plus	

Features:

```
Query  1        CGCTGATGTTTGGTGTTTACACTAGACTGATGAGTTGCGAACGGGTGAGTAACGCGTAGG  60
                |||||| |||||||||||||||||||||||||||||||||||||||||||||||||||||
Sbjct  1989918  CGCTGAGGTTTGGTGTTTACACTAGACTGATGAGTTGCGAACGGGTGAGTAACGCGTAGG  1989977
Query  61       TAACCTGCCTCATAGCGGGGGATAACTATTGGAAACGATAGCTAATACCGCATAAGAGTA  120
                ||||||||||||||||||||||||||||||||||||||||||||||||||||||||||||
Sbjct  1989978  TAACCTGCCTCATAGCGGGGGATAACTATTGGAAACGATAGCTAATACCGCATAAGAGTA  1990037
Query  121      ATTAACACATGTTAGTTATTTAAAAGGAGCAATTGCTTCACTGTGAGATGGACCTGCGTT  180
                ||||||||||||||||||||||||||||||||||||||||||||||||||||||||||||
Sbjct  1990038  ATTAACACATGTTAGTTATTTAAAAGGAGCAATTGCTTCACTGTGAGATGGACCTGCGTT  1990097
Query  181      GTATTAGCTAGTTGGTGAGGTAAAGGCTCACCAAGGCGACGATACATAGCCGACCTGAGA  240
                ||||||||||||||||||||||||||||||||||||||||||||||||||||||||||||
Sbjct  1990098  GTATTAGCTAGTTGGTGAGGTAAAGGCTCACCAAGGCGACGATACATAGCCGACCTGAGA  1990157
Query  241      GGGTGATCGGCCACACTGGGACTGAGACACGGCCCAGACTCCTACGGGAGGCAGCAGTAG  300
                ||||||||||||||||||||||||||||||||||||||||||||||||||||||||||||
Sbjct  1990158  GGGTGATCGGCCACACTGGGACTGAGACACGGCCCAGACTCCTACGGGAGGCAGCAGTAG  1990217
Query  301      GGAATCTTCGGCAATGGACGGAAGTCTGACCGAGCAACGCCGCGTGAGTGAAGAAGGTTT  360
                ||||||||||||||||||||||||||||||||||||||||||||||||||||||||||||
Sbjct  1990218  GGAATCTTCGGCAATGGACGGAAGTCTGACCGAGCAACGCCGCGTGAGTGAAGAAGGTTT  1990277
Query  361      TCGGATCGTAAAGCTCTGTTGTTAGAGAAGAACGTTGGTAGGAGTGGAAAATCTACCAAG  420
                ||||||||||||||||||||||||||||||||||||||||||||||||||||||||||||
Sbjct  1990278  TCGGATCGTAAAGCTCTGTTGTTAGAGAAGAACGTTGGTAGGAGTGGAAAATCTACCAAG  1990337
Query  421      TGACGGTAACTAACCAGAAAGGGACGGCTAACTACGTGCCAGCAGCCGCGGTAATACGTA  480
                ||||||||||||||||||||||||||||||||||||||||||||||||||||||||||||
Sbjct  1990338  TGACGGTAACTAACCAGAAAGGGACGGCTAACTACGTGCCAGCAGCCGCGGTAATACGTA  1990397
Query  481      GGTCCCGAGCGTTGTCCGGATTTATTGGGCGTAAAGCGAGCGCAGGCGGTTCTTTAAGTC  540
                ||||||||||||||||||||||||||||||||||||||||||||||||||||||||||||
Sbjct  1990398  GGTCCCGAGCGTTGTCCGGATTTATTGGGCGTAAAGCGAGCGCAGGCGGTTCTTTAAGTC  1990457
Query  541      TGAAGTTAAAGGCAGTGGCTTAACCATTGTACGCTTTGGAAACTGGAGGACTTGAGTGCA  600
                ||||||||||||||||||||||||||||||||||||||||||||||||||||||||||||
Sbjct  1990458  TGAAGTTAAAGGCAGTGGCTTAACCATTGTACGCTTTGGAAACTGGAGGACTTGAGTGCA  1990517
Query  601      GAAGGGGAGAGTGGAATTCCATGTGTAGCGGTGAAATGCGTAGATATATGGAGGAACACC  660
                ||||||||||||||||||||||||||||||||||||||||||||||||||||||||||||
Sbjct  1990518  GAAGGGGAGAGTGGAATTCCATGTGTAGCGGTGAAATGCGTAGATATATGGAGGAACACC  1990577
Query  661      GGTGGCGAAAGCGGCTCTCTGGTCTGTAACTGACGCTGAGGCTCGAAAGCGTGGGGAGCA  720
                ||||||||||||||||||||||||||||||||||||||||||||||||||||||||||||
Sbjct  1990578  GGTGGCGAAAGCGGCTCTCTGGTCTGTAACTGACGCTGAGGCTCGAAAGCGTGGGGAGCA  1990637
Query  721      AACAGGATTAGATACCCTGGTAGTCCACGCCGTAAACGATGAGTGCTAGGTGTTAGGCCC  780
                ||||||||||||||||||||||||||||||||||||||||||||||||||||||||||||
Sbjct  1990638  AACAGGATTAGATACCCTGGTAGTCCACGCCGTAAACGATGAGTGCTAGGTGTTAGGCCC  1990697
Query  781      TTTCCGGGGCTTAGTGCCGCAGCTAACGCATTAAGCACTC  820
                ||||||||||||||||||||||||||||||||||||||||
Sbjct  1990698  TTTCCGGGGCTTAGTGCCGCAGCTAACGCATTAAGCACTC  1990737
```

Range 6: 2058895 to 2059714

Score	Expect	Identities	Gaps	Strand	Frame
1509 bits(817)	0.0()	819/820(99%)	0/820(0%)	Plus/Plus	

Features:

```
Query  1        CGCTGATGTTTGGTGTTTACACTAGACTGATGAGTTGCGAACGGGTGAGTAACGCGTAGG  60
                |||||| |||||||||||||||||||||||||||||||||||||||||||||||||||||
Sbjct  2058895  CGCTGAGGTTTGGTGTTTACACTAGACTGATGAGTTGCGAACGGGTGAGTAACGCGTAGG  2058954
Query  61       TAACCTGCCTCATAGCGGGGGATAACTATTGGAAACGATAGCTAATACCGCATAAGAGTA  120
                ||||||||||||||||||||||||||||||||||||||||||||||||||||||||||||
Sbjct  2058955  TAACCTGCCTCATAGCGGGGGATAACTATTGGAAACGATAGCTAATACCGCATAAGAGTA  2059014
Query  121      ATTAACACATGTTAGTTATTTAAAAGGAGCAATTGCTTCACTGTGAGATGGACCTGCGTT  180
                ||||||||||||||||||||||||||||||||||||||||||||||||||||||||||||
Sbjct  2059015  ATTAACACATGTTAGTTATTTAAAAGGAGCAATTGCTTCACTGTGAGATGGACCTGCGTT  2059074
Query  181      GTATTAGCTAGTTGGTGAGGTAAAGGCTCACCAAGGCGACGATACATAGCCGACCTGAGA  240
```

```
                ||||||||||||||||||||||||||||||||||||||||||||||||||||||||||||
Sbjct  2059075  GTATTAGCTAGTTGGTGAGGTAAAGGCTCACCAAGGCGACGATACATAGCCGACCTGAGA  2059134
Query  241      GGGTGATCGGCCACACTGGGACTGAGACACGGCCCAGACTCCTACGGGAGGCAGCAGTAG  300
                ||||||||||||||||||||||||||||||||||||||||||||||||||||||||||||
Sbjct  2059135  GGGTGATCGGCCACACTGGGACTGAGACACGGCCCAGACTCCTACGGGAGGCAGCAGTAG  2059194
Query  301      GGAATCTTCGGCAATGGACGGAAGTCTGACCGAGCAACGCCGCGTGAGTGAAGAAGGTTT  360
                ||||||||||||||||||||||||||||||||||||||||||||||||||||||||||||
Sbjct  2059195  GGAATCTTCGGCAATGGACGGAAGTCTGACCGAGCAACGCCGCGTGAGTGAAGAAGGTTT  2059254
Query  361      TCGGATCGTAAAGCTCTGTTGTTAGAGAAGAACGTTGGTAGGAGTGGAAAATCTACCAAG  420
                ||||||||||||||||||||||||||||||||||||||||||||||||||||||||||||
Sbjct  2059255  TCGGATCGTAAAGCTCTGTTGTTAGAGAAGAACGTTGGTAGGAGTGGAAAATCTACCAAG  2059314
Query  421      TGACGGTAACTAACCAGAAAGGGACGGCTAACTACGTGCCAGCAGCCGCGGTAATACGTA  480
                ||||||||||||||||||||||||||||||||||||||||||||||||||||||||||||
Sbjct  2059315  TGACGGTAACTAACCAGAAAGGGACGGCTAACTACGTGCCAGCAGCCGCGGTAATACGTA  2059374
Query  481      GGTCCCGAGCGTTGTCCGGATTTATTGGGCGTAAAGCGAGCGCAGGCGGTTCTTTAAGTC  540
                ||||||||||||||||||||||||||||||||||||||||||||||||||||||||||||
Sbjct  2059375  GGTCCCGAGCGTTGTCCGGATTTATTGGGCGTAAAGCGAGCGCAGGCGGTTCTTTAAGTC  2059434
Query  541      TGAAGTTAAAGGCAGTGGCTTAACCATTGTACGCTTTGGAAACTGGAGGACTTGAGTGCA  600
                ||||||||||||||||||||||||||||||||||||||||||||||||||||||||||||
Sbjct  2059435  TGAAGTTAAAGGCAGTGGCTTAACCATTGTACGCTTTGGAAACTGGAGGACTTGAGTGCA  2059494
Query  601      GAAGGGGAGAGTGGAATTCCATGTGTAGCGGTGAAATGCGTAGATATATGGAGGAACACC  660
                ||||||||||||||||||||||||||||||||||||||||||||||||||||||||||||
Sbjct  2059495  GAAGGGGAGAGTGGAATTCCATGTGTAGCGGTGAAATGCGTAGATATATGGAGGAACACC  2059554
Query  661      GGTGGCGAAAGCGGCTCTCTGGTCTGTAACTGACGCTGAGGCTCGAAAGCGTGGGGAGCA  720
                ||||||||||||||||||||||||||||||||||||||||||||||||||||||||||||
Sbjct  2059555  GGTGGCGAAAGCGGCTCTCTGGTCTGTAACTGACGCTGAGGCTCGAAAGCGTGGGGAGCA  2059614
Query  721      AACAGGATTAGATACCCTGGTAGTCCACGCCGTAAACGATGAGTGCTAGGTGTTAGGCCC  780
                ||||||||||||||||||||||||||||||||||||||||||||||||||||||||||||
Sbjct  2059615  AACAGGATTAGATACCCTGGTAGTCCACGCCGTAAACGATGAGTGCTAGGTGTTAGGCCC  2059674
Query  781      TTTCCGGGGCTTAGTGCCGCAGCTAACGCATTAAGCACTC  820
                ||||||||||||||||||||||||||||||||||||||||
Sbjct  2059675  TTTCCGGGGCTTAGTGCCGCAGCTAACGCATTAAGCACTC  2059714
```

Range 7: 2132924 to 2133743

Score	Expect	Identities	Gaps	Strand	Frame
1509 bits(817)	0.0()	819/820(99%)	0/820(0%)	Plus/Plus	

Features:

```
Query  1        CGCTGATGTTTGGTGTTTACACTAGACTGATGAGTTGCGAACGGGTGAGTAACGCGTAGG  60
                |||||| |||||||||||||||||||||||||||||||||||||||||||||||||||||
Sbjct  2132924  CGCTGAGGTTTGGTGTTTACACTAGACTGATGAGTTGCGAACGGGTGAGTAACGCGTAGG  2132983
Query  61       TAACCTGCCTCATAGCGGGGGATAACTATTGGAAACGATAGCTAATACCGCATAAGAGTA  120
                ||||||||||||||||||||||||||||||||||||||||||||||||||||||||||||
Sbjct  2132984  TAACCTGCCTCATAGCGGGGGATAACTATTGGAAACGATAGCTAATACCGCATAAGAGTA  2133043
Query  121      ATTAACACATGTTAGTTATTTAAAAGGAGCAATTGCTTCACTGTGAGATGGACCTGCGTT  180
                ||||||||||||||||||||||||||||||||||||||||||||||||||||||||||||
Sbjct  2133044  ATTAACACATGTTAGTTATTTAAAAGGAGCAATTGCTTCACTGTGAGATGGACCTGCGTT  2133103
Query  181      GTATTAGCTAGTTGGTGAGGTAAAGGCTCACCAAGGCGACGATACATAGCCGACCTGAGA  240
                ||||||||||||||||||||||||||||||||||||||||||||||||||||||||||||
Sbjct  2133104  GTATTAGCTAGTTGGTGAGGTAAAGGCTCACCAAGGCGACGATACATAGCCGACCTGAGA  2133163
Query  241      GGGTGATCGGCCACACTGGGACTGAGACACGGCCCAGACTCCTACGGGAGGCAGCAGTAG  300
                ||||||||||||||||||||||||||||||||||||||||||||||||||||||||||||
Sbjct  2133164  GGGTGATCGGCCACACTGGGACTGAGACACGGCCCAGACTCCTACGGGAGGCAGCAGTAG  2133223
Query  301      GGAATCTTCGGCAATGGACGGAAGTCTGACCGAGCAACGCCGCGTGAGTGAAGAAGGTTT  360
                ||||||||||||||||||||||||||||||||||||||||||||||||||||||||||||
Sbjct  2133224  GGAATCTTCGGCAATGGACGGAAGTCTGACCGAGCAACGCCGCGTGAGTGAAGAAGGTTT  2133283
Query  361      TCGGATCGTAAAGCTCTGTTGTTAGAGAAGAACGTTGGTAGGAGTGGAAAATCTACCAAG  420
                ||||||||||||||||||||||||||||||||||||||||||||||||||||||||||||
Sbjct  2133284  TCGGATCGTAAAGCTCTGTTGTTAGAGAAGAACGTTGGTAGGAGTGGAAAATCTACCAAG  2133343
Query  421      TGACGGTAACTAACCAGAAAGGGACGGCTAACTACGTGCCAGCAGCCGCGGTAATACGTA  480
                ||||||||||||||||||||||||||||||||||||||||||||||||||||||||||||
Sbjct  2133344  TGACGGTAACTAACCAGAAAGGGACGGCTAACTACGTGCCAGCAGCCGCGGTAATACGTA  2133403
Query  481      GGTCCCGAGCGTTGTCCGGATTTATTGGGCGTAAAGCGAGCGCAGGCGGTTCTTTAAGTC  540
                ||||||||||||||||||||||||||||||||||||||||||||||||||||||||||||
Sbjct  2133404  GGTCCCGAGCGTTGTCCGGATTTATTGGGCGTAAAGCGAGCGCAGGCGGTTCTTTAAGTC  2133463
Query  541      TGAAGTTAAAGGCAGTGGCTTAACCATTGTACGCTTTGGAAACTGGAGGACTTGAGTGCA  600
                ||||||||||||||||||||||||||||||||||||||||||||||||||||||||||||
Sbjct  2133464  TGAAGTTAAAGGCAGTGGCTTAACCATTGTACGCTTTGGAAACTGGAGGACTTGAGTGCA  2133523
Query  601      GAAGGGGAGAGTGGAATTCCATGTGTAGCGGTGAAATGCGTAGATATATGGAGGAACACC  660
                ||||||||||||||||||||||||||||||||||||||||||||||||||||||||||||
Sbjct  2133524  GAAGGGGAGAGTGGAATTCCATGTGTAGCGGTGAAATGCGTAGATATATGGAGGAACACC  2133583
Query  661      GGTGGCGAAAGCGGCTCTCTGGTCTGTAACTGACGCTGAGGCTCGAAAGCGTGGGGAGCA  720
                ||||||||||||||||||||||||||||||||||||||||||||||||||||||||||||
Sbjct  2133584  GGTGGCGAAAGCGGCTCTCTGGTCTGTAACTGACGCTGAGGCTCGAAAGCGTGGGGAGCA  2133643
Query  721      AACAGGATTAGATACCCTGGTAGTCCACGCCGTAAACGATGAGTGCTAGGTGTTAGGCCC  780
                ||||||||||||||||||||||||||||||||||||||||||||||||||||||||||||
Sbjct  2133644  AACAGGATTAGATACCCTGGTAGTCCACGCCGTAAACGATGAGTGCTAGGTGTTAGGCCC  2133703
Query  781      TTTCCGGGGCTTAGTGCCGCAGCTAACGCATTAAGCACTC  820
                ||||||||||||||||||||||||||||||||||||||||
Sbjct  2133704  TTTCCGGGGCTTAGTGCCGCAGCTAACGCATTAAGCACTC  2133743
```

BLAST is a registered trademark of the National Library of Medicine

Support center Mailing list YouTube

- National Library Of Medicine
- National Institutes Of Health
- U.S. Department of Health & Human Services
- USA.gov

NCBI
National Center for Biotechnology Information, U.S. National Library of Medicine 8600 Rockville Pike, Bethesda MD, 20894 USA
Policies and Guidelines | Contact
