# Supplementary material for: Isolation of Streptococcus agalactiae in a female llama (Lama glama) in South Tyrol (Italy)
Source: BMC Vet Res. 2018 Nov 13;14:343. doi: 10.1186/s12917-018-1676-9 (PMC6234556; doi:10.1186/s12917-018-1676-9)
Supplement: Supplementary file 2 — BLAST allignment of the lung isolate. (PDF 397 kb) [file 12917_2018_1676_MOESM2_ESM.pdf]

BLAST Results

Questions/comments

Job title: Consensus for segment MG386600 for specimen...

**RID**	RDAW2742014 (Expires on 08-18 19:03 pm)
**Query ID**	lcl|Query_34053
**Description**	Consensus for segment MG386600 for specimen 18RS-1924-6
**Molecule type**	nucleic acid
**Query Length**	810

**Database Name**	nr
**Description**	Nucleotide collection (nt)
**Program**	BLASTN 2.8.0+

Graphic Summary

Distribution of the top 200 Blast Hits on 100 subject sequences

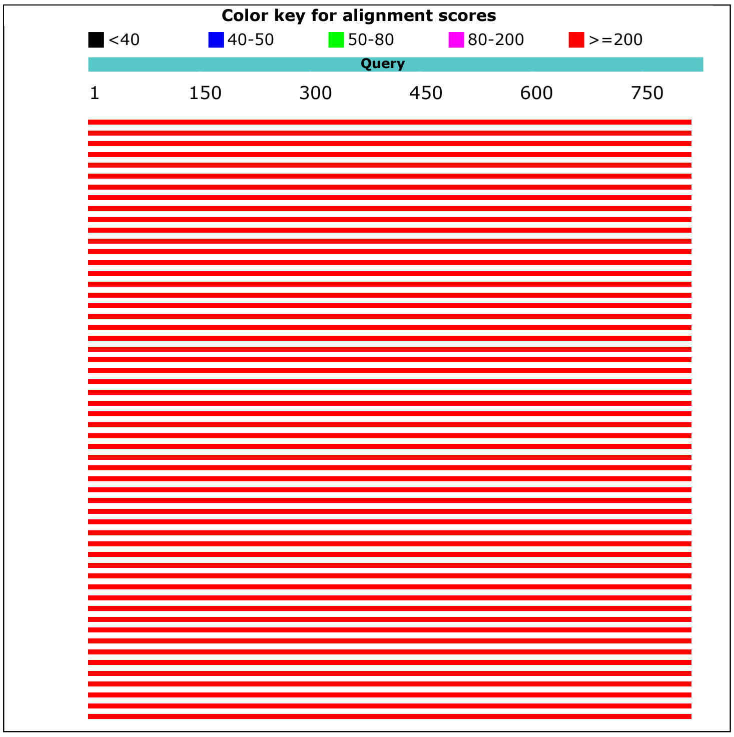

Descriptions

Sequences producing significant alignments:

Description	Max score	Total score	Query cover	E value	Ident	Accession
Streptococcus agalactiae strain GZ120 16S ribosomal RNA gene, partial sequence	1496	1496	100%	0.0	100%	MG386600.1
Streptococcus agalactiae strain B111 chromosome, complete genome	1496	10478	100%	0.0	100%	CP021772.1
Streptococcus agalactiae strain B507 chromosome, complete genome	1496	10461	100%	0.0	100%	CP021771.1
Streptococcus agalactiae strain B509 chromosome, complete genome	1496	10467	100%	0.0	100%	CP021769.1
Streptococcus agalactiae strain NCTC11930 genome assembly, chromosome: 1	1496	10456	100%	0.0	100%	LS483342.1
Streptococcus agalactiae strain SGEHI2015-25 chromosome, complete genome	1496	10450	100%	0.0	100%	CP025029.1
Streptococcus agalactiae strain SGEHI2015-107 chromosome, complete genome	1496	10445	100%	0.0	100%	CP025027.1
Streptococcus agalactiae strain QX1503 16S ribosomal RNA gene, partial sequence	1496	1496	100%	0.0	100%	MF113268.1
Streptococcus agalactiae strain HN1517 16S ribosomal RNA gene, partial sequence	1496	1496	100%	0.0	100%	MF113266.1
Streptococcus agalactiae strain HN1503 16S ribosomal RNA gene, partial sequence	1496	1496	100%	0.0	100%	MF113265.1
Streptococcus agalactiae strain GXN1417 16S ribosomal RNA gene, partial sequence	1496	1496	100%	0.0	100%	MF113264.1
Streptococcus agalactiae strain GXN1402 16S ribosomal RNA gene, partial sequence	1496	1496	100%	0.0	100%	MF113263.1
Streptococcus agalactiae strain GD1221 16S ribosomal RNA gene, partial sequence	1496	1496	100%	0.0	100%	MF113262.1
Streptococcus agalactiae strain GD1212 16S ribosomal RNA gene, partial sequence	1496	1496	100%	0.0	100%	MF113261.1
Streptococcus agalactiae strain NYD007 16S ribosomal RNA gene, partial sequence	1496	1496	100%	0.0	100%	MF113259.1
Streptococcus agalactiae strain NYD001 16S ribosomal RNA gene, partial sequence	1496	1496	100%	0.0	100%	MF113258.1
Streptococcus agalactiae strain BJ1411 16S ribosomal RNA gene, partial sequence	1496	1496	100%	0.0	100%	MF113257.1
Streptococcus agalactiae strain QX1501 16S ribosomal RNA gene, partial sequence	1496	1496	100%	0.0	100%	MF037804.1
Streptococcus agalactiae strain PK1601 16S ribosomal RNA gene, partial sequence	1496	1496	100%	0.0	100%	MF037803.1
Streptococcus agalactiae strain HN1506 16S ribosomal RNA gene, partial sequence	1496	1496	100%	0.0	100%	MF037798.1
Streptococcus agalactiae strain GXN1411 16S ribosomal RNA gene, partial sequence	1496	1496	100%	0.0	100%	MF037796.1
Streptococcus agalactiae strain NYD03 16S ribosomal RNA gene, partial sequence	1496	1496	100%	0.0	100%	MF037794.1
Streptococcus agalactiae strain NZY014 16S ribosomal RNA gene, partial sequence	1496	1496	100%	0.0	100%	MF037793.1
Streptococcus agalactiae strain BJ1402 16S ribosomal RNA gene, partial sequence	1496	1496	100%	0.0	100%	MF037792.1
Streptococcus agalactiae strain ZJRZ1623 16S ribosomal RNA gene, partial sequence	1496	1496	100%	0.0	100%	MF037790.1
Streptococcus agalactiae strain ZJRZ1620 16S ribosomal RNA gene, partial sequence	1496	1496	100%	0.0	100%	MF037789.1
Streptococcus agalactiae strain ZJRZ1619 16S ribosomal RNA gene, partial sequence	1496	1496	100%	0.0	100%	MF037788.1
Streptococcus agalactiae strain ZJRZ1604 16S ribosomal RNA gene, partial sequence	1496	1496	100%	0.0	100%	MF037786.1
Streptococcus agalactiae strain ZJRZ1603 16S ribosomal RNA gene, partial sequence	1496	1496	100%	0.0	100%	MF037785.1
Streptococcus agalactiae strain NJJNB1619 16S ribosomal RNA gene, partial sequence	1496	1496	100%	0.0	100%	MF037784.1
Streptococcus agalactiae strain NJJNB1612 16S ribosomal RNA gene, partial sequence	1496	1496	100%	0.0	100%	MF037783.1
Streptococcus agalactiae strain NJJNB1608 16S ribosomal RNA gene, partial sequence	1496	1496	100%	0.0	100%	MF037782.1
Streptococcus agalactiae strain NJJNB1606 16S ribosomal RNA gene, partial sequence	1496	1496	100%	0.0	100%	MF037781.1
Streptococcus agalactiae strain NJJNB1602 16S ribosomal RNA gene, partial sequence	1496	1496	100%	0.0	100%	MF037780.1
Streptococcus agalactiae strain HAHY1335 16S ribosomal RNA gene, partial sequence	1496	1496	100%	0.0	100%	MF037778.1
Streptococcus agalactiae strain HAHY1332 16S ribosomal RNA gene, partial sequence	1496	1496	100%	0.0	100%	MF037776.1
Streptococcus agalactiae strain HAHY1322 16S ribosomal RNA gene, partial sequence	1496	1496	100%	0.0	100%	MF037775.1
Streptococcus agalactiae strain HAHY1315 16S ribosomal RNA gene, partial sequence	1496	1496	100%	0.0	100%	MF037774.1
Streptococcus agalactiae strain HAQH1336 16S ribosomal RNA gene, partial sequence	1496	1496	100%	0.0	100%	MF037772.1
Streptococcus agalactiae strain HAQH1335 16S ribosomal RNA gene, partial sequence	1496	1496	100%	0.0	100%	MF037771.1
Streptococcus agalactiae strain HAQH1312 16S ribosomal RNA gene, partial sequence	1496	1496	100%	0.0	100%	MF037768.1
Streptococcus agalactiae strain HAQH1309 16S ribosomal RNA gene, partial sequence	1496	1496	100%	0.0	100%	MF037767.1
Streptococcus agalactiae strain NJJNA1632 16S ribosomal RNA gene, partial sequence	1496	1496	100%	0.0	100%	MF037766.1
Streptococcus agalactiae strain NJJNA1619 16S ribosomal RNA gene, partial sequence	1496	1496	100%	0.0	100%	MF037764.1
Streptococcus agalactiae strain NJJNA1613 16S ribosomal RNA gene, partial sequence	1496	1496	100%	0.0	100%	MF037763.1
Streptococcus agalactiae strain NJJNA1612 16S ribosomal RNA gene, partial sequence	1496	1496	100%	0.0	100%	MF037762.1
Streptococcus agalactiae strain NJJNA1608 16S ribosomal RNA gene, partial sequence	1496	1496	100%	0.0	100%	MF037761.1
Streptococcus agalactiae strain NJPK1419 16S ribosomal RNA gene, partial sequence	1496	1496	100%	0.0	100%	MF037759.1
Streptococcus agalactiae strain NJPK1407 16S ribosomal RNA gene, partial sequence	1496	1496	100%	0.0	100%	MF037757.1
Streptococcus agalactiae strain NJPK1406 16S ribosomal RNA gene, partial sequence	1496	1496	100%	0.0	100%	MF037756.1
Streptococcus agalactiae strain HZJG1236 16S ribosomal RNA gene, partial sequence	1496	1496	100%	0.0	100%	MF037754.1
Streptococcus agalactiae strain HZJG1228 16S ribosomal RNA gene, partial sequence	1496	1496	100%	0.0	100%	MF037753.1
Streptococcus agalactiae strain HZJG1216 16S ribosomal RNA gene, partial sequence	1496	1496	100%	0.0	100%	MF037751.1
Streptococcus agalactiae strain HZJG1211 16S ribosomal RNA gene, partial sequence	1496	1496	100%	0.0	100%	MF037750.1
Streptococcus agalactiae strain CZFY1231 16S ribosomal RNA gene, partial sequence	1496	1496	100%	0.0	100%	MF037748.1
Streptococcus agalactiae strain CZFY1227 16S ribosomal RNA gene, partial sequence	1496	1496	100%	0.0	100%	MF037747.1
Streptococcus agalactiae strain CZFY1214 16S ribosomal RNA gene, partial sequence	1496	1496	100%	0.0	100%	MF037746.1
Streptococcus agalactiae strain CZFY1209 16S ribosomal RNA gene, partial sequence	1496	1496	100%	0.0	100%	MF037744.1
Streptococcus agalactiae strain CZFY1207 16S ribosomal RNA gene, partial sequence	1496	1496	100%	0.0	100%	MF037743.1
Streptococcus agalactiae strain SHFX1111 16S ribosomal RNA gene, partial sequence	1496	1496	100%	0.0	100%	MF037741.1
Streptococcus agalactiae strain SHFX1106 16S ribosomal RNA gene, partial sequence	1496	1496	100%	0.0	100%	MF037740.1
Streptococcus agalactiae strain SHFX1102 16S ribosomal RNA gene, partial sequence	1496	1496	100%	0.0	100%	MF037738.1

Description	Max score	Total score	Query cover	E value	Ident	Accession
Streptococcus agalactiae strain SHFX1101 16S ribosomal RNA gene, partial sequence	1496	1496	100%	0.0	100%	MF037737.1
Streptococcus agalactiae strain 3X23 16S ribosomal RNA gene, partial sequence	1496	1496	100%	0.0	100%	KY765027.1
Streptococcus agalactiae strain C001, complete genome	1496	8981	100%	0.0	100%	CP008813.1
Streptococcus agalactiae strain SG-M8, complete genome	1496	10478	100%	0.0	100%	CP021868.1
Streptococcus agalactiae strain CUGBS591, complete genome	1496	10467	100%	0.0	100%	CP021862.1
Streptococcus agalactiae strain Sag37, complete genome	1496	10446	100%	0.0	100%	CP019978.1
Streptococcus agalactiae strain GBS-M002, complete genome	1496	10478	100%	0.0	100%	CP013908.1
Uncultured bacterium clone gao21 16S ribosomal RNA gene, partial sequence	1496	1496	100%	0.0	100%	KU363876.1
Streptococcus agalactiae strain WC1535, complete genome	1496	10434	100%	0.0	100%	CP016501.1
Streptococcus agalactiae strain CU_GBS_98, complete genome	1496	7462	100%	0.0	100%	CP010875.1
Streptococcus agalactiae strain CU_GBS_08 chromosome, complete genome	1496	7462	100%	0.0	100%	CP010874.1
Streptococcus agalactiae strain GBS85147, complete genome	1496	8955	100%	0.0	100%	CP010319.1
Streptococcus agalactiae strain GX064 genome	1496	8953	100%	0.0	100%	CP011327.1
Streptococcus agalactiae strain YM001 genome	1496	8953	100%	0.0	100%	CP011326.1
Streptococcus agalactiae strain HN016, complete genome	1496	10445	100%	0.0	100%	CP011325.1
Streptococcus agalactiae strain HZ10 16S ribosomal RNA gene, partial sequence	1496	1496	100%	0.0	100%	KF111311.1
Uncultured Streptococcus sp. clone M52 16S ribosomal RNA gene, partial sequence	1496	1496	100%	0.0	100%	JX841329.1
Uncultured Streptococcus sp. clone M11 16S ribosomal RNA gene, partial sequence	1496	1496	100%	0.0	100%	JX841322.1
Streptococcus agalactiae GD201008-001, complete genome	1496	10413	100%	0.0	100%	CP003810.1
Streptococcus agalactiae strain B7 16S ribosomal RNA gene, partial sequence	1496	1496	100%	0.0	100%	JN176347.1
Uncultured bacterium clone GoC_Bac_17_D1_C0_M0 16S ribosomal RNA gene, partial sequence	1496	1496	100%	0.0	100%	FJ813519.1
Streptococcus agalactiae strain 14-ninetytwomp 16S ribosomal RNA gene, partial sequence	1496	1496	100%	0.0	100%	EU075069.1
Streptococcus agalactiae A909, complete genome	1496	10450	100%	0.0	100%	CP000114.1
Streptococcus agalactiae strain GZ2058 16S ribosomal RNA gene, partial sequence	1495	1495	99%	0.0	100%	MG386601.1
Streptococcus agalactiae strain B105 chromosome, complete genome	1495	10465	99%	0.0	100%	CP021773.1
Streptococcus agalactiae strain B508 chromosome, complete genome	1495	10465	99%	0.0	100%	CP021770.1
Streptococcus agalactiae strain NCTC8187 genome assembly, chromosome: 1	1495	8951	100%	0.0	100%	LS483387.1
Streptococcus agalactiae strain L0023-06 16S ribosomal RNA gene, partial sequence	1495	1495	99%	0.0	100%	MH463668.1
Streptococcus agalactiae strain L0023-02F 16S ribosomal RNA gene, partial sequence	1495	1495	99%	0.0	100%	MH447034.1
Streptococcus agalactiae strain NQ-2018-5 16S ribosomal RNA gene, partial sequence	1495	1495	99%	0.0	100%	MH423900.1
Streptococcus agalactiae strain QMA0271 chromosome, complete genome	1495	2990	99%	0.0	100%	CP029632.1
Streptococcus agalactiae strain 16CS3 16S ribosomal RNA gene, partial sequence	1495	1495	99%	0.0	100%	MH158279.1
Streptococcus agalactiae strain 7PS3 16S ribosomal RNA gene, partial sequence	1495	1495	99%	0.0	100%	MH109009.1
Streptococcus agalactiae strain YU12C1 MCC 3039 16S ribosomal RNA gene, partial sequence	1495	1495	99%	0.0	100%	MH021640.1
Streptococcus agalactiae strain YUC18-1 MCC 3038 16S ribosomal RNA gene, partial sequence	1495	1495	99%	0.0	100%	MH021639.1
Streptococcus agalactiae strain FDAARGOS_254 chromosome, complete genome	1495	10465	99%	0.0	100%	CP020449.2
Streptococcus sp. 'group B' strain FDAARGOS_229 chromosome, complete genome	1495	10443	99%	0.0	100%	CP020432.2
Streptococcus agalactiae strain SG-M6, complete genome	1495	10465	99%	0.0	100%	CP021869.1

Alignments

Streptococcus agalactiae strain GZ120 16S ribosomal RNA gene, partial sequence
Sequence ID: **MG386600.1** Length: 1406 Number of Matches: 1
Range 1: 8 to 817

Score	Expect	Identities	Gaps	Strand	Frame
1496 bits(810)	0.0()	810/810(100%)	0/810(0%)	Plus/Plus	

Features:

```
Query  1    TGTTTGGTGTTTACACTAGACTGATGAGTTGCGAACGGGTGAGTAACGCGTAGGTAACCT  60
            ||||||||||||||||||||||||||||||||||||||||||||||||||||||||||||
Sbjct  8    TGTTTGGTGTTTACACTAGACTGATGAGTTGCGAACGGGTGAGTAACGCGTAGGTAACCT  67

Query  61   GCCTCATAGCGGGGGATAACTATTGGAAACGATAGCTAATACCGCATAAGAGTAATTAAC  120
            ||||||||||||||||||||||||||||||||||||||||||||||||||||||||||||
Sbjct  68   GCCTCATAGCGGGGGATAACTATTGGAAACGATAGCTAATACCGCATAAGAGTAATTAAC  127

Query  121  ACATGTTAGTTATTTAAAAGGAGCAATTGCTTCACTGTGAGATGGACCTGCGTTGTATTA  180
            ||||||||||||||||||||||||||||||||||||||||||||||||||||||||||||
Sbjct  128  ACATGTTAGTTATTTAAAAGGAGCAATTGCTTCACTGTGAGATGGACCTGCGTTGTATTA  187

Query  181  GCTAGTTGGTGAGGTAAAGGCTCACCAAGGCGACGATACATAGCCGACCTGAGAGGGTGA  240
            ||||||||||||||||||||||||||||||||||||||||||||||||||||||||||||
Sbjct  188  GCTAGTTGGTGAGGTAAAGGCTCACCAAGGCGACGATACATAGCCGACCTGAGAGGGTGA  247

Query  241  TCGGCCACACTGGGACTGAGACACGGCCCAGACTCCTACGGGAGGCAGCAGTAGGGAATC  300
            ||||||||||||||||||||||||||||||||||||||||||||||||||||||||||||
Sbjct  248  TCGGCCACACTGGGACTGAGACACGGCCCAGACTCCTACGGGAGGCAGCAGTAGGGAATC  307

Query  301  TTCGGCAATGGACGGAAGTCTGACCGAGCAACGCCGCGTGAGTGAAGAAGGTTTTCGGAT  360
            ||||||||||||||||||||||||||||||||||||||||||||||||||||||||||||
Sbjct  308  TTCGGCAATGGACGGAAGTCTGACCGAGCAACGCCGCGTGAGTGAAGAAGGTTTTCGGAT  367

Query  361  CGTAAAGCTCTGTTGTTAGAGAAGAACGTTGGTAGGAGTGGAAAATCTACCAAGTGACGG  420
            ||||||||||||||||||||||||||||||||||||||||||||||||||||||||||||
Sbjct  368  CGTAAAGCTCTGTTGTTAGAGAAGAACGTTGGTAGGAGTGGAAAATCTACCAAGTGACGG  427

Query  421  TAACTAACCAGAAAGGGACGGCTAACTACGTGCCAGCAGCCGCGGTAATACGTAGGTCCC  480
            ||||||||||||||||||||||||||||||||||||||||||||||||||||||||||||
Sbjct  428  TAACTAACCAGAAAGGGACGGCTAACTACGTGCCAGCAGCCGCGGTAATACGTAGGTCCC  487

Query  481  GAGCGTTGTCCGGATTTATTGGGCGTAAAGCGAGCGCAGGCGGTTCTTTAAGTCTGAAGT  540
            ||||||||||||||||||||||||||||||||||||||||||||||||||||||||||||
Sbjct  488  GAGCGTTGTCCGGATTTATTGGGCGTAAAGCGAGCGCAGGCGGTTCTTTAAGTCTGAAGT  547

Query  541  TAAAGGCAGTGGCTTAACCATTGTACGCTTTGGAAACTGGAGGACTTGAGTGCAGAAGGG  600
            ||||||||||||||||||||||||||||||||||||||||||||||||||||||||||||
Sbjct  548  TAAAGGCAGTGGCTTAACCATTGTACGCTTTGGAAACTGGAGGACTTGAGTGCAGAAGGG  607

Query  601  GAGAGTGGAATTCCATGTGTAGCGGTGAAATGCGTAGATATATGGAGGAACACCGGTGGC  660
            ||||||||||||||||||||||||||||||||||||||||||||||||||||||||||||
Sbjct  608  GAGAGTGGAATTCCATGTGTAGCGGTGAAATGCGTAGATATATGGAGGAACACCGGTGGC  667

Query  661  GAAAGCGGCTCTCTGGTCTGTAACTGACGCTGAGGCTCGAAAGCGTGGGGAGCAAACAGG  720
            ||||||||||||||||||||||||||||||||||||||||||||||||||||||||||||
Sbjct  668  GAAAGCGGCTCTCTGGTCTGTAACTGACGCTGAGGCTCGAAAGCGTGGGGAGCAAACAGG  727
```

```
Query  721  ATTAGATACCCTGGTAGTCCACGCCGTAAACGATGAGTGCTAGGTGTTAGGCCCTTTCCG  780
            ||||||||||||||||||||||||||||||||||||||||||||||||||||||||||||
Sbjct  728  ATTAGATACCCTGGTAGTCCACGCCGTAAACGATGAGTGCTAGGTGTTAGGCCCTTTCCG  787

Query  781  GGGCTTAGTGCCGCAGCTAACGCATTAAGC  810
            ||||||||||||||||||||||||||||||
Sbjct  788  GGGCTTAGTGCCGCAGCTAACGCATTAAGC  817
```

Streptococcus agalactiae strain B111 chromosome, complete genome
Sequence ID: **CP021772.1** Length: 2150631 Number of Matches: 7
Range 1: 1414836 to 1415645

Score	Expect	Identities	Gaps	Strand	Frame
1496 bits(810)	0.0()	810/810(100%)	0/810(0%)	Plus/Plus	

Features:

```
Query  1        TGTTTGGTGTTTACACTAGACTGATGAGTTGCGAACGGGTGAGTAACGCGTAGGTAACCT  60
                ||||||||||||||||||||||||||||||||||||||||||||||||||||||||||||
Sbjct  1414836  TGTTTGGTGTTTACACTAGACTGATGAGTTGCGAACGGGTGAGTAACGCGTAGGTAACCT  1414895
Query  61       GCCTCATAGCGGGGGATAACTATTGGAAACGATAGCTAATACCGCATAAGAGTAATTAAC  120
                ||||||||||||||||||||||||||||||||||||||||||||||||||||||||||||
Sbjct  1414896  GCCTCATAGCGGGGGATAACTATTGGAAACGATAGCTAATACCGCATAAGAGTAATTAAC  1414955
Query  121      ACATGTTAGTTATTTAAAAGGAGCAATTGCTTCACTGTGAGATGGACCTGCGTTGTATTA  180
                ||||||||||||||||||||||||||||||||||||||||||||||||||||||||||||
Sbjct  1414956  ACATGTTAGTTATTTAAAAGGAGCAATTGCTTCACTGTGAGATGGACCTGCGTTGTATTA  1415015
Query  181      GCTAGTTGGTGAGGTAAAGGCTCACCAAGGCGACGATACATAGCCGACCTGAGAGGGTGA  240
                ||||||||||||||||||||||||||||||||||||||||||||||||||||||||||||
Sbjct  1415016  GCTAGTTGGTGAGGTAAAGGCTCACCAAGGCGACGATACATAGCCGACCTGAGAGGGTGA  1415075
Query  241      TCGGCCACACTGGGACTGAGACACGGCCCAGACTCCTACGGGAGGCAGCAGTAGGGAATC  300
                ||||||||||||||||||||||||||||||||||||||||||||||||||||||||||||
Sbjct  1415076  TCGGCCACACTGGGACTGAGACACGGCCCAGACTCCTACGGGAGGCAGCAGTAGGGAATC  1415135
Query  301      TTCGGCAATGGACGGAAGTCTGACCGAGCAACGCCGCGTGAGTGAAGAAGGTTTTCGGAT  360
                ||||||||||||||||||||||||||||||||||||||||||||||||||||||||||||
Sbjct  1415136  TTCGGCAATGGACGGAAGTCTGACCGAGCAACGCCGCGTGAGTGAAGAAGGTTTTCGGAT  1415195
Query  361      CGTAAAGCTCTGTTGTTAGAGAAGAACGTTGGTAGGAGTGGAAAATCTACCAAGTGACGG  420
                ||||||||||||||||||||||||||||||||||||||||||||||||||||||||||||
Sbjct  1415196  CGTAAAGCTCTGTTGTTAGAGAAGAACGTTGGTAGGAGTGGAAAATCTACCAAGTGACGG  1415255
Query  421      TAACTAACCAGAAAGGGACGGCTAACTACGTGCCAGCAGCCGCGGTAATACGTAGGTCCC  480
                ||||||||||||||||||||||||||||||||||||||||||||||||||||||||||||
Sbjct  1415256  TAACTAACCAGAAAGGGACGGCTAACTACGTGCCAGCAGCCGCGGTAATACGTAGGTCCC  1415315
Query  481      GAGCGTTGTCCGGATTTATTGGGCGTAAAGCGAGCGCAGGCGGTTCTTTAAGTCTGAAGT  540
                ||||||||||||||||||||||||||||||||||||||||||||||||||||||||||||
Sbjct  1415316  GAGCGTTGTCCGGATTTATTGGGCGTAAAGCGAGCGCAGGCGGTTCTTTAAGTCTGAAGT  1415375
Query  541      TAAAGGCAGTGGCTTAACCATTGTACGCTTTGGAAACTGGAGGACTTGAGTGCAGAAGGG  600
                ||||||||||||||||||||||||||||||||||||||||||||||||||||||||||||
Sbjct  1415376  TAAAGGCAGTGGCTTAACCATTGTACGCTTTGGAAACTGGAGGACTTGAGTGCAGAAGGG  1415435
Query  601      GAGAGTGGAATTCCATGTGTAGCGGTGAAATGCGTAGATATATGGAGGAACACCGGTGGC  660
                ||||||||||||||||||||||||||||||||||||||||||||||||||||||||||||
Sbjct  1415436  GAGAGTGGAATTCCATGTGTAGCGGTGAAATGCGTAGATATATGGAGGAACACCGGTGGC  1415495
Query  661      GAAAGCGGCTCTCTGGTCTGTAACTGACGCTGAGGCTCGAAAGCGTGGGGAGCAAACAGG  720
                ||||||||||||||||||||||||||||||||||||||||||||||||||||||||||||
Sbjct  1415496  GAAAGCGGCTCTCTGGTCTGTAACTGACGCTGAGGCTCGAAAGCGTGGGGAGCAAACAGG  1415555
Query  721      ATTAGATACCCTGGTAGTCCACGCCGTAAACGATGAGTGCTAGGTGTTAGGCCCTTTCCG  780
                ||||||||||||||||||||||||||||||||||||||||||||||||||||||||||||
Sbjct  1415556  ATTAGATACCCTGGTAGTCCACGCCGTAAACGATGAGTGCTAGGTGTTAGGCCCTTTCCG  1415615
Query  781      GGGCTTAGTGCCGCAGCTAACGCATTAAGC  810
                ||||||||||||||||||||||||||||||
Sbjct  1415616  GGGCTTAGTGCCGCAGCTAACGCATTAAGC  1415645
```

Range 2: 1420670 to 1421479

Score	Expect	Identities	Gaps	Strand	Frame
1496 bits(810)	0.0()	810/810(100%)	0/810(0%)	Plus/Plus	

Features:

```
Query  1        TGTTTGGTGTTTACACTAGACTGATGAGTTGCGAACGGGTGAGTAACGCGTAGGTAACCT  60
                ||||||||||||||||||||||||||||||||||||||||||||||||||||||||||||
Sbjct  1420670  TGTTTGGTGTTTACACTAGACTGATGAGTTGCGAACGGGTGAGTAACGCGTAGGTAACCT  1420729
Query  61       GCCTCATAGCGGGGGATAACTATTGGAAACGATAGCTAATACCGCATAAGAGTAATTAAC  120
                ||||||||||||||||||||||||||||||||||||||||||||||||||||||||||||
Sbjct  1420730  GCCTCATAGCGGGGGATAACTATTGGAAACGATAGCTAATACCGCATAAGAGTAATTAAC  1420789
Query  121      ACATGTTAGTTATTTAAAAGGAGCAATTGCTTCACTGTGAGATGGACCTGCGTTGTATTA  180
                ||||||||||||||||||||||||||||||||||||||||||||||||||||||||||||
Sbjct  1420790  ACATGTTAGTTATTTAAAAGGAGCAATTGCTTCACTGTGAGATGGACCTGCGTTGTATTA  1420849
Query  181      GCTAGTTGGTGAGGTAAAGGCTCACCAAGGCGACGATACATAGCCGACCTGAGAGGGTGA  240
                ||||||||||||||||||||||||||||||||||||||||||||||||||||||||||||
Sbjct  1420850  GCTAGTTGGTGAGGTAAAGGCTCACCAAGGCGACGATACATAGCCGACCTGAGAGGGTGA  1420909
Query  241      TCGGCCACACTGGGACTGAGACACGGCCCAGACTCCTACGGGAGGCAGCAGTAGGGAATC  300
                ||||||||||||||||||||||||||||||||||||||||||||||||||||||||||||
Sbjct  1420910  TCGGCCACACTGGGACTGAGACACGGCCCAGACTCCTACGGGAGGCAGCAGTAGGGAATC  1420969
Query  301      TTCGGCAATGGACGGAAGTCTGACCGAGCAACGCCGCGTGAGTGAAGAAGGTTTTCGGAT  360
                ||||||||||||||||||||||||||||||||||||||||||||||||||||||||||||
Sbjct  1420970  TTCGGCAATGGACGGAAGTCTGACCGAGCAACGCCGCGTGAGTGAAGAAGGTTTTCGGAT  1421029
Query  361      CGTAAAGCTCTGTTGTTAGAGAAGAACGTTGGTAGGAGTGGAAAATCTACCAAGTGACGG  420
                ||||||||||||||||||||||||||||||||||||||||||||||||||||||||||||
Sbjct  1421030  CGTAAAGCTCTGTTGTTAGAGAAGAACGTTGGTAGGAGTGGAAAATCTACCAAGTGACGG  1421089
Query  421      TAACTAACCAGAAAGGGACGGCTAACTACGTGCCAGCAGCCGCGGTAATACGTAGGTCCC  480
                ||||||||||||||||||||||||||||||||||||||||||||||||||||||||||||
Sbjct  1421090  TAACTAACCAGAAAGGGACGGCTAACTACGTGCCAGCAGCCGCGGTAATACGTAGGTCCC  1421149
Query  481      GAGCGTTGTCCGGATTTATTGGGCGTAAAGCGAGCGCAGGCGGTTCTTTAAGTCTGAAGT  540
                ||||||||||||||||||||||||||||||||||||||||||||||||||||||||||||
Sbjct  1421150  GAGCGTTGTCCGGATTTATTGGGCGTAAAGCGAGCGCAGGCGGTTCTTTAAGTCTGAAGT  1421209
Query  541      TAAAGGCAGTGGCTTAACCATTGTACGCTTTGGAAACTGGAGGACTTGAGTGCAGAAGGG  600
                ||||||||||||||||||||||||||||||||||||||||||||||||||||||||||||
Sbjct  1421210  TAAAGGCAGTGGCTTAACCATTGTACGCTTTGGAAACTGGAGGACTTGAGTGCAGAAGGG  1421269
Query  601      GAGAGTGGAATTCCATGTGTAGCGGTGAAATGCGTAGATATATGGAGGAACACCGGTGGC  660
                ||||||||||||||||||||||||||||||||||||||||||||||||||||||||||||
Sbjct  1421270  GAGAGTGGAATTCCATGTGTAGCGGTGAAATGCGTAGATATATGGAGGAACACCGGTGGC  1421329
Query  661      GAAAGCGGCTCTCTGGTCTGTAACTGACGCTGAGGCTCGAAAGCGTGGGGAGCAAACAGG  720
                ||||||||||||||||||||||||||||||||||||||||||||||||||||||||||||
Sbjct  1421330  GAAAGCGGCTCTCTGGTCTGTAACTGACGCTGAGGCTCGAAAGCGTGGGGAGCAAACAGG  1421389
Query  721      ATTAGATACCCTGGTAGTCCACGCCGTAAACGATGAGTGCTAGGTGTTAGGCCCTTTCCG  780
                ||||||||||||||||||||||||||||||||||||||||||||||||||||||||||||
Sbjct  1421390  ATTAGATACCCTGGTAGTCCACGCCGTAAACGATGAGTGCTAGGTGTTAGGCCCTTTCCG  1421449
Query  781      GGGCTTAGTGCCGCAGCTAACGCATTAAGC  810
                ||||||||||||||||||||||||||||||
Sbjct  1421450  GGGCTTAGTGCCGCAGCTAACGCATTAAGC  1421479
```

Range 3: 1489474 to 1490283

Score	Expect	Identities	Gaps	Strand	Frame
1496 bits(810)	0.0()	810/810(100%)	0/810(0%)	Plus/Plus	

Features:

```
Query  1        TGTTTGGTGTTTACACTAGACTGATGAGTTGCGAACGGGTGAGTAACGCGTAGGTAACCT  60
                ||||||||||||||||||||||||||||||||||||||||||||||||||||||||||||
Sbjct  1489474  TGTTTGGTGTTTACACTAGACTGATGAGTTGCGAACGGGTGAGTAACGCGTAGGTAACCT  1489533

Query  61       GCCTCATAGCGGGGGATAACTATTGGAAACGATAGCTAATACCGCATAAGAGTAATTAAC  120
                ||||||||||||||||||||||||||||||||||||||||||||||||||||||||||||
Sbjct  1489534  GCCTCATAGCGGGGGATAACTATTGGAAACGATAGCTAATACCGCATAAGAGTAATTAAC  1489593

Query  121      ACATGTTAGTTATTTAAAAGGAGCAATTGCTTCACTGTGAGATGGACCTGCGTTGTATTA  180
                ||||||||||||||||||||||||||||||||||||||||||||||||||||||||||||
Sbjct  1489594  ACATGTTAGTTATTTAAAAGGAGCAATTGCTTCACTGTGAGATGGACCTGCGTTGTATTA  1489653

Query  181      GCTAGTTGGTGAGGTAAAGGCTCACCAAGGCGACGATACATAGCCGACCTGAGAGGGTGA  240
                ||||||||||||||||||||||||||||||||||||||||||||||||||||||||||||
Sbjct  1489654  GCTAGTTGGTGAGGTAAAGGCTCACCAAGGCGACGATACATAGCCGACCTGAGAGGGTGA  1489713

Query  241      TCGGCCACACTGGGACTGAGACACGGCCCAGACTCCTACGGGAGGCAGCAGTAGGGAATC  300
                ||||||||||||||||||||||||||||||||||||||||||||||||||||||||||||
Sbjct  1489714  TCGGCCACACTGGGACTGAGACACGGCCCAGACTCCTACGGGAGGCAGCAGTAGGGAATC  1489773

Query  301      TTCGGCAATGGACGGAAGTCTGACCGAGCAACGCCGCGTGAGTGAAGAAGGTTTTCGGAT  360
                ||||||||||||||||||||||||||||||||||||||||||||||||||||||||||||
Sbjct  1489774  TTCGGCAATGGACGGAAGTCTGACCGAGCAACGCCGCGTGAGTGAAGAAGGTTTTCGGAT  1489833

Query  361      CGTAAAGCTCTGTTGTTAGAGAAGAACGTTGGTAGGAGTGGAAAATCTACCAAGTGACGG  420
                ||||||||||||||||||||||||||||||||||||||||||||||||||||||||||||
Sbjct  1489834  CGTAAAGCTCTGTTGTTAGAGAAGAACGTTGGTAGGAGTGGAAAATCTACCAAGTGACGG  1489893

Query  421      TAACTAACCAGAAAGGGACGGCTAACTACGTGCCAGCAGCCGCGGTAATACGTAGGTCCC  480
                ||||||||||||||||||||||||||||||||||||||||||||||||||||||||||||
Sbjct  1489894  TAACTAACCAGAAAGGGACGGCTAACTACGTGCCAGCAGCCGCGGTAATACGTAGGTCCC  1489953

Query  481      GAGCGTTGTCCGGATTTATTGGGCGTAAAGCGAGCGCAGGCGGTTCTTTAAGTCTGAAGT  540
                ||||||||||||||||||||||||||||||||||||||||||||||||||||||||||||
Sbjct  1489954  GAGCGTTGTCCGGATTTATTGGGCGTAAAGCGAGCGCAGGCGGTTCTTTAAGTCTGAAGT  1490013

Query  541      TAAAGGCAGTGGCTTAACCATTGTACGCTTTGGAAACTGGAGGACTTGAGTGCAGAAGGG  600
                ||||||||||||||||||||||||||||||||||||||||||||||||||||||||||||
Sbjct  1490014  TAAAGGCAGTGGCTTAACCATTGTACGCTTTGGAAACTGGAGGACTTGAGTGCAGAAGGG  1490073

Query  601      GAGAGTGGAATTCCATGTGTAGCGGTGAAATGCGTAGATATATGGAGGAACACCGGTGGC  660
                ||||||||||||||||||||||||||||||||||||||||||||||||||||||||||||
Sbjct  1490074  GAGAGTGGAATTCCATGTGTAGCGGTGAAATGCGTAGATATATGGAGGAACACCGGTGGC  1490133

Query  661      GAAAGCGGCTCTCTGGTCTGTAACTGACGCTGAGGCTCGAAAGCGTGGGGAGCAAACAGG  720
                ||||||||||||||||||||||||||||||||||||||||||||||||||||||||||||
Sbjct  1490134  GAAAGCGGCTCTCTGGTCTGTAACTGACGCTGAGGCTCGAAAGCGTGGGGAGCAAACAGG  1490193

Query  721      ATTAGATACCCTGGTAGTCCACGCCGTAAACGATGAGTGCTAGGTGTTAGGCCCTTTCCG  780
                ||||||||||||||||||||||||||||||||||||||||||||||||||||||||||||
Sbjct  1490194  ATTAGATACCCTGGTAGTCCACGCCGTAAACGATGAGTGCTAGGTGTTAGGCCCTTTCCG  1490253

Query  781      GGGCTTAGTGCCGCAGCTAACGCATTAAGC  810
                ||||||||||||||||||||||||||||||
Sbjct  1490254  GGGCTTAGTGCCGCAGCTAACGCATTAAGC  1490283
```

Range 4: 1563504 to 1564313

Score	Expect	Identities	Gaps	Strand	Frame
1496 bits(810)	0.0()	810/810(100%)	0/810(0%)	Plus/Plus	

Features:

```
Query  1        TGTTTGGTGTTTACACTAGACTGATGAGTTGCGAACGGGTGAGTAACGCGTAGGTAACCT  60
                ||||||||||||||||||||||||||||||||||||||||||||||||||||||||||||
Sbjct  1563504  TGTTTGGTGTTTACACTAGACTGATGAGTTGCGAACGGGTGAGTAACGCGTAGGTAACCT  1563563

Query  61       GCCTCATAGCGGGGGATAACTATTGGAAACGATAGCTAATACCGCATAAGAGTAATTAAC  120
                ||||||||||||||||||||||||||||||||||||||||||||||||||||||||||||
Sbjct  1563564  GCCTCATAGCGGGGGATAACTATTGGAAACGATAGCTAATACCGCATAAGAGTAATTAAC  1563623

Query  121      ACATGTTAGTTATTTAAAAGGAGCAATTGCTTCACTGTGAGATGGACCTGCGTTGTATTA  180
                ||||||||||||||||||||||||||||||||||||||||||||||||||||||||||||
Sbjct  1563624  ACATGTTAGTTATTTAAAAGGAGCAATTGCTTCACTGTGAGATGGACCTGCGTTGTATTA  1563683

Query  181      GCTAGTTGGTGAGGTAAAGGCTCACCAAGGCGACGATACATAGCCGACCTGAGAGGGTGA  240
                ||||||||||||||||||||||||||||||||||||||||||||||||||||||||||||
Sbjct  1563684  GCTAGTTGGTGAGGTAAAGGCTCACCAAGGCGACGATACATAGCCGACCTGAGAGGGTGA  1563743

Query  241      TCGGCCACACTGGGACTGAGACACGGCCCAGACTCCTACGGGAGGCAGCAGTAGGGAATC  300
                ||||||||||||||||||||||||||||||||||||||||||||||||||||||||||||
Sbjct  1563744  TCGGCCACACTGGGACTGAGACACGGCCCAGACTCCTACGGGAGGCAGCAGTAGGGAATC  1563803

Query  301      TTCGGCAATGGACGGAAGTCTGACCGAGCAACGCCGCGTGAGTGAAGAAGGTTTTCGGAT  360
                ||||||||||||||||||||||||||||||||||||||||||||||||||||||||||||
Sbjct  1563804  TTCGGCAATGGACGGAAGTCTGACCGAGCAACGCCGCGTGAGTGAAGAAGGTTTTCGGAT  1563863

Query  361      CGTAAAGCTCTGTTGTTAGAGAAGAACGTTGGTAGGAGTGGAAAATCTACCAAGTGACGG  420
                ||||||||||||||||||||||||||||||||||||||||||||||||||||||||||||
Sbjct  1563864  CGTAAAGCTCTGTTGTTAGAGAAGAACGTTGGTAGGAGTGGAAAATCTACCAAGTGACGG  1563923

Query  421      TAACTAACCAGAAAGGGACGGCTAACTACGTGCCAGCAGCCGCGGTAATACGTAGGTCCC  480
                ||||||||||||||||||||||||||||||||||||||||||||||||||||||||||||
Sbjct  1563924  TAACTAACCAGAAAGGGACGGCTAACTACGTGCCAGCAGCCGCGGTAATACGTAGGTCCC  1563983

Query  481      GAGCGTTGTCCGGATTTATTGGGCGTAAAGCGAGCGCAGGCGGTTCTTTAAGTCTGAAGT  540
                ||||||||||||||||||||||||||||||||||||||||||||||||||||||||||||
Sbjct  1563984  GAGCGTTGTCCGGATTTATTGGGCGTAAAGCGAGCGCAGGCGGTTCTTTAAGTCTGAAGT  1564043

Query  541      TAAAGGCAGTGGCTTAACCATTGTACGCTTTGGAAACTGGAGGACTTGAGTGCAGAAGGG  600
                ||||||||||||||||||||||||||||||||||||||||||||||||||||||||||||
Sbjct  1564044  TAAAGGCAGTGGCTTAACCATTGTACGCTTTGGAAACTGGAGGACTTGAGTGCAGAAGGG  1564103

Query  601      GAGAGTGGAATTCCATGTGTAGCGGTGAAATGCGTAGATATATGGAGGAACACCGGTGGC  660
                ||||||||||||||||||||||||||||||||||||||||||||||||||||||||||||
Sbjct  1564104  GAGAGTGGAATTCCATGTGTAGCGGTGAAATGCGTAGATATATGGAGGAACACCGGTGGC  1564163

Query  661      GAAAGCGGCTCTCTGGTCTGTAACTGACGCTGAGGCTCGAAAGCGTGGGGAGCAAACAGG  720
                ||||||||||||||||||||||||||||||||||||||||||||||||||||||||||||
Sbjct  1564164  GAAAGCGGCTCTCTGGTCTGTAACTGACGCTGAGGCTCGAAAGCGTGGGGAGCAAACAGG  1564223

Query  721      ATTAGATACCCTGGTAGTCCACGCCGTAAACGATGAGTGCTAGGTGTTAGGCCCTTTCCG  780
                ||||||||||||||||||||||||||||||||||||||||||||||||||||||||||||
Sbjct  1564224  ATTAGATACCCTGGTAGTCCACGCCGTAAACGATGAGTGCTAGGTGTTAGGCCCTTTCCG  1564283

Query  781      GGGCTTAGTGCCGCAGCTAACGCATTAAGC  810
                ||||||||||||||||||||||||||||||
Sbjct  1564284  GGGCTTAGTGCCGCAGCTAACGCATTAAGC  1564313
```

Range 5: 1671615 to 1672424

Score	Expect	Identities	Gaps	Strand	Frame
1496 bits(810)	0.0()	810/810(100%)	0/810(0%)	Plus/Plus	

Features:

```
Query  1        TGTTTGGTGTTTACACTAGACTGATGAGTTGCGAACGGGTGAGTAACGCGTAGGTAACCT  60
                ||||||||||||||||||||||||||||||||||||||||||||||||||||||||||||
Sbjct  1671615  TGTTTGGTGTTTACACTAGACTGATGAGTTGCGAACGGGTGAGTAACGCGTAGGTAACCT  1671674

Query  61       GCCTCATAGCGGGGGATAACTATTGGAAACGATAGCTAATACCGCATAAGAGTAATTAAC  120
                ||||||||||||||||||||||||||||||||||||||||||||||||||||||||||||
Sbjct  1671675  GCCTCATAGCGGGGGATAACTATTGGAAACGATAGCTAATACCGCATAAGAGTAATTAAC  1671734

Query  121      ACATGTTAGTTATTTAAAAGGAGCAATTGCTTCACTGTGAGATGGACCTGCGTTGTATTA  180
                ||||||||||||||||||||||||||||||||||||||||||||||||||||||||||||
Sbjct  1671735  ACATGTTAGTTATTTAAAAGGAGCAATTGCTTCACTGTGAGATGGACCTGCGTTGTATTA  1671794

Query  181      GCTAGTTGGTGAGGTAAAGGCTCACCAAGGCGACGATACATAGCCGACCTGAGAGGGTGA  240
                ||||||||||||||||||||||||||||||||||||||||||||||||||||||||||||
Sbjct  1671795  GCTAGTTGGTGAGGTAAAGGCTCACCAAGGCGACGATACATAGCCGACCTGAGAGGGTGA  1671854

Query  241      TCGGCCACACTGGGACTGAGACACGGCCCAGACTCCTACGGGAGGCAGCAGTAGGGAATC  300
                ||||||||||||||||||||||||||||||||||||||||||||||||||||||||||||
Sbjct  1671855  TCGGCCACACTGGGACTGAGACACGGCCCAGACTCCTACGGGAGGCAGCAGTAGGGAATC  1671914

Query  301      TTCGGCAATGGACGGAAGTCTGACCGAGCAACGCCGCGTGAGTGAAGAAGGTTTTCGGAT  360
```

```
                ||||||||||||||||||||||||||||||||||||||||||||||||||||||||||||
Sbjct  1671915  TTCGGCAATGGACGGAAGTCTGACCGAGCAACGCCGCGTGAGTGAAGAAGGTTTTCGGAT  1671974
Query  361      CGTAAAGCTCTGTTGTTAGAGAAGAACGTTGGTAGGAGTGGAAAATCTACCAAGTGACGG  420
                ||||||||||||||||||||||||||||||||||||||||||||||||||||||||||||
Sbjct  1671975  CGTAAAGCTCTGTTGTTAGAGAAGAACGTTGGTAGGAGTGGAAAATCTACCAAGTGACGG  1672034
Query  421      TAACTAACCAGAAAGGGACGGCTAACTACGTGCCAGCAGCCGCGGTAATACGTAGGTCCC  480
                ||||||||||||||||||||||||||||||||||||||||||||||||||||||||||||
Sbjct  1672035  TAACTAACCAGAAAGGGACGGCTAACTACGTGCCAGCAGCCGCGGTAATACGTAGGTCCC  1672094
Query  481      GAGCGTTGTCCGGATTTATTGGGCGTAAAGCGAGCGCAGGCGGTTCTTTAAGTCTGAAGT  540
                ||||||||||||||||||||||||||||||||||||||||||||||||||||||||||||
Sbjct  1672095  GAGCGTTGTCCGGATTTATTGGGCGTAAAGCGAGCGCAGGCGGTTCTTTAAGTCTGAAGT  1672154
Query  541      TAAAGGCAGTGGCTTAACCATTGTACGCTTTGGAAACTGGAGGACTTGAGTGCAGAAGGG  600
                ||||||||||||||||||||||||||||||||||||||||||||||||||||||||||||
Sbjct  1672155  TAAAGGCAGTGGCTTAACCATTGTACGCTTTGGAAACTGGAGGACTTGAGTGCAGAAGGG  1672214
Query  601      GAGAGTGGAATTCCATGTGTAGCGGTGAAATGCGTAGATATATGGAGGAACACCGGTGGC  660
                ||||||||||||||||||||||||||||||||||||||||||||||||||||||||||||
Sbjct  1672215  GAGAGTGGAATTCCATGTGTAGCGGTGAAATGCGTAGATATATGGAGGAACACCGGTGGC  1672274
Query  661      GAAAGCGGCTCTCTGGTCTGTAACTGACGCTGAGGCTCGAAAGCGTGGGGAGCAAACAGG  720
                ||||||||||||||||||||||||||||||||||||||||||||||||||||||||||||
Sbjct  1672275  GAAAGCGGCTCTCTGGTCTGTAACTGACGCTGAGGCTCGAAAGCGTGGGGAGCAAACAGG  1672334
Query  721      ATTAGATACCCTGGTAGTCCACGCCGTAAACGATGAGTGCTAGGTGTTAGGCCCTTTCCG  780
                ||||||||||||||||||||||||||||||||||||||||||||||||||||||||||||
Sbjct  1672335  ATTAGATACCCTGGTAGTCCACGCCGTAAACGATGAGTGCTAGGTGTTAGGCCCTTTCCG  1672394
Query  781      GGGCTTAGTGCCGCAGCTAACGCATTAAGC  810
                ||||||||||||||||||||||||||||||
Sbjct  1672395  GGGCTTAGTGCCGCAGCTAACGCATTAAGC  1672424
```

Range 6: 1768954 to 1769763

Score	Expect	Identities	Gaps	Strand	Frame
1496 bits(810)	0.0()	810/810(100%)	0/810(0%)	Plus/Plus	

Features:

```
Query  1        TGTTTGGTGTTTACACTAGACTGATGAGTTGCGAACGGGTGAGTAACGCGTAGGTAACCT  60
                ||||||||||||||||||||||||||||||||||||||||||||||||||||||||||||
Sbjct  1768954  TGTTTGGTGTTTACACTAGACTGATGAGTTGCGAACGGGTGAGTAACGCGTAGGTAACCT  1769013
Query  61       GCCTCATAGCGGGGGATAACTATTGGAAACGATAGCTAATACCGCATAAGAGTAATTAAC  120
                ||||||||||||||||||||||||||||||||||||||||||||||||||||||||||||
Sbjct  1769014  GCCTCATAGCGGGGGATAACTATTGGAAACGATAGCTAATACCGCATAAGAGTAATTAAC  1769073
Query  121      ACATGTTAGTTATTTAAAAGGAGCAATTGCTTCACTGTGAGATGGACCTGCGTTGTATTA  180
                ||||||||||||||||||||||||||||||||||||||||||||||||||||||||||||
Sbjct  1769074  ACATGTTAGTTATTTAAAAGGAGCAATTGCTTCACTGTGAGATGGACCTGCGTTGTATTA  1769133
Query  181      GCTAGTTGGTGAGGTAAAGGCTCACCAAGGCGACGATACATAGCCGACCTGAGAGGGTGA  240
                ||||||||||||||||||||||||||||||||||||||||||||||||||||||||||||
Sbjct  1769134  GCTAGTTGGTGAGGTAAAGGCTCACCAAGGCGACGATACATAGCCGACCTGAGAGGGTGA  1769193
Query  241      TCGGCCACACTGGGACTGAGACACGGCCCAGACTCCTACGGGAGGCAGCAGTAGGGAATC  300
                ||||||||||||||||||||||||||||||||||||||||||||||||||||||||||||
Sbjct  1769194  TCGGCCACACTGGGACTGAGACACGGCCCAGACTCCTACGGGAGGCAGCAGTAGGGAATC  1769253
Query  301      TTCGGCAATGGACGGAAGTCTGACCGAGCAACGCCGCGTGAGTGAAGAAGGTTTTCGGAT  360
                ||||||||||||||||||||||||||||||||||||||||||||||||||||||||||||
Sbjct  1769254  TTCGGCAATGGACGGAAGTCTGACCGAGCAACGCCGCGTGAGTGAAGAAGGTTTTCGGAT  1769313
Query  361      CGTAAAGCTCTGTTGTTAGAGAAGAACGTTGGTAGGAGTGGAAAATCTACCAAGTGACGG  420
                ||||||||||||||||||||||||||||||||||||||||||||||||||||||||||||
Sbjct  1769314  CGTAAAGCTCTGTTGTTAGAGAAGAACGTTGGTAGGAGTGGAAAATCTACCAAGTGACGG  1769373
Query  421      TAACTAACCAGAAAGGGACGGCTAACTACGTGCCAGCAGCCGCGGTAATACGTAGGTCCC  480
                ||||||||||||||||||||||||||||||||||||||||||||||||||||||||||||
Sbjct  1769374  TAACTAACCAGAAAGGGACGGCTAACTACGTGCCAGCAGCCGCGGTAATACGTAGGTCCC  1769433
Query  481      GAGCGTTGTCCGGATTTATTGGGCGTAAAGCGAGCGCAGGCGGTTCTTTAAGTCTGAAGT  540
                ||||||||||||||||||||||||||||||||||||||||||||||||||||||||||||
Sbjct  1769434  GAGCGTTGTCCGGATTTATTGGGCGTAAAGCGAGCGCAGGCGGTTCTTTAAGTCTGAAGT  1769493
Query  541      TAAAGGCAGTGGCTTAACCATTGTACGCTTTGGAAACTGGAGGACTTGAGTGCAGAAGGG  600
                ||||||||||||||||||||||||||||||||||||||||||||||||||||||||||||
Sbjct  1769494  TAAAGGCAGTGGCTTAACCATTGTACGCTTTGGAAACTGGAGGACTTGAGTGCAGAAGGG  1769553
Query  601      GAGAGTGGAATTCCATGTGTAGCGGTGAAATGCGTAGATATATGGAGGAACACCGGTGGC  660
                ||||||||||||||||||||||||||||||||||||||||||||||||||||||||||||
Sbjct  1769554  GAGAGTGGAATTCCATGTGTAGCGGTGAAATGCGTAGATATATGGAGGAACACCGGTGGC  1769613
Query  661      GAAAGCGGCTCTCTGGTCTGTAACTGACGCTGAGGCTCGAAAGCGTGGGGAGCAAACAGG  720
                ||||||||||||||||||||||||||||||||||||||||||||||||||||||||||||
Sbjct  1769614  GAAAGCGGCTCTCTGGTCTGTAACTGACGCTGAGGCTCGAAAGCGTGGGGAGCAAACAGG  1769673
Query  721      ATTAGATACCCTGGTAGTCCACGCCGTAAACGATGAGTGCTAGGTGTTAGGCCCTTTCCG  780
                ||||||||||||||||||||||||||||||||||||||||||||||||||||||||||||
Sbjct  1769674  ATTAGATACCCTGGTAGTCCACGCCGTAAACGATGAGTGCTAGGTGTTAGGCCCTTTCCG  1769733
Query  781      GGGCTTAGTGCCGCAGCTAACGCATTAAGC  810
                ||||||||||||||||||||||||||||||
Sbjct  1769734  GGGCTTAGTGCCGCAGCTAACGCATTAAGC  1769763
```

Range 7: 1838097 to 1838906

Score	Expect	Identities	Gaps	Strand	Frame
1496 bits(810)	0.0()	810/810(100%)	0/810(0%)	Plus/Plus	

Features:

```
Query  1        TGTTTGGTGTTTACACTAGACTGATGAGTTGCGAACGGGTGAGTAACGCGTAGGTAACCT  60
                ||||||||||||||||||||||||||||||||||||||||||||||||||||||||||||
Sbjct  1838097  TGTTTGGTGTTTACACTAGACTGATGAGTTGCGAACGGGTGAGTAACGCGTAGGTAACCT  1838156
Query  61       GCCTCATAGCGGGGGATAACTATTGGAAACGATAGCTAATACCGCATAAGAGTAATTAAC  120
                ||||||||||||||||||||||||||||||||||||||||||||||||||||||||||||
Sbjct  1838157  GCCTCATAGCGGGGGATAACTATTGGAAACGATAGCTAATACCGCATAAGAGTAATTAAC  1838216
Query  121      ACATGTTAGTTATTTAAAAGGAGCAATTGCTTCACTGTGAGATGGACCTGCGTTGTATTA  180
                ||||||||||||||||||||||||||||||||||||||||||||||||||||||||||||
Sbjct  1838217  ACATGTTAGTTATTTAAAAGGAGCAATTGCTTCACTGTGAGATGGACCTGCGTTGTATTA  1838276
Query  181      GCTAGTTGGTGAGGTAAAGGCTCACCAAGGCGACGATACATAGCCGACCTGAGAGGGTGA  240
                ||||||||||||||||||||||||||||||||||||||||||||||||||||||||||||
Sbjct  1838277  GCTAGTTGGTGAGGTAAAGGCTCACCAAGGCGACGATACATAGCCGACCTGAGAGGGTGA  1838336
Query  241      TCGGCCACACTGGGACTGAGACACGGCCCAGACTCCTACGGGAGGCAGCAGTAGGGAATC  300
                ||||||||||||||||||||||||||||||||||||||||||||||||||||||||||||
Sbjct  1838337  TCGGCCACACTGGGACTGAGACACGGCCCAGACTCCTACGGGAGGCAGCAGTAGGGAATC  1838396
Query  301      TTCGGCAATGGACGGAAGTCTGACCGAGCAACGCCGCGTGAGTGAAGAAGGTTTTCGGAT  360
                ||||||||||||||||||||||||||||||||||||||||||||||||||||||||||||
Sbjct  1838397  TTCGGCAATGGACGGAAGTCTGACCGAGCAACGCCGCGTGAGTGAAGAAGGTTTTCGGAT  1838456
Query  361      CGTAAAGCTCTGTTGTTAGAGAAGAACGTTGGTAGGAGTGGAAAATCTACCAAGTGACGG  420
                ||||||||||||||||||||||||||||||||||||||||||||||||||||||||||||
Sbjct  1838457  CGTAAAGCTCTGTTGTTAGAGAAGAACGTTGGTAGGAGTGGAAAATCTACCAAGTGACGG  1838516
Query  421      TAACTAACCAGAAAGGGACGGCTAACTACGTGCCAGCAGCCGCGGTAATACGTAGGTCCC  480
                ||||||||||||||||||||||||||||||||||||||||||||||||||||||||||||
Sbjct  1838517  TAACTAACCAGAAAGGGACGGCTAACTACGTGCCAGCAGCCGCGGTAATACGTAGGTCCC  1838576
Query  481      GAGCGTTGTCCGGATTTATTGGGCGTAAAGCGAGCGCAGGCGGTTCTTTAAGTCTGAAGT  540
                ||||||||||||||||||||||||||||||||||||||||||||||||||||||||||||
Sbjct  1838577  GAGCGTTGTCCGGATTTATTGGGCGTAAAGCGAGCGCAGGCGGTTCTTTAAGTCTGAAGT  1838636
Query  541      TAAAGGCAGTGGCTTAACCATTGTACGCTTTGGAAACTGGAGGACTTGAGTGCAGAAGGG  600
                ||||||||||||||||||||||||||||||||||||||||||||||||||||||||||||
Sbjct  1838637  TAAAGGCAGTGGCTTAACCATTGTACGCTTTGGAAACTGGAGGACTTGAGTGCAGAAGGG  1838696
Query  601      GAGAGTGGAATTCCATGTGTAGCGGTGAAATGCGTAGATATATGGAGGAACACCGGTGGC  660
                ||||||||||||||||||||||||||||||||||||||||||||||||||||||||||||
Sbjct  1838697  GAGAGTGGAATTCCATGTGTAGCGGTGAAATGCGTAGATATATGGAGGAACACCGGTGGC  1838756
```

```
Query  661      GAAAGCGGCTCTCTGGTCTGTAACTGACGCTGAGGCTCGAAAGCGTGGGGAGCAAACAGG  720
                ||||||||||||||||||||||||||||||||||||||||||||||||||||||||||||
Sbjct  1838757  GAAAGCGGCTCTCTGGTCTGTAACTGACGCTGAGGCTCGAAAGCGTGGGGAGCAAACAGG  1838816

Query  721      ATTAGATACCCTGGTAGTCCACGCCGTAAACGATGAGTGCTAGGTGTTAGGCCCTTTCCG  780
                ||||||||||||||||||||||||||||||||||||||||||||||||||||||||||||
Sbjct  1838817  ATTAGATACCCTGGTAGTCCACGCCGTAAACGATGAGTGCTAGGTGTTAGGCCCTTTCCG  1838876

Query  781      GGGCTTAGTGCCGCAGCTAACGCATTAAGC  810
                ||||||||||||||||||||||||||||||
Sbjct  1838877  GGGCTTAGTGCCGCAGCTAACGCATTAAGC  1838906
```

Streptococcus agalactiae strain B507 chromosome, complete genome
Sequence ID: **CP021771.1** Length: 2082479 Number of Matches: 7
Range 1: 79709 to 80518

Score	Expect	Identities	Gaps	Strand	Frame
1496 bits(810)	0.0()	810/810(100%)	0/810(0%)	Plus/Plus	

Features:

```
Query  1      TGTTTGGTGTTTACACTAGACTGATGAGTTGCGAACGGGTGAGTAACGCGTAGGTAACCT  60
              ||||||||||||||||||||||||||||||||||||||||||||||||||||||||||||
Sbjct  79709  TGTTTGGTGTTTACACTAGACTGATGAGTTGCGAACGGGTGAGTAACGCGTAGGTAACCT  79768

Query  61     GCCTCATAGCGGGGGATAACTATTGGAAACGATAGCTAATACCGCATAAGAGTAATTAAC  120
              ||||||||||||||||||||||||||||||||||||||||||||||||||||||||||||
Sbjct  79769  GCCTCATAGCGGGGGATAACTATTGGAAACGATAGCTAATACCGCATAAGAGTAATTAAC  79828

Query  121    ACATGTTAGTTATTTAAAAGGAGCAATTGCTTCACTGTGAGATGGACCTGCGTTGTATTA  180
              ||||||||||||||||||||||||||||||||||||||||||||||||||||||||||||
Sbjct  79829  ACATGTTAGTTATTTAAAAGGAGCAATTGCTTCACTGTGAGATGGACCTGCGTTGTATTA  79888

Query  181    GCTAGTTGGTGAGGTAAAGGCTCACCAAGGCGACGATACATAGCCGACCTGAGAGGGTGA  240
              ||||||||||||||||||||||||||||||||||||||||||||||||||||||||||||
Sbjct  79889  GCTAGTTGGTGAGGTAAAGGCTCACCAAGGCGACGATACATAGCCGACCTGAGAGGGTGA  79948

Query  241    TCGGCCACACTGGGACTGAGACACGGCCCAGACTCCTACGGGAGGCAGCAGTAGGGAATC  300
              ||||||||||||||||||||||||||||||||||||||||||||||||||||||||||||
Sbjct  79949  TCGGCCACACTGGGACTGAGACACGGCCCAGACTCCTACGGGAGGCAGCAGTAGGGAATC  80008

Query  301    TTCGGCAATGGACGGAAGTCTGACCGAGCAACGCCGCGTGAGTGAAGAAGGTTTTCGGAT  360
              ||||||||||||||||||||||||||||||||||||||||||||||||||||||||||||
Sbjct  80009  TTCGGCAATGGACGGAAGTCTGACCGAGCAACGCCGCGTGAGTGAAGAAGGTTTTCGGAT  80068

Query  361    CGTAAAGCTCTGTTGTTAGAGAAGAACGTTGGTAGGAGTGGAAAATCTACCAAGTGACGG  420
              ||||||||||||||||||||||||||||||||||||||||||||||||||||||||||||
Sbjct  80069  CGTAAAGCTCTGTTGTTAGAGAAGAACGTTGGTAGGAGTGGAAAATCTACCAAGTGACGG  80128

Query  421    TAACTAACCAGAAAGGGACGGCTAACTACGTGCCAGCAGCCGCGGTAATACGTAGGTCCC  480
              ||||||||||||||||||||||||||||||||||||||||||||||||||||||||||||
Sbjct  80129  TAACTAACCAGAAAGGGACGGCTAACTACGTGCCAGCAGCCGCGGTAATACGTAGGTCCC  80188

Query  481    GAGCGTTGTCCGGATTTATTGGGCGTAAAGCGAGCGCAGGCGGTTCTTTAAGTCTGAAGT  540
              ||||||||||||||||||||||||||||||||||||||||||||||||||||||||||||
Sbjct  80189  GAGCGTTGTCCGGATTTATTGGGCGTAAAGCGAGCGCAGGCGGTTCTTTAAGTCTGAAGT  80248

Query  541    TAAAGGCAGTGGCTTAACCATTGTACGCTTTGGAAACTGGAGGACTTGAGTGCAGAAGGG  600
              ||||||||||||||||||||||||||||||||||||||||||||||||||||||||||||
Sbjct  80249  TAAAGGCAGTGGCTTAACCATTGTACGCTTTGGAAACTGGAGGACTTGAGTGCAGAAGGG  80308

Query  601    GAGAGTGGAATTCCATGTGTAGCGGTGAAATGCGTAGATATATGGAGGAACACCGGTGGC  660
              ||||||||||||||||||||||||||||||||||||||||||||||||||||||||||||
Sbjct  80309  GAGAGTGGAATTCCATGTGTAGCGGTGAAATGCGTAGATATATGGAGGAACACCGGTGGC  80368

Query  661    GAAAGCGGCTCTCTGGTCTGTAACTGACGCTGAGGCTCGAAAGCGTGGGGAGCAAACAGG  720
              ||||||||||||||||||||||||||||||||||||||||||||||||||||||||||||
Sbjct  80369  GAAAGCGGCTCTCTGGTCTGTAACTGACGCTGAGGCTCGAAAGCGTGGGGAGCAAACAGG  80428

Query  721    ATTAGATACCCTGGTAGTCCACGCCGTAAACGATGAGTGCTAGGTGTTAGGCCCTTTCCG  780
              ||||||||||||||||||||||||||||||||||||||||||||||||||||||||||||
Sbjct  80429  ATTAGATACCCTGGTAGTCCACGCCGTAAACGATGAGTGCTAGGTGTTAGGCCCTTTCCG  80488

Query  781    GGGCTTAGTGCCGCAGCTAACGCATTAAGC  810
              ||||||||||||||||||||||||||||||
Sbjct  80489  GGGCTTAGTGCCGCAGCTAACGCATTAAGC  80518
```

Range 2: 177051 to 177860

Score	Expect	Identities	Gaps	Strand	Frame
1496 bits(810)	0.0()	810/810(100%)	0/810(0%)	Plus/Plus	

Features:

```
Query  1       TGTTTGGTGTTTACACTAGACTGATGAGTTGCGAACGGGTGAGTAACGCGTAGGTAACCT  60
               ||||||||||||||||||||||||||||||||||||||||||||||||||||||||||||
Sbjct  177051  TGTTTGGTGTTTACACTAGACTGATGAGTTGCGAACGGGTGAGTAACGCGTAGGTAACCT  177110

Query  61      GCCTCATAGCGGGGGATAACTATTGGAAACGATAGCTAATACCGCATAAGAGTAATTAAC  120
               ||||||||||||||||||||||||||||||||||||||||||||||||||||||||||||
Sbjct  177111  GCCTCATAGCGGGGGATAACTATTGGAAACGATAGCTAATACCGCATAAGAGTAATTAAC  177170

Query  121     ACATGTTAGTTATTTAAAAGGAGCAATTGCTTCACTGTGAGATGGACCTGCGTTGTATTA  180
               ||||||||||||||||||||||||||||||||||||||||||||||||||||||||||||
Sbjct  177171  ACATGTTAGTTATTTAAAAGGAGCAATTGCTTCACTGTGAGATGGACCTGCGTTGTATTA  177230

Query  181     GCTAGTTGGTGAGGTAAAGGCTCACCAAGGCGACGATACATAGCCGACCTGAGAGGGTGA  240
               ||||||||||||||||||||||||||||||||||||||||||||||||||||||||||||
Sbjct  177231  GCTAGTTGGTGAGGTAAAGGCTCACCAAGGCGACGATACATAGCCGACCTGAGAGGGTGA  177290

Query  241     TCGGCCACACTGGGACTGAGACACGGCCCAGACTCCTACGGGAGGCAGCAGTAGGGAATC  300
               ||||||||||||||||||||||||||||||||||||||||||||||||||||||||||||
Sbjct  177291  TCGGCCACACTGGGACTGAGACACGGCCCAGACTCCTACGGGAGGCAGCAGTAGGGAATC  177350

Query  301     TTCGGCAATGGACGGAAGTCTGACCGAGCAACGCCGCGTGAGTGAAGAAGGTTTTCGGAT  360
               ||||||||||||||||||||||||||||||||||||||||||||||||||||||||||||
Sbjct  177351  TTCGGCAATGGACGGAAGTCTGACCGAGCAACGCCGCGTGAGTGAAGAAGGTTTTCGGAT  177410

Query  361     CGTAAAGCTCTGTTGTTAGAGAAGAACGTTGGTAGGAGTGGAAAATCTACCAAGTGACGG  420
               ||||||||||||||||||||||||||||||||||||||||||||||||||||||||||||
Sbjct  177411  CGTAAAGCTCTGTTGTTAGAGAAGAACGTTGGTAGGAGTGGAAAATCTACCAAGTGACGG  177470

Query  421     TAACTAACCAGAAAGGGACGGCTAACTACGTGCCAGCAGCCGCGGTAATACGTAGGTCCC  480
               ||||||||||||||||||||||||||||||||||||||||||||||||||||||||||||
Sbjct  177471  TAACTAACCAGAAAGGGACGGCTAACTACGTGCCAGCAGCCGCGGTAATACGTAGGTCCC  177530

Query  481     GAGCGTTGTCCGGATTTATTGGGCGTAAAGCGAGCGCAGGCGGTTCTTTAAGTCTGAAGT  540
               ||||||||||||||||||||||||||||||||||||||||||||||||||||||||||||
Sbjct  177531  GAGCGTTGTCCGGATTTATTGGGCGTAAAGCGAGCGCAGGCGGTTCTTTAAGTCTGAAGT  177590

Query  541     TAAAGGCAGTGGCTTAACCATTGTACGCTTTGGAAACTGGAGGACTTGAGTGCAGAAGGG  600
               ||||||||||||||||||||||||||||||||||||||||||||||||||||||||||||
Sbjct  177591  TAAAGGCAGTGGCTTAACCATTGTACGCTTTGGAAACTGGAGGACTTGAGTGCAGAAGGG  177650

Query  601     GAGAGTGGAATTCCATGTGTAGCGGTGAAATGCGTAGATATATGGAGGAACACCGGTGGC  660
               ||||||||||||||||||||||||||||||||||||||||||||||||||||||||||||
Sbjct  177651  GAGAGTGGAATTCCATGTGTAGCGGTGAAATGCGTAGATATATGGAGGAACACCGGTGGC  177710

Query  661     GAAAGCGGCTCTCTGGTCTGTAACTGACGCTGAGGCTCGAAAGCGTGGGGAGCAAACAGG  720
               ||||||||||||||||||||||||||||||||||||||||||||||||||||||||||||
Sbjct  177711  GAAAGCGGCTCTCTGGTCTGTAACTGACGCTGAGGCTCGAAAGCGTGGGGAGCAAACAGG  177770

Query  721     ATTAGATACCCTGGTAGTCCACGCCGTAAACGATGAGTGCTAGGTGTTAGGCCCTTTCCG  780
               ||||||||||||||||||||||||||||||||||||||||||||||||||||||||||||
Sbjct  177771  ATTAGATACCCTGGTAGTCCACGCCGTAAACGATGAGTGCTAGGTGTTAGGCCCTTTCCG  177830

Query  781     GGGCTTAGTGCCGCAGCTAACGCATTAAGC  810
               ||||||||||||||||||||||||||||||
Sbjct  177831  GGGCTTAGTGCCGCAGCTAACGCATTAAGC  177860
```

Range 3: 246195 to 247004

Score	Expect	Identities	Gaps	Strand	Frame

1496 bits(810)	0.0()	810/810(100%)	0/810(0%)	Plus/Plus	

Features:

```
Query  1       TGTTTGGTGTTTACACTAGACTGATGAGTTGCGAACGGGTGAGTAACGCGTAGGTAACCT  60
               ||||||||||||||||||||||||||||||||||||||||||||||||||||||||||||
Sbjct  246195  TGTTTGGTGTTTACACTAGACTGATGAGTTGCGAACGGGTGAGTAACGCGTAGGTAACCT  246254

Query  61      GCCTCATAGCGGGGGATAACTATTGGAAACGATAGCTAATACCGCATAAGAGTAATTAAC  120
               ||||||||||||||||||||||||||||||||||||||||||||||||||||||||||||
Sbjct  246255  GCCTCATAGCGGGGGATAACTATTGGAAACGATAGCTAATACCGCATAAGAGTAATTAAC  246314

Query  121     ACATGTTAGTTATTTAAAAGGAGCAATTGCTTCACTGTGAGATGGACCTGCGTTGTATTA  180
               ||||||||||||||||||||||||||||||||||||||||||||||||||||||||||||
Sbjct  246315  ACATGTTAGTTATTTAAAAGGAGCAATTGCTTCACTGTGAGATGGACCTGCGTTGTATTA  246374

Query  181     GCTAGTTGGTGAGGTAAAGGCTCACCAAGGCGACGATACATAGCCGACCTGAGAGGGTGA  240
               ||||||||||||||||||||||||||||||||||||||||||||||||||||||||||||
Sbjct  246375  GCTAGTTGGTGAGGTAAAGGCTCACCAAGGCGACGATACATAGCCGACCTGAGAGGGTGA  246434

Query  241     TCGGCCACACTGGGACTGAGACACGGCCCAGACTCCTACGGGAGGCAGCAGTAGGGAATC  300
               ||||||||||||||||||||||||||||||||||||||||||||||||||||||||||||
Sbjct  246435  TCGGCCACACTGGGACTGAGACACGGCCCAGACTCCTACGGGAGGCAGCAGTAGGGAATC  246494

Query  301     TTCGGCAATGGACGGAAGTCTGACCGAGCAACGCCGCGTGAGTGAAGAAGGTTTTCGGAT  360
               ||||||||||||||||||||||||||||||||||||||||||||||||||||||||||||
Sbjct  246495  TTCGGCAATGGACGGAAGTCTGACCGAGCAACGCCGCGTGAGTGAAGAAGGTTTTCGGAT  246554

Query  361     CGTAAAGCTCTGTTGTTAGAGAAGAACGTTGGTAGGAGTGGAAAATCTACCAAGTGACGG  420
               ||||||||||||||||||||||||||||||||||||||||||||||||||||||||||||
Sbjct  246555  CGTAAAGCTCTGTTGTTAGAGAAGAACGTTGGTAGGAGTGGAAAATCTACCAAGTGACGG  246614

Query  421     TAACTAACCAGAAAGGGACGGCTAACTACGTGCCAGCAGCCGCGGTAATACGTAGGTCCC  480
               ||||||||||||||||||||||||||||||||||||||||||||||||||||||||||||
Sbjct  246615  TAACTAACCAGAAAGGGACGGCTAACTACGTGCCAGCAGCCGCGGTAATACGTAGGTCCC  246674

Query  481     GAGCGTTGTCCGGATTTATTGGGCGTAAAGCGAGCGCAGGCGGTTCTTTAAGTCTGAAGT  540
               ||||||||||||||||||||||||||||||||||||||||||||||||||||||||||||
Sbjct  246675  GAGCGTTGTCCGGATTTATTGGGCGTAAAGCGAGCGCAGGCGGTTCTTTAAGTCTGAAGT  246734

Query  541     TAAAGGCAGTGGCTTAACCATTGTACGCTTTGGAAACTGGAGGACTTGAGTGCAGAAGGG  600
               ||||||||||||||||||||||||||||||||||||||||||||||||||||||||||||
Sbjct  246735  TAAAGGCAGTGGCTTAACCATTGTACGCTTTGGAAACTGGAGGACTTGAGTGCAGAAGGG  246794

Query  601     GAGAGTGGAATTCCATGTGTAGCGGTGAAATGCGTAGATATATGGAGGAACACCGGTGGC  660
               ||||||||||||||||||||||||||||||||||||||||||||||||||||||||||||
Sbjct  246795  GAGAGTGGAATTCCATGTGTAGCGGTGAAATGCGTAGATATATGGAGGAACACCGGTGGC  246854

Query  661     GAAAGCGGCTCTCTGGTCTGTAACTGACGCTGAGGCTCGAAAGCGTGGGGAGCAAACAGG  720
               ||||||||||||||||||||||||||||||||||||||||||||||||||||||||||||
Sbjct  246855  GAAAGCGGCTCTCTGGTCTGTAACTGACGCTGAGGCTCGAAAGCGTGGGGAGCAAACAGG  246914

Query  721     ATTAGATACCCTGGTAGTCCACGCCGTAAACGATGAGTGCTAGGTGTTAGGCCCTTTCCG  780
               ||||||||||||||||||||||||||||||||||||||||||||||||||||||||||||
Sbjct  246915  ATTAGATACCCTGGTAGTCCACGCCGTAAACGATGAGTGCTAGGTGTTAGGCCCTTTCCG  246974

Query  781     GGGCTTAGTGCCGCAGCTAACGCATTAAGC  810
               ||||||||||||||||||||||||||||||
Sbjct  246975  GGGCTTAGTGCCGCAGCTAACGCATTAAGC  247004
```

Range 4: 2066909 to 2067718

Score	Expect	Identities	Gaps	Strand	Frame
1496 bits(810)	0.0()	810/810(100%)	0/810(0%)	Plus/Plus	

Features:

```
Query  1        TGTTTGGTGTTTACACTAGACTGATGAGTTGCGAACGGGTGAGTAACGCGTAGGTAACCT  60
                ||||||||||||||||||||||||||||||||||||||||||||||||||||||||||||
Sbjct  2066909  TGTTTGGTGTTTACACTAGACTGATGAGTTGCGAACGGGTGAGTAACGCGTAGGTAACCT  2066968

Query  61       GCCTCATAGCGGGGGATAACTATTGGAAACGATAGCTAATACCGCATAAGAGTAATTAAC  120
                ||||||||||||||||||||||||||||||||||||||||||||||||||||||||||||
Sbjct  2066969  GCCTCATAGCGGGGGATAACTATTGGAAACGATAGCTAATACCGCATAAGAGTAATTAAC  2067028

Query  121      ACATGTTAGTTATTTAAAAGGAGCAATTGCTTCACTGTGAGATGGACCTGCGTTGTATTA  180
                ||||||||||||||||||||||||||||||||||||||||||||||||||||||||||||
Sbjct  2067029  ACATGTTAGTTATTTAAAAGGAGCAATTGCTTCACTGTGAGATGGACCTGCGTTGTATTA  2067088

Query  181      GCTAGTTGGTGAGGTAAAGGCTCACCAAGGCGACGATACATAGCCGACCTGAGAGGGTGA  240
                ||||||||||||||||||||||||||||||||||||||||||||||||||||||||||||
Sbjct  2067089  GCTAGTTGGTGAGGTAAAGGCTCACCAAGGCGACGATACATAGCCGACCTGAGAGGGTGA  2067148

Query  241      TCGGCCACACTGGGACTGAGACACGGCCCAGACTCCTACGGGAGGCAGCAGTAGGGAATC  300
                ||||||||||||||||||||||||||||||||||||||||||||||||||||||||||||
Sbjct  2067149  TCGGCCACACTGGGACTGAGACACGGCCCAGACTCCTACGGGAGGCAGCAGTAGGGAATC  2067208

Query  301      TTCGGCAATGGACGGAAGTCTGACCGAGCAACGCCGCGTGAGTGAAGAAGGTTTTCGGAT  360
                ||||||||||||||||||||||||||||||||||||||||||||||||||||||||||||
Sbjct  2067209  TTCGGCAATGGACGGAAGTCTGACCGAGCAACGCCGCGTGAGTGAAGAAGGTTTTCGGAT  2067268

Query  361      CGTAAAGCTCTGTTGTTAGAGAAGAACGTTGGTAGGAGTGGAAAATCTACCAAGTGACGG  420
                ||||||||||||||||||||||||||||||||||||||||||||||||||||||||||||
Sbjct  2067269  CGTAAAGCTCTGTTGTTAGAGAAGAACGTTGGTAGGAGTGGAAAATCTACCAAGTGACGG  2067328

Query  421      TAACTAACCAGAAAGGGACGGCTAACTACGTGCCAGCAGCCGCGGTAATACGTAGGTCCC  480
                ||||||||||||||||||||||||||||||||||||||||||||||||||||||||||||
Sbjct  2067329  TAACTAACCAGAAAGGGACGGCTAACTACGTGCCAGCAGCCGCGGTAATACGTAGGTCCC  2067388

Query  481      GAGCGTTGTCCGGATTTATTGGGCGTAAAGCGAGCGCAGGCGGTTCTTTAAGTCTGAAGT  540
                ||||||||||||||||||||||||||||||||||||||||||||||||||||||||||||
Sbjct  2067389  GAGCGTTGTCCGGATTTATTGGGCGTAAAGCGAGCGCAGGCGGTTCTTTAAGTCTGAAGT  2067448

Query  541      TAAAGGCAGTGGCTTAACCATTGTACGCTTTGGAAACTGGAGGACTTGAGTGCAGAAGGG  600
                ||||||||||||||||||||||||||||||||||||||||||||||||||||||||||||
Sbjct  2067449  TAAAGGCAGTGGCTTAACCATTGTACGCTTTGGAAACTGGAGGACTTGAGTGCAGAAGGG  2067508

Query  601      GAGAGTGGAATTCCATGTGTAGCGGTGAAATGCGTAGATATATGGAGGAACACCGGTGGC  660
                ||||||||||||||||||||||||||||||||||||||||||||||||||||||||||||
Sbjct  2067509  GAGAGTGGAATTCCATGTGTAGCGGTGAAATGCGTAGATATATGGAGGAACACCGGTGGC  2067568

Query  661      GAAAGCGGCTCTCTGGTCTGTAACTGACGCTGAGGCTCGAAAGCGTGGGGAGCAAACAGG  720
                ||||||||||||||||||||||||||||||||||||||||||||||||||||||||||||
Sbjct  2067569  GAAAGCGGCTCTCTGGTCTGTAACTGACGCTGAGGCTCGAAAGCGTGGGGAGCAAACAGG  2067628

Query  721      ATTAGATACCCTGGTAGTCCACGCCGTAAACGATGAGTGCTAGGTGTTAGGCCCTTTCCG  780
                ||||||||||||||||||||||||||||||||||||||||||||||||||||||||||||
Sbjct  2067629  ATTAGATACCCTGGTAGTCCACGCCGTAAACGATGAGTGCTAGGTGTTAGGCCCTTTCCG  2067688

Query  781      GGGCTTAGTGCCGCAGCTAACGCATTAAGC  810
                ||||||||||||||||||||||||||||||
Sbjct  2067689  GGGCTTAGTGCCGCAGCTAACGCATTAAGC  2067718
```

Range 5: 1899350 to 1900159

Score	Expect	Identities	Gaps	Strand	Frame
1491 bits(807)	0.0()	809/810(99%)	0/810(0%)	Plus/Plus	

Features:

```
Query  1        TGTTTGGTGTTTACACTAGACTGATGAGTTGCGAACGGGTGAGTAACGCGTAGGTAACCT  60
                ||||||||||||||||||||||||||||||||||||||||||||||||||||||||||||
Sbjct  1899350  TGTTTGGTGTTTACACTAGACTGATGAGTTGCGAACGGGTGAGTAACGCGTAGGTAACCT  1899409

Query  61       GCCTCATAGCGGGGGATAACTATTGGAAACGATAGCTAATACCGCATAAGAGTAATTAAC  120
                ||||||||||||||||||||||||||||||||||||||||||||||||||||| ||||||
Sbjct  1899410  GCCTCATAGCGGGGGATAACTATTGGAAACGATAGCTAATACCGCATAAGAGTGATTAAC  1899469

Query  121      ACATGTTAGTTATTTAAAAGGAGCAATTGCTTCACTGTGAGATGGACCTGCGTTGTATTA  180
                ||||||||||||||||||||||||||||||||||||||||||||||||||||||||||||
Sbjct  1899470  ACATGTTAGTTATTTAAAAGGAGCAATTGCTTCACTGTGAGATGGACCTGCGTTGTATTA  1899529

Query  181      GCTAGTTGGTGAGGTAAAGGCTCACCAAGGCGACGATACATAGCCGACCTGAGAGGGTGA  240
                ||||||||||||||||||||||||||||||||||||||||||||||||||||||||||||
Sbjct  1899530  GCTAGTTGGTGAGGTAAAGGCTCACCAAGGCGACGATACATAGCCGACCTGAGAGGGTGA  1899589

Query  241      TCGGCCACACTGGGACTGAGACACGGCCCAGACTCCTACGGGAGGCAGCAGTAGGGAATC  300
                ||||||||||||||||||||||||||||||||||||||||||||||||||||||||||||
```

```
Sbjct  1899590  TCGGCCACACTGGGACTGAGACACGGCCCAGACTCCTACGGGAGGCAGCAGTAGGGAATC  1899649
Query  301      TTCGGCAATGGACGGAAGTCTGACCGAGCAACGCCGCGTGAGTGAAGAAGGTTTTCGGAT  360
                ||||||||||||||||||||||||||||||||||||||||||||||||||||||||||||
Sbjct  1899650  TTCGGCAATGGACGGAAGTCTGACCGAGCAACGCCGCGTGAGTGAAGAAGGTTTTCGGAT  1899709
Query  361      CGTAAAGCTCTGTTGTTAGAGAAGAACGTTGGTAGGAGTGGAAAATCTACCAAGTGACGG  420
                ||||||||||||||||||||||||||||||||||||||||||||||||||||||||||||
Sbjct  1899710  CGTAAAGCTCTGTTGTTAGAGAAGAACGTTGGTAGGAGTGGAAAATCTACCAAGTGACGG  1899769
Query  421      TAACTAACCAGAAAGGGACGGCTAACTACGTGCCAGCAGCCGCGGTAATACGTAGGTCCC  480
                ||||||||||||||||||||||||||||||||||||||||||||||||||||||||||||
Sbjct  1899770  TAACTAACCAGAAAGGGACGGCTAACTACGTGCCAGCAGCCGCGGTAATACGTAGGTCCC  1899829
Query  481      GAGCGTTGTCCGGATTTATTGGGCGTAAAGCGAGCGCAGGCGGTTCTTTAAGTCTGAAGT  540
                ||||||||||||||||||||||||||||||||||||||||||||||||||||||||||||
Sbjct  1899830  GAGCGTTGTCCGGATTTATTGGGCGTAAAGCGAGCGCAGGCGGTTCTTTAAGTCTGAAGT  1899889
Query  541      TAAAGGCAGTGGCTTAACCATTGTACGCTTTGGAAACTGGAGGACTTGAGTGCAGAAGGG  600
                ||||||||||||||||||||||||||||||||||||||||||||||||||||||||||||
Sbjct  1899890  TAAAGGCAGTGGCTTAACCATTGTACGCTTTGGAAACTGGAGGACTTGAGTGCAGAAGGG  1899949
Query  601      GAGAGTGGAATTCCATGTGTAGCGGTGAAATGCGTAGATATATGGAGGAACACCGGTGGC  660
                ||||||||||||||||||||||||||||||||||||||||||||||||||||||||||||
Sbjct  1899950  GAGAGTGGAATTCCATGTGTAGCGGTGAAATGCGTAGATATATGGAGGAACACCGGTGGC  1900009
Query  661      GAAAGCGGCTCTCTGGTCTGTAACTGACGCTGAGGCTCGAAAGCGTGGGGAGCAAACAGG  720
                ||||||||||||||||||||||||||||||||||||||||||||||||||||||||||||
Sbjct  1900010  GAAAGCGGCTCTCTGGTCTGTAACTGACGCTGAGGCTCGAAAGCGTGGGGAGCAAACAGG  1900069
Query  721      ATTAGATACCCTGGTAGTCCACGCCGTAAACGATGAGTGCTAGGTGTTAGGCCCTTTCCG  780
                ||||||||||||||||||||||||||||||||||||||||||||||||||||||||||||
Sbjct  1900070  ATTAGATACCCTGGTAGTCCACGCCGTAAACGATGAGTGCTAGGTGTTAGGCCCTTTCCG  1900129
Query  781      GGGCTTAGTGCCGCAGCTAACGCATTAAGC  810
                ||||||||||||||||||||||||||||||
Sbjct  1900130  GGGCTTAGTGCCGCAGCTAACGCATTAAGC  1900159
```

Range 6: 1905182 to 1905991

Score	Expect	Identities	Gaps	Strand	Frame
1491 bits(807)	0.0()	809/810(99%)	0/810(0%)	Plus/Plus	

Features:

```
Query  1        TGTTTGGTGTTTACACTAGACTGATGAGTTGCGAACGGGTGAGTAACGCGTAGGTAACCT  60
                ||||||||||||||||||||||||||||||||||||||||||||||||||||||||||||
Sbjct  1905182  TGTTTGGTGTTTACACTAGACTGATGAGTTGCGAACGGGTGAGTAACGCGTAGGTAACCT  1905241
Query  61       GCCTCATAGCGGGGGATAACTATTGGAAACGATAGCTAATACCGCATAAGAGTAATTAAC  120
                ||||||||||||||||||||||||||||||||||||||||||||||||||||| ||||||
Sbjct  1905242  GCCTCATAGCGGGGGATAACTATTGGAAACGATAGCTAATACCGCATAAGAGTGATTAAC  1905301
Query  121      ACATGTTAGTTATTTAAAAGGAGCAATTGCTTCACTGTGAGATGGACCTGCGTTGTATTA  180
                ||||||||||||||||||||||||||||||||||||||||||||||||||||||||||||
Sbjct  1905302  ACATGTTAGTTATTTAAAAGGAGCAATTGCTTCACTGTGAGATGGACCTGCGTTGTATTA  1905361
Query  181      GCTAGTTGGTGAGGTAAAGGCTCACCAAGGCGACGATACATAGCCGACCTGAGAGGGTGA  240
                ||||||||||||||||||||||||||||||||||||||||||||||||||||||||||||
Sbjct  1905362  GCTAGTTGGTGAGGTAAAGGCTCACCAAGGCGACGATACATAGCCGACCTGAGAGGGTGA  1905421
Query  241      TCGGCCACACTGGGACTGAGACACGGCCCAGACTCCTACGGGAGGCAGCAGTAGGGAATC  300
                ||||||||||||||||||||||||||||||||||||||||||||||||||||||||||||
Sbjct  1905422  TCGGCCACACTGGGACTGAGACACGGCCCAGACTCCTACGGGAGGCAGCAGTAGGGAATC  1905481
Query  301      TTCGGCAATGGACGGAAGTCTGACCGAGCAACGCCGCGTGAGTGAAGAAGGTTTTCGGAT  360
                ||||||||||||||||||||||||||||||||||||||||||||||||||||||||||||
Sbjct  1905482  TTCGGCAATGGACGGAAGTCTGACCGAGCAACGCCGCGTGAGTGAAGAAGGTTTTCGGAT  1905541
Query  361      CGTAAAGCTCTGTTGTTAGAGAAGAACGTTGGTAGGAGTGGAAAATCTACCAAGTGACGG  420
                ||||||||||||||||||||||||||||||||||||||||||||||||||||||||||||
Sbjct  1905542  CGTAAAGCTCTGTTGTTAGAGAAGAACGTTGGTAGGAGTGGAAAATCTACCAAGTGACGG  1905601
Query  421      TAACTAACCAGAAAGGGACGGCTAACTACGTGCCAGCAGCCGCGGTAATACGTAGGTCCC  480
                ||||||||||||||||||||||||||||||||||||||||||||||||||||||||||||
Sbjct  1905602  TAACTAACCAGAAAGGGACGGCTAACTACGTGCCAGCAGCCGCGGTAATACGTAGGTCCC  1905661
Query  481      GAGCGTTGTCCGGATTTATTGGGCGTAAAGCGAGCGCAGGCGGTTCTTTAAGTCTGAAGT  540
                ||||||||||||||||||||||||||||||||||||||||||||||||||||||||||||
Sbjct  1905662  GAGCGTTGTCCGGATTTATTGGGCGTAAAGCGAGCGCAGGCGGTTCTTTAAGTCTGAAGT  1905721
Query  541      TAAAGGCAGTGGCTTAACCATTGTACGCTTTGGAAACTGGAGGACTTGAGTGCAGAAGGG  600
                ||||||||||||||||||||||||||||||||||||||||||||||||||||||||||||
Sbjct  1905722  TAAAGGCAGTGGCTTAACCATTGTACGCTTTGGAAACTGGAGGACTTGAGTGCAGAAGGG  1905781
Query  601      GAGAGTGGAATTCCATGTGTAGCGGTGAAATGCGTAGATATATGGAGGAACACCGGTGGC  660
                ||||||||||||||||||||||||||||||||||||||||||||||||||||||||||||
Sbjct  1905782  GAGAGTGGAATTCCATGTGTAGCGGTGAAATGCGTAGATATATGGAGGAACACCGGTGGC  1905841
Query  661      GAAAGCGGCTCTCTGGTCTGTAACTGACGCTGAGGCTCGAAAGCGTGGGGAGCAAACAGG  720
                ||||||||||||||||||||||||||||||||||||||||||||||||||||||||||||
Sbjct  1905842  GAAAGCGGCTCTCTGGTCTGTAACTGACGCTGAGGCTCGAAAGCGTGGGGAGCAAACAGG  1905901
Query  721      ATTAGATACCCTGGTAGTCCACGCCGTAAACGATGAGTGCTAGGTGTTAGGCCCTTTCCG  780
                ||||||||||||||||||||||||||||||||||||||||||||||||||||||||||||
Sbjct  1905902  ATTAGATACCCTGGTAGTCCACGCCGTAAACGATGAGTGCTAGGTGTTAGGCCCTTTCCG  1905961
Query  781      GGGCTTAGTGCCGCAGCTAACGCATTAAGC  810
                ||||||||||||||||||||||||||||||
Sbjct  1905962  GGGCTTAGTGCCGCAGCTAACGCATTAAGC  1905991
```

Range 7: 1973838 to 1974647

Score	Expect	Identities	Gaps	Strand	Frame
1491 bits(807)	0.0()	809/810(99%)	0/810(0%)	Plus/Plus	

Features:

```
Query  1        TGTTTGGTGTTTACACTAGACTGATGAGTTGCGAACGGGTGAGTAACGCGTAGGTAACCT  60
                ||||||||||||||||||||||||||||||||||||||||||||||||||||||||||||
Sbjct  1973838  TGTTTGGTGTTTACACTAGACTGATGAGTTGCGAACGGGTGAGTAACGCGTAGGTAACCT  1973897
Query  61       GCCTCATAGCGGGGGATAACTATTGGAAACGATAGCTAATACCGCATAAGAGTAATTAAC  120
                ||||||||||||||||||||||||||||||||||||||||||||||||||||||||||||
Sbjct  1973898  GCCTCATAGCGGGGGATAACTATTGGAAACGATAGCTAATACCGCATAAGAGTAATTAAC  1973957
Query  121      ACATGTTAGTTATTTAAAAGGAGCAATTGCTTCACTGTGAGATGGACCTGCGTTGTATTA  180
                ||||||||||||||||||||||||||||||||||||||||||||||||||||||||||||
Sbjct  1973958  ACATGTTAGTTATTTAAAAGGAGCAATTGCTTCACTGTGAGATGGACCTGCGTTGTATTA  1974017
Query  181      GCTAGTTGGTGAGGTAAAGGCTCACCAAGGCGACGATACATAGCCGACCTGAGAGGGTGA  240
                ||||||||||||||||||||||||||||||||||||||||||||||||||||||||||||
Sbjct  1974018  GCTAGTTGGTGAGGTAAAGGCTCACCAAGGCGACGATACATAGCCGACCTGAGAGGGTGA  1974077
Query  241      TCGGCCACACTGGGACTGAGACACGGCCCAGACTCCTACGGGAGGCAGCAGTAGGGAATC  300
                ||||||||||||||||||||||||||||||||||||||||||||||||||||||||||||
Sbjct  1974078  TCGGCCACACTGGGACTGAGACACGGCCCAGACTCCTACGGGAGGCAGCAGTAGGGAATC  1974137
Query  301      TTCGGCAATGGACGGAAGTCTGACCGAGCAACGCCGCGTGAGTGAAGAAGGTTTTCGGAT  360
                ||||||||||||||||||||||||||||||||||||||||||||||||||||||||||||
Sbjct  1974138  TTCGGCAATGGACGGAAGTCTGACCGAGCAACGCCGCGTGAGTGAAGAAGGTTTTCGGAT  1974197
Query  361      CGTAAAGCTCTGTTGTTAGAGAAGAACGTTGGTAGGAGTGGAAAATCTACCAAGTGACGG  420
                ||||||||||||||||||||||||||||||||||||||||||||||||||||||||||||
Sbjct  1974198  CGTAAAGCTCTGTTGTTAGAGAAGAACGTTGGTAGGAGTGGAAAATCTACCAAGTGACGG  1974257
Query  421      TAACTAACCAGAAAGGGACGGCTAACTACGTGCCAGCAGCCGCGGTAATACGTAGGTCCC  480
                ||||||||||||||||||||||||||||||||||||||||||||||||||||||||||||
Sbjct  1974258  TAACTAACCAGAAAGGGACGGCTAACTACGTGCCAGCAGCCGCGGTAATACGTAGGTCCC  1974317
Query  481      GAGCGTTGTCCGGATTTATTGGGCGTAAAGCGAGCGCAGGCGGTTCTTTAAGTCTGAAGT  540
                ||||||||||||||||||||||||||||||||||||||||||||||||||||||||||||
Sbjct  1974318  GAGCGTTGTCCGGATTTATTGGGCGTAAAGCGAGCGCAGGCGGTTCTTTAAGTCTGAAGT  1974377
Query  541      TAAAGGCAGTGGCTTAACCATTGTACGCTTTGGAAACTGGAGGACTTGAGTGCAGAAGGG  600
                ||||||||||||||||||||||||||||||||||||||||||||||||||||||||||||
Sbjct  1974378  TAAAGGCAGTGGCTTAACCATTGTACGCTTTGGAAACTGGAGGACTTGAGTGCAGAAGGG  1974437
Query  601      GAGAGTGGAATTCCATGTGTAGCGGTGAAATGCGTAGATATATGGAGGAACACCGGTGGC  660
                ............................................................
```

```
                  ||||||||||||||||||||||||||||||||||||||||||||||||||||||||||||
Sbjct  1974438   GAGAGTGGAATTCCATGTGTAGCGGTGAAATGCGTAGATATATGGAGGAACACCGGTGGC  1974497

Query  661       GAAAGCGGCTCTCTGGTCTGTAACTGACGCTGAGGCTCGAAAGCGTGGGGAGCAAACAGG  720
                  ||||||||||||||||||||||||||||||||||||||||||||||||||||||||||||
Sbjct  1974498   GAAAGCGGCTCTCTGGTCTGTAACTGACGCTGAGGCTCGAAAGCGTGGGGAGCAAACAGG  1974557

Query  721       ATTAGATACCCTGGTAGTCCACGCCGTAAACGATGAGTGCTAGGTGTTAGGCCCTTTCCG  780
                  ||||||||||||||||||||||||||||||||||||||||||||||||||| ||||||||
Sbjct  1974558   ATTAGATACCCTGGTAGTCCACGCCGTAAACGATGAGTGCTAGGTGTTAGGTCCTTTCCG  1974617

Query  781       GGGCTTAGTGCCGCAGCTAACGCATTAAGC  810
                  ||||||||||||||||||||||||||||||
Sbjct  1974618   GGGCTTAGTGCCGCAGCTAACGCATTAAGC  1974647
```

Streptococcus agalactiae strain B509 chromosome, complete genome

Sequence ID: **CP021769.1** Length: 2060637 Number of Matches: 7

Range 1: 56186 to 56995

Score	Expect	Identities	Gaps	Strand	Frame
1496 bits(810)	0.0()	810/810(100%)	0/810(0%)	Plus/Minus	

Features:

```
Query  1      TGTTTGGTGTTTACACTAGACTGATGAGTTGCGAACGGGTGAGTAACGCGTAGGTAACCT  60
              ||||||||||||||||||||||||||||||||||||||||||||||||||||||||||||
Sbjct  56995  TGTTTGGTGTTTACACTAGACTGATGAGTTGCGAACGGGTGAGTAACGCGTAGGTAACCT  56936

Query  61     GCCTCATAGCGGGGGATAACTATTGGAAACGATAGCTAATACCGCATAAGAGTAATTAAC  120
              ||||||||||||||||||||||||||||||||||||||||||||||||||||||||||||
Sbjct  56935  GCCTCATAGCGGGGGATAACTATTGGAAACGATAGCTAATACCGCATAAGAGTAATTAAC  56876

Query  121    ACATGTTAGTTATTTAAAAGGAGCAATTGCTTCACTGTGAGATGGACCTGCGTTGTATTA  180
              ||||||||||||||||||||||||||||||||||||||||||||||||||||||||||||
Sbjct  56875  ACATGTTAGTTATTTAAAAGGAGCAATTGCTTCACTGTGAGATGGACCTGCGTTGTATTA  56816

Query  181    GCTAGTTGGTGAGGTAAAGGCTCACCAAGGCGACGATACATAGCCGACCTGAGAGGGTGA  240
              ||||||||||||||||||||||||||||||||||||||||||||||||||||||||||||
Sbjct  56815  GCTAGTTGGTGAGGTAAAGGCTCACCAAGGCGACGATACATAGCCGACCTGAGAGGGTGA  56756

Query  241    TCGGCCACACTGGGACTGAGACACGGCCCAGACTCCTACGGGAGGCAGCAGTAGGGAATC  300
              ||||||||||||||||||||||||||||||||||||||||||||||||||||||||||||
Sbjct  56755  TCGGCCACACTGGGACTGAGACACGGCCCAGACTCCTACGGGAGGCAGCAGTAGGGAATC  56696

Query  301    TTCGGCAATGGACGGAAGTCTGACCGAGCAACGCCGCGTGAGTGAAGAAGGTTTTCGGAT  360
              ||||||||||||||||||||||||||||||||||||||||||||||||||||||||||||
Sbjct  56695  TTCGGCAATGGACGGAAGTCTGACCGAGCAACGCCGCGTGAGTGAAGAAGGTTTTCGGAT  56636

Query  361    CGTAAAGCTCTGTTGTTAGAGAAGAACGTTGGTAGGAGTGGAAAATCTACCAAGTGACGG  420
              ||||||||||||||||||||||||||||||||||||||||||||||||||||||||||||
Sbjct  56635  CGTAAAGCTCTGTTGTTAGAGAAGAACGTTGGTAGGAGTGGAAAATCTACCAAGTGACGG  56576

Query  421    TAACTAACCAGAAAGGGACGGCTAACTACGTGCCAGCAGCCGCGGTAATACGTAGGTCCC  480
              ||||||||||||||||||||||||||||||||||||||||||||||||||||||||||||
Sbjct  56575  TAACTAACCAGAAAGGGACGGCTAACTACGTGCCAGCAGCCGCGGTAATACGTAGGTCCC  56516

Query  481    GAGCGTTGTCCGGATTTATTGGGCGTAAAGCGAGCGCAGGCGGTTCTTTAAGTCTGAAGT  540
              ||||||||||||||||||||||||||||||||||||||||||||||||||||||||||||
Sbjct  56515  GAGCGTTGTCCGGATTTATTGGGCGTAAAGCGAGCGCAGGCGGTTCTTTAAGTCTGAAGT  56456

Query  541    TAAAGGCAGTGGCTTAACCATTGTACGCTTTGGAAACTGGAGGACTTGAGTGCAGAAGGG  600
              ||||||||||||||||||||||||||||||||||||||||||||||||||||||||||||
Sbjct  56455  TAAAGGCAGTGGCTTAACCATTGTACGCTTTGGAAACTGGAGGACTTGAGTGCAGAAGGG  56396

Query  601    GAGAGTGGAATTCCATGTGTAGCGGTGAAATGCGTAGATATATGGAGGAACACCGGTGGC  660
              ||||||||||||||||||||||||||||||||||||||||||||||||||||||||||||
Sbjct  56395  GAGAGTGGAATTCCATGTGTAGCGGTGAAATGCGTAGATATATGGAGGAACACCGGTGGC  56336

Query  661    GAAAGCGGCTCTCTGGTCTGTAACTGACGCTGAGGCTCGAAAGCGTGGGGAGCAAACAGG  720
              ||||||||||||||||||||||||||||||||||||||||||||||||||||||||||||
Sbjct  56335  GAAAGCGGCTCTCTGGTCTGTAACTGACGCTGAGGCTCGAAAGCGTGGGGAGCAAACAGG  56276

Query  721    ATTAGATACCCTGGTAGTCCACGCCGTAAACGATGAGTGCTAGGTGTTAGGCCCTTTCCG  780
              ||||||||||||||||||||||||||||||||||||||||||||||||||||||||||||
Sbjct  56275  ATTAGATACCCTGGTAGTCCACGCCGTAAACGATGAGTGCTAGGTGTTAGGCCCTTTCCG  56216

Query  781    GGGCTTAGTGCCGCAGCTAACGCATTAAGC  810
              ||||||||||||||||||||||||||||||
Sbjct  56215  GGGCTTAGTGCCGCAGCTAACGCATTAAGC  56186
```

Range 2: 125329 to 126138

Score	Expect	Identities	Gaps	Strand	Frame
1496 bits(810)	0.0()	810/810(100%)	0/810(0%)	Plus/Minus	

Features:

```
Query  1       TGTTTGGTGTTTACACTAGACTGATGAGTTGCGAACGGGTGAGTAACGCGTAGGTAACCT  60
               ||||||||||||||||||||||||||||||||||||||||||||||||||||||||||||
Sbjct  126138  TGTTTGGTGTTTACACTAGACTGATGAGTTGCGAACGGGTGAGTAACGCGTAGGTAACCT  126079

Query  61      GCCTCATAGCGGGGGATAACTATTGGAAACGATAGCTAATACCGCATAAGAGTAATTAAC  120
               ||||||||||||||||||||||||||||||||||||||||||||||||||||||||||||
Sbjct  126078  GCCTCATAGCGGGGGATAACTATTGGAAACGATAGCTAATACCGCATAAGAGTAATTAAC  126019

Query  121     ACATGTTAGTTATTTAAAAGGAGCAATTGCTTCACTGTGAGATGGACCTGCGTTGTATTA  180
               ||||||||||||||||||||||||||||||||||||||||||||||||||||||||||||
Sbjct  126018  ACATGTTAGTTATTTAAAAGGAGCAATTGCTTCACTGTGAGATGGACCTGCGTTGTATTA  125959

Query  181     GCTAGTTGGTGAGGTAAAGGCTCACCAAGGCGACGATACATAGCCGACCTGAGAGGGTGA  240
               ||||||||||||||||||||||||||||||||||||||||||||||||||||||||||||
Sbjct  125958  GCTAGTTGGTGAGGTAAAGGCTCACCAAGGCGACGATACATAGCCGACCTGAGAGGGTGA  125899

Query  241     TCGGCCACACTGGGACTGAGACACGGCCCAGACTCCTACGGGAGGCAGCAGTAGGGAATC  300
               ||||||||||||||||||||||||||||||||||||||||||||||||||||||||||||
Sbjct  125898  TCGGCCACACTGGGACTGAGACACGGCCCAGACTCCTACGGGAGGCAGCAGTAGGGAATC  125839

Query  301     TTCGGCAATGGACGGAAGTCTGACCGAGCAACGCCGCGTGAGTGAAGAAGGTTTTCGGAT  360
               ||||||||||||||||||||||||||||||||||||||||||||||||||||||||||||
Sbjct  125838  TTCGGCAATGGACGGAAGTCTGACCGAGCAACGCCGCGTGAGTGAAGAAGGTTTTCGGAT  125779

Query  361     CGTAAAGCTCTGTTGTTAGAGAAGAACGTTGGTAGGAGTGGAAAATCTACCAAGTGACGG  420
               ||||||||||||||||||||||||||||||||||||||||||||||||||||||||||||
Sbjct  125778  CGTAAAGCTCTGTTGTTAGAGAAGAACGTTGGTAGGAGTGGAAAATCTACCAAGTGACGG  125719

Query  421     TAACTAACCAGAAAGGGACGGCTAACTACGTGCCAGCAGCCGCGGTAATACGTAGGTCCC  480
               ||||||||||||||||||||||||||||||||||||||||||||||||||||||||||||
Sbjct  125718  TAACTAACCAGAAAGGGACGGCTAACTACGTGCCAGCAGCCGCGGTAATACGTAGGTCCC  125659

Query  481     GAGCGTTGTCCGGATTTATTGGGCGTAAAGCGAGCGCAGGCGGTTCTTTAAGTCTGAAGT  540
               ||||||||||||||||||||||||||||||||||||||||||||||||||||||||||||
Sbjct  125658  GAGCGTTGTCCGGATTTATTGGGCGTAAAGCGAGCGCAGGCGGTTCTTTAAGTCTGAAGT  125599

Query  541     TAAAGGCAGTGGCTTAACCATTGTACGCTTTGGAAACTGGAGGACTTGAGTGCAGAAGGG  600
               ||||||||||||||||||||||||||||||||||||||||||||||||||||||||||||
Sbjct  125598  TAAAGGCAGTGGCTTAACCATTGTACGCTTTGGAAACTGGAGGACTTGAGTGCAGAAGGG  125539

Query  601     GAGAGTGGAATTCCATGTGTAGCGGTGAAATGCGTAGATATATGGAGGAACACCGGTGGC  660
               ||||||||||||||||||||||||||||||||||||||||||||||||||||||||||||
Sbjct  125538  GAGAGTGGAATTCCATGTGTAGCGGTGAAATGCGTAGATATATGGAGGAACACCGGTGGC  125479

Query  661     GAAAGCGGCTCTCTGGTCTGTAACTGACGCTGAGGCTCGAAAGCGTGGGGAGCAAACAGG  720
               ||||||||||||||||||||||||||||||||||||||||||||||||||||||||||||
Sbjct  125478  GAAAGCGGCTCTCTGGTCTGTAACTGACGCTGAGGCTCGAAAGCGTGGGGAGCAAACAGG  125419

Query  721     ATTAGATACCCTGGTAGTCCACGCCGTAAACGATGAGTGCTAGGTGTTAGGCCCTTTCCG  780
               ||||||||||||||||||||||||||||||||||||||||||||||||||||||||||||
Sbjct  125418  ATTAGATACCCTGGTAGTCCACGCCGTAAACGATGAGTGCTAGGTGTTAGGCCCTTTCCG  125359

Query  781     GGGCTTAGTGCCGCAGCTAACGCATTAAGC  810
               ||||||||||||||||||||||||||||||
Sbjct  125358  GGGCTTAGTGCCGCAGCTAACGCATTAAGC  125329
```

Range 3: 222670 to 223479

Score	Expect	Identities	Gaps	Strand	Frame
1496 bits(810)	0.0()	810/810(100%)	0/810(0%)	Plus/Minus	

Features:

```
Query  1       TGTTTGGTGTTTACACTAGACTGATGAGTTGCGAACGGGTGAGTAACGCGTAGGTAACCT  60
               ||||||||||||||||||||||||||||||||||||||||||||||||||||||||||||
Sbjct  223479  TGTTTGGTGTTTACACTAGACTGATGAGTTGCGAACGGGTGAGTAACGCGTAGGTAACCT  223420

Query  61      GCCTCATAGCGGGGGATAACTATTGGAAACGATAGCTAATACCGCATAAGAGTAATTAAC  120
               ||||||||||||||||||||||||||||||||||||||||||||||||||||||||||||
Sbjct  223419  GCCTCATAGCGGGGGATAACTATTGGAAACGATAGCTAATACCGCATAAGAGTAATTAAC  223360

Query  121     ACATGTTAGTTATTTAAAAGGAGCAATTGCTTCACTGTGAGATGGACCTGCGTTGTATTA  180
               ||||||||||||||||||||||||||||||||||||||||||||||||||||||||||||
Sbjct  223359  ACATGTTAGTTATTTAAAAGGAGCAATTGCTTCACTGTGAGATGGACCTGCGTTGTATTA  223300

Query  181     GCTAGTTGGTGAGGTAAAGGCTCACCAAGGCGACGATACATAGCCGACCTGAGAGGGTGA  240
               ||||||||||||||||||||||||||||||||||||||||||||||||||||||||||||
Sbjct  223299  GCTAGTTGGTGAGGTAAAGGCTCACCAAGGCGACGATACATAGCCGACCTGAGAGGGTGA  223240

Query  241     TCGGCCACACTGGGACTGAGACACGGCCCAGACTCCTACGGGAGGCAGCAGTAGGGAATC  300
               ||||||||||||||||||||||||||||||||||||||||||||||||||||||||||||
Sbjct  223239  TCGGCCACACTGGGACTGAGACACGGCCCAGACTCCTACGGGAGGCAGCAGTAGGGAATC  223180

Query  301     TTCGGCAATGGACGGAAGTCTGACCGAGCAACGCCGCGTGAGTGAAGAAGGTTTTCGGAT  360
               ||||||||||||||||||||||||||||||||||||||||||||||||||||||||||||
Sbjct  223179  TTCGGCAATGGACGGAAGTCTGACCGAGCAACGCCGCGTGAGTGAAGAAGGTTTTCGGAT  223120

Query  361     CGTAAAGCTCTGTTGTTAGAGAAGAACGTTGGTAGGAGTGGAAAATCTACCAAGTGACGG  420
               ||||||||||||||||||||||||||||||||||||||||||||||||||||||||||||
Sbjct  223119  CGTAAAGCTCTGTTGTTAGAGAAGAACGTTGGTAGGAGTGGAAAATCTACCAAGTGACGG  223060

Query  421     TAACTAACCAGAAAGGGACGGCTAACTACGTGCCAGCAGCCGCGGTAATACGTAGGTCCC  480
               ||||||||||||||||||||||||||||||||||||||||||||||||||||||||||||
Sbjct  223059  TAACTAACCAGAAAGGGACGGCTAACTACGTGCCAGCAGCCGCGGTAATACGTAGGTCCC  223000

Query  481     GAGCGTTGTCCGGATTTATTGGGCGTAAAGCGAGCGCAGGCGGTTCTTTAAGTCTGAAGT  540
               ||||||||||||||||||||||||||||||||||||||||||||||||||||||||||||
Sbjct  222999  GAGCGTTGTCCGGATTTATTGGGCGTAAAGCGAGCGCAGGCGGTTCTTTAAGTCTGAAGT  222940

Query  541     TAAAGGCAGTGGCTTAACCATTGTACGCTTTGGAAACTGGAGGACTTGAGTGCAGAAGGG  600
               ||||||||||||||||||||||||||||||||||||||||||||||||||||||||||||
Sbjct  222939  TAAAGGCAGTGGCTTAACCATTGTACGCTTTGGAAACTGGAGGACTTGAGTGCAGAAGGG  222880

Query  601     GAGAGTGGAATTCCATGTGTAGCGGTGAAATGCGTAGATATATGGAGGAACACCGGTGGC  660
               ||||||||||||||||||||||||||||||||||||||||||||||||||||||||||||
Sbjct  222879  GAGAGTGGAATTCCATGTGTAGCGGTGAAATGCGTAGATATATGGAGGAACACCGGTGGC  222820

Query  661     GAAAGCGGCTCTCTGGTCTGTAACTGACGCTGAGGCTCGAAAGCGTGGGGAGCAAACAGG  720
               ||||||||||||||||||||||||||||||||||||||||||||||||||||||||||||
Sbjct  222819  GAAAGCGGCTCTCTGGTCTGTAACTGACGCTGAGGCTCGAAAGCGTGGGGAGCAAACAGG  222760

Query  721     ATTAGATACCCTGGTAGTCCACGCCGTAAACGATGAGTGCTAGGTGTTAGGCCCTTTCCG  780
               ||||||||||||||||||||||||||||||||||||||||||||||||||||||||||||
Sbjct  222759  ATTAGATACCCTGGTAGTCCACGCCGTAAACGATGAGTGCTAGGTGTTAGGCCCTTTCCG  222700

Query  781     GGGCTTAGTGCCGCAGCTAACGCATTAAGC  810
               ||||||||||||||||||||||||||||||
Sbjct  222699  GGGCTTAGTGCCGCAGCTAACGCATTAAGC  222670
```

Range 4: 391512 to 392321

Score	Expect	Identities	Gaps	Strand	Frame
1496 bits(810)	0.0()	810/810(100%)	0/810(0%)	Plus/Minus	

Features:

```
Query  1       TGTTTGGTGTTTACACTAGACTGATGAGTTGCGAACGGGTGAGTAACGCGTAGGTAACCT  60
               ||||||||||||||||||||||||||||||||||||||||||||||||||||||||||||
Sbjct  392321  TGTTTGGTGTTTACACTAGACTGATGAGTTGCGAACGGGTGAGTAACGCGTAGGTAACCT  392262

Query  61      GCCTCATAGCGGGGGATAACTATTGGAAACGATAGCTAATACCGCATAAGAGTAATTAAC  120
               ||||||||||||||||||||||||||||||||||||||||||||||||||||||||||||
Sbjct  392261  GCCTCATAGCGGGGGATAACTATTGGAAACGATAGCTAATACCGCATAAGAGTAATTAAC  392202

Query  121     ACATGTTAGTTATTTAAAAGGAGCAATTGCTTCACTGTGAGATGGACCTGCGTTGTATTA  180
               ||||||||||||||||||||||||||||||||||||||||||||||||||||||||||||
Sbjct  392201  ACATGTTAGTTATTTAAAAGGAGCAATTGCTTCACTGTGAGATGGACCTGCGTTGTATTA  392142

Query  181     GCTAGTTGGTGAGGTAAAGGCTCACCAAGGCGACGATACATAGCCGACCTGAGAGGGTGA  240
               ||||||||||||||||||||||||||||||||||||||||||||||||||||||||||||
Sbjct  392141  GCTAGTTGGTGAGGTAAAGGCTCACCAAGGCGACGATACATAGCCGACCTGAGAGGGTGA  392082

Query  241     TCGGCCACACTGGGACTGAGACACGGCCCAGACTCCTACGGGAGGCAGCAGTAGGGAATC  300
               ||||||||||||||||||||||||||||||||||||||||||||||||||||||||||||
Sbjct  392081  TCGGCCACACTGGGACTGAGACACGGCCCAGACTCCTACGGGAGGCAGCAGTAGGGAATC  392022

Query  301     TTCGGCAATGGACGGAAGTCTGACCGAGCAACGCCGCGTGAGTGAAGAAGGTTTTCGGAT  360
               ||||||||||||||||||||||||||||||||||||||||||||||||||||||||||||
Sbjct  392021  TTCGGCAATGGACGGAAGTCTGACCGAGCAACGCCGCGTGAGTGAAGAAGGTTTTCGGAT  391962

Query  361     CGTAAAGCTCTGTTGTTAGAGAAGAACGTTGGTAGGAGTGGAAAATCTACCAAGTGACGG  420
               ||||||||||||||||||||||||||||||||||||||||||||||||||||||||||||
Sbjct  391961  CGTAAAGCTCTGTTGTTAGAGAAGAACGTTGGTAGGAGTGGAAAATCTACCAAGTGACGG  391902

Query  421     TAACTAACCAGAAAGGGACGGCTAACTACGTGCCAGCAGCCGCGGTAATACGTAGGTCCC  480
               ||||||||||||||||||||||||||||||||||||||||||||||||||||||||||||
Sbjct  391901  TAACTAACCAGAAAGGGACGGCTAACTACGTGCCAGCAGCCGCGGTAATACGTAGGTCCC  391842

Query  481     GAGCGTTGTCCGGATTTATTGGGCGTAAAGCGAGCGCAGGCGGTTCTTTAAGTCTGAAGT  540
               ||||||||||||||||||||||||||||||||||||||||||||||||||||||||||||
Sbjct  391841  GAGCGTTGTCCGGATTTATTGGGCGTAAAGCGAGCGCAGGCGGTTCTTTAAGTCTGAAGT  391782

Query  541     TAAAGGCAGTGGCTTAACCATTGTACGCTTTGGAAACTGGAGGACTTGAGTGCAGAAGGG  600
               ||||||||||||||||||||||||||||||||||||||||||||||||||||||||||||
Sbjct  391781  TAAAGGCAGTGGCTTAACCATTGTACGCTTTGGAAACTGGAGGACTTGAGTGCAGAAGGG  391722

Query  601     GAGAGTGGAATTCCATGTGTAGCGGTGAAATGCGTAGATATATGGAGGAACACCGGTGGC  660
               ||||||||||||||||||||||||||||||||||||||||||||||||||||||||||||
Sbjct  391721  GAGAGTGGAATTCCATGTGTAGCGGTGAAATGCGTAGATATATGGAGGAACACCGGTGGC  391662

Query  661     GAAAGCGGCTCTCTGGTCTGTAACTGACGCTGAGGCTCGAAAGCGTGGGGAGCAAACAGG  720
               ||||||||||||||||||||||||||||||||||||||||||||||||||||||||||||
Sbjct  391661  GAAAGCGGCTCTCTGGTCTGTAACTGACGCTGAGGCTCGAAAGCGTGGGGAGCAAACAGG  391602

Query  721     ATTAGATACCCTGGTAGTCCACGCCGTAAACGATGAGTGCTAGGTGTTAGGCCCTTTCCG  780
               ||||||||||||||||||||||||||||||||||||||||||||||||||||||||||||
Sbjct  391601  ATTAGATACCCTGGTAGTCCACGCCGTAAACGATGAGTGCTAGGTGTTAGGCCCTTTCCG  391542

Query  781     GGGCTTAGTGCCGCAGCTAACGCATTAAGC  810
               ||||||||||||||||||||||||||||||
Sbjct  391541  GGGCTTAGTGCCGCAGCTAACGCATTAAGC  391512
```

Range 5: 466033 to 466842

Score	Expect	Identities	Gaps	Strand	Frame
1496 bits(810)	0.0()	810/810(100%)	0/810(0%)	Plus/Minus	

Features:

```
Query  1       TGTTTGGTGTTTACACTAGACTGATGAGTTGCGAACGGGTGAGTAACGCGTAGGTAACCT  60
               ||||||||||||||||||||||||||||||||||||||||||||||||||||||||||||
Sbjct  466842  TGTTTGGTGTTTACACTAGACTGATGAGTTGCGAACGGGTGAGTAACGCGTAGGTAACCT  466783

Query  61      GCCTCATAGCGGGGGATAACTATTGGAAACGATAGCTAATACCGCATAAGAGTAATTAAC  120
               ||||||||||||||||||||||||||||||||||||||||||||||||||||||||||||
Sbjct  466782  GCCTCATAGCGGGGGATAACTATTGGAAACGATAGCTAATACCGCATAAGAGTAATTAAC  466723

Query  121     ACATGTTAGTTATTTAAAAGGAGCAATTGCTTCACTGTGAGATGGACCTGCGTTGTATTA  180
               ||||||||||||||||||||||||||||||||||||||||||||||||||||||||||||
Sbjct  466722  ACATGTTAGTTATTTAAAAGGAGCAATTGCTTCACTGTGAGATGGACCTGCGTTGTATTA  466663

Query  181     GCTAGTTGGTGAGGTAAAGGCTCACCAAGGCGACGATACATAGCCGACCTGAGAGGGTGA  240
               ||||||||||||||||||||||||||||||||||||||||||||||||||||||||||||
Sbjct  466662  GCTAGTTGGTGAGGTAAAGGCTCACCAAGGCGACGATACATAGCCGACCTGAGAGGGTGA  466603
```

```
Query  241     TCGGCCACACTGGGACTGAGACACGGCCCAGACTCCTACGGGAGGCAGCAGTAGGGAATC  300
               ||||||||||||||||||||||||||||||||||||||||||||||||||||||||||||
Sbjct  466602  TCGGCCACACTGGGACTGAGACACGGCCCAGACTCCTACGGGAGGCAGCAGTAGGGAATC  466543

Query  301     TTCGGCAATGGACGGAAGTCTGACCGAGCAACGCCGCGTGAGTGAAGAAGGTTTTCGGAT  360
               ||||||||||||||||||||||||||||||||||||||||||||||||||||||||||||
Sbjct  466542  TTCGGCAATGGACGGAAGTCTGACCGAGCAACGCCGCGTGAGTGAAGAAGGTTTTCGGAT  466483

Query  361     CGTAAAGCTCTGTTGTTAGAGAAGAACGTTGGTAGGAGTGGAAAATCTACCAAGTGACGG  420
               ||||||||||||||||||||||||||||||||||||||||||||||||||||||||||||
Sbjct  466482  CGTAAAGCTCTGTTGTTAGAGAAGAACGTTGGTAGGAGTGGAAAATCTACCAAGTGACGG  466423

Query  421     TAACTAACCAGAAAGGGACGGCTAACTACGTGCCAGCAGCCGCGGTAATACGTAGGTCCC  480
               ||||||||||||||||||||||||||||||||||||||||||||||||||||||||||||
Sbjct  466422  TAACTAACCAGAAAGGGACGGCTAACTACGTGCCAGCAGCCGCGGTAATACGTAGGTCCC  466363

Query  481     GAGCGTTGTCCGGATTTATTGGGCGTAAAGCGAGCGCAGGCGGTTCTTTAAGTCTGAAGT  540
               ||||||||||||||||||||||||||||||||||||||||||||||||||||||||||||
Sbjct  466362  GAGCGTTGTCCGGATTTATTGGGCGTAAAGCGAGCGCAGGCGGTTCTTTAAGTCTGAAGT  466303

Query  541     TAAAGGCAGTGGCTTAACCATTGTACGCTTTGGAAACTGGAGGACTTGAGTGCAGAAGGG  600
               ||||||||||||||||||||||||||||||||||||||||||||||||||||||||||||
Sbjct  466302  TAAAGGCAGTGGCTTAACCATTGTACGCTTTGGAAACTGGAGGACTTGAGTGCAGAAGGG  466243

Query  601     GAGAGTGGAATTCCATGTGTAGCGGTGAAATGCGTAGATATATGGAGGAACACCGGTGGC  660
               ||||||||||||||||||||||||||||||||||||||||||||||||||||||||||||
Sbjct  466242  GAGAGTGGAATTCCATGTGTAGCGGTGAAATGCGTAGATATATGGAGGAACACCGGTGGC  466183

Query  661     GAAAGCGGCTCTCTGGTCTGTAACTGACGCTGAGGCTCGAAAGCGTGGGGAGCAAACAGG  720
               ||||||||||||||||||||||||||||||||||||||||||||||||||||||||||||
Sbjct  466182  GAAAGCGGCTCTCTGGTCTGTAACTGACGCTGAGGCTCGAAAGCGTGGGGAGCAAACAGG  466123

Query  721     ATTAGATACCCTGGTAGTCCACGCCGTAAACGATGAGTGCTAGGTGTTAGGCCCTTTCCG  780
               ||||||||||||||||||||||||||||||||||||||||||||||||||||||||||||
Sbjct  466122  ATTAGATACCCTGGTAGTCCACGCCGTAAACGATGAGTGCTAGGTGTTAGGCCCTTTCCG  466063

Query  781     GGGCTTAGTGCCGCAGCTAACGCATTAAGC  810
               ||||||||||||||||||||||||||||||
Sbjct  466062  GGGCTTAGTGCCGCAGCTAACGCATTAAGC  466033
```

Range 6: 307247 to 308056

Score	Expect	Identities	Gaps	Strand	Frame
1491 bits(807)	0.0()	809/810(99%)	0/810(0%)	Plus/Minus	

Features:

```
Query  1       TGTTTGGTGTTTACACTAGACTGATGAGTTGCGAACGGGTGAGTAACGCGTAGGTAACCT  60
               ||||||||||||||||||||||||||||||||||||||||||||||||||||||||||||
Sbjct  308056  TGTTTGGTGTTTACACTAGACTGATGAGTTGCGAACGGGTGAGTAACGCGTAGGTAACCT  307997

Query  61      GCCTCATAGCGGGGGATAACTATTGGAAACGATAGCTAATACCGCATAAGAGTAATTAAC  120
               ||||||||||||||||||||||||||||||||||||||||||||||||||||| ||||||
Sbjct  307996  GCCTCATAGCGGGGGATAACTATTGGAAACGATAGCTAATACCGCATAAGAGTGATTAAC  307937

Query  121     ACATGTTAGTTATTTAAAAGGAGCAATTGCTTCACTGTGAGATGGACCTGCGTTGTATTA  180
               ||||||||||||||||||||||||||||||||||||||||||||||||||||||||||||
Sbjct  307936  ACATGTTAGTTATTTAAAAGGAGCAATTGCTTCACTGTGAGATGGACCTGCGTTGTATTA  307877

Query  181     GCTAGTTGGTGAGGTAAAGGCTCACCAAGGCGACGATACATAGCCGACCTGAGAGGGTGA  240
               ||||||||||||||||||||||||||||||||||||||||||||||||||||||||||||
Sbjct  307876  GCTAGTTGGTGAGGTAAAGGCTCACCAAGGCGACGATACATAGCCGACCTGAGAGGGTGA  307817

Query  241     TCGGCCACACTGGGACTGAGACACGGCCCAGACTCCTACGGGAGGCAGCAGTAGGGAATC  300
               ||||||||||||||||||||||||||||||||||||||||||||||||||||||||||||
Sbjct  307816  TCGGCCACACTGGGACTGAGACACGGCCCAGACTCCTACGGGAGGCAGCAGTAGGGAATC  307757

Query  301     TTCGGCAATGGACGGAAGTCTGACCGAGCAACGCCGCGTGAGTGAAGAAGGTTTTCGGAT  360
               ||||||||||||||||||||||||||||||||||||||||||||||||||||||||||||
Sbjct  307756  TTCGGCAATGGACGGAAGTCTGACCGAGCAACGCCGCGTGAGTGAAGAAGGTTTTCGGAT  307697

Query  361     CGTAAAGCTCTGTTGTTAGAGAAGAACGTTGGTAGGAGTGGAAAATCTACCAAGTGACGG  420
               ||||||||||||||||||||||||||||||||||||||||||||||||||||||||||||
Sbjct  307696  CGTAAAGCTCTGTTGTTAGAGAAGAACGTTGGTAGGAGTGGAAAATCTACCAAGTGACGG  307637

Query  421     TAACTAACCAGAAAGGGACGGCTAACTACGTGCCAGCAGCCGCGGTAATACGTAGGTCCC  480
               ||||||||||||||||||||||||||||||||||||||||||||||||||||||||||||
Sbjct  307636  TAACTAACCAGAAAGGGACGGCTAACTACGTGCCAGCAGCCGCGGTAATACGTAGGTCCC  307577

Query  481     GAGCGTTGTCCGGATTTATTGGGCGTAAAGCGAGCGCAGGCGGTTCTTTAAGTCTGAAGT  540
               ||||||||||||||||||||||||||||||||||||||||||||||||||||||||||||
Sbjct  307576  GAGCGTTGTCCGGATTTATTGGGCGTAAAGCGAGCGCAGGCGGTTCTTTAAGTCTGAAGT  307517

Query  541     TAAAGGCAGTGGCTTAACCATTGTACGCTTTGGAAACTGGAGGACTTGAGTGCAGAAGGG  600
               ||||||||||||||||||||||||||||||||||||||||||||||||||||||||||||
Sbjct  307516  TAAAGGCAGTGGCTTAACCATTGTACGCTTTGGAAACTGGAGGACTTGAGTGCAGAAGGG  307457

Query  601     GAGAGTGGAATTCCATGTGTAGCGGTGAAATGCGTAGATATATGGAGGAACACCGGTGGC  660
               ||||||||||||||||||||||||||||||||||||||||||||||||||||||||||||
Sbjct  307456  GAGAGTGGAATTCCATGTGTAGCGGTGAAATGCGTAGATATATGGAGGAACACCGGTGGC  307397

Query  661     GAAAGCGGCTCTCTGGTCTGTAACTGACGCTGAGGCTCGAAAGCGTGGGGAGCAAACAGG  720
               ||||||||||||||||||||||||||||||||||||||||||||||||||||||||||||
Sbjct  307396  GAAAGCGGCTCTCTGGTCTGTAACTGACGCTGAGGCTCGAAAGCGTGGGGAGCAAACAGG  307337

Query  721     ATTAGATACCCTGGTAGTCCACGCCGTAAACGATGAGTGCTAGGTGTTAGGCCCTTTCCG  780
               ||||||||||||||||||||||||||||||||||||||||||||||||||||||||||||
Sbjct  307336  ATTAGATACCCTGGTAGTCCACGCCGTAAACGATGAGTGCTAGGTGTTAGGCCCTTTCCG  307277

Query  781     GGGCTTAGTGCCGCAGCTAACGCATTAAGC  810
               ||||||||||||||||||||||||||||||
Sbjct  307276  GGGCTTAGTGCCGCAGCTAACGCATTAAGC  307247
```

Range 7: 460201 to 461010

Score	Expect	Identities	Gaps	Strand	Frame
1491 bits(807)	0.0()	809/810(99%)	0/810(0%)	Plus/Minus	

Features:

```
Query  1       TGTTTGGTGTTTACACTAGACTGATGAGTTGCGAACGGGTGAGTAACGCGTAGGTAACCT  60
               ||||||||||||||||||||||||||||||||||||||||||||||||||||||||||||
Sbjct  461010  TGTTTGGTGTTTACACTAGACTGATGAGTTGCGAACGGGTGAGTAACGCGTAGGTAACCT  460951

Query  61      GCCTCATAGCGGGGGATAACTATTGGAAACGATAGCTAATACCGCATAAGAGTAATTAAC  120
               ||||||||||||||||||||||||||||||||||||||||||||||||||||| ||||||
Sbjct  460950  GCCTCATAGCGGGGGATAACTATTGGAAACGATAGCTAATACCGCATAAGAGTGATTAAC  460891

Query  121     ACATGTTAGTTATTTAAAAGGAGCAATTGCTTCACTGTGAGATGGACCTGCGTTGTATTA  180
               ||||||||||||||||||||||||||||||||||||||||||||||||||||||||||||
Sbjct  460890  ACATGTTAGTTATTTAAAAGGAGCAATTGCTTCACTGTGAGATGGACCTGCGTTGTATTA  460831

Query  181     GCTAGTTGGTGAGGTAAAGGCTCACCAAGGCGACGATACATAGCCGACCTGAGAGGGTGA  240
               ||||||||||||||||||||||||||||||||||||||||||||||||||||||||||||
Sbjct  460830  GCTAGTTGGTGAGGTAAAGGCTCACCAAGGCGACGATACATAGCCGACCTGAGAGGGTGA  460771

Query  241     TCGGCCACACTGGGACTGAGACACGGCCCAGACTCCTACGGGAGGCAGCAGTAGGGAATC  300
               ||||||||||||||||||||||||||||||||||||||||||||||||||||||||||||
Sbjct  460770  TCGGCCACACTGGGACTGAGACACGGCCCAGACTCCTACGGGAGGCAGCAGTAGGGAATC  460711

Query  301     TTCGGCAATGGACGGAAGTCTGACCGAGCAACGCCGCGTGAGTGAAGAAGGTTTTCGGAT  360
               ||||||||||||||||||||||||||||||||||||||||||||||||||||||||||||
Sbjct  460710  TTCGGCAATGGACGGAAGTCTGACCGAGCAACGCCGCGTGAGTGAAGAAGGTTTTCGGAT  460651

Query  361     CGTAAAGCTCTGTTGTTAGAGAAGAACGTTGGTAGGAGTGGAAAATCTACCAAGTGACGG  420
               ||||||||||||||||||||||||||||||||||||||||||||||||||||||||||||
Sbjct  460650  CGTAAAGCTCTGTTGTTAGAGAAGAACGTTGGTAGGAGTGGAAAATCTACCAAGTGACGG  460591

Query  421     TAACTAACCAGAAAGGGACGGCTAACTACGTGCCAGCAGCCGCGGTAATACGTAGGTCCC  480
               ||||||||||||||||||||||||||||||||||||||||||||||||||||||||||||
Sbjct  460590  TAACTAACCAGAAAGGGACGGCTAACTACGTGCCAGCAGCCGCGGTAATACGTAGGTCCC  460531

Query  481     GAGCGTTGTCCGGATTTATTGGGCGTAAAGCGAGCGCAGGCGGTTCTTTAAGTCTGAAGT  540
               ||||||||||||||||||||||||||||||||||||||||||||||||||||||||||||
Sbjct  460530  GAGCGTTGTCCGGATTTATTGGGCGTAAAGCGAGCGCAGGCGGTTCTTTAAGTCTGAAGT  460471

Query  541     TAAAGGCAGTGGCTTAACCATTGTACGCTTTGGAAACTGGAGGACTTGAGTGCAGAAGGG  600
               ||||||||||||||||||||||||||||||||||||||||||||||||||||||||||||
```

```
Sbjct  460470   TAAAGGCAGTGGCTTAACCATTGTACGCTTTGGAAACTGGAGGACTTGAGTGCAGAAGGG  460411
Query  601      GAGAGTGGAATTCCATGTGTAGCGGTGAAATGCGTAGATATATGGAGGAACACCGGTGGC  660
                ||||||||||||||||||||||||||||||||||||||||||||||||||||||||||||
Sbjct  460410   GAGAGTGGAATTCCATGTGTAGCGGTGAAATGCGTAGATATATGGAGGAACACCGGTGGC  460351
Query  661      GAAAGCGGCTCTCTGGTCTGTAACTGACGCTGAGGCTCGAAAGCGTGGGGAGCAAACAGG  720
                ||||||||||||||||||||||||||||||||||||||||||||||||||||||||||||
Sbjct  460350   GAAAGCGGCTCTCTGGTCTGTAACTGACGCTGAGGCTCGAAAGCGTGGGGAGCAAACAGG  460291
Query  721      ATTAGATACCCTGGTAGTCCACGCCGTAAACGATGAGTGCTAGGTGTTAGGCCCTTTCCG  780
                ||||||||||||||||||||||||||||||||||||||||||||||||||||||||||||
Sbjct  460290   ATTAGATACCCTGGTAGTCCACGCCGTAAACGATGAGTGCTAGGTGTTAGGCCCTTTCCG  460231
Query  781      GGGCTTAGTGCCGCAGCTAACGCATTAAGC  810
                ||||||||||||||||||||||||||||||
Sbjct  460230   GGGCTTAGTGCCGCAGCTAACGCATTAAGC  460201
```

Streptococcus agalactiae strain NCTC11930 genome assembly, chromosome: 1
Sequence ID: **LS483342.1** Length: 2063270 Number of Matches: 7
Range 1: 1610911 to 1611720

Score	Expect	Identities	Gaps	Strand	Frame
1496 bits(810)	0.0()	810/810(100%)	0/810(0%)	Plus/Minus	

Features:

```
Query  1        TGTTTGGTGTTTACACTAGACTGATGAGTTGCGAACGGGTGAGTAACGCGTAGGTAACCT  60
                ||||||||||||||||||||||||||||||||||||||||||||||||||||||||||||
Sbjct  1611720  TGTTTGGTGTTTACACTAGACTGATGAGTTGCGAACGGGTGAGTAACGCGTAGGTAACCT  1611661
Query  61       GCCTCATAGCGGGGGATAACTATTGGAAACGATAGCTAATACCGCATAAGAGTAATTAAC  120
                ||||||||||||||||||||||||||||||||||||||||||||||||||||||||||||
Sbjct  1611660  GCCTCATAGCGGGGGATAACTATTGGAAACGATAGCTAATACCGCATAAGAGTAATTAAC  1611601
Query  121      ACATGTTAGTTATTTAAAAGGAGCAATTGCTTCACTGTGAGATGGACCTGCGTTGTATTA  180
                ||||||||||||||||||||||||||||||||||||||||||||||||||||||||||||
Sbjct  1611600  ACATGTTAGTTATTTAAAAGGAGCAATTGCTTCACTGTGAGATGGACCTGCGTTGTATTA  1611541
Query  181      GCTAGTTGGTGAGGTAAAGGCTCACCAAGGCGACGATACATAGCCGACCTGAGAGGGTGA  240
                ||||||||||||||||||||||||||||||||||||||||||||||||||||||||||||
Sbjct  1611540  GCTAGTTGGTGAGGTAAAGGCTCACCAAGGCGACGATACATAGCCGACCTGAGAGGGTGA  1611481
Query  241      TCGGCCACACTGGGACTGAGACACGGCCCAGACTCCTACGGGAGGCAGCAGTAGGGAATC  300
                ||||||||||||||||||||||||||||||||||||||||||||||||||||||||||||
Sbjct  1611480  TCGGCCACACTGGGACTGAGACACGGCCCAGACTCCTACGGGAGGCAGCAGTAGGGAATC  1611421
Query  301      TTCGGCAATGGACGGAAGTCTGACCGAGCAACGCCGCGTGAGTGAAGAAGGTTTTCGGAT  360
                ||||||||||||||||||||||||||||||||||||||||||||||||||||||||||||
Sbjct  1611420  TTCGGCAATGGACGGAAGTCTGACCGAGCAACGCCGCGTGAGTGAAGAAGGTTTTCGGAT  1611361
Query  361      CGTAAAGCTCTGTTGTTAGAGAAGAACGTTGGTAGGAGTGGAAAATCTACCAAGTGACGG  420
                ||||||||||||||||||||||||||||||||||||||||||||||||||||||||||||
Sbjct  1611360  CGTAAAGCTCTGTTGTTAGAGAAGAACGTTGGTAGGAGTGGAAAATCTACCAAGTGACGG  1611301
Query  421      TAACTAACCAGAAAGGGACGGCTAACTACGTGCCAGCAGCCGCGGTAATACGTAGGTCCC  480
                ||||||||||||||||||||||||||||||||||||||||||||||||||||||||||||
Sbjct  1611300  TAACTAACCAGAAAGGGACGGCTAACTACGTGCCAGCAGCCGCGGTAATACGTAGGTCCC  1611241
Query  481      GAGCGTTGTCCGGATTTATTGGGCGTAAAGCGAGCGCAGGCGGTTCTTTAAGTCTGAAGT  540
                ||||||||||||||||||||||||||||||||||||||||||||||||||||||||||||
Sbjct  1611240  GAGCGTTGTCCGGATTTATTGGGCGTAAAGCGAGCGCAGGCGGTTCTTTAAGTCTGAAGT  1611181
Query  541      TAAAGGCAGTGGCTTAACCATTGTACGCTTTGGAAACTGGAGGACTTGAGTGCAGAAGGG  600
                ||||||||||||||||||||||||||||||||||||||||||||||||||||||||||||
Sbjct  1611180  TAAAGGCAGTGGCTTAACCATTGTACGCTTTGGAAACTGGAGGACTTGAGTGCAGAAGGG  1611121
Query  601      GAGAGTGGAATTCCATGTGTAGCGGTGAAATGCGTAGATATATGGAGGAACACCGGTGGC  660
                ||||||||||||||||||||||||||||||||||||||||||||||||||||||||||||
Sbjct  1611120  GAGAGTGGAATTCCATGTGTAGCGGTGAAATGCGTAGATATATGGAGGAACACCGGTGGC  1611061
Query  661      GAAAGCGGCTCTCTGGTCTGTAACTGACGCTGAGGCTCGAAAGCGTGGGGAGCAAACAGG  720
                ||||||||||||||||||||||||||||||||||||||||||||||||||||||||||||
Sbjct  1611060  GAAAGCGGCTCTCTGGTCTGTAACTGACGCTGAGGCTCGAAAGCGTGGGGAGCAAACAGG  1611001
Query  721      ATTAGATACCCTGGTAGTCCACGCCGTAAACGATGAGTGCTAGGTGTTAGGCCCTTTCCG  780
                ||||||||||||||||||||||||||||||||||||||||||||||||||||||||||||
Sbjct  1611000  ATTAGATACCCTGGTAGTCCACGCCGTAAACGATGAGTGCTAGGTGTTAGGCCCTTTCCG  1610941
Query  781      GGGCTTAGTGCCGCAGCTAACGCATTAAGC  810
                ||||||||||||||||||||||||||||||
Sbjct  1610940  GGGCTTAGTGCCGCAGCTAACGCATTAAGC  1610911
```

Range 2: 1795429 to 1796238

Score	Expect	Identities	Gaps	Strand	Frame
1496 bits(810)	0.0()	810/810(100%)	0/810(0%)	Plus/Minus	

Features:

```
Query  1        TGTTTGGTGTTTACACTAGACTGATGAGTTGCGAACGGGTGAGTAACGCGTAGGTAACCT  60
                ||||||||||||||||||||||||||||||||||||||||||||||||||||||||||||
Sbjct  1796238  TGTTTGGTGTTTACACTAGACTGATGAGTTGCGAACGGGTGAGTAACGCGTAGGTAACCT  1796179
Query  61       GCCTCATAGCGGGGGATAACTATTGGAAACGATAGCTAATACCGCATAAGAGTAATTAAC  120
                ||||||||||||||||||||||||||||||||||||||||||||||||||||||||||||
Sbjct  1796178  GCCTCATAGCGGGGGATAACTATTGGAAACGATAGCTAATACCGCATAAGAGTAATTAAC  1796119
Query  121      ACATGTTAGTTATTTAAAAGGAGCAATTGCTTCACTGTGAGATGGACCTGCGTTGTATTA  180
                ||||||||||||||||||||||||||||||||||||||||||||||||||||||||||||
Sbjct  1796118  ACATGTTAGTTATTTAAAAGGAGCAATTGCTTCACTGTGAGATGGACCTGCGTTGTATTA  1796059
Query  181      GCTAGTTGGTGAGGTAAAGGCTCACCAAGGCGACGATACATAGCCGACCTGAGAGGGTGA  240
                ||||||||||||||||||||||||||||||||||||||||||||||||||||||||||||
Sbjct  1796058  GCTAGTTGGTGAGGTAAAGGCTCACCAAGGCGACGATACATAGCCGACCTGAGAGGGTGA  1795999
Query  241      TCGGCCACACTGGGACTGAGACACGGCCCAGACTCCTACGGGAGGCAGCAGTAGGGAATC  300
                ||||||||||||||||||||||||||||||||||||||||||||||||||||||||||||
Sbjct  1795998  TCGGCCACACTGGGACTGAGACACGGCCCAGACTCCTACGGGAGGCAGCAGTAGGGAATC  1795939
Query  301      TTCGGCAATGGACGGAAGTCTGACCGAGCAACGCCGCGTGAGTGAAGAAGGTTTTCGGAT  360
                ||||||||||||||||||||||||||||||||||||||||||||||||||||||||||||
Sbjct  1795938  TTCGGCAATGGACGGAAGTCTGACCGAGCAACGCCGCGTGAGTGAAGAAGGTTTTCGGAT  1795879
Query  361      CGTAAAGCTCTGTTGTTAGAGAAGAACGTTGGTAGGAGTGGAAAATCTACCAAGTGACGG  420
                ||||||||||||||||||||||||||||||||||||||||||||||||||||||||||||
Sbjct  1795878  CGTAAAGCTCTGTTGTTAGAGAAGAACGTTGGTAGGAGTGGAAAATCTACCAAGTGACGG  1795819
Query  421      TAACTAACCAGAAAGGGACGGCTAACTACGTGCCAGCAGCCGCGGTAATACGTAGGTCCC  480
                ||||||||||||||||||||||||||||||||||||||||||||||||||||||||||||
Sbjct  1795818  TAACTAACCAGAAAGGGACGGCTAACTACGTGCCAGCAGCCGCGGTAATACGTAGGTCCC  1795759
Query  481      GAGCGTTGTCCGGATTTATTGGGCGTAAAGCGAGCGCAGGCGGTTCTTTAAGTCTGAAGT  540
                ||||||||||||||||||||||||||||||||||||||||||||||||||||||||||||
Sbjct  1795758  GAGCGTTGTCCGGATTTATTGGGCGTAAAGCGAGCGCAGGCGGTTCTTTAAGTCTGAAGT  1795699
Query  541      TAAAGGCAGTGGCTTAACCATTGTACGCTTTGGAAACTGGAGGACTTGAGTGCAGAAGGG  600
                ||||||||||||||||||||||||||||||||||||||||||||||||||||||||||||
Sbjct  1795698  TAAAGGCAGTGGCTTAACCATTGTACGCTTTGGAAACTGGAGGACTTGAGTGCAGAAGGG  1795639
Query  601      GAGAGTGGAATTCCATGTGTAGCGGTGAAATGCGTAGATATATGGAGGAACACCGGTGGC  660
                ||||||||||||||||||||||||||||||||||||||||||||||||||||||||||||
Sbjct  1795638  GAGAGTGGAATTCCATGTGTAGCGGTGAAATGCGTAGATATATGGAGGAACACCGGTGGC  1795579
Query  661      GAAAGCGGCTCTCTGGTCTGTAACTGACGCTGAGGCTCGAAAGCGTGGGGAGCAAACAGG  720
                ||||||||||||||||||||||||||||||||||||||||||||||||||||||||||||
Sbjct  1795578  GAAAGCGGCTCTCTGGTCTGTAACTGACGCTGAGGCTCGAAAGCGTGGGGAGCAAACAGG  1795519
Query  721      ATTAGATACCCTGGTAGTCCACGCCGTAAACGATGAGTGCTAGGTGTTAGGCCCTTTCCG  780
                ||||||||||||||||||||||||||||||||||||||||||||||||||||||||||||
Sbjct  1795518  ATTAGATACCCTGGTAGTCCACGCCGTAAACGATGAGTGCTAGGTGTTAGGCCCTTTCCG  1795459
Query  781      GGGCTTAGTGCCGCAGCTAACGCATTAAGC  810
                ||||||||||||||||||||||||||||||
Sbjct  1795458  GGGCTTAGTGCCGCAGCTAACGCATTAAGC  1795429
```

Range 3: 1879067 to 1879876

Score	Expect	Identities	Gaps	Strand	Frame
1496 bits(810)	0.0()	810/810(100%)	0/810(0%)	Plus/Minus	

Features:

```
Query  1        TGTTTGGTGTTTACACTAGACTGATGAGTTGCGAACGGGTGAGTAACGCGTAGGTAACCT  60
                ||||||||||||||||||||||||||||||||||||||||||||||||||||||||||||
Sbjct  1879876  TGTTTGGTGTTTACACTAGACTGATGAGTTGCGAACGGGTGAGTAACGCGTAGGTAACCT  1879817
Query  61       GCCTCATAGCGGGGGATAACTATTGGAAACGATAGCTAATACCGCATAAGAGTAATTAAC  120
                ||||||||||||||||||||||||||||||||||||||||||||||||||||||||||||
Sbjct  1879816  GCCTCATAGCGGGGGATAACTATTGGAAACGATAGCTAATACCGCATAAGAGTAATTAAC  1879757
Query  121      ACATGTTAGTTATTTAAAAGGAGCAATTGCTTCACTGTGAGATGGACCTGCGTTGTATTA  180
                ||||||||||||||||||||||||||||||||||||||||||||||||||||||||||||
Sbjct  1879756  ACATGTTAGTTATTTAAAAGGAGCAATTGCTTCACTGTGAGATGGACCTGCGTTGTATTA  1879697
Query  181      GCTAGTTGGTGAGGTAAAGGCTCACCAAGGCGACGATACATAGCCGACCTGAGAGGGTGA  240
                ||||||||||||||||||||||||||||||||||||||||||||||||||||||||||||
Sbjct  1879696  GCTAGTTGGTGAGGTAAAGGCTCACCAAGGCGACGATACATAGCCGACCTGAGAGGGTGA  1879637
Query  241      TCGGCCACACTGGGACTGAGACACGGCCCAGACTCCTACGGGAGGCAGCAGTAGGGAATC  300
                ||||||||||||||||||||||||||||||||||||||||||||||||||||||||||||
Sbjct  1879636  TCGGCCACACTGGGACTGAGACACGGCCCAGACTCCTACGGGAGGCAGCAGTAGGGAATC  1879577
Query  301      TTCGGCAATGGACGGAAGTCTGACCGAGCAACGCCGCGTGAGTGAAGAAGGTTTTCGGAT  360
                ||||||||||||||||||||||||||||||||||||||||||||||||||||||||||||
Sbjct  1879576  TTCGGCAATGGACGGAAGTCTGACCGAGCAACGCCGCGTGAGTGAAGAAGGTTTTCGGAT  1879517
Query  361      CGTAAAGCTCTGTTGTTAGAGAAGAACGTTGGTAGGAGTGGAAAATCTACCAAGTGACGG  420
                ||||||||||||||||||||||||||||||||||||||||||||||||||||||||||||
Sbjct  1879516  CGTAAAGCTCTGTTGTTAGAGAAGAACGTTGGTAGGAGTGGAAAATCTACCAAGTGACGG  1879457
Query  421      TAACTAACCAGAAAGGGACGGCTAACTACGTGCCAGCAGCCGCGGTAATACGTAGGTCCC  480
                ||||||||||||||||||||||||||||||||||||||||||||||||||||||||||||
Sbjct  1879456  TAACTAACCAGAAAGGGACGGCTAACTACGTGCCAGCAGCCGCGGTAATACGTAGGTCCC  1879397
Query  481      GAGCGTTGTCCGGATTTATTGGGCGTAAAGCGAGCGCAGGCGGTTCTTTAAGTCTGAAGT  540
                ||||||||||||||||||||||||||||||||||||||||||||||||||||||||||||
Sbjct  1879396  GAGCGTTGTCCGGATTTATTGGGCGTAAAGCGAGCGCAGGCGGTTCTTTAAGTCTGAAGT  1879337
Query  541      TAAAGGCAGTGGCTTAACCATTGTACGCTTTGGAAACTGGAGGACTTGAGTGCAGAAGGG  600
                ||||||||||||||||||||||||||||||||||||||||||||||||||||||||||||
Sbjct  1879336  TAAAGGCAGTGGCTTAACCATTGTACGCTTTGGAAACTGGAGGACTTGAGTGCAGAAGGG  1879277
Query  601      GAGAGTGGAATTCCATGTGTAGCGGTGAAATGCGTAGATATATGGAGGAACACCGGTGGC  660
                ||||||||||||||||||||||||||||||||||||||||||||||||||||||||||||
Sbjct  1879276  GAGAGTGGAATTCCATGTGTAGCGGTGAAATGCGTAGATATATGGAGGAACACCGGTGGC  1879217
Query  661      GAAAGCGGCTCTCTGGTCTGTAACTGACGCTGAGGCTCGAAAGCGTGGGGAGCAAACAGG  720
                ||||||||||||||||||||||||||||||||||||||||||||||||||||||||||||
Sbjct  1879216  GAAAGCGGCTCTCTGGTCTGTAACTGACGCTGAGGCTCGAAAGCGTGGGGAGCAAACAGG  1879157
Query  721      ATTAGATACCCTGGTAGTCCACGCCGTAAACGATGAGTGCTAGGTGTTAGGCCCTTTCCG  780
                ||||||||||||||||||||||||||||||||||||||||||||||||||||||||||||
Sbjct  1879156  ATTAGATACCCTGGTAGTCCACGCCGTAAACGATGAGTGCTAGGTGTTAGGCCCTTTCCG  1879097
Query  781      GGGCTTAGTGCCGCAGCTAACGCATTAAGC  810
                ||||||||||||||||||||||||||||||
Sbjct  1879096  GGGCTTAGTGCCGCAGCTAACGCATTAAGC  1879067
```

Range 4: 16371 to 17180

Score	Expect	Identities	Gaps	Strand	Frame
1491 bits(807)	0.0()	809/810(99%)	0/810(0%)	Plus/Plus	

Features:

```
Query  1      TGTTTGGTGTTTACACTAGACTGATGAGTTGCGAACGGGTGAGTAACGCGTAGGTAACCT  60
              ||||||||||||||||||||||||||||||||||||||||||||||||||||||||||||
Sbjct  16371  TGTTTGGTGTTTACACTAGACTGATGAGTTGCGAACGGGTGAGTAACGCGTAGGTAACCT  16430
Query  61     GCCTCATAGCGGGGGATAACTATTGGAAACGATAGCTAATACCGCATAAGAGTAATTAAC  120
              ||||||||||||||||||||||||||||||||||||||||||||||||||||| ||||||
Sbjct  16431  GCCTCATAGCGGGGGATAACTATTGGAAACGATAGCTAATACCGCATAAGAGTGATTAAC  16490
Query  121    ACATGTTAGTTATTTAAAAGGAGCAATTGCTTCACTGTGAGATGGACCTGCGTTGTATTA  180
              ||||||||||||||||||||||||||||||||||||||||||||||||||||||||||||
Sbjct  16491  ACATGTTAGTTATTTAAAAGGAGCAATTGCTTCACTGTGAGATGGACCTGCGTTGTATTA  16550
Query  181    GCTAGTTGGTGAGGTAAAGGCTCACCAAGGCGACGATACATAGCCGACCTGAGAGGGTGA  240
              ||||||||||||||||||||||||||||||||||||||||||||||||||||||||||||
Sbjct  16551  GCTAGTTGGTGAGGTAAAGGCTCACCAAGGCGACGATACATAGCCGACCTGAGAGGGTGA  16610
Query  241    TCGGCCACACTGGGACTGAGACACGGCCCAGACTCCTACGGGAGGCAGCAGTAGGGAATC  300
              ||||||||||||||||||||||||||||||||||||||||||||||||||||||||||||
Sbjct  16611  TCGGCCACACTGGGACTGAGACACGGCCCAGACTCCTACGGGAGGCAGCAGTAGGGAATC  16670
Query  301    TTCGGCAATGGACGGAAGTCTGACCGAGCAACGCCGCGTGAGTGAAGAAGGTTTTCGGAT  360
              ||||||||||||||||||||||||||||||||||||||||||||||||||||||||||||
Sbjct  16671  TTCGGCAATGGACGGAAGTCTGACCGAGCAACGCCGCGTGAGTGAAGAAGGTTTTCGGAT  16730
Query  361    CGTAAAGCTCTGTTGTTAGAGAAGAACGTTGGTAGGAGTGGAAAATCTACCAAGTGACGG  420
              ||||||||||||||||||||||||||||||||||||||||||||||||||||||||||||
Sbjct  16731  CGTAAAGCTCTGTTGTTAGAGAAGAACGTTGGTAGGAGTGGAAAATCTACCAAGTGACGG  16790
Query  421    TAACTAACCAGAAAGGGACGGCTAACTACGTGCCAGCAGCCGCGGTAATACGTAGGTCCC  480
              ||||||||||||||||||||||||||||||||||||||||||||||||||||||||||||
Sbjct  16791  TAACTAACCAGAAAGGGACGGCTAACTACGTGCCAGCAGCCGCGGTAATACGTAGGTCCC  16850
Query  481    GAGCGTTGTCCGGATTTATTGGGCGTAAAGCGAGCGCAGGCGGTTCTTTAAGTCTGAAGT  540
              ||||||||||||||||||||||||||||||||||||||||||||||||||||||||||||
Sbjct  16851  GAGCGTTGTCCGGATTTATTGGGCGTAAAGCGAGCGCAGGCGGTTCTTTAAGTCTGAAGT  16910
Query  541    TAAAGGCAGTGGCTTAACCATTGTACGCTTTGGAAACTGGAGGACTTGAGTGCAGAAGGG  600
              ||||||||||||||||||||||||||||||||||||||||||||||||||||||||||||
Sbjct  16911  TAAAGGCAGTGGCTTAACCATTGTACGCTTTGGAAACTGGAGGACTTGAGTGCAGAAGGG  16970
Query  601    GAGAGTGGAATTCCATGTGTAGCGGTGAAATGCGTAGATATATGGAGGAACACCGGTGGC  660
              ||||||||||||||||||||||||||||||||||||||||||||||||||||||||||||
Sbjct  16971  GAGAGTGGAATTCCATGTGTAGCGGTGAAATGCGTAGATATATGGAGGAACACCGGTGGC  17030
Query  661    GAAAGCGGCTCTCTGGTCTGTAACTGACGCTGAGGCTCGAAAGCGTGGGGAGCAAACAGG  720
              ||||||||||||||||||||||||||||||||||||||||||||||||||||||||||||
Sbjct  17031  GAAAGCGGCTCTCTGGTCTGTAACTGACGCTGAGGCTCGAAAGCGTGGGGAGCAAACAGG  17090
Query  721    ATTAGATACCCTGGTAGTCCACGCCGTAAACGATGAGTGCTAGGTGTTAGGCCCTTTCCG  780
              ||||||||||||||||||||||||||||||||||||||||||||||||||||||||||||
Sbjct  17091  ATTAGATACCCTGGTAGTCCACGCCGTAAACGATGAGTGCTAGGTGTTAGGCCCTTTCCG  17150
Query  781    GGGCTTAGTGCCGCAGCTAACGCATTAAGC  810
              ||||||||||||||||||||||||||||||
Sbjct  17151  GGGCTTAGTGCCGCAGCTAACGCATTAAGC  17180
```

Range 5: 22203 to 23012

Score	Expect	Identities	Gaps	Strand	Frame
1491 bits(807)	0.0()	809/810(99%)	0/810(0%)	Plus/Plus	

Features:

```
Query  1      TGTTTGGTGTTTACACTAGACTGATGAGTTGCGAACGGGTGAGTAACGCGTAGGTAACCT  60
              ||||||||||||||||||||||||||||||||||||||||||||||||||||||||||||
Sbjct  22203  TGTTTGGTGTTTACACTAGACTGATGAGTTGCGAACGGGTGAGTAACGCGTAGGTAACCT  22262
Query  61     GCCTCATAGCGGGGGATAACTATTGGAAACGATAGCTAATACCGCATAAGAGTAATTAAC  120
              ||||||||||||||||||||||||||||||||||||||||||||||||||||| ||||||
Sbjct  22263  GCCTCATAGCGGGGGATAACTATTGGAAACGATAGCTAATACCGCATAAGAGTGATTAAC  22322
Query  121    ACATGTTAGTTATTTAAAAGGAGCAATTGCTTCACTGTGAGATGGACCTGCGTTGTATTA  180
              ||||||||||||||||||||||||||||||||||||||||||||||||||||||||||||
```

```
Sbjct  22323  ACATGTTAGTTATTTAAAAGGAGCAATTGCTTCACTGTGAGATGGACCTGCGTTGTATTA  22382
Query  181    GCTAGTTGGTGAGGTAAAGGCTCACCAAGGCGACGATACATAGCCGACCTGAGAGGGTGA  240
              ||||||||||||||||||||||||||||||||||||||||||||||||||||||||||||
Sbjct  22383  GCTAGTTGGTGAGGTAAAGGCTCACCAAGGCGACGATACATAGCCGACCTGAGAGGGTGA  22442
Query  241    TCGGCCACACTGGGACTGAGACACGGCCCAGACTCCTACGGGAGGCAGCAGTAGGGAATC  300
              ||||||||||||||||||||||||||||||||||||||||||||||||||||||||||||
Sbjct  22443  TCGGCCACACTGGGACTGAGACACGGCCCAGACTCCTACGGGAGGCAGCAGTAGGGAATC  22502
Query  301    TTCGGCAATGGACGGAAGTCTGACCGAGCAACGCCGCGTGAGTGAAGAAGGTTTTCGGAT  360
              ||||||||||||||||||||||||||||||||||||||||||||||||||||||||||||
Sbjct  22503  TTCGGCAATGGACGGAAGTCTGACCGAGCAACGCCGCGTGAGTGAAGAAGGTTTTCGGAT  22562
Query  361    CGTAAAGCTCTGTTGTTAGAGAAGAACGTTGGTAGGAGTGGAAAATCTACCAAGTGACGG  420
              ||||||||||||||||||||||||||||||||||||||||||||||||||||||||||||
Sbjct  22563  CGTAAAGCTCTGTTGTTAGAGAAGAACGTTGGTAGGAGTGGAAAATCTACCAAGTGACGG  22622
Query  421    TAACTAACCAGAAAGGGACGGCTAACTACGTGCCAGCAGCCGCGGTAATACGTAGGTCCC  480
              ||||||||||||||||||||||||||||||||||||||||||||||||||||||||||||
Sbjct  22623  TAACTAACCAGAAAGGGACGGCTAACTACGTGCCAGCAGCCGCGGTAATACGTAGGTCCC  22682
Query  481    GAGCGTTGTCCGGATTTATTGGGCGTAAAGCGAGCGCAGGCGGTTCTTTAAGTCTGAAGT  540
              ||||||||||||||||||||||||||||||||||||||||||||||||||||||||||||
Sbjct  22683  GAGCGTTGTCCGGATTTATTGGGCGTAAAGCGAGCGCAGGCGGTTCTTTAAGTCTGAAGT  22742
Query  541    TAAAGGCAGTGGCTTAACCATTGTACGCTTTGGAAACTGGAGGACTTGAGTGCAGAAGGG  600
              ||||||||||||||||||||||||||||||||||||||||||||||||||||||||||||
Sbjct  22743  TAAAGGCAGTGGCTTAACCATTGTACGCTTTGGAAACTGGAGGACTTGAGTGCAGAAGGG  22802
Query  601    GAGAGTGGAATTCCATGTGTAGCGGTGAAATGCGTAGATATATGGAGGAACACCGGTGGC  660
              ||||||||||||||||||||||||||||||||||||||||||||||||||||||||||||
Sbjct  22803  GAGAGTGGAATTCCATGTGTAGCGGTGAAATGCGTAGATATATGGAGGAACACCGGTGGC  22862
Query  661    GAAAGCGGCTCTCTGGTCTGTAACTGACGCTGAGGCTCGAAAGCGTGGGGAGCAAACAGG  720
              ||||||||||||||||||||||||||||||||||||||||||||||||||||||||||||
Sbjct  22863  GAAAGCGGCTCTCTGGTCTGTAACTGACGCTGAGGCTCGAAAGCGTGGGGAGCAAACAGG  22922
Query  721    ATTAGATACCCTGGTAGTCCACGCCGTAAACGATGAGTGCTAGGTGTTAGGCCCTTTCCG  780
              ||||||||||||||||||||||||||||||||||||||||||||||||||||||||||||
Sbjct  22923  ATTAGATACCCTGGTAGTCCACGCCGTAAACGATGAGTGCTAGGTGTTAGGCCCTTTCCG  22982
Query  781    GGGCTTAGTGCCGCAGCTAACGCATTAAGC  810
              ||||||||||||||||||||||||||||||
Sbjct  22983  GGGCTTAGTGCCGCAGCTAACGCATTAAGC  23012
```

Range 6: 90892 to 91701

Score	Expect	Identities	Gaps	Strand	Frame
1491 bits(807)	0.0()	809/810(99%)	0/810(0%)	Plus/Plus	

Features:

```
Query  1      TGTTTGGTGTTTACACTAGACTGATGAGTTGCGAACGGGTGAGTAACGCGTAGGTAACCT  60
              ||||||||||||||||||||||||||||||||||||||||||||||||||||||||||||
Sbjct  90892  TGTTTGGTGTTTACACTAGACTGATGAGTTGCGAACGGGTGAGTAACGCGTAGGTAACCT  90951
Query  61     GCCTCATAGCGGGGGATAACTATTGGAAACGATAGCTAATACCGCATAAGAGTAATTAAC  120
              ||||||||||||||||||||||||||||||||||||||||||||||||||||| ||||||
Sbjct  90952  GCCTCATAGCGGGGGATAACTATTGGAAACGATAGCTAATACCGCATAAGAGTGATTAAC  91011
Query  121    ACATGTTAGTTATTTAAAAGGAGCAATTGCTTCACTGTGAGATGGACCTGCGTTGTATTA  180
              ||||||||||||||||||||||||||||||||||||||||||||||||||||||||||||
Sbjct  91012  ACATGTTAGTTATTTAAAAGGAGCAATTGCTTCACTGTGAGATGGACCTGCGTTGTATTA  91071
Query  181    GCTAGTTGGTGAGGTAAAGGCTCACCAAGGCGACGATACATAGCCGACCTGAGAGGGTGA  240
              ||||||||||||||||||||||||||||||||||||||||||||||||||||||||||||
Sbjct  91072  GCTAGTTGGTGAGGTAAAGGCTCACCAAGGCGACGATACATAGCCGACCTGAGAGGGTGA  91131
Query  241    TCGGCCACACTGGGACTGAGACACGGCCCAGACTCCTACGGGAGGCAGCAGTAGGGAATC  300
              ||||||||||||||||||||||||||||||||||||||||||||||||||||||||||||
Sbjct  91132  TCGGCCACACTGGGACTGAGACACGGCCCAGACTCCTACGGGAGGCAGCAGTAGGGAATC  91191
Query  301    TTCGGCAATGGACGGAAGTCTGACCGAGCAACGCCGCGTGAGTGAAGAAGGTTTTCGGAT  360
              ||||||||||||||||||||||||||||||||||||||||||||||||||||||||||||
Sbjct  91192  TTCGGCAATGGACGGAAGTCTGACCGAGCAACGCCGCGTGAGTGAAGAAGGTTTTCGGAT  91251
Query  361    CGTAAAGCTCTGTTGTTAGAGAAGAACGTTGGTAGGAGTGGAAAATCTACCAAGTGACGG  420
              ||||||||||||||||||||||||||||||||||||||||||||||||||||||||||||
Sbjct  91252  CGTAAAGCTCTGTTGTTAGAGAAGAACGTTGGTAGGAGTGGAAAATCTACCAAGTGACGG  91311
Query  421    TAACTAACCAGAAAGGGACGGCTAACTACGTGCCAGCAGCCGCGGTAATACGTAGGTCCC  480
              ||||||||||||||||||||||||||||||||||||||||||||||||||||||||||||
Sbjct  91312  TAACTAACCAGAAAGGGACGGCTAACTACGTGCCAGCAGCCGCGGTAATACGTAGGTCCC  91371
Query  481    GAGCGTTGTCCGGATTTATTGGGCGTAAAGCGAGCGCAGGCGGTTCTTTAAGTCTGAAGT  540
              ||||||||||||||||||||||||||||||||||||||||||||||||||||||||||||
Sbjct  91372  GAGCGTTGTCCGGATTTATTGGGCGTAAAGCGAGCGCAGGCGGTTCTTTAAGTCTGAAGT  91431
Query  541    TAAAGGCAGTGGCTTAACCATTGTACGCTTTGGAAACTGGAGGACTTGAGTGCAGAAGGG  600
              ||||||||||||||||||||||||||||||||||||||||||||||||||||||||||||
Sbjct  91432  TAAAGGCAGTGGCTTAACCATTGTACGCTTTGGAAACTGGAGGACTTGAGTGCAGAAGGG  91491
Query  601    GAGAGTGGAATTCCATGTGTAGCGGTGAAATGCGTAGATATATGGAGGAACACCGGTGGC  660
              ||||||||||||||||||||||||||||||||||||||||||||||||||||||||||||
Sbjct  91492  GAGAGTGGAATTCCATGTGTAGCGGTGAAATGCGTAGATATATGGAGGAACACCGGTGGC  91551
Query  661    GAAAGCGGCTCTCTGGTCTGTAACTGACGCTGAGGCTCGAAAGCGTGGGGAGCAAACAGG  720
              ||||||||||||||||||||||||||||||||||||||||||||||||||||||||||||
Sbjct  91552  GAAAGCGGCTCTCTGGTCTGTAACTGACGCTGAGGCTCGAAAGCGTGGGGAGCAAACAGG  91611
Query  721    ATTAGATACCCTGGTAGTCCACGCCGTAAACGATGAGTGCTAGGTGTTAGGCCCTTTCCG  780
              ||||||||||||||||||||||||||||||||||||||||||||||||||||||||||||
Sbjct  91612  ATTAGATACCCTGGTAGTCCACGCCGTAAACGATGAGTGCTAGGTGTTAGGCCCTTTCCG  91671
Query  781    GGGCTTAGTGCCGCAGCTAACGCATTAAGC  810
              ||||||||||||||||||||||||||||||
Sbjct  91672  GGGCTTAGTGCCGCAGCTAACGCATTAAGC  91701
```

Range 7: 1698087 to 1698896

Score	Expect	Identities	Gaps	Strand	Frame
1491 bits(807)	0.0()	809/810(99%)	0/810(0%)	Plus/Minus	

Features:

```
Query  1        TGTTTGGTGTTTACACTAGACTGATGAGTTGCGAACGGGTGAGTAACGCGTAGGTAACCT  60
                ||||||||||||||||||||||||||||||||||||||||||||||||||||||||||||
Sbjct  1698896  TGTTTGGTGTTTACACTAGACTGATGAGTTGCGAACGGGTGAGTAACGCGTAGGTAACCT  1698837
Query  61       GCCTCATAGCGGGGGATAACTATTGGAAACGATAGCTAATACCGCATAAGAGTAATTAAC  120
                ||||||||||||||||||||||||||||||||||||||||||||||||||||| ||||||
Sbjct  1698836  GCCTCATAGCGGGGGATAACTATTGGAAACGATAGCTAATACCGCATAAGAGTGATTAAC  1698777
Query  121      ACATGTTAGTTATTTAAAAGGAGCAATTGCTTCACTGTGAGATGGACCTGCGTTGTATTA  180
                ||||||||||||||||||||||||||||||||||||||||||||||||||||||||||||
Sbjct  1698776  ACATGTTAGTTATTTAAAAGGAGCAATTGCTTCACTGTGAGATGGACCTGCGTTGTATTA  1698717
Query  181      GCTAGTTGGTGAGGTAAAGGCTCACCAAGGCGACGATACATAGCCGACCTGAGAGGGTGA  240
                ||||||||||||||||||||||||||||||||||||||||||||||||||||||||||||
Sbjct  1698716  GCTAGTTGGTGAGGTAAAGGCTCACCAAGGCGACGATACATAGCCGACCTGAGAGGGTGA  1698657
Query  241      TCGGCCACACTGGGACTGAGACACGGCCCAGACTCCTACGGGAGGCAGCAGTAGGGAATC  300
                ||||||||||||||||||||||||||||||||||||||||||||||||||||||||||||
Sbjct  1698656  TCGGCCACACTGGGACTGAGACACGGCCCAGACTCCTACGGGAGGCAGCAGTAGGGAATC  1698597
Query  301      TTCGGCAATGGACGGAAGTCTGACCGAGCAACGCCGCGTGAGTGAAGAAGGTTTTCGGAT  360
                ||||||||||||||||||||||||||||||||||||||||||||||||||||||||||||
Sbjct  1698596  TTCGGCAATGGACGGAAGTCTGACCGAGCAACGCCGCGTGAGTGAAGAAGGTTTTCGGAT  1698537
Query  361      CGTAAAGCTCTGTTGTTAGAGAAGAACGTTGGTAGGAGTGGAAAATCTACCAAGTGACGG  420
                ||||||||||||||||||||||||||||||||||||||||||||||||||||||||||||
Sbjct  1698536  CGTAAAGCTCTGTTGTTAGAGAAGAACGTTGGTAGGAGTGGAAAATCTACCAAGTGACGG  1698477
Query  421      TAACTAACCAGAAAGGGACGGCTAACTACGTGCCAGCAGCCGCGGTAATACGTAGGTCCC  480
                ||||||||||||||||||||||||||||||||||||||||||||||||||||||||||||
Sbjct  1698476  TAACTAACCAGAAAGGGACGGCTAACTACGTGCCAGCAGCCGCGGTAATACGTAGGTCCC  1698417
Query  481      GAGCGTTGTCCGGATTTATTGGGCGTAAAGCGAGCGCAGGCGGTTCTTTAAGTCTGAAGT  540
```

```
                  ||||||||||||||||||||||||||||||||||||||||||||||||||||||||||||
Sbjct  1698416  GAGCGTTGTCCGGATTTATTGGGCGTAAAGCGAGCGCAGGCGGTTCTTTAAGTCTGAAGT  1698357
Query  541      TAAAGGCAGTGGCTTAACCATTGTACGCTTTGGAAACTGGAGGACTTGAGTGCAGAAGGG  600
                ||||||||||||||||||||||||||||||||||||||||||||||||||||||||||||
Sbjct  1698356  TAAAGGCAGTGGCTTAACCATTGTACGCTTTGGAAACTGGAGGACTTGAGTGCAGAAGGG  1698297
Query  601      GAGAGTGGAATTCCATGTGTAGCGGTGAAATGCGTAGATATATGGAGGAACACCGGTGGC  660
                ||||||||||||||||||||||||||||||||||||||||||||||||||||||||||||
Sbjct  1698296  GAGAGTGGAATTCCATGTGTAGCGGTGAAATGCGTAGATATATGGAGGAACACCGGTGGC  1698237
Query  661      GAAAGCGGCTCTCTGGTCTGTAACTGACGCTGAGGCTCGAAAGCGTGGGGAGCAAACAGG  720
                ||||||||||||||||||||||||||||||||||||||||||||||||||||||||||||
Sbjct  1698236  GAAAGCGGCTCTCTGGTCTGTAACTGACGCTGAGGCTCGAAAGCGTGGGGAGCAAACAGG  1698177
Query  721      ATTAGATACCCTGGTAGTCCACGCCGTAAACGATGAGTGCTAGGTGTTAGGCCCTTTCCG  780
                ||||||||||||||||||||||||||||||||||||||||||||||||||||||||||||
Sbjct  1698176  ATTAGATACCCTGGTAGTCCACGCCGTAAACGATGAGTGCTAGGTGTTAGGCCCTTTCCG  1698117
Query  781      GGGCTTAGTGCCGCAGCTAACGCATTAAGC  810
                ||||||||||||||||||||||||||||||
Sbjct  1698116  GGGCTTAGTGCCGCAGCTAACGCATTAAGC  1698087
```

Streptococcus agalactiae strain SGEHI2015-25 chromosome, complete genome
Sequence ID: **CP025029.1** Length: 2054713 Number of Matches: 7
Range 1: 71562 to 72371

Score	Expect	Identities	Gaps	Strand	Frame
1496 bits(810)	0.0()	810/810(100%)	0/810(0%)	Plus/Plus	

Features:

```
Query  1      TGTTTGGTGTTTACACTAGACTGATGAGTTGCGAACGGGTGAGTAACGCGTAGGTAACCT  60
              ||||||||||||||||||||||||||||||||||||||||||||||||||||||||||||
Sbjct  71562  TGTTTGGTGTTTACACTAGACTGATGAGTTGCGAACGGGTGAGTAACGCGTAGGTAACCT  71621
Query  61     GCCTCATAGCGGGGGATAACTATTGGAAACGATAGCTAATACCGCATAAGAGTAATTAAC  120
              ||||||||||||||||||||||||||||||||||||||||||||||||||||||||||||
Sbjct  71622  GCCTCATAGCGGGGGATAACTATTGGAAACGATAGCTAATACCGCATAAGAGTAATTAAC  71681
Query  121    ACATGTTAGTTATTTAAAAGGAGCAATTGCTTCACTGTGAGATGGACCTGCGTTGTATTA  180
              ||||||||||||||||||||||||||||||||||||||||||||||||||||||||||||
Sbjct  71682  ACATGTTAGTTATTTAAAAGGAGCAATTGCTTCACTGTGAGATGGACCTGCGTTGTATTA  71741
Query  181    GCTAGTTGGTGAGGTAAAGGCTCACCAAGGCGACGATACATAGCCGACCTGAGAGGGTGA  240
              ||||||||||||||||||||||||||||||||||||||||||||||||||||||||||||
Sbjct  71742  GCTAGTTGGTGAGGTAAAGGCTCACCAAGGCGACGATACATAGCCGACCTGAGAGGGTGA  71801
Query  241    TCGGCCACACTGGGACTGAGACACGGCCCAGACTCCTACGGGAGGCAGCAGTAGGGAATC  300
              ||||||||||||||||||||||||||||||||||||||||||||||||||||||||||||
Sbjct  71802  TCGGCCACACTGGGACTGAGACACGGCCCAGACTCCTACGGGAGGCAGCAGTAGGGAATC  71861
Query  301    TTCGGCAATGGACGGAAGTCTGACCGAGCAACGCCGCGTGAGTGAAGAAGGTTTTCGGAT  360
              ||||||||||||||||||||||||||||||||||||||||||||||||||||||||||||
Sbjct  71862  TTCGGCAATGGACGGAAGTCTGACCGAGCAACGCCGCGTGAGTGAAGAAGGTTTTCGGAT  71921
Query  361    CGTAAAGCTCTGTTGTTAGAGAAGAACGTTGGTAGGAGTGGAAAATCTACCAAGTGACGG  420
              ||||||||||||||||||||||||||||||||||||||||||||||||||||||||||||
Sbjct  71922  CGTAAAGCTCTGTTGTTAGAGAAGAACGTTGGTAGGAGTGGAAAATCTACCAAGTGACGG  71981
Query  421    TAACTAACCAGAAAGGGACGGCTAACTACGTGCCAGCAGCCGCGGTAATACGTAGGTCCC  480
              ||||||||||||||||||||||||||||||||||||||||||||||||||||||||||||
Sbjct  71982  TAACTAACCAGAAAGGGACGGCTAACTACGTGCCAGCAGCCGCGGTAATACGTAGGTCCC  72041
Query  481    GAGCGTTGTCCGGATTTATTGGGCGTAAAGCGAGCGCAGGCGGTTCTTTAAGTCTGAAGT  540
              ||||||||||||||||||||||||||||||||||||||||||||||||||||||||||||
Sbjct  72042  GAGCGTTGTCCGGATTTATTGGGCGTAAAGCGAGCGCAGGCGGTTCTTTAAGTCTGAAGT  72101
Query  541    TAAAGGCAGTGGCTTAACCATTGTACGCTTTGGAAACTGGAGGACTTGAGTGCAGAAGGG  600
              ||||||||||||||||||||||||||||||||||||||||||||||||||||||||||||
Sbjct  72102  TAAAGGCAGTGGCTTAACCATTGTACGCTTTGGAAACTGGAGGACTTGAGTGCAGAAGGG  72161
Query  601    GAGAGTGGAATTCCATGTGTAGCGGTGAAATGCGTAGATATATGGAGGAACACCGGTGGC  660
              ||||||||||||||||||||||||||||||||||||||||||||||||||||||||||||
Sbjct  72162  GAGAGTGGAATTCCATGTGTAGCGGTGAAATGCGTAGATATATGGAGGAACACCGGTGGC  72221
Query  661    GAAAGCGGCTCTCTGGTCTGTAACTGACGCTGAGGCTCGAAAGCGTGGGGAGCAAACAGG  720
              ||||||||||||||||||||||||||||||||||||||||||||||||||||||||||||
Sbjct  72222  GAAAGCGGCTCTCTGGTCTGTAACTGACGCTGAGGCTCGAAAGCGTGGGGAGCAAACAGG  72281
Query  721    ATTAGATACCCTGGTAGTCCACGCCGTAAACGATGAGTGCTAGGTGTTAGGCCCTTTCCG  780
              ||||||||||||||||||||||||||||||||||||||||||||||||||||||||||||
Sbjct  72282  ATTAGATACCCTGGTAGTCCACGCCGTAAACGATGAGTGCTAGGTGTTAGGCCCTTTCCG  72341
Query  781    GGGCTTAGTGCCGCAGCTAACGCATTAAGC  810
              ||||||||||||||||||||||||||||||
Sbjct  72342  GGGCTTAGTGCCGCAGCTAACGCATTAAGC  72371
```

Range 2: 2051755 to 2052564

Score	Expect	Identities	Gaps	Strand	Frame
1496 bits(810)	0.0()	810/810(100%)	0/810(0%)	Plus/Plus	

Features:

```
Query  1        TGTTTGGTGTTTACACTAGACTGATGAGTTGCGAACGGGTGAGTAACGCGTAGGTAACCT  60
                ||||||||||||||||||||||||||||||||||||||||||||||||||||||||||||
Sbjct  2051755  TGTTTGGTGTTTACACTAGACTGATGAGTTGCGAACGGGTGAGTAACGCGTAGGTAACCT  2051814
Query  61       GCCTCATAGCGGGGGATAACTATTGGAAACGATAGCTAATACCGCATAAGAGTAATTAAC  120
                ||||||||||||||||||||||||||||||||||||||||||||||||||||||||||||
Sbjct  2051815  GCCTCATAGCGGGGGATAACTATTGGAAACGATAGCTAATACCGCATAAGAGTAATTAAC  2051874
Query  121      ACATGTTAGTTATTTAAAAGGAGCAATTGCTTCACTGTGAGATGGACCTGCGTTGTATTA  180
                ||||||||||||||||||||||||||||||||||||||||||||||||||||||||||||
Sbjct  2051875  ACATGTTAGTTATTTAAAAGGAGCAATTGCTTCACTGTGAGATGGACCTGCGTTGTATTA  2051934
Query  181      GCTAGTTGGTGAGGTAAAGGCTCACCAAGGCGACGATACATAGCCGACCTGAGAGGGTGA  240
                ||||||||||||||||||||||||||||||||||||||||||||||||||||||||||||
Sbjct  2051935  GCTAGTTGGTGAGGTAAAGGCTCACCAAGGCGACGATACATAGCCGACCTGAGAGGGTGA  2051994
Query  241      TCGGCCACACTGGGACTGAGACACGGCCCAGACTCCTACGGGAGGCAGCAGTAGGGAATC  300
                ||||||||||||||||||||||||||||||||||||||||||||||||||||||||||||
Sbjct  2051995  TCGGCCACACTGGGACTGAGACACGGCCCAGACTCCTACGGGAGGCAGCAGTAGGGAATC  2052054
Query  301      TTCGGCAATGGACGGAAGTCTGACCGAGCAACGCCGCGTGAGTGAAGAAGGTTTTCGGAT  360
                ||||||||||||||||||||||||||||||||||||||||||||||||||||||||||||
Sbjct  2052055  TTCGGCAATGGACGGAAGTCTGACCGAGCAACGCCGCGTGAGTGAAGAAGGTTTTCGGAT  2052114
Query  361      CGTAAAGCTCTGTTGTTAGAGAAGAACGTTGGTAGGAGTGGAAAATCTACCAAGTGACGG  420
                ||||||||||||||||||||||||||||||||||||||||||||||||||||||||||||
Sbjct  2052115  CGTAAAGCTCTGTTGTTAGAGAAGAACGTTGGTAGGAGTGGAAAATCTACCAAGTGACGG  2052174
Query  421      TAACTAACCAGAAAGGGACGGCTAACTACGTGCCAGCAGCCGCGGTAATACGTAGGTCCC  480
                ||||||||||||||||||||||||||||||||||||||||||||||||||||||||||||
Sbjct  2052175  TAACTAACCAGAAAGGGACGGCTAACTACGTGCCAGCAGCCGCGGTAATACGTAGGTCCC  2052234
Query  481      GAGCGTTGTCCGGATTTATTGGGCGTAAAGCGAGCGCAGGCGGTTCTTTAAGTCTGAAGT  540
                ||||||||||||||||||||||||||||||||||||||||||||||||||||||||||||
Sbjct  2052235  GAGCGTTGTCCGGATTTATTGGGCGTAAAGCGAGCGCAGGCGGTTCTTTAAGTCTGAAGT  2052294
Query  541      TAAAGGCAGTGGCTTAACCATTGTACGCTTTGGAAACTGGAGGACTTGAGTGCAGAAGGG  600
                ||||||||||||||||||||||||||||||||||||||||||||||||||||||||||||
Sbjct  2052295  TAAAGGCAGTGGCTTAACCATTGTACGCTTTGGAAACTGGAGGACTTGAGTGCAGAAGGG  2052354
Query  601      GAGAGTGGAATTCCATGTGTAGCGGTGAAATGCGTAGATATATGGAGGAACACCGGTGGC  660
                ||||||||||||||||||||||||||||||||||||||||||||||||||||||||||||
Sbjct  2052355  GAGAGTGGAATTCCATGTGTAGCGGTGAAATGCGTAGATATATGGAGGAACACCGGTGGC  2052414
Query  661      GAAAGCGGCTCTCTGGTCTGTAACTGACGCTGAGGCTCGAAAGCGTGGGGAGCAAACAGG  720
                ||||||||||||||||||||||||||||||||||||||||||||||||||||||||||||
Sbjct  2052415  GAAAGCGGCTCTCTGGTCTGTAACTGACGCTGAGGCTCGAAAGCGTGGGGAGCAAACAGG  2052474
Query  721      ATTAGATACCCTGGTAGTCCACGCCGTAAACGATGAGTGCTAGGTGTTAGGCCCTTTCCG  780
                ||||||||||||||||||||||||||||||||||||||||||||||||||||||||||||
Sbjct  2052475  ATTAGATACCCTGGTAGTCCACGCCGTAAACGATGAGTGCTAGGTGTTAGGCCCTTTCCG  2052534
```

```
Query  781      GGGCTTAGTGCCGCAGCTAACGCATTAAGC  810
                ||||||||||||||||||||||||||||||
Sbjct  2052535  GGGCTTAGTGCCGCAGCTAACGCATTAAGC  2052564
```

Range 3: 2874 to 3683

Score	Expect	Identities	Gaps	Strand	Frame
1491 bits(807)	0.0()	809/810(99%)	0/810(0%)	Plus/Plus	

Features:

```
Query  1     TGTTTGGTGTTTACACTAGACTGATGAGTTGCGAACGGGTGAGTAACGCGTAGGTAACCT  60
             ||||||||||||||||||||||||||||||||||||||||||||||||||||||||||||
Sbjct  2874  TGTTTGGTGTTTACACTAGACTGATGAGTTGCGAACGGGTGAGTAACGCGTAGGTAACCT  2933

Query  61    GCCTCATAGCGGGGGATAACTATTGGAAACGATAGCTAATACCGCATAAGAGTAATTAAC  120
             ||||||||||||||||||||||||||||||||||||||||||||||||||||| ||||||
Sbjct  2934  GCCTCATAGCGGGGGATAACTATTGGAAACGATAGCTAATACCGCATAAGAGTGATTAAC  2993

Query  121   ACATGTTAGTTATTTAAAAGGAGCAATTGCTTCACTGTGAGATGGACCTGCGTTGTATTA  180
             ||||||||||||||||||||||||||||||||||||||||||||||||||||||||||||
Sbjct  2994  ACATGTTAGTTATTTAAAAGGAGCAATTGCTTCACTGTGAGATGGACCTGCGTTGTATTA  3053

Query  181   GCTAGTTGGTGAGGTAAAGGCTCACCAAGGCGACGATACATAGCCGACCTGAGAGGGTGA  240
             ||||||||||||||||||||||||||||||||||||||||||||||||||||||||||||
Sbjct  3054  GCTAGTTGGTGAGGTAAAGGCTCACCAAGGCGACGATACATAGCCGACCTGAGAGGGTGA  3113

Query  241   TCGGCCACACTGGGACTGAGACACGGCCCAGACTCCTACGGGAGGCAGCAGTAGGGAATC  300
             ||||||||||||||||||||||||||||||||||||||||||||||||||||||||||||
Sbjct  3114  TCGGCCACACTGGGACTGAGACACGGCCCAGACTCCTACGGGAGGCAGCAGTAGGGAATC  3173

Query  301   TTCGGCAATGGACGGAAGTCTGACCGAGCAACGCCGCGTGAGTGAAGAAGGTTTTCGGAT  360
             ||||||||||||||||||||||||||||||||||||||||||||||||||||||||||||
Sbjct  3174  TTCGGCAATGGACGGAAGTCTGACCGAGCAACGCCGCGTGAGTGAAGAAGGTTTTCGGAT  3233

Query  361   CGTAAAGCTCTGTTGTTAGAGAAGAACGTTGGTAGGAGTGGAAAATCTACCAAGTGACGG  420
             ||||||||||||||||||||||||||||||||||||||||||||||||||||||||||||
Sbjct  3234  CGTAAAGCTCTGTTGTTAGAGAAGAACGTTGGTAGGAGTGGAAAATCTACCAAGTGACGG  3293

Query  421   TAACTAACCAGAAAGGGACGGCTAACTACGTGCCAGCAGCCGCGGTAATACGTAGGTCCC  480
             ||||||||||||||||||||||||||||||||||||||||||||||||||||||||||||
Sbjct  3294  TAACTAACCAGAAAGGGACGGCTAACTACGTGCCAGCAGCCGCGGTAATACGTAGGTCCC  3353

Query  481   GAGCGTTGTCCGGATTTATTGGGCGTAAAGCGAGCGCAGGCGGTTCTTTAAGTCTGAAGT  540
             ||||||||||||||||||||||||||||||||||||||||||||||||||||||||||||
Sbjct  3354  GAGCGTTGTCCGGATTTATTGGGCGTAAAGCGAGCGCAGGCGGTTCTTTAAGTCTGAAGT  3413

Query  541   TAAAGGCAGTGGCTTAACCATTGTACGCTTTGGAAACTGGAGGACTTGAGTGCAGAAGGG  600
             ||||||||||||||||||||||||||||||||||||||||||||||||||||||||||||
Sbjct  3414  TAAAGGCAGTGGCTTAACCATTGTACGCTTTGGAAACTGGAGGACTTGAGTGCAGAAGGG  3473

Query  601   GAGAGTGGAATTCCATGTGTAGCGGTGAAATGCGTAGATATATGGAGGAACACCGGTGGC  660
             ||||||||||||||||||||||||||||||||||||||||||||||||||||||||||||
Sbjct  3474  GAGAGTGGAATTCCATGTGTAGCGGTGAAATGCGTAGATATATGGAGGAACACCGGTGGC  3533

Query  661   GAAAGCGGCTCTCTGGTCTGTAACTGACGCTGAGGCTCGAAAGCGTGGGGAGCAAACAGG  720
             ||||||||||||||||||||||||||||||||||||||||||||||||||||||||||||
Sbjct  3534  GAAAGCGGCTCTCTGGTCTGTAACTGACGCTGAGGCTCGAAAGCGTGGGGAGCAAACAGG  3593

Query  721   ATTAGATACCCTGGTAGTCCACGCCGTAAACGATGAGTGCTAGGTGTTAGGCCCTTTCCG  780
             ||||||||||||||||||||||||||||||||||||||||||||||||||||||||||||
Sbjct  3594  ATTAGATACCCTGGTAGTCCACGCCGTAAACGATGAGTGCTAGGTGTTAGGCCCTTTCCG  3653

Query  781   GGGCTTAGTGCCGCAGCTAACGCATTAAGC  810
             ||||||||||||||||||||||||||||||
Sbjct  3654  GGGCTTAGTGCCGCAGCTAACGCATTAAGC  3683
```

Range 4: 189206 to 190015

Score	Expect	Identities	Gaps	Strand	Frame
1491 bits(807)	0.0()	809/810(99%)	0/810(0%)	Plus/Plus	

Features:

```
Query  1       TGTTTGGTGTTTACACTAGACTGATGAGTTGCGAACGGGTGAGTAACGCGTAGGTAACCT  60
               ||||||||||||||||||||||||||||||||||||||||||||||||||||||||||||
Sbjct  189206  TGTTTGGTGTTTACACTAGACTGATGAGTTGCGAACGGGTGAGTAACGCGTAGGTAACCT  189265

Query  61      GCCTCATAGCGGGGGATAACTATTGGAAACGATAGCTAATACCGCATAAGAGTAATTAAC  120
               ||||||||||||||||||||||||||||||||||||||||||||||||||||| ||||||
Sbjct  189266  GCCTCATAGCGGGGGATAACTATTGGAAACGATAGCTAATACCGCATAAGAGTGATTAAC  189325

Query  121     ACATGTTAGTTATTTAAAAGGAGCAATTGCTTCACTGTGAGATGGACCTGCGTTGTATTA  180
               ||||||||||||||||||||||||||||||||||||||||||||||||||||||||||||
Sbjct  189326  ACATGTTAGTTATTTAAAAGGAGCAATTGCTTCACTGTGAGATGGACCTGCGTTGTATTA  189385

Query  181     GCTAGTTGGTGAGGTAAAGGCTCACCAAGGCGACGATACATAGCCGACCTGAGAGGGTGA  240
               ||||||||||||||||||||||||||||||||||||||||||||||||||||||||||||
Sbjct  189386  GCTAGTTGGTGAGGTAAAGGCTCACCAAGGCGACGATACATAGCCGACCTGAGAGGGTGA  189445

Query  241     TCGGCCACACTGGGACTGAGACACGGCCCAGACTCCTACGGGAGGCAGCAGTAGGGAATC  300
               ||||||||||||||||||||||||||||||||||||||||||||||||||||||||||||
Sbjct  189446  TCGGCCACACTGGGACTGAGACACGGCCCAGACTCCTACGGGAGGCAGCAGTAGGGAATC  189505

Query  301     TTCGGCAATGGACGGAAGTCTGACCGAGCAACGCCGCGTGAGTGAAGAAGGTTTTCGGAT  360
               ||||||||||||||||||||||||||||||||||||||||||||||||||||||||||||
Sbjct  189506  TTCGGCAATGGACGGAAGTCTGACCGAGCAACGCCGCGTGAGTGAAGAAGGTTTTCGGAT  189565

Query  361     CGTAAAGCTCTGTTGTTAGAGAAGAACGTTGGTAGGAGTGGAAAATCTACCAAGTGACGG  420
               ||||||||||||||||||||||||||||||||||||||||||||||||||||||||||||
Sbjct  189566  CGTAAAGCTCTGTTGTTAGAGAAGAACGTTGGTAGGAGTGGAAAATCTACCAAGTGACGG  189625

Query  421     TAACTAACCAGAAAGGGACGGCTAACTACGTGCCAGCAGCCGCGGTAATACGTAGGTCCC  480
               ||||||||||||||||||||||||||||||||||||||||||||||||||||||||||||
Sbjct  189626  TAACTAACCAGAAAGGGACGGCTAACTACGTGCCAGCAGCCGCGGTAATACGTAGGTCCC  189685

Query  481     GAGCGTTGTCCGGATTTATTGGGCGTAAAGCGAGCGCAGGCGGTTCTTTAAGTCTGAAGT  540
               ||||||||||||||||||||||||||||||||||||||||||||||||||||||||||||
Sbjct  189686  GAGCGTTGTCCGGATTTATTGGGCGTAAAGCGAGCGCAGGCGGTTCTTTAAGTCTGAAGT  189745

Query  541     TAAAGGCAGTGGCTTAACCATTGTACGCTTTGGAAACTGGAGGACTTGAGTGCAGAAGGG  600
               ||||||||||||||||||||||||||||||||||||||||||||||||||||||||||||
Sbjct  189746  TAAAGGCAGTGGCTTAACCATTGTACGCTTTGGAAACTGGAGGACTTGAGTGCAGAAGGG  189805

Query  601     GAGAGTGGAATTCCATGTGTAGCGGTGAAATGCGTAGATATATGGAGGAACACCGGTGGC  660
               ||||||||||||||||||||||||||||||||||||||||||||||||||||||||||||
Sbjct  189806  GAGAGTGGAATTCCATGTGTAGCGGTGAAATGCGTAGATATATGGAGGAACACCGGTGGC  189865

Query  661     GAAAGCGGCTCTCTGGTCTGTAACTGACGCTGAGGCTCGAAAGCGTGGGGAGCAAACAGG  720
               ||||||||||||||||||||||||||||||||||||||||||||||||||||||||||||
Sbjct  189866  GAAAGCGGCTCTCTGGTCTGTAACTGACGCTGAGGCTCGAAAGCGTGGGGAGCAAACAGG  189925

Query  721     ATTAGATACCCTGGTAGTCCACGCCGTAAACGATGAGTGCTAGGTGTTAGGCCCTTTCCG  780
               ||||||||||||||||||||||||||||||||||||||||||||||||||||||||||||
Sbjct  189926  ATTAGATACCCTGGTAGTCCACGCCGTAAACGATGAGTGCTAGGTGTTAGGCCCTTTCCG  189985

Query  781     GGGCTTAGTGCCGCAGCTAACGCATTAAGC  810
               ||||||||||||||||||||||||||||||
Sbjct  189986  GGGCTTAGTGCCGCAGCTAACGCATTAAGC  190015
```

Range 5: 273793 to 274602

Score	Expect	Identities	Gaps	Strand	Frame
1491 bits(807)	0.0()	809/810(99%)	0/810(0%)	Plus/Plus	

Features:

```
Query  1       TGTTTGGTGTTTACACTAGACTGATGAGTTGCGAACGGGTGAGTAACGCGTAGGTAACCT  60
               ||||||||||||||||||||||||||||||||||||||||||||||||||||||||||||
Sbjct  273793  TGTTTGGTGTTTACACTAGACTGATGAGTTGCGAACGGGTGAGTAACGCGTAGGTAACCT  273852

Query  61      GCCTCATAGCGGGGGATAACTATTGGAAACGATAGCTAATACCGCATAAGAGTAATTAAC  120
               ||||||||||||||||||||||||||||||||||||||||||||||||||||| ||||||
```

```
Sbjct  273853  GCCTCATAGCGGGGGATAACTATTGGAAACGATAGCTAATACCGCATAAGAGTGATTAAC  273912
Query  121     ACATGTTAGTTATTTAAAAGGAGCAATTGCTTCACTGTGAGATGGACCTGCGTTGTATTA  180
               ||||||||||||||||||||||||||||||||||||||||||||||||||||||||||||
Sbjct  273913  ACATGTTAGTTATTTAAAAGGAGCAATTGCTTCACTGTGAGATGGACCTGCGTTGTATTA  273972
Query  181     GCTAGTTGGTGAGGTAAAGGCTCACCAAGGCGACGATACATAGCCGACCTGAGAGGGTGA  240
               ||||||||||||||||||||||||||||||||||||||||||||||||||||||||||||
Sbjct  273973  GCTAGTTGGTGAGGTAAAGGCTCACCAAGGCGACGATACATAGCCGACCTGAGAGGGTGA  274032
Query  241     TCGGCCACACTGGGACTGAGACACGGCCCAGACTCCTACGGGAGGCAGCAGTAGGGAATC  300
               ||||||||||||||||||||||||||||||||||||||||||||||||||||||||||||
Sbjct  274033  TCGGCCACACTGGGACTGAGACACGGCCCAGACTCCTACGGGAGGCAGCAGTAGGGAATC  274092
Query  301     TTCGGCAATGGACGGAAGTCTGACCGAGCAACGCCGCGTGAGTGAAGAAGGTTTTCGGAT  360
               ||||||||||||||||||||||||||||||||||||||||||||||||||||||||||||
Sbjct  274093  TTCGGCAATGGACGGAAGTCTGACCGAGCAACGCCGCGTGAGTGAAGAAGGTTTTCGGAT  274152
Query  361     CGTAAAGCTCTGTTGTTAGAGAAGAACGTTGGTAGGAGTGGAAAATCTACCAAGTGACGG  420
               ||||||||||||||||||||||||||||||||||||||||||||||||||||||||||||
Sbjct  274153  CGTAAAGCTCTGTTGTTAGAGAAGAACGTTGGTAGGAGTGGAAAATCTACCAAGTGACGG  274212
Query  421     TAACTAACCAGAAAGGGACGGCTAACTACGTGCCAGCAGCCGCGGTAATACGTAGGTCCC  480
               ||||||||||||||||||||||||||||||||||||||||||||||||||||||||||||
Sbjct  274213  TAACTAACCAGAAAGGGACGGCTAACTACGTGCCAGCAGCCGCGGTAATACGTAGGTCCC  274272
Query  481     GAGCGTTGTCCGGATTTATTGGGCGTAAAGCGAGCGCAGGCGGTTCTTTAAGTCTGAAGT  540
               ||||||||||||||||||||||||||||||||||||||||||||||||||||||||||||
Sbjct  274273  GAGCGTTGTCCGGATTTATTGGGCGTAAAGCGAGCGCAGGCGGTTCTTTAAGTCTGAAGT  274332
Query  541     TAAAGGCAGTGGCTTAACCATTGTACGCTTTGGAAACTGGAGGACTTGAGTGCAGAAGGG  600
               ||||||||||||||||||||||||||||||||||||||||||||||||||||||||||||
Sbjct  274333  TAAAGGCAGTGGCTTAACCATTGTACGCTTTGGAAACTGGAGGACTTGAGTGCAGAAGGG  274392
Query  601     GAGAGTGGAATTCCATGTGTAGCGGTGAAATGCGTAGATATATGGAGGAACACCGGTGGC  660
               ||||||||||||||||||||||||||||||||||||||||||||||||||||||||||||
Sbjct  274393  GAGAGTGGAATTCCATGTGTAGCGGTGAAATGCGTAGATATATGGAGGAACACCGGTGGC  274452
Query  661     GAAAGCGGCTCTCTGGTCTGTAACTGACGCTGAGGCTCGAAAGCGTGGGGAGCAAACAGG  720
               ||||||||||||||||||||||||||||||||||||||||||||||||||||||||||||
Sbjct  274453  GAAAGCGGCTCTCTGGTCTGTAACTGACGCTGAGGCTCGAAAGCGTGGGGAGCAAACAGG  274512
Query  721     ATTAGATACCCTGGTAGTCCACGCCGTAAACGATGAGTGCTAGGTGTTAGGCCCTTTCCG  780
               ||||||||||||||||||||||||||||||||||||||||||||||||||||||||||||
Sbjct  274513  ATTAGATACCCTGGTAGTCCACGCCGTAAACGATGAGTGCTAGGTGTTAGGCCCTTTCCG  274572
Query  781     GGGCTTAGTGCCGCAGCTAACGCATTAAGC  810
               ||||||||||||||||||||||||||||||
Sbjct  274573  GGGCTTAGTGCCGCAGCTAACGCATTAAGC  274602
```

Range 6: 371232 to 372041

Score	Expect	Identities	Gaps	Strand	Frame
1491 bits(807)	0.0()	809/810(99%)	0/810(0%)	Plus/Plus	

Features:

```
Query  1       TGTTTGGTGTTTACACTAGACTGATGAGTTGCGAACGGGTGAGTAACGCGTAGGTAACCT  60
               ||||||||||||||||||||||||||||||||||||||||||||||||||||||||||||
Sbjct  371232  TGTTTGGTGTTTACACTAGACTGATGAGTTGCGAACGGGTGAGTAACGCGTAGGTAACCT  371291
Query  61      GCCTCATAGCGGGGGATAACTATTGGAAACGATAGCTAATACCGCATAAGAGTAATTAAC  120
               ||||||||||||||||||||||||||||||||||||||||||||||||||||| ||||||
Sbjct  371292  GCCTCATAGCGGGGGATAACTATTGGAAACGATAGCTAATACCGCATAAGAGTGATTAAC  371351
Query  121     ACATGTTAGTTATTTAAAAGGAGCAATTGCTTCACTGTGAGATGGACCTGCGTTGTATTA  180
               ||||||||||||||||||||||||||||||||||||||||||||||||||||||||||||
Sbjct  371352  ACATGTTAGTTATTTAAAAGGAGCAATTGCTTCACTGTGAGATGGACCTGCGTTGTATTA  371411
Query  181     GCTAGTTGGTGAGGTAAAGGCTCACCAAGGCGACGATACATAGCCGACCTGAGAGGGTGA  240
               ||||||||||||||||||||||||||||||||||||||||||||||||||||||||||||
Sbjct  371412  GCTAGTTGGTGAGGTAAAGGCTCACCAAGGCGACGATACATAGCCGACCTGAGAGGGTGA  371471
Query  241     TCGGCCACACTGGGACTGAGACACGGCCCAGACTCCTACGGGAGGCAGCAGTAGGGAATC  300
               ||||||||||||||||||||||||||||||||||||||||||||||||||||||||||||
Sbjct  371472  TCGGCCACACTGGGACTGAGACACGGCCCAGACTCCTACGGGAGGCAGCAGTAGGGAATC  371531
Query  301     TTCGGCAATGGACGGAAGTCTGACCGAGCAACGCCGCGTGAGTGAAGAAGGTTTTCGGAT  360
               ||||||||||||||||||||||||||||||||||||||||||||||||||||||||||||
Sbjct  371532  TTCGGCAATGGACGGAAGTCTGACCGAGCAACGCCGCGTGAGTGAAGAAGGTTTTCGGAT  371591
Query  361     CGTAAAGCTCTGTTGTTAGAGAAGAACGTTGGTAGGAGTGGAAAATCTACCAAGTGACGG  420
               ||||||||||||||||||||||||||||||||||||||||||||||||||||||||||||
Sbjct  371592  CGTAAAGCTCTGTTGTTAGAGAAGAACGTTGGTAGGAGTGGAAAATCTACCAAGTGACGG  371651
Query  421     TAACTAACCAGAAAGGGACGGCTAACTACGTGCCAGCAGCCGCGGTAATACGTAGGTCCC  480
               ||||||||||||||||||||||||||||||||||||||||||||||||||||||||||||
Sbjct  371652  TAACTAACCAGAAAGGGACGGCTAACTACGTGCCAGCAGCCGCGGTAATACGTAGGTCCC  371711
Query  481     GAGCGTTGTCCGGATTTATTGGGCGTAAAGCGAGCGCAGGCGGTTCTTTAAGTCTGAAGT  540
               ||||||||||||||||||||||||||||||||||||||||||||||||||||||||||||
Sbjct  371712  GAGCGTTGTCCGGATTTATTGGGCGTAAAGCGAGCGCAGGCGGTTCTTTAAGTCTGAAGT  371771
Query  541     TAAAGGCAGTGGCTTAACCATTGTACGCTTTGGAAACTGGAGGACTTGAGTGCAGAAGGG  600
               ||||||||||||||||||||||||||||||||||||||||||||||||||||||||||||
Sbjct  371772  TAAAGGCAGTGGCTTAACCATTGTACGCTTTGGAAACTGGAGGACTTGAGTGCAGAAGGG  371831
Query  601     GAGAGTGGAATTCCATGTGTAGCGGTGAAATGCGTAGATATATGGAGGAACACCGGTGGC  660
               ||||||||||||||||||||||||||||||||||||||||||||||||||||||||||||
Sbjct  371832  GAGAGTGGAATTCCATGTGTAGCGGTGAAATGCGTAGATATATGGAGGAACACCGGTGGC  371891
Query  661     GAAAGCGGCTCTCTGGTCTGTAACTGACGCTGAGGCTCGAAAGCGTGGGGAGCAAACAGG  720
               ||||||||||||||||||||||||||||||||||||||||||||||||||||||||||||
Sbjct  371892  GAAAGCGGCTCTCTGGTCTGTAACTGACGCTGAGGCTCGAAAGCGTGGGGAGCAAACAGG  371951
Query  721     ATTAGATACCCTGGTAGTCCACGCCGTAAACGATGAGTGCTAGGTGTTAGGCCCTTTCCG  780
               ||||||||||||||||||||||||||||||||||||||||||||||||||||||||||||
Sbjct  371952  ATTAGATACCCTGGTAGTCCACGCCGTAAACGATGAGTGCTAGGTGTTAGGCCCTTTCCG  372011
Query  781     GGGCTTAGTGCCGCAGCTAACGCATTAAGC  810
               ||||||||||||||||||||||||||||||
Sbjct  372012  GGGCTTAGTGCCGCAGCTAACGCATTAAGC  372041
```

Range 7: 440359 to 441168

Score	Expect	Identities	Gaps	Strand	Frame
1491 bits(807)	0.0()	809/810(99%)	0/810(0%)	Plus/Plus	

Features:

```
Query  1       TGTTTGGTGTTTACACTAGACTGATGAGTTGCGAACGGGTGAGTAACGCGTAGGTAACCT  60
               ||||||||||||||||||||||||||||||||||||||||||||||||||||||||||||
Sbjct  440359  TGTTTGGTGTTTACACTAGACTGATGAGTTGCGAACGGGTGAGTAACGCGTAGGTAACCT  440418
Query  61      GCCTCATAGCGGGGGATAACTATTGGAAACGATAGCTAATACCGCATAAGAGTAATTAAC  120
               ||||||||||||||||||||||||||||||||||||||||||||||||||||| ||||||
Sbjct  440419  GCCTCATAGCGGGGGATAACTATTGGAAACGATAGCTAATACCGCATAAGAGTGATTAAC  440478
Query  121     ACATGTTAGTTATTTAAAAGGAGCAATTGCTTCACTGTGAGATGGACCTGCGTTGTATTA  180
               ||||||||||||||||||||||||||||||||||||||||||||||||||||||||||||
Sbjct  440479  ACATGTTAGTTATTTAAAAGGAGCAATTGCTTCACTGTGAGATGGACCTGCGTTGTATTA  440538
Query  181     GCTAGTTGGTGAGGTAAAGGCTCACCAAGGCGACGATACATAGCCGACCTGAGAGGGTGA  240
               ||||||||||||||||||||||||||||||||||||||||||||||||||||||||||||
Sbjct  440539  GCTAGTTGGTGAGGTAAAGGCTCACCAAGGCGACGATACATAGCCGACCTGAGAGGGTGA  440598
Query  241     TCGGCCACACTGGGACTGAGACACGGCCCAGACTCCTACGGGAGGCAGCAGTAGGGAATC  300
               ||||||||||||||||||||||||||||||||||||||||||||||||||||||||||||
Sbjct  440599  TCGGCCACACTGGGACTGAGACACGGCCCAGACTCCTACGGGAGGCAGCAGTAGGGAATC  440658
Query  301     TTCGGCAATGGACGGAAGTCTGACCGAGCAACGCCGCGTGAGTGAAGAAGGTTTTCGGAT  360
               ||||||||||||||||||||||||||||||||||||||||||||||||||||||||||||
Sbjct  440659  TTCGGCAATGGACGGAAGTCTGACCGAGCAACGCCGCGTGAGTGAAGAAGGTTTTCGGAT  440718
Query  361     CGTAAAGCTCTGTTGTTAGAGAAGAACGTTGGTAGGAGTGGAAAATCTACCAAGTGACGG  420
               ||||||||||||||||||||||||||||||||||||||||||||||||||||||||||||
Sbjct  440719  CGTAAAGCTCTGTTGTTAGAGAAGAACGTTGGTAGGAGTGGAAAATCTACCAAGTGACGG  440778
Query  421     TAACTAACCAGAAAGGGACGGCTAACTACGTGCCAGCAGCCGCGGTAATACGTAGGTCCC  480
```

```
                ||||||||||||||||||||||||||||||||||||||||||||||||||||||||||||
Sbjct  440779   TAACTAACCAGAAAGGGACGGCTAACTACGTGCCAGCAGCCGCGGTAATACGTAGGTCCC  440838

Query  481      GAGCGTTGTCCGGATTTATTGGGCGTAAAGCGAGCGCAGGCGGTTCTTTAAGTCTGAAGT  540
                ||||||||||||||||||||||||||||||||||||||||||||||||||||||||||||
Sbjct  440839   GAGCGTTGTCCGGATTTATTGGGCGTAAAGCGAGCGCAGGCGGTTCTTTAAGTCTGAAGT  440898

Query  541      TAAAGGCAGTGGCTTAACCATTGTACGCTTTGGAAACTGGAGGACTTGAGTGCAGAAGGG  600
                ||||||||||||||||||||||||||||||||||||||||||||||||||||||||||||
Sbjct  440899   TAAAGGCAGTGGCTTAACCATTGTACGCTTTGGAAACTGGAGGACTTGAGTGCAGAAGGG  440958

Query  601      GAGAGTGGAATTCCATGTGTAGCGGTGAAATGCGTAGATATATGGAGGAACACCGGTGGC  660
                ||||||||||||||||||||||||||||||||||||||||||||||||||||||||||||
Sbjct  440959   GAGAGTGGAATTCCATGTGTAGCGGTGAAATGCGTAGATATATGGAGGAACACCGGTGGC  441018

Query  661      GAAAGCGGCTCTCTGGTCTGTAACTGACGCTGAGGCTCGAAAGCGTGGGGAGCAAACAGG  720
                ||||||||||||||||||||||||||||||||||||||||||||||||||||||||||||
Sbjct  441019   GAAAGCGGCTCTCTGGTCTGTAACTGACGCTGAGGCTCGAAAGCGTGGGGAGCAAACAGG  441078

Query  721      ATTAGATACCCTGGTAGTCCACGCCGTAAACGATGAGTGCTAGGTGTTAGGCCCTTTCCG  780
                ||||||||||||||||||||||||||||||||||||||||||||||||||||||||||||
Sbjct  441079   ATTAGATACCCTGGTAGTCCACGCCGTAAACGATGAGTGCTAGGTGTTAGGCCCTTTCCG  441138

Query  781      GGGCTTAGTGCCGCAGCTAACGCATTAAGC  810
                ||||||||||||||||||||||||||||||
Sbjct  441139   GGGCTTAGTGCCGCAGCTAACGCATTAAGC  441168
```

Streptococcus agalactiae strain SGEHI2015-107 chromosome, complete genome
Sequence ID: **CP025027.1** Length: 2016805 Number of Matches: 7
Range 1: 71559 to 72368

Score	Expect	Identities	Gaps	Strand	Frame
1496 bits(810)	0.0()	810/810(100%)	0/810(0%)	Plus/Plus	

Features:

```
Query  1      TGTTTGGTGTTTACACTAGACTGATGAGTTGCGAACGGGTGAGTAACGCGTAGGTAACCT  60
              ||||||||||||||||||||||||||||||||||||||||||||||||||||||||||||
Sbjct  71559  TGTTTGGTGTTTACACTAGACTGATGAGTTGCGAACGGGTGAGTAACGCGTAGGTAACCT  71618

Query  61     GCCTCATAGCGGGGGATAACTATTGGAAACGATAGCTAATACCGCATAAGAGTAATTAAC  120
              ||||||||||||||||||||||||||||||||||||||||||||||||||||||||||||
Sbjct  71619  GCCTCATAGCGGGGGATAACTATTGGAAACGATAGCTAATACCGCATAAGAGTAATTAAC  71678

Query  121    ACATGTTAGTTATTTAAAAGGAGCAATTGCTTCACTGTGAGATGGACCTGCGTTGTATTA  180
              ||||||||||||||||||||||||||||||||||||||||||||||||||||||||||||
Sbjct  71679  ACATGTTAGTTATTTAAAAGGAGCAATTGCTTCACTGTGAGATGGACCTGCGTTGTATTA  71738

Query  181    GCTAGTTGGTGAGGTAAAGGCTCACCAAGGCGACGATACATAGCCGACCTGAGAGGGTGA  240
              ||||||||||||||||||||||||||||||||||||||||||||||||||||||||||||
Sbjct  71739  GCTAGTTGGTGAGGTAAAGGCTCACCAAGGCGACGATACATAGCCGACCTGAGAGGGTGA  71798

Query  241    TCGGCCACACTGGGACTGAGACACGGCCCAGACTCCTACGGGAGGCAGCAGTAGGGAATC  300
              ||||||||||||||||||||||||||||||||||||||||||||||||||||||||||||
Sbjct  71799  TCGGCCACACTGGGACTGAGACACGGCCCAGACTCCTACGGGAGGCAGCAGTAGGGAATC  71858

Query  301    TTCGGCAATGGACGGAAGTCTGACCGAGCAACGCCGCGTGAGTGAAGAAGGTTTTCGGAT  360
              ||||||||||||||||||||||||||||||||||||||||||||||||||||||||||||
Sbjct  71859  TTCGGCAATGGACGGAAGTCTGACCGAGCAACGCCGCGTGAGTGAAGAAGGTTTTCGGAT  71918

Query  361    CGTAAAGCTCTGTTGTTAGAGAAGAACGTTGGTAGGAGTGGAAAATCTACCAAGTGACGG  420
              ||||||||||||||||||||||||||||||||||||||||||||||||||||||||||||
Sbjct  71919  CGTAAAGCTCTGTTGTTAGAGAAGAACGTTGGTAGGAGTGGAAAATCTACCAAGTGACGG  71978

Query  421    TAACTAACCAGAAAGGGACGGCTAACTACGTGCCAGCAGCCGCGGTAATACGTAGGTCCC  480
              ||||||||||||||||||||||||||||||||||||||||||||||||||||||||||||
Sbjct  71979  TAACTAACCAGAAAGGGACGGCTAACTACGTGCCAGCAGCCGCGGTAATACGTAGGTCCC  72038

Query  481    GAGCGTTGTCCGGATTTATTGGGCGTAAAGCGAGCGCAGGCGGTTCTTTAAGTCTGAAGT  540
              ||||||||||||||||||||||||||||||||||||||||||||||||||||||||||||
Sbjct  72039  GAGCGTTGTCCGGATTTATTGGGCGTAAAGCGAGCGCAGGCGGTTCTTTAAGTCTGAAGT  72098

Query  541    TAAAGGCAGTGGCTTAACCATTGTACGCTTTGGAAACTGGAGGACTTGAGTGCAGAAGGG  600
              ||||||||||||||||||||||||||||||||||||||||||||||||||||||||||||
Sbjct  72099  TAAAGGCAGTGGCTTAACCATTGTACGCTTTGGAAACTGGAGGACTTGAGTGCAGAAGGG  72158

Query  601    GAGAGTGGAATTCCATGTGTAGCGGTGAAATGCGTAGATATATGGAGGAACACCGGTGGC  660
              ||||||||||||||||||||||||||||||||||||||||||||||||||||||||||||
Sbjct  72159  GAGAGTGGAATTCCATGTGTAGCGGTGAAATGCGTAGATATATGGAGGAACACCGGTGGC  72218

Query  661    GAAAGCGGCTCTCTGGTCTGTAACTGACGCTGAGGCTCGAAAGCGTGGGGAGCAAACAGG  720
              ||||||||||||||||||||||||||||||||||||||||||||||||||||||||||||
Sbjct  72219  GAAAGCGGCTCTCTGGTCTGTAACTGACGCTGAGGCTCGAAAGCGTGGGGAGCAAACAGG  72278

Query  721    ATTAGATACCCTGGTAGTCCACGCCGTAAACGATGAGTGCTAGGTGTTAGGCCCTTTCCG  780
              ||||||||||||||||||||||||||||||||||||||||||||||||||||||||||||
Sbjct  72279  ATTAGATACCCTGGTAGTCCACGCCGTAAACGATGAGTGCTAGGTGTTAGGCCCTTTCCG  72338

Query  781    GGGCTTAGTGCCGCAGCTAACGCATTAAGC  810
              ||||||||||||||||||||||||||||||
Sbjct  72339  GGGCTTAGTGCCGCAGCTAACGCATTAAGC  72368
```

Range 2: 2874 to 3683

Score	Expect	Identities	Gaps	Strand	Frame
1491 bits(807)	0.0()	809/810(99%)	0/810(0%)	Plus/Plus	

Features:

```
Query  1     TGTTTGGTGTTTACACTAGACTGATGAGTTGCGAACGGGTGAGTAACGCGTAGGTAACCT  60
             ||||||||||||||||||||||||||||||||||||||||||||||||||||||||||||
Sbjct  2874  TGTTTGGTGTTTACACTAGACTGATGAGTTGCGAACGGGTGAGTAACGCGTAGGTAACCT  2933

Query  61    GCCTCATAGCGGGGGATAACTATTGGAAACGATAGCTAATACCGCATAAGAGTAATTAAC  120
             ||||||||||||||||||||||||||||||||||||||||||||||||||||| ||||||
Sbjct  2934  GCCTCATAGCGGGGGATAACTATTGGAAACGATAGCTAATACCGCATAAGAGTGATTAAC  2993

Query  121   ACATGTTAGTTATTTAAAAGGAGCAATTGCTTCACTGTGAGATGGACCTGCGTTGTATTA  180
             ||||||||||||||||||||||||||||||||||||||||||||||||||||||||||||
Sbjct  2994  ACATGTTAGTTATTTAAAAGGAGCAATTGCTTCACTGTGAGATGGACCTGCGTTGTATTA  3053

Query  181   GCTAGTTGGTGAGGTAAAGGCTCACCAAGGCGACGATACATAGCCGACCTGAGAGGGTGA  240
             ||||||||||||||||||||||||||||||||||||||||||||||||||||||||||||
Sbjct  3054  GCTAGTTGGTGAGGTAAAGGCTCACCAAGGCGACGATACATAGCCGACCTGAGAGGGTGA  3113

Query  241   TCGGCCACACTGGGACTGAGACACGGCCCAGACTCCTACGGGAGGCAGCAGTAGGGAATC  300
             ||||||||||||||||||||||||||||||||||||||||||||||||||||||||||||
Sbjct  3114  TCGGCCACACTGGGACTGAGACACGGCCCAGACTCCTACGGGAGGCAGCAGTAGGGAATC  3173

Query  301   TTCGGCAATGGACGGAAGTCTGACCGAGCAACGCCGCGTGAGTGAAGAAGGTTTTCGGAT  360
             ||||||||||||||||||||||||||||||||||||||||||||||||||||||||||||
Sbjct  3174  TTCGGCAATGGACGGAAGTCTGACCGAGCAACGCCGCGTGAGTGAAGAAGGTTTTCGGAT  3233

Query  361   CGTAAAGCTCTGTTGTTAGAGAAGAACGTTGGTAGGAGTGGAAAATCTACCAAGTGACGG  420
             ||||||||||||||||||||||||||||||||||||||||||||||||||||||||||||
Sbjct  3234  CGTAAAGCTCTGTTGTTAGAGAAGAACGTTGGTAGGAGTGGAAAATCTACCAAGTGACGG  3293

Query  421   TAACTAACCAGAAAGGGACGGCTAACTACGTGCCAGCAGCCGCGGTAATACGTAGGTCCC  480
             ||||||||||||||||||||||||||||||||||||||||||||||||||||||||||||
Sbjct  3294  TAACTAACCAGAAAGGGACGGCTAACTACGTGCCAGCAGCCGCGGTAATACGTAGGTCCC  3353

Query  481   GAGCGTTGTCCGGATTTATTGGGCGTAAAGCGAGCGCAGGCGGTTCTTTAAGTCTGAAGT  540
             ||||||||||||||||||||||||||||||||||||||||||||||||||||||||||||
Sbjct  3354  GAGCGTTGTCCGGATTTATTGGGCGTAAAGCGAGCGCAGGCGGTTCTTTAAGTCTGAAGT  3413

Query  541   TAAAGGCAGTGGCTTAACCATTGTACGCTTTGGAAACTGGAGGACTTGAGTGCAGAAGGG  600
             ||||||||||||||||||||||||||||||||||||||||||||||||||||||||||||
Sbjct  3414  TAAAGGCAGTGGCTTAACCATTGTACGCTTTGGAAACTGGAGGACTTGAGTGCAGAAGGG  3473

Query  601   GAGAGTGGAATTCCATGTGTAGCGGTGAAATGCGTAGATATATGGAGGAACACCGGTGGC  660
             ||||||||||||||||||||||||||||||||||||||||||||||||||||||||||||
Sbjct  3474  GAGAGTGGAATTCCATGTGTAGCGGTGAAATGCGTAGATATATGGAGGAACACCGGTGGC  3533

Query  661   GAAAGCGGCTCTCTGGTCTGTAACTGACGCTGAGGCTCGAAAGCGTGGGGAGCAAACAGG  720
             ||||||||||||||||||||||||||||||||||||||||||||||||||||||||||||
Sbjct  3534  GAAAGCGGCTCTCTGGTCTGTAACTGACGCTGAGGCTCGAAAGCGTGGGGAGCAAACAGG  3593
```

```
Query  721   ATTAGATACCCTGGTAGTCCACGCCGTAAACGATGAGTGCTAGGTGTTAGGCCCTTTCCG  780
             ||||||||||||||||||||||||||||||||||||||||||||||||||||||||||||
Sbjct  3594  ATTAGATACCCTGGTAGTCCACGCCGTAAACGATGAGTGCTAGGTGTTAGGCCCTTTCCG  3653

Query  781   GGGCTTAGTGCCGCAGCTAACGCATTAAGC  810
             ||||||||||||||||||||||||||||||
Sbjct  3654  GGGCTTAGTGCCGCAGCTAACGCATTAAGC  3683
```

Range 3: 151251 to 152060

Score	Expect	Identities	Gaps	Strand	Frame
1491 bits(807)	0.0()	809/810(99%)	0/810(0%)	Plus/Plus	

Features:

```
Query  1       TGTTTGGTGTTTACACTAGACTGATGAGTTGCGAACGGGTGAGTAACGCGTAGGTAACCT  60
               ||||||||||||||||||||||||||||||||||||||||||||||||||||||||||||
Sbjct  151251  TGTTTGGTGTTTACACTAGACTGATGAGTTGCGAACGGGTGAGTAACGCGTAGGTAACCT  151310

Query  61      GCCTCATAGCGGGGGATAACTATTGGAAACGATAGCTAATACCGCATAAGAGTAATTAAC  120
               ||||||||||||||||||||||||||||||||||||||||||||||||||||| ||||||
Sbjct  151311  GCCTCATAGCGGGGGATAACTATTGGAAACGATAGCTAATACCGCATAAGAGTGATTAAC  151370

Query  121     ACATGTTAGTTATTTAAAAGGAGCAATTGCTTCACTGTGAGATGGACCTGCGTTGTATTA  180
               ||||||||||||||||||||||||||||||||||||||||||||||||||||||||||||
Sbjct  151371  ACATGTTAGTTATTTAAAAGGAGCAATTGCTTCACTGTGAGATGGACCTGCGTTGTATTA  151430

Query  181     GCTAGTTGGTGAGGTAAAGGCTCACCAAGGCGACGATACATAGCCGACCTGAGAGGGTGA  240
               ||||||||||||||||||||||||||||||||||||||||||||||||||||||||||||
Sbjct  151431  GCTAGTTGGTGAGGTAAAGGCTCACCAAGGCGACGATACATAGCCGACCTGAGAGGGTGA  151490

Query  241     TCGGCCACACTGGGACTGAGACACGGCCCAGACTCCTACGGGAGGCAGCAGTAGGGAATC  300
               ||||||||||||||||||||||||||||||||||||||||||||||||||||||||||||
Sbjct  151491  TCGGCCACACTGGGACTGAGACACGGCCCAGACTCCTACGGGAGGCAGCAGTAGGGAATC  151550

Query  301     TTCGGCAATGGACGGAAGTCTGACCGAGCAACGCCGCGTGAGTGAAGAAGGTTTTCGGAT  360
               ||||||||||||||||||||||||||||||||||||||||||||||||||||||||||||
Sbjct  151551  TTCGGCAATGGACGGAAGTCTGACCGAGCAACGCCGCGTGAGTGAAGAAGGTTTTCGGAT  151610

Query  361     CGTAAAGCTCTGTTGTTAGAGAAGAACGTTGGTAGGAGTGGAAAATCTACCAAGTGACGG  420
               ||||||||||||||||||||||||||||||||||||||||||||||||||||||||||||
Sbjct  151611  CGTAAAGCTCTGTTGTTAGAGAAGAACGTTGGTAGGAGTGGAAAATCTACCAAGTGACGG  151670

Query  421     TAACTAACCAGAAAGGGACGGCTAACTACGTGCCAGCAGCCGCGGTAATACGTAGGTCCC  480
               ||||||||||||||||||||||||||||||||||||||||||||||||||||||||||||
Sbjct  151671  TAACTAACCAGAAAGGGACGGCTAACTACGTGCCAGCAGCCGCGGTAATACGTAGGTCCC  151730

Query  481     GAGCGTTGTCCGGATTTATTGGGCGTAAAGCGAGCGCAGGCGGTTCTTTAAGTCTGAAGT  540
               ||||||||||||||||||||||||||||||||||||||||||||||||||||||||||||
Sbjct  151731  GAGCGTTGTCCGGATTTATTGGGCGTAAAGCGAGCGCAGGCGGTTCTTTAAGTCTGAAGT  151790

Query  541     TAAAGGCAGTGGCTTAACCATTGTACGCTTTGGAAACTGGAGGACTTGAGTGCAGAAGGG  600
               ||||||||||||||||||||||||||||||||||||||||||||||||||||||||||||
Sbjct  151791  TAAAGGCAGTGGCTTAACCATTGTACGCTTTGGAAACTGGAGGACTTGAGTGCAGAAGGG  151850

Query  601     GAGAGTGGAATTCCATGTGTAGCGGTGAAATGCGTAGATATATGGAGGAACACCGGTGGC  660
               ||||||||||||||||||||||||||||||||||||||||||||||||||||||||||||
Sbjct  151851  GAGAGTGGAATTCCATGTGTAGCGGTGAAATGCGTAGATATATGGAGGAACACCGGTGGC  151910

Query  661     GAAAGCGGCTCTCTGGTCTGTAACTGACGCTGAGGCTCGAAAGCGTGGGGAGCAAACAGG  720
               ||||||||||||||||||||||||||||||||||||||||||||||||||||||||||||
Sbjct  151911  GAAAGCGGCTCTCTGGTCTGTAACTGACGCTGAGGCTCGAAAGCGTGGGGAGCAAACAGG  151970

Query  721     ATTAGATACCCTGGTAGTCCACGCCGTAAACGATGAGTGCTAGGTGTTAGGCCCTTTCCG  780
               ||||||||||||||||||||||||||||||||||||||||||||||||||||||||||||
Sbjct  151971  ATTAGATACCCTGGTAGTCCACGCCGTAAACGATGAGTGCTAGGTGTTAGGCCCTTTCCG  152030

Query  781     GGGCTTAGTGCCGCAGCTAACGCATTAAGC  810
               ||||||||||||||||||||||||||||||
Sbjct  152031  GGGCTTAGTGCCGCAGCTAACGCATTAAGC  152060
```

Range 4: 235838 to 236647

Score	Expect	Identities	Gaps	Strand	Frame
1491 bits(807)	0.0()	809/810(99%)	0/810(0%)	Plus/Plus	

Features:

```
Query  1       TGTTTGGTGTTTACACTAGACTGATGAGTTGCGAACGGGTGAGTAACGCGTAGGTAACCT  60
               ||||||||||||||||||||||||||||||||||||||||||||||||||||||||||||
Sbjct  235838  TGTTTGGTGTTTACACTAGACTGATGAGTTGCGAACGGGTGAGTAACGCGTAGGTAACCT  235897

Query  61      GCCTCATAGCGGGGGATAACTATTGGAAACGATAGCTAATACCGCATAAGAGTAATTAAC  120
               ||||||||||||||||||||||||||||||||||||||||||||||||||||| ||||||
Sbjct  235898  GCCTCATAGCGGGGGATAACTATTGGAAACGATAGCTAATACCGCATAAGAGTGATTAAC  235957

Query  121     ACATGTTAGTTATTTAAAAGGAGCAATTGCTTCACTGTGAGATGGACCTGCGTTGTATTA  180
               ||||||||||||||||||||||||||||||||||||||||||||||||||||||||||||
Sbjct  235958  ACATGTTAGTTATTTAAAAGGAGCAATTGCTTCACTGTGAGATGGACCTGCGTTGTATTA  236017

Query  181     GCTAGTTGGTGAGGTAAAGGCTCACCAAGGCGACGATACATAGCCGACCTGAGAGGGTGA  240
               ||||||||||||||||||||||||||||||||||||||||||||||||||||||||||||
Sbjct  236018  GCTAGTTGGTGAGGTAAAGGCTCACCAAGGCGACGATACATAGCCGACCTGAGAGGGTGA  236077

Query  241     TCGGCCACACTGGGACTGAGACACGGCCCAGACTCCTACGGGAGGCAGCAGTAGGGAATC  300
               ||||||||||||||||||||||||||||||||||||||||||||||||||||||||||||
Sbjct  236078  TCGGCCACACTGGGACTGAGACACGGCCCAGACTCCTACGGGAGGCAGCAGTAGGGAATC  236137

Query  301     TTCGGCAATGGACGGAAGTCTGACCGAGCAACGCCGCGTGAGTGAAGAAGGTTTTCGGAT  360
               ||||||||||||||||||||||||||||||||||||||||||||||||||||||||||||
Sbjct  236138  TTCGGCAATGGACGGAAGTCTGACCGAGCAACGCCGCGTGAGTGAAGAAGGTTTTCGGAT  236197

Query  361     CGTAAAGCTCTGTTGTTAGAGAAGAACGTTGGTAGGAGTGGAAAATCTACCAAGTGACGG  420
               ||||||||||||||||||||||||||||||||||||||||||||||||||||||||||||
Sbjct  236198  CGTAAAGCTCTGTTGTTAGAGAAGAACGTTGGTAGGAGTGGAAAATCTACCAAGTGACGG  236257

Query  421     TAACTAACCAGAAAGGGACGGCTAACTACGTGCCAGCAGCCGCGGTAATACGTAGGTCCC  480
               ||||||||||||||||||||||||||||||||||||||||||||||||||||||||||||
Sbjct  236258  TAACTAACCAGAAAGGGACGGCTAACTACGTGCCAGCAGCCGCGGTAATACGTAGGTCCC  236317

Query  481     GAGCGTTGTCCGGATTTATTGGGCGTAAAGCGAGCGCAGGCGGTTCTTTAAGTCTGAAGT  540
               ||||||||||||||||||||||||||||||||||||||||||||||||||||||||||||
Sbjct  236318  GAGCGTTGTCCGGATTTATTGGGCGTAAAGCGAGCGCAGGCGGTTCTTTAAGTCTGAAGT  236377

Query  541     TAAAGGCAGTGGCTTAACCATTGTACGCTTTGGAAACTGGAGGACTTGAGTGCAGAAGGG  600
               ||||||||||||||||||||||||||||||||||||||||||||||||||||||||||||
Sbjct  236378  TAAAGGCAGTGGCTTAACCATTGTACGCTTTGGAAACTGGAGGACTTGAGTGCAGAAGGG  236437

Query  601     GAGAGTGGAATTCCATGTGTAGCGGTGAAATGCGTAGATATATGGAGGAACACCGGTGGC  660
               ||||||||||||||||||||||||||||||||||||||||||||||||||||||||||||
Sbjct  236438  GAGAGTGGAATTCCATGTGTAGCGGTGAAATGCGTAGATATATGGAGGAACACCGGTGGC  236497

Query  661     GAAAGCGGCTCTCTGGTCTGTAACTGACGCTGAGGCTCGAAAGCGTGGGGAGCAAACAGG  720
               ||||||||||||||||||||||||||||||||||||||||||||||||||||||||||||
Sbjct  236498  GAAAGCGGCTCTCTGGTCTGTAACTGACGCTGAGGCTCGAAAGCGTGGGGAGCAAACAGG  236557

Query  721     ATTAGATACCCTGGTAGTCCACGCCGTAAACGATGAGTGCTAGGTGTTAGGCCCTTTCCG  780
               ||||||||||||||||||||||||||||||||||||||||||||||||||||||||||||
Sbjct  236558  ATTAGATACCCTGGTAGTCCACGCCGTAAACGATGAGTGCTAGGTGTTAGGCCCTTTCCG  236617

Query  781     GGGCTTAGTGCCGCAGCTAACGCATTAAGC  810
               ||||||||||||||||||||||||||||||
Sbjct  236618  GGGCTTAGTGCCGCAGCTAACGCATTAAGC  236647
```

Range 5: 333173 to 333982

Score	Expect	Identities	Gaps	Strand	Frame
1491 bits(807)	0.0()	809/810(99%)	0/810(0%)	Plus/Plus	

Features:

```
Query  1       TGTTTGGTGTTTACACTAGACTGATGAGTTGCGAACGGGTGAGTAACGCGTAGGTAACCT  60
               ||||||||||||||||||||||||||||||||||||||||||||||||||||||||||||
```

```
Sbjct  333173   TGTTTGGTGTTTACACTAGACTGATGAGTTGCGAACGGGTGAGTAACGCGTAGGTAACCT  333232
Query  61       GCCTCATAGCGGGGGATAACTATTGGAAACGATAGCTAATACCGCATAAGAGTAATTAAC  120
                ||||||||||||||||||||||||||||||||||||||||||||||||||||| ||||||
Sbjct  333233   GCCTCATAGCGGGGGATAACTATTGGAAACGATAGCTAATACCGCATAAGAGTGATTAAC  333292
Query  121      ACATGTTAGTTATTTAAAAGGAGCAATTGCTTCACTGTGAGATGGACCTGCGTTGTATTA  180
                ||||||||||||||||||||||||||||||||||||||||||||||||||||||||||||
Sbjct  333293   ACATGTTAGTTATTTAAAAGGAGCAATTGCTTCACTGTGAGATGGACCTGCGTTGTATTA  333352
Query  181      GCTAGTTGGTGAGGTAAAGGCTCACCAAGGCGACGATACATAGCCGACCTGAGAGGGTGA  240
                ||||||||||||||||||||||||||||||||||||||||||||||||||||||||||||
Sbjct  333353   GCTAGTTGGTGAGGTAAAGGCTCACCAAGGCGACGATACATAGCCGACCTGAGAGGGTGA  333412
Query  241      TCGGCCACACTGGGACTGAGACACGGCCCAGACTCCTACGGGAGGCAGCAGTAGGGAATC  300
                ||||||||||||||||||||||||||||||||||||||||||||||||||||||||||||
Sbjct  333413   TCGGCCACACTGGGACTGAGACACGGCCCAGACTCCTACGGGAGGCAGCAGTAGGGAATC  333472
Query  301      TTCGGCAATGGACGGAAGTCTGACCGAGCAACGCCGCGTGAGTGAAGAAGGTTTTCGGAT  360
                ||||||||||||||||||||||||||||||||||||||||||||||||||||||||||||
Sbjct  333473   TTCGGCAATGGACGGAAGTCTGACCGAGCAACGCCGCGTGAGTGAAGAAGGTTTTCGGAT  333532
Query  361      CGTAAAGCTCTGTTGTTAGAGAAGAACGTTGGTAGGAGTGGAAAATCTACCAAGTGACGG  420
                ||||||||||||||||||||||||||||||||||||||||||||||||||||||||||||
Sbjct  333533   CGTAAAGCTCTGTTGTTAGAGAAGAACGTTGGTAGGAGTGGAAAATCTACCAAGTGACGG  333592
Query  421      TAACTAACCAGAAAGGGACGGCTAACTACGTGCCAGCAGCCGCGGTAATACGTAGGTCCC  480
                ||||||||||||||||||||||||||||||||||||||||||||||||||||||||||||
Sbjct  333593   TAACTAACCAGAAAGGGACGGCTAACTACGTGCCAGCAGCCGCGGTAATACGTAGGTCCC  333652
Query  481      GAGCGTTGTCCGGATTTATTGGGCGTAAAGCGAGCGCAGGCGGTTCTTTAAGTCTGAAGT  540
                ||||||||||||||||||||||||||||||||||||||||||||||||||||||||||||
Sbjct  333653   GAGCGTTGTCCGGATTTATTGGGCGTAAAGCGAGCGCAGGCGGTTCTTTAAGTCTGAAGT  333712
Query  541      TAAAGGCAGTGGCTTAACCATTGTACGCTTTGGAAACTGGAGGACTTGAGTGCAGAAGGG  600
                ||||||||||||||||||||||||||||||||||||||||||||||||||||||||||||
Sbjct  333713   TAAAGGCAGTGGCTTAACCATTGTACGCTTTGGAAACTGGAGGACTTGAGTGCAGAAGGG  333772
Query  601      GAGAGTGGAATTCCATGTGTAGCGGTGAAATGCGTAGATATATGGAGGAACACCGGTGGC  660
                ||||||||||||||||||||||||||||||||||||||||||||||||||||||||||||
Sbjct  333773   GAGAGTGGAATTCCATGTGTAGCGGTGAAATGCGTAGATATATGGAGGAACACCGGTGGC  333832
Query  661      GAAAGCGGCTCTCTGGTCTGTAACTGACGCTGAGGCTCGAAAGCGTGGGGAGCAAACAGG  720
                ||||||||||||||||||||||||||||||||||||||||||||||||||||||||||||
Sbjct  333833   GAAAGCGGCTCTCTGGTCTGTAACTGACGCTGAGGCTCGAAAGCGTGGGGAGCAAACAGG  333892
Query  721      ATTAGATACCCTGGTAGTCCACGCCGTAAACGATGAGTGCTAGGTGTTAGGCCCTTTCCG  780
                ||||||||||||||||||||||||||||||||||||||||||||||||||||||||||||
Sbjct  333893   ATTAGATACCCTGGTAGTCCACGCCGTAAACGATGAGTGCTAGGTGTTAGGCCCTTTCCG  333952
Query  781      GGGCTTAGTGCCGCAGCTAACGCATTAAGC  810
                ||||||||||||||||||||||||||||||
Sbjct  333953   GGGCTTAGTGCCGCAGCTAACGCATTAAGC  333982
```

Range 6: 402300 to 403109

Score	Expect	Identities	Gaps	Strand	Frame
1491 bits(807)	0.0()	809/810(99%)	0/810(0%)	Plus/Plus	

Features:

```
Query  1        TGTTTGGTGTTTACACTAGACTGATGAGTTGCGAACGGGTGAGTAACGCGTAGGTAACCT  60
                ||||||||||||||||||||||||||||||||||||||||||||||||||||||||||||
Sbjct  402300   TGTTTGGTGTTTACACTAGACTGATGAGTTGCGAACGGGTGAGTAACGCGTAGGTAACCT  402359
Query  61       GCCTCATAGCGGGGGATAACTATTGGAAACGATAGCTAATACCGCATAAGAGTAATTAAC  120
                ||||||||||||||||||||||||||||||||||||||||||||||||||||| ||||||
Sbjct  402360   GCCTCATAGCGGGGGATAACTATTGGAAACGATAGCTAATACCGCATAAGAGTGATTAAC  402419
Query  121      ACATGTTAGTTATTTAAAAGGAGCAATTGCTTCACTGTGAGATGGACCTGCGTTGTATTA  180
                ||||||||||||||||||||||||||||||||||||||||||||||||||||||||||||
Sbjct  402420   ACATGTTAGTTATTTAAAAGGAGCAATTGCTTCACTGTGAGATGGACCTGCGTTGTATTA  402479
Query  181      GCTAGTTGGTGAGGTAAAGGCTCACCAAGGCGACGATACATAGCCGACCTGAGAGGGTGA  240
                ||||||||||||||||||||||||||||||||||||||||||||||||||||||||||||
Sbjct  402480   GCTAGTTGGTGAGGTAAAGGCTCACCAAGGCGACGATACATAGCCGACCTGAGAGGGTGA  402539
Query  241      TCGGCCACACTGGGACTGAGACACGGCCCAGACTCCTACGGGAGGCAGCAGTAGGGAATC  300
                ||||||||||||||||||||||||||||||||||||||||||||||||||||||||||||
Sbjct  402540   TCGGCCACACTGGGACTGAGACACGGCCCAGACTCCTACGGGAGGCAGCAGTAGGGAATC  402599
Query  301      TTCGGCAATGGACGGAAGTCTGACCGAGCAACGCCGCGTGAGTGAAGAAGGTTTTCGGAT  360
                ||||||||||||||||||||||||||||||||||||||||||||||||||||||||||||
Sbjct  402600   TTCGGCAATGGACGGAAGTCTGACCGAGCAACGCCGCGTGAGTGAAGAAGGTTTTCGGAT  402659
Query  361      CGTAAAGCTCTGTTGTTAGAGAAGAACGTTGGTAGGAGTGGAAAATCTACCAAGTGACGG  420
                ||||||||||||||||||||||||||||||||||||||||||||||||||||||||||||
Sbjct  402660   CGTAAAGCTCTGTTGTTAGAGAAGAACGTTGGTAGGAGTGGAAAATCTACCAAGTGACGG  402719
Query  421      TAACTAACCAGAAAGGGACGGCTAACTACGTGCCAGCAGCCGCGGTAATACGTAGGTCCC  480
                ||||||||||||||||||||||||||||||||||||||||||||||||||||||||||||
Sbjct  402720   TAACTAACCAGAAAGGGACGGCTAACTACGTGCCAGCAGCCGCGGTAATACGTAGGTCCC  402779
Query  481      GAGCGTTGTCCGGATTTATTGGGCGTAAAGCGAGCGCAGGCGGTTCTTTAAGTCTGAAGT  540
                ||||||||||||||||||||||||||||||||||||||||||||||||||||||||||||
Sbjct  402780   GAGCGTTGTCCGGATTTATTGGGCGTAAAGCGAGCGCAGGCGGTTCTTTAAGTCTGAAGT  402839
Query  541      TAAAGGCAGTGGCTTAACCATTGTACGCTTTGGAAACTGGAGGACTTGAGTGCAGAAGGG  600
                ||||||||||||||||||||||||||||||||||||||||||||||||||||||||||||
Sbjct  402840   TAAAGGCAGTGGCTTAACCATTGTACGCTTTGGAAACTGGAGGACTTGAGTGCAGAAGGG  402899
Query  601      GAGAGTGGAATTCCATGTGTAGCGGTGAAATGCGTAGATATATGGAGGAACACCGGTGGC  660
                ||||||||||||||||||||||||||||||||||||||||||||||||||||||||||||
Sbjct  402900   GAGAGTGGAATTCCATGTGTAGCGGTGAAATGCGTAGATATATGGAGGAACACCGGTGGC  402959
Query  661      GAAAGCGGCTCTCTGGTCTGTAACTGACGCTGAGGCTCGAAAGCGTGGGGAGCAAACAGG  720
                ||||||||||||||||||||||||||||||||||||||||||||||||||||||||||||
Sbjct  402960   GAAAGCGGCTCTCTGGTCTGTAACTGACGCTGAGGCTCGAAAGCGTGGGGAGCAAACAGG  403019
Query  721      ATTAGATACCCTGGTAGTCCACGCCGTAAACGATGAGTGCTAGGTGTTAGGCCCTTTCCG  780
                ||||||||||||||||||||||||||||||||||||||||||||||||||||||||||||
Sbjct  403020   ATTAGATACCCTGGTAGTCCACGCCGTAAACGATGAGTGCTAGGTGTTAGGCCCTTTCCG  403079
Query  781      GGGCTTAGTGCCGCAGCTAACGCATTAAGC  810
                ||||||||||||||||||||||||||||||
Sbjct  403080   GGGCTTAGTGCCGCAGCTAACGCATTAAGC  403109
```

Range 7: 2013847 to 2014656

Score	Expect	Identities	Gaps	Strand	Frame
1491 bits(807)	0.0()	809/810(99%)	0/810(0%)	Plus/Plus	

Features:

```
Query  1         TGTTTGGTGTTTACACTAGACTGATGAGTTGCGAACGGGTGAGTAACGCGTAGGTAACCT  60
                 ||||||||||||||||||||||||||||||||||||||||||||||||||||||||||||
Sbjct  2013847   TGTTTGGTGTTTACACTAGACTGATGAGTTGCGAACGGGTGAGTAACGCGTAGGTAACCT  2013906
Query  61        GCCTCATAGCGGGGGATAACTATTGGAAACGATAGCTAATACCGCATAAGAGTAATTAAC  120
                 ||||||||||||||||||||||||||||||||||||||||||||||||||||| ||||||
Sbjct  2013907   GCCTCATAGCGGGGGATAACTATTGGAAACGATAGCTAATACCGCATAAGAGTGATTAAC  2013966
Query  121       ACATGTTAGTTATTTAAAAGGAGCAATTGCTTCACTGTGAGATGGACCTGCGTTGTATTA  180
                 ||||||||||||||||||||||||||||||||||||||||||||||||||||||||||||
Sbjct  2013967   ACATGTTAGTTATTTAAAAGGAGCAATTGCTTCACTGTGAGATGGACCTGCGTTGTATTA  2014026
Query  181       GCTAGTTGGTGAGGTAAAGGCTCACCAAGGCGACGATACATAGCCGACCTGAGAGGGTGA  240
                 ||||||||||||||||||||||||||||||||||||||||||||||||||||||||||||
Sbjct  2014027   GCTAGTTGGTGAGGTAAAGGCTCACCAAGGCGACGATACATAGCCGACCTGAGAGGGTGA  2014086
Query  241       TCGGCCACACTGGGACTGAGACACGGCCCAGACTCCTACGGGAGGCAGCAGTAGGGAATC  300
                 ||||||||||||||||||||||||||||||||||||||||||||||||||||||||||||
Sbjct  2014087   TCGGCCACACTGGGACTGAGACACGGCCCAGACTCCTACGGGAGGCAGCAGTAGGGAATC  2014146
Query  301       TTCGGCAATGGACGGAAGTCTGACCGAGCAACGCCGCGTGAGTGAAGAAGGTTTTCGGAT  360
                 ||||||||||||||||||||||||||||||||||||||||||||||||||||||||||||
Sbjct  2014147   TTCGGCAATGGACGGAAGTCTGACCGAGCAACGCCGCGTGAGTGAAGAAGGTTTTCGGAT  2014206
Query  361       CGTAAAGCTCTGTTGTTAGAGAAGAACGTTGGTAGGAGTGGAAAATCTACCAAGTGACGG  420
```

```
               ||||||||||||||||||||||||||||||||||||||||||||||||||||||||||||
Sbjct  2014207 CGTAAAGCTCTGTTGTTAGAGAAGAACGTTGGTAGGAGTGGAAAATCTACCAAGTGACGG  2014266

Query  421     TAACTAACCAGAAAGGGACGGCTAACTACGTGCCAGCAGCCGCGGTAATACGTAGGTCCC  480
               ||||||||||||||||||||||||||||||||||||||||||||||||||||||||||||
Sbjct  2014267 TAACTAACCAGAAAGGGACGGCTAACTACGTGCCAGCAGCCGCGGTAATACGTAGGTCCC  2014326

Query  481     GAGCGTTGTCCGGATTTATTGGGCGTAAAGCGAGCGCAGGCGGTTCTTTAAGTCTGAAGT  540
               ||||||||||||||||||||||||||||||||||||||||||||||||||||||||||||
Sbjct  2014327 GAGCGTTGTCCGGATTTATTGGGCGTAAAGCGAGCGCAGGCGGTTCTTTAAGTCTGAAGT  2014386

Query  541     TAAAGGCAGTGGCTTAACCATTGTACGCTTTGGAAACTGGAGGACTTGAGTGCAGAAGGG  600
               ||||||||||||||||||||||||||||||||||||||||||||||||||||||||||||
Sbjct  2014387 TAAAGGCAGTGGCTTAACCATTGTACGCTTTGGAAACTGGAGGACTTGAGTGCAGAAGGG  2014446

Query  601     GAGAGTGGAATTCCATGTGTAGCGGTGAAATGCGTAGATATATGGAGGAACACCGGTGGC  660
               ||||||||||||||||||||||||||||||||||||||||||||||||||||||||||||
Sbjct  2014447 GAGAGTGGAATTCCATGTGTAGCGGTGAAATGCGTAGATATATGGAGGAACACCGGTGGC  2014506

Query  661     GAAAGCGGCTCTCTGGTCTGTAACTGACGCTGAGGCTCGAAAGCGTGGGGAGCAAACAGG  720
               ||||||||||||||||||||||||||||||||||||||||||||||||||||||||||||
Sbjct  2014507 GAAAGCGGCTCTCTGGTCTGTAACTGACGCTGAGGCTCGAAAGCGTGGGGAGCAAACAGG  2014566

Query  721     ATTAGATACCCTGGTAGTCCACGCCGTAAACGATGAGTGCTAGGTGTTAGGCCCTTTCCG  780
               ||||||||||||||||||||||||||||||||||||||||||||||||||||||||||||
Sbjct  2014567 ATTAGATACCCTGGTAGTCCACGCCGTAAACGATGAGTGCTAGGTGTTAGGCCCTTTCCG  2014626

Query  781     GGGCTTAGTGCCGCAGCTAACGCATTAAGC  810
               ||||||||||||||||||||||||||||||
Sbjct  2014627 GGGCTTAGTGCCGCAGCTAACGCATTAAGC  2014656
```

Streptococcus agalactiae strain QX1503 16S ribosomal RNA gene, partial sequence
Sequence ID: **MF113268.1** Length: 1510 Number of Matches: 1
Range 1: 66 to 875

Score	Expect	Identities	Gaps	Strand	Frame
1496 bits(810)	0.0()	810/810(100%)	0/810(0%)	Plus/Plus	

Features:

```
Query  1    TGTTTGGTGTTTACACTAGACTGATGAGTTGCGAACGGGTGAGTAACGCGTAGGTAACCT  60
            ||||||||||||||||||||||||||||||||||||||||||||||||||||||||||||
Sbjct  66   TGTTTGGTGTTTACACTAGACTGATGAGTTGCGAACGGGTGAGTAACGCGTAGGTAACCT  125

Query  61   GCCTCATAGCGGGGGATAACTATTGGAAACGATAGCTAATACCGCATAAGAGTAATTAAC  120
            ||||||||||||||||||||||||||||||||||||||||||||||||||||||||||||
Sbjct  126  GCCTCATAGCGGGGGATAACTATTGGAAACGATAGCTAATACCGCATAAGAGTAATTAAC  185

Query  121  ACATGTTAGTTATTTAAAAGGAGCAATTGCTTCACTGTGAGATGGACCTGCGTTGTATTA  180
            ||||||||||||||||||||||||||||||||||||||||||||||||||||||||||||
Sbjct  186  ACATGTTAGTTATTTAAAAGGAGCAATTGCTTCACTGTGAGATGGACCTGCGTTGTATTA  245

Query  181  GCTAGTTGGTGAGGTAAAGGCTCACCAAGGCGACGATACATAGCCGACCTGAGAGGGTGA  240
            ||||||||||||||||||||||||||||||||||||||||||||||||||||||||||||
Sbjct  246  GCTAGTTGGTGAGGTAAAGGCTCACCAAGGCGACGATACATAGCCGACCTGAGAGGGTGA  305

Query  241  TCGGCCACACTGGGACTGAGACACGGCCCAGACTCCTACGGGAGGCAGCAGTAGGGAATC  300
            ||||||||||||||||||||||||||||||||||||||||||||||||||||||||||||
Sbjct  306  TCGGCCACACTGGGACTGAGACACGGCCCAGACTCCTACGGGAGGCAGCAGTAGGGAATC  365

Query  301  TTCGGCAATGGACGGAAGTCTGACCGAGCAACGCCGCGTGAGTGAAGAAGGTTTTCGGAT  360
            ||||||||||||||||||||||||||||||||||||||||||||||||||||||||||||
Sbjct  366  TTCGGCAATGGACGGAAGTCTGACCGAGCAACGCCGCGTGAGTGAAGAAGGTTTTCGGAT  425

Query  361  CGTAAAGCTCTGTTGTTAGAGAAGAACGTTGGTAGGAGTGGAAAATCTACCAAGTGACGG  420
            ||||||||||||||||||||||||||||||||||||||||||||||||||||||||||||
Sbjct  426  CGTAAAGCTCTGTTGTTAGAGAAGAACGTTGGTAGGAGTGGAAAATCTACCAAGTGACGG  485

Query  421  TAACTAACCAGAAAGGGACGGCTAACTACGTGCCAGCAGCCGCGGTAATACGTAGGTCCC  480
            ||||||||||||||||||||||||||||||||||||||||||||||||||||||||||||
Sbjct  486  TAACTAACCAGAAAGGGACGGCTAACTACGTGCCAGCAGCCGCGGTAATACGTAGGTCCC  545

Query  481  GAGCGTTGTCCGGATTTATTGGGCGTAAAGCGAGCGCAGGCGGTTCTTTAAGTCTGAAGT  540
            ||||||||||||||||||||||||||||||||||||||||||||||||||||||||||||
Sbjct  546  GAGCGTTGTCCGGATTTATTGGGCGTAAAGCGAGCGCAGGCGGTTCTTTAAGTCTGAAGT  605

Query  541  TAAAGGCAGTGGCTTAACCATTGTACGCTTTGGAAACTGGAGGACTTGAGTGCAGAAGGG  600
            ||||||||||||||||||||||||||||||||||||||||||||||||||||||||||||
Sbjct  606  TAAAGGCAGTGGCTTAACCATTGTACGCTTTGGAAACTGGAGGACTTGAGTGCAGAAGGG  665

Query  601  GAGAGTGGAATTCCATGTGTAGCGGTGAAATGCGTAGATATATGGAGGAACACCGGTGGC  660
            ||||||||||||||||||||||||||||||||||||||||||||||||||||||||||||
Sbjct  666  GAGAGTGGAATTCCATGTGTAGCGGTGAAATGCGTAGATATATGGAGGAACACCGGTGGC  725

Query  661  GAAAGCGGCTCTCTGGTCTGTAACTGACGCTGAGGCTCGAAAGCGTGGGGAGCAAACAGG  720
            ||||||||||||||||||||||||||||||||||||||||||||||||||||||||||||
Sbjct  726  GAAAGCGGCTCTCTGGTCTGTAACTGACGCTGAGGCTCGAAAGCGTGGGGAGCAAACAGG  785

Query  721  ATTAGATACCCTGGTAGTCCACGCCGTAAACGATGAGTGCTAGGTGTTAGGCCCTTTCCG  780
            ||||||||||||||||||||||||||||||||||||||||||||||||||||||||||||
Sbjct  786  ATTAGATACCCTGGTAGTCCACGCCGTAAACGATGAGTGCTAGGTGTTAGGCCCTTTCCG  845

Query  781  GGGCTTAGTGCCGCAGCTAACGCATTAAGC  810
            ||||||||||||||||||||||||||||||
Sbjct  846  GGGCTTAGTGCCGCAGCTAACGCATTAAGC  875
```

Streptococcus agalactiae strain HN1517 16S ribosomal RNA gene, partial sequence
Sequence ID: **MF113266.1** Length: 1510 Number of Matches: 1
Range 1: 66 to 875

Score	Expect	Identities	Gaps	Strand	Frame
1496 bits(810)	0.0()	810/810(100%)	0/810(0%)	Plus/Plus	

Features:

```
Query  1    TGTTTGGTGTTTACACTAGACTGATGAGTTGCGAACGGGTGAGTAACGCGTAGGTAACCT  60
            ||||||||||||||||||||||||||||||||||||||||||||||||||||||||||||
Sbjct  66   TGTTTGGTGTTTACACTAGACTGATGAGTTGCGAACGGGTGAGTAACGCGTAGGTAACCT  125

Query  61   GCCTCATAGCGGGGGATAACTATTGGAAACGATAGCTAATACCGCATAAGAGTAATTAAC  120
            ||||||||||||||||||||||||||||||||||||||||||||||||||||||||||||
Sbjct  126  GCCTCATAGCGGGGGATAACTATTGGAAACGATAGCTAATACCGCATAAGAGTAATTAAC  185

Query  121  ACATGTTAGTTATTTAAAAGGAGCAATTGCTTCACTGTGAGATGGACCTGCGTTGTATTA  180
            ||||||||||||||||||||||||||||||||||||||||||||||||||||||||||||
Sbjct  186  ACATGTTAGTTATTTAAAAGGAGCAATTGCTTCACTGTGAGATGGACCTGCGTTGTATTA  245

Query  181  GCTAGTTGGTGAGGTAAAGGCTCACCAAGGCGACGATACATAGCCGACCTGAGAGGGTGA  240
            ||||||||||||||||||||||||||||||||||||||||||||||||||||||||||||
Sbjct  246  GCTAGTTGGTGAGGTAAAGGCTCACCAAGGCGACGATACATAGCCGACCTGAGAGGGTGA  305

Query  241  TCGGCCACACTGGGACTGAGACACGGCCCAGACTCCTACGGGAGGCAGCAGTAGGGAATC  300
            ||||||||||||||||||||||||||||||||||||||||||||||||||||||||||||
Sbjct  306  TCGGCCACACTGGGACTGAGACACGGCCCAGACTCCTACGGGAGGCAGCAGTAGGGAATC  365

Query  301  TTCGGCAATGGACGGAAGTCTGACCGAGCAACGCCGCGTGAGTGAAGAAGGTTTTCGGAT  360
            ||||||||||||||||||||||||||||||||||||||||||||||||||||||||||||
Sbjct  366  TTCGGCAATGGACGGAAGTCTGACCGAGCAACGCCGCGTGAGTGAAGAAGGTTTTCGGAT  425

Query  361  CGTAAAGCTCTGTTGTTAGAGAAGAACGTTGGTAGGAGTGGAAAATCTACCAAGTGACGG  420
            ||||||||||||||||||||||||||||||||||||||||||||||||||||||||||||
Sbjct  426  CGTAAAGCTCTGTTGTTAGAGAAGAACGTTGGTAGGAGTGGAAAATCTACCAAGTGACGG  485

Query  421  TAACTAACCAGAAAGGGACGGCTAACTACGTGCCAGCAGCCGCGGTAATACGTAGGTCCC  480
            ||||||||||||||||||||||||||||||||||||||||||||||||||||||||||||
Sbjct  486  TAACTAACCAGAAAGGGACGGCTAACTACGTGCCAGCAGCCGCGGTAATACGTAGGTCCC  545

Query  481  GAGCGTTGTCCGGATTTATTGGGCGTAAAGCGAGCGCAGGCGGTTCTTTAAGTCTGAAGT  540
            ||||||||||||||||||||||||||||||||||||||||||||||||||||||||||||
Sbjct  546  GAGCGTTGTCCGGATTTATTGGGCGTAAAGCGAGCGCAGGCGGTTCTTTAAGTCTGAAGT  605

Query  541  TAAAGGCAGTGGCTTAACCATTGTACGCTTTGGAAACTGGAGGACTTGAGTGCAGAAGGG  600
            ||||||||||||||||||||||||||||||||||||||||||||||||||||||||||||
Sbjct  606  TAAAGGCAGTGGCTTAACCATTGTACGCTTTGGAAACTGGAGGACTTGAGTGCAGAAGGG  665
```

```
Query  601   GAGAGTGGAATTCCATGTGTAGCGGTGAAATGCGTAGATATATGGAGGAACACCGGTGGC  660
             ||||||||||||||||||||||||||||||||||||||||||||||||||||||||||||
Sbjct  666   GAGAGTGGAATTCCATGTGTAGCGGTGAAATGCGTAGATATATGGAGGAACACCGGTGGC  725
Query  661   GAAAGCGGCTCTCTGGTCTGTAACTGACGCTGAGGCTCGAAAGCGTGGGGAGCAAACAGG  720
             ||||||||||||||||||||||||||||||||||||||||||||||||||||||||||||
Sbjct  726   GAAAGCGGCTCTCTGGTCTGTAACTGACGCTGAGGCTCGAAAGCGTGGGGAGCAAACAGG  785
Query  721   ATTAGATACCCTGGTAGTCCACGCCGTAAACGATGAGTGCTAGGTGTTAGGCCCTTTCCG  780
             ||||||||||||||||||||||||||||||||||||||||||||||||||||||||||||
Sbjct  786   ATTAGATACCCTGGTAGTCCACGCCGTAAACGATGAGTGCTAGGTGTTAGGCCCTTTCCG  845
Query  781   GGGCTTAGTGCCGCAGCTAACGCATTAAGC  810
             ||||||||||||||||||||||||||||||
Sbjct  846   GGGCTTAGTGCCGCAGCTAACGCATTAAGC  875
```

Streptococcus agalactiae strain HN1503 16S ribosomal RNA gene, partial sequence
Sequence ID: **MF113265.1** Length: 1510 Number of Matches: 1
Range 1: 66 to 875

Score	Expect	Identities	Gaps	Strand	Frame
1496 bits(810)	0.0()	810/810(100%)	0/810(0%)	Plus/Plus	

Features:

```
Query  1     TGTTTGGTGTTTACACTAGACTGATGAGTTGCGAACGGGTGAGTAACGCGTAGGTAACCT  60
             ||||||||||||||||||||||||||||||||||||||||||||||||||||||||||||
Sbjct  66    TGTTTGGTGTTTACACTAGACTGATGAGTTGCGAACGGGTGAGTAACGCGTAGGTAACCT  125
Query  61    GCCTCATAGCGGGGGATAACTATTGGAAACGATAGCTAATACCGCATAAGAGTAATTAAC  120
             ||||||||||||||||||||||||||||||||||||||||||||||||||||||||||||
Sbjct  126   GCCTCATAGCGGGGGATAACTATTGGAAACGATAGCTAATACCGCATAAGAGTAATTAAC  185
Query  121   ACATGTTAGTTATTTAAAAGGAGCAATTGCTTCACTGTGAGATGGACCTGCGTTGTATTA  180
             ||||||||||||||||||||||||||||||||||||||||||||||||||||||||||||
Sbjct  186   ACATGTTAGTTATTTAAAAGGAGCAATTGCTTCACTGTGAGATGGACCTGCGTTGTATTA  245
Query  181   GCTAGTTGGTGAGGTAAAGGCTCACCAAGGCGACGATACATAGCCGACCTGAGAGGGTGA  240
             ||||||||||||||||||||||||||||||||||||||||||||||||||||||||||||
Sbjct  246   GCTAGTTGGTGAGGTAAAGGCTCACCAAGGCGACGATACATAGCCGACCTGAGAGGGTGA  305
Query  241   TCGGCCACACTGGGACTGAGACACGGCCCAGACTCCTACGGGAGGCAGCAGTAGGGAATC  300
             ||||||||||||||||||||||||||||||||||||||||||||||||||||||||||||
Sbjct  306   TCGGCCACACTGGGACTGAGACACGGCCCAGACTCCTACGGGAGGCAGCAGTAGGGAATC  365
Query  301   TTCGGCAATGGACGGAAGTCTGACCGAGCAACGCCGCGTGAGTGAAGAAGGTTTTCGGAT  360
             ||||||||||||||||||||||||||||||||||||||||||||||||||||||||||||
Sbjct  366   TTCGGCAATGGACGGAAGTCTGACCGAGCAACGCCGCGTGAGTGAAGAAGGTTTTCGGAT  425
Query  361   CGTAAAGCTCTGTTGTTAGAGAAGAACGTTGGTAGGAGTGGAAAATCTACCAAGTGACGG  420
             ||||||||||||||||||||||||||||||||||||||||||||||||||||||||||||
Sbjct  426   CGTAAAGCTCTGTTGTTAGAGAAGAACGTTGGTAGGAGTGGAAAATCTACCAAGTGACGG  485
Query  421   TAACTAACCAGAAAGGGACGGCTAACTACGTGCCAGCAGCCGCGGTAATACGTAGGTCCC  480
             ||||||||||||||||||||||||||||||||||||||||||||||||||||||||||||
Sbjct  486   TAACTAACCAGAAAGGGACGGCTAACTACGTGCCAGCAGCCGCGGTAATACGTAGGTCCC  545
Query  481   GAGCGTTGTCCGGATTTATTGGGCGTAAAGCGAGCGCAGGCGGTTCTTTAAGTCTGAAGT  540
             ||||||||||||||||||||||||||||||||||||||||||||||||||||||||||||
Sbjct  546   GAGCGTTGTCCGGATTTATTGGGCGTAAAGCGAGCGCAGGCGGTTCTTTAAGTCTGAAGT  605
Query  541   TAAAGGCAGTGGCTTAACCATTGTACGCTTTGGAAACTGGAGGACTTGAGTGCAGAAGGG  600
             ||||||||||||||||||||||||||||||||||||||||||||||||||||||||||||
Sbjct  606   TAAAGGCAGTGGCTTAACCATTGTACGCTTTGGAAACTGGAGGACTTGAGTGCAGAAGGG  665
Query  601   GAGAGTGGAATTCCATGTGTAGCGGTGAAATGCGTAGATATATGGAGGAACACCGGTGGC  660
             ||||||||||||||||||||||||||||||||||||||||||||||||||||||||||||
Sbjct  666   GAGAGTGGAATTCCATGTGTAGCGGTGAAATGCGTAGATATATGGAGGAACACCGGTGGC  725
Query  661   GAAAGCGGCTCTCTGGTCTGTAACTGACGCTGAGGCTCGAAAGCGTGGGGAGCAAACAGG  720
             ||||||||||||||||||||||||||||||||||||||||||||||||||||||||||||
Sbjct  726   GAAAGCGGCTCTCTGGTCTGTAACTGACGCTGAGGCTCGAAAGCGTGGGGAGCAAACAGG  785
Query  721   ATTAGATACCCTGGTAGTCCACGCCGTAAACGATGAGTGCTAGGTGTTAGGCCCTTTCCG  780
             ||||||||||||||||||||||||||||||||||||||||||||||||||||||||||||
Sbjct  786   ATTAGATACCCTGGTAGTCCACGCCGTAAACGATGAGTGCTAGGTGTTAGGCCCTTTCCG  845
Query  781   GGGCTTAGTGCCGCAGCTAACGCATTAAGC  810
             ||||||||||||||||||||||||||||||
Sbjct  846   GGGCTTAGTGCCGCAGCTAACGCATTAAGC  875
```

Streptococcus agalactiae strain GXN1417 16S ribosomal RNA gene, partial sequence
Sequence ID: **MF113264.1** Length: 1510 Number of Matches: 1
Range 1: 66 to 875

Score	Expect	Identities	Gaps	Strand	Frame
1496 bits(810)	0.0()	810/810(100%)	0/810(0%)	Plus/Plus	

Features:

```
Query  1     TGTTTGGTGTTTACACTAGACTGATGAGTTGCGAACGGGTGAGTAACGCGTAGGTAACCT  60
             ||||||||||||||||||||||||||||||||||||||||||||||||||||||||||||
Sbjct  66    TGTTTGGTGTTTACACTAGACTGATGAGTTGCGAACGGGTGAGTAACGCGTAGGTAACCT  125
Query  61    GCCTCATAGCGGGGGATAACTATTGGAAACGATAGCTAATACCGCATAAGAGTAATTAAC  120
             ||||||||||||||||||||||||||||||||||||||||||||||||||||||||||||
Sbjct  126   GCCTCATAGCGGGGGATAACTATTGGAAACGATAGCTAATACCGCATAAGAGTAATTAAC  185
Query  121   ACATGTTAGTTATTTAAAAGGAGCAATTGCTTCACTGTGAGATGGACCTGCGTTGTATTA  180
             ||||||||||||||||||||||||||||||||||||||||||||||||||||||||||||
Sbjct  186   ACATGTTAGTTATTTAAAAGGAGCAATTGCTTCACTGTGAGATGGACCTGCGTTGTATTA  245
Query  181   GCTAGTTGGTGAGGTAAAGGCTCACCAAGGCGACGATACATAGCCGACCTGAGAGGGTGA  240
             ||||||||||||||||||||||||||||||||||||||||||||||||||||||||||||
Sbjct  246   GCTAGTTGGTGAGGTAAAGGCTCACCAAGGCGACGATACATAGCCGACCTGAGAGGGTGA  305
Query  241   TCGGCCACACTGGGACTGAGACACGGCCCAGACTCCTACGGGAGGCAGCAGTAGGGAATC  300
             ||||||||||||||||||||||||||||||||||||||||||||||||||||||||||||
Sbjct  306   TCGGCCACACTGGGACTGAGACACGGCCCAGACTCCTACGGGAGGCAGCAGTAGGGAATC  365
Query  301   TTCGGCAATGGACGGAAGTCTGACCGAGCAACGCCGCGTGAGTGAAGAAGGTTTTCGGAT  360
             ||||||||||||||||||||||||||||||||||||||||||||||||||||||||||||
Sbjct  366   TTCGGCAATGGACGGAAGTCTGACCGAGCAACGCCGCGTGAGTGAAGAAGGTTTTCGGAT  425
Query  361   CGTAAAGCTCTGTTGTTAGAGAAGAACGTTGGTAGGAGTGGAAAATCTACCAAGTGACGG  420
             ||||||||||||||||||||||||||||||||||||||||||||||||||||||||||||
Sbjct  426   CGTAAAGCTCTGTTGTTAGAGAAGAACGTTGGTAGGAGTGGAAAATCTACCAAGTGACGG  485
Query  421   TAACTAACCAGAAAGGGACGGCTAACTACGTGCCAGCAGCCGCGGTAATACGTAGGTCCC  480
             ||||||||||||||||||||||||||||||||||||||||||||||||||||||||||||
Sbjct  486   TAACTAACCAGAAAGGGACGGCTAACTACGTGCCAGCAGCCGCGGTAATACGTAGGTCCC  545
Query  481   GAGCGTTGTCCGGATTTATTGGGCGTAAAGCGAGCGCAGGCGGTTCTTTAAGTCTGAAGT  540
             ||||||||||||||||||||||||||||||||||||||||||||||||||||||||||||
Sbjct  546   GAGCGTTGTCCGGATTTATTGGGCGTAAAGCGAGCGCAGGCGGTTCTTTAAGTCTGAAGT  605
Query  541   TAAAGGCAGTGGCTTAACCATTGTACGCTTTGGAAACTGGAGGACTTGAGTGCAGAAGGG  600
             ||||||||||||||||||||||||||||||||||||||||||||||||||||||||||||
Sbjct  606   TAAAGGCAGTGGCTTAACCATTGTACGCTTTGGAAACTGGAGGACTTGAGTGCAGAAGGG  665
Query  601   GAGAGTGGAATTCCATGTGTAGCGGTGAAATGCGTAGATATATGGAGGAACACCGGTGGC  660
             ||||||||||||||||||||||||||||||||||||||||||||||||||||||||||||
Sbjct  666   GAGAGTGGAATTCCATGTGTAGCGGTGAAATGCGTAGATATATGGAGGAACACCGGTGGC  725
Query  661   GAAAGCGGCTCTCTGGTCTGTAACTGACGCTGAGGCTCGAAAGCGTGGGGAGCAAACAGG  720
             ||||||||||||||||||||||||||||||||||||||||||||||||||||||||||||
Sbjct  726   GAAAGCGGCTCTCTGGTCTGTAACTGACGCTGAGGCTCGAAAGCGTGGGGAGCAAACAGG  785
Query  721   ATTAGATACCCTGGTAGTCCACGCCGTAAACGATGAGTGCTAGGTGTTAGGCCCTTTCCG  780
             ||||||||||||||||||||||||||||||||||||||||||||||||||||||||||||
Sbjct  786   ATTAGATACCCTGGTAGTCCACGCCGTAAACGATGAGTGCTAGGTGTTAGGCCCTTTCCG  845
Query  781   GGGCTTAGTGCCGCAGCTAACGCATTAAGC  810
```

```
              ||||||||||||||||||||||||||||||
Sbjct  846   GGGCTTAGTGCCGCAGCTAACGCATTAAGC  875
```

Streptococcus agalactiae strain GXN1402 16S ribosomal RNA gene, partial sequence
Sequence ID: **MF113263.1** Length: 1510 Number of Matches: 1
Range 1: 66 to 875

Score	Expect	Identities	Gaps	Strand	Frame
1496 bits(810)	0.0()	810/810(100%)	0/810(0%)	Plus/Plus	

Features:

```
Query  1     TGTTTGGTGTTTACACTAGACTGATGAGTTGCGAACGGGTGAGTAACGCGTAGGTAACCT  60
             ||||||||||||||||||||||||||||||||||||||||||||||||||||||||||||
Sbjct  66    TGTTTGGTGTTTACACTAGACTGATGAGTTGCGAACGGGTGAGTAACGCGTAGGTAACCT  125

Query  61    GCCTCATAGCGGGGGATAACTATTGGAAACGATAGCTAATACCGCATAAGAGTAATTAAC  120
             ||||||||||||||||||||||||||||||||||||||||||||||||||||||||||||
Sbjct  126   GCCTCATAGCGGGGGATAACTATTGGAAACGATAGCTAATACCGCATAAGAGTAATTAAC  185

Query  121   ACATGTTAGTTATTTAAAAGGAGCAATTGCTTCACTGTGAGATGGACCTGCGTTGTATTA  180
             ||||||||||||||||||||||||||||||||||||||||||||||||||||||||||||
Sbjct  186   ACATGTTAGTTATTTAAAAGGAGCAATTGCTTCACTGTGAGATGGACCTGCGTTGTATTA  245

Query  181   GCTAGTTGGTGAGGTAAAGGCTCACCAAGGCGACGATACATAGCCGACCTGAGAGGGTGA  240
             ||||||||||||||||||||||||||||||||||||||||||||||||||||||||||||
Sbjct  246   GCTAGTTGGTGAGGTAAAGGCTCACCAAGGCGACGATACATAGCCGACCTGAGAGGGTGA  305

Query  241   TCGGCCACACTGGGACTGAGACACGGCCCAGACTCCTACGGGAGGCAGCAGTAGGGAATC  300
             ||||||||||||||||||||||||||||||||||||||||||||||||||||||||||||
Sbjct  306   TCGGCCACACTGGGACTGAGACACGGCCCAGACTCCTACGGGAGGCAGCAGTAGGGAATC  365

Query  301   TTCGGCAATGGACGGAAGTCTGACCGAGCAACGCCGCGTGAGTGAAGAAGGTTTTCGGAT  360
             ||||||||||||||||||||||||||||||||||||||||||||||||||||||||||||
Sbjct  366   TTCGGCAATGGACGGAAGTCTGACCGAGCAACGCCGCGTGAGTGAAGAAGGTTTTCGGAT  425

Query  361   CGTAAAGCTCTGTTGTTAGAGAAGAACGTTGGTAGGAGTGGAAAATCTACCAAGTGACGG  420
             ||||||||||||||||||||||||||||||||||||||||||||||||||||||||||||
Sbjct  426   CGTAAAGCTCTGTTGTTAGAGAAGAACGTTGGTAGGAGTGGAAAATCTACCAAGTGACGG  485

Query  421   TAACTAACCAGAAAGGGACGGCTAACTACGTGCCAGCAGCCGCGGTAATACGTAGGTCCC  480
             ||||||||||||||||||||||||||||||||||||||||||||||||||||||||||||
Sbjct  486   TAACTAACCAGAAAGGGACGGCTAACTACGTGCCAGCAGCCGCGGTAATACGTAGGTCCC  545

Query  481   GAGCGTTGTCCGGATTTATTGGGCGTAAAGCGAGCGCAGGCGGTTCTTTAAGTCTGAAGT  540
             ||||||||||||||||||||||||||||||||||||||||||||||||||||||||||||
Sbjct  546   GAGCGTTGTCCGGATTTATTGGGCGTAAAGCGAGCGCAGGCGGTTCTTTAAGTCTGAAGT  605

Query  541   TAAAGGCAGTGGCTTAACCATTGTACGCTTTGGAAACTGGAGGACTTGAGTGCAGAAGGG  600
             ||||||||||||||||||||||||||||||||||||||||||||||||||||||||||||
Sbjct  606   TAAAGGCAGTGGCTTAACCATTGTACGCTTTGGAAACTGGAGGACTTGAGTGCAGAAGGG  665

Query  601   GAGAGTGGAATTCCATGTGTAGCGGTGAAATGCGTAGATATATGGAGGAACACCGGTGGC  660
             ||||||||||||||||||||||||||||||||||||||||||||||||||||||||||||
Sbjct  666   GAGAGTGGAATTCCATGTGTAGCGGTGAAATGCGTAGATATATGGAGGAACACCGGTGGC  725

Query  661   GAAAGCGGCTCTCTGGTCTGTAACTGACGCTGAGGCTCGAAAGCGTGGGGAGCAAACAGG  720
             ||||||||||||||||||||||||||||||||||||||||||||||||||||||||||||
Sbjct  726   GAAAGCGGCTCTCTGGTCTGTAACTGACGCTGAGGCTCGAAAGCGTGGGGAGCAAACAGG  785

Query  721   ATTAGATACCCTGGTAGTCCACGCCGTAAACGATGAGTGCTAGGTGTTAGGCCCTTTCCG  780
             ||||||||||||||||||||||||||||||||||||||||||||||||||||||||||||
Sbjct  786   ATTAGATACCCTGGTAGTCCACGCCGTAAACGATGAGTGCTAGGTGTTAGGCCCTTTCCG  845

Query  781   GGGCTTAGTGCCGCAGCTAACGCATTAAGC  810
             ||||||||||||||||||||||||||||||
Sbjct  846   GGGCTTAGTGCCGCAGCTAACGCATTAAGC  875
```

Streptococcus agalactiae strain GD1221 16S ribosomal RNA gene, partial sequence
Sequence ID: **MF113262.1** Length: 1510 Number of Matches: 1
Range 1: 66 to 875

Score	Expect	Identities	Gaps	Strand	Frame
1496 bits(810)	0.0()	810/810(100%)	0/810(0%)	Plus/Plus	

Features:

```
Query  1     TGTTTGGTGTTTACACTAGACTGATGAGTTGCGAACGGGTGAGTAACGCGTAGGTAACCT  60
             ||||||||||||||||||||||||||||||||||||||||||||||||||||||||||||
Sbjct  66    TGTTTGGTGTTTACACTAGACTGATGAGTTGCGAACGGGTGAGTAACGCGTAGGTAACCT  125

Query  61    GCCTCATAGCGGGGGATAACTATTGGAAACGATAGCTAATACCGCATAAGAGTAATTAAC  120
             ||||||||||||||||||||||||||||||||||||||||||||||||||||||||||||
Sbjct  126   GCCTCATAGCGGGGGATAACTATTGGAAACGATAGCTAATACCGCATAAGAGTAATTAAC  185

Query  121   ACATGTTAGTTATTTAAAAGGAGCAATTGCTTCACTGTGAGATGGACCTGCGTTGTATTA  180
             ||||||||||||||||||||||||||||||||||||||||||||||||||||||||||||
Sbjct  186   ACATGTTAGTTATTTAAAAGGAGCAATTGCTTCACTGTGAGATGGACCTGCGTTGTATTA  245

Query  181   GCTAGTTGGTGAGGTAAAGGCTCACCAAGGCGACGATACATAGCCGACCTGAGAGGGTGA  240
             ||||||||||||||||||||||||||||||||||||||||||||||||||||||||||||
Sbjct  246   GCTAGTTGGTGAGGTAAAGGCTCACCAAGGCGACGATACATAGCCGACCTGAGAGGGTGA  305

Query  241   TCGGCCACACTGGGACTGAGACACGGCCCAGACTCCTACGGGAGGCAGCAGTAGGGAATC  300
             ||||||||||||||||||||||||||||||||||||||||||||||||||||||||||||
Sbjct  306   TCGGCCACACTGGGACTGAGACACGGCCCAGACTCCTACGGGAGGCAGCAGTAGGGAATC  365

Query  301   TTCGGCAATGGACGGAAGTCTGACCGAGCAACGCCGCGTGAGTGAAGAAGGTTTTCGGAT  360
             ||||||||||||||||||||||||||||||||||||||||||||||||||||||||||||
Sbjct  366   TTCGGCAATGGACGGAAGTCTGACCGAGCAACGCCGCGTGAGTGAAGAAGGTTTTCGGAT  425

Query  361   CGTAAAGCTCTGTTGTTAGAGAAGAACGTTGGTAGGAGTGGAAAATCTACCAAGTGACGG  420
             ||||||||||||||||||||||||||||||||||||||||||||||||||||||||||||
Sbjct  426   CGTAAAGCTCTGTTGTTAGAGAAGAACGTTGGTAGGAGTGGAAAATCTACCAAGTGACGG  485

Query  421   TAACTAACCAGAAAGGGACGGCTAACTACGTGCCAGCAGCCGCGGTAATACGTAGGTCCC  480
             ||||||||||||||||||||||||||||||||||||||||||||||||||||||||||||
Sbjct  486   TAACTAACCAGAAAGGGACGGCTAACTACGTGCCAGCAGCCGCGGTAATACGTAGGTCCC  545

Query  481   GAGCGTTGTCCGGATTTATTGGGCGTAAAGCGAGCGCAGGCGGTTCTTTAAGTCTGAAGT  540
             ||||||||||||||||||||||||||||||||||||||||||||||||||||||||||||
Sbjct  546   GAGCGTTGTCCGGATTTATTGGGCGTAAAGCGAGCGCAGGCGGTTCTTTAAGTCTGAAGT  605

Query  541   TAAAGGCAGTGGCTTAACCATTGTACGCTTTGGAAACTGGAGGACTTGAGTGCAGAAGGG  600
             ||||||||||||||||||||||||||||||||||||||||||||||||||||||||||||
Sbjct  606   TAAAGGCAGTGGCTTAACCATTGTACGCTTTGGAAACTGGAGGACTTGAGTGCAGAAGGG  665

Query  601   GAGAGTGGAATTCCATGTGTAGCGGTGAAATGCGTAGATATATGGAGGAACACCGGTGGC  660
             ||||||||||||||||||||||||||||||||||||||||||||||||||||||||||||
Sbjct  666   GAGAGTGGAATTCCATGTGTAGCGGTGAAATGCGTAGATATATGGAGGAACACCGGTGGC  725

Query  661   GAAAGCGGCTCTCTGGTCTGTAACTGACGCTGAGGCTCGAAAGCGTGGGGAGCAAACAGG  720
             ||||||||||||||||||||||||||||||||||||||||||||||||||||||||||||
Sbjct  726   GAAAGCGGCTCTCTGGTCTGTAACTGACGCTGAGGCTCGAAAGCGTGGGGAGCAAACAGG  785

Query  721   ATTAGATACCCTGGTAGTCCACGCCGTAAACGATGAGTGCTAGGTGTTAGGCCCTTTCCG  780
             ||||||||||||||||||||||||||||||||||||||||||||||||||||||||||||
Sbjct  786   ATTAGATACCCTGGTAGTCCACGCCGTAAACGATGAGTGCTAGGTGTTAGGCCCTTTCCG  845

Query  781   GGGCTTAGTGCCGCAGCTAACGCATTAAGC  810
             ||||||||||||||||||||||||||||||
Sbjct  846   GGGCTTAGTGCCGCAGCTAACGCATTAAGC  875
```

Streptococcus agalactiae strain GD1212 16S ribosomal RNA gene, partial sequence
Sequence ID: **MF113261.1** Length: 1510 Number of Matches: 1
Range 1: 66 to 875

Score	Expect	Identities	Gaps	Strand	Frame

1496 bits(810)	0.0()	810/810(100%)	0/810(0%)	Plus/Plus	

Features:

```
Query  1    TGTTTGGTGTTTACACTAGACTGATGAGTTGCGAACGGGTGAGTAACGCGTAGGTAACCT  60
            ||||||||||||||||||||||||||||||||||||||||||||||||||||||||||||
Sbjct  66   TGTTTGGTGTTTACACTAGACTGATGAGTTGCGAACGGGTGAGTAACGCGTAGGTAACCT  125

Query  61   GCCTCATAGCGGGGGATAACTATTGGAAACGATAGCTAATACCGCATAAGAGTAATTAAC  120
            ||||||||||||||||||||||||||||||||||||||||||||||||||||||||||||
Sbjct  126  GCCTCATAGCGGGGGATAACTATTGGAAACGATAGCTAATACCGCATAAGAGTAATTAAC  185

Query  121  ACATGTTAGTTATTTAAAAGGAGCAATTGCTTCACTGTGAGATGGACCTGCGTTGTATTA  180
            ||||||||||||||||||||||||||||||||||||||||||||||||||||||||||||
Sbjct  186  ACATGTTAGTTATTTAAAAGGAGCAATTGCTTCACTGTGAGATGGACCTGCGTTGTATTA  245

Query  181  GCTAGTTGGTGAGGTAAAGGCTCACCAAGGCGACGATACATAGCCGACCTGAGAGGGTGA  240
            ||||||||||||||||||||||||||||||||||||||||||||||||||||||||||||
Sbjct  246  GCTAGTTGGTGAGGTAAAGGCTCACCAAGGCGACGATACATAGCCGACCTGAGAGGGTGA  305

Query  241  TCGGCCACACTGGGACTGAGACACGGCCCAGACTCCTACGGGAGGCAGCAGTAGGGAATC  300
            ||||||||||||||||||||||||||||||||||||||||||||||||||||||||||||
Sbjct  306  TCGGCCACACTGGGACTGAGACACGGCCCAGACTCCTACGGGAGGCAGCAGTAGGGAATC  365

Query  301  TTCGGCAATGGACGGAAGTCTGACCGAGCAACGCCGCGTGAGTGAAGAAGGTTTTCGGAT  360
            ||||||||||||||||||||||||||||||||||||||||||||||||||||||||||||
Sbjct  366  TTCGGCAATGGACGGAAGTCTGACCGAGCAACGCCGCGTGAGTGAAGAAGGTTTTCGGAT  425

Query  361  CGTAAAGCTCTGTTGTTAGAGAAGAACGTTGGTAGGAGTGGAAAATCTACCAAGTGACGG  420
            ||||||||||||||||||||||||||||||||||||||||||||||||||||||||||||
Sbjct  426  CGTAAAGCTCTGTTGTTAGAGAAGAACGTTGGTAGGAGTGGAAAATCTACCAAGTGACGG  485

Query  421  TAACTAACCAGAAAGGGACGGCTAACTACGTGCCAGCAGCCGCGGTAATACGTAGGTCCC  480
            ||||||||||||||||||||||||||||||||||||||||||||||||||||||||||||
Sbjct  486  TAACTAACCAGAAAGGGACGGCTAACTACGTGCCAGCAGCCGCGGTAATACGTAGGTCCC  545

Query  481  GAGCGTTGTCCGGATTTATTGGGCGTAAAGCGAGCGCAGGCGGTTCTTTAAGTCTGAAGT  540
            ||||||||||||||||||||||||||||||||||||||||||||||||||||||||||||
Sbjct  546  GAGCGTTGTCCGGATTTATTGGGCGTAAAGCGAGCGCAGGCGGTTCTTTAAGTCTGAAGT  605

Query  541  TAAAGGCAGTGGCTTAACCATTGTACGCTTTGGAAACTGGAGGACTTGAGTGCAGAAGGG  600
            ||||||||||||||||||||||||||||||||||||||||||||||||||||||||||||
Sbjct  606  TAAAGGCAGTGGCTTAACCATTGTACGCTTTGGAAACTGGAGGACTTGAGTGCAGAAGGG  665

Query  601  GAGAGTGGAATTCCATGTGTAGCGGTGAAATGCGTAGATATATGGAGGAACACCGGTGGC  660
            ||||||||||||||||||||||||||||||||||||||||||||||||||||||||||||
Sbjct  666  GAGAGTGGAATTCCATGTGTAGCGGTGAAATGCGTAGATATATGGAGGAACACCGGTGGC  725

Query  661  GAAAGCGGCTCTCTGGTCTGTAACTGACGCTGAGGCTCGAAAGCGTGGGGAGCAAACAGG  720
            ||||||||||||||||||||||||||||||||||||||||||||||||||||||||||||
Sbjct  726  GAAAGCGGCTCTCTGGTCTGTAACTGACGCTGAGGCTCGAAAGCGTGGGGAGCAAACAGG  785

Query  721  ATTAGATACCCTGGTAGTCCACGCCGTAAACGATGAGTGCTAGGTGTTAGGCCCTTTCCG  780
            ||||||||||||||||||||||||||||||||||||||||||||||||||||||||||||
Sbjct  786  ATTAGATACCCTGGTAGTCCACGCCGTAAACGATGAGTGCTAGGTGTTAGGCCCTTTCCG  845

Query  781  GGGCTTAGTGCCGCAGCTAACGCATTAAGC  810
            ||||||||||||||||||||||||||||||
Sbjct  846  GGGCTTAGTGCCGCAGCTAACGCATTAAGC  875
```

Streptococcus agalactiae strain NYD007 16S ribosomal RNA gene, partial sequence
Sequence ID: **MF113259.1** Length: 1510 Number of Matches: 1
Range 1: 66 to 875

Score	Expect	Identities	Gaps	Strand	Frame
1496 bits(810)	0.0()	810/810(100%)	0/810(0%)	Plus/Plus	

Features:

```
Query  1    TGTTTGGTGTTTACACTAGACTGATGAGTTGCGAACGGGTGAGTAACGCGTAGGTAACCT  60
            ||||||||||||||||||||||||||||||||||||||||||||||||||||||||||||
Sbjct  66   TGTTTGGTGTTTACACTAGACTGATGAGTTGCGAACGGGTGAGTAACGCGTAGGTAACCT  125

Query  61   GCCTCATAGCGGGGGATAACTATTGGAAACGATAGCTAATACCGCATAAGAGTAATTAAC  120
            ||||||||||||||||||||||||||||||||||||||||||||||||||||||||||||
Sbjct  126  GCCTCATAGCGGGGGATAACTATTGGAAACGATAGCTAATACCGCATAAGAGTAATTAAC  185

Query  121  ACATGTTAGTTATTTAAAAGGAGCAATTGCTTCACTGTGAGATGGACCTGCGTTGTATTA  180
            ||||||||||||||||||||||||||||||||||||||||||||||||||||||||||||
Sbjct  186  ACATGTTAGTTATTTAAAAGGAGCAATTGCTTCACTGTGAGATGGACCTGCGTTGTATTA  245

Query  181  GCTAGTTGGTGAGGTAAAGGCTCACCAAGGCGACGATACATAGCCGACCTGAGAGGGTGA  240
            ||||||||||||||||||||||||||||||||||||||||||||||||||||||||||||
Sbjct  246  GCTAGTTGGTGAGGTAAAGGCTCACCAAGGCGACGATACATAGCCGACCTGAGAGGGTGA  305

Query  241  TCGGCCACACTGGGACTGAGACACGGCCCAGACTCCTACGGGAGGCAGCAGTAGGGAATC  300
            ||||||||||||||||||||||||||||||||||||||||||||||||||||||||||||
Sbjct  306  TCGGCCACACTGGGACTGAGACACGGCCCAGACTCCTACGGGAGGCAGCAGTAGGGAATC  365

Query  301  TTCGGCAATGGACGGAAGTCTGACCGAGCAACGCCGCGTGAGTGAAGAAGGTTTTCGGAT  360
            ||||||||||||||||||||||||||||||||||||||||||||||||||||||||||||
Sbjct  366  TTCGGCAATGGACGGAAGTCTGACCGAGCAACGCCGCGTGAGTGAAGAAGGTTTTCGGAT  425

Query  361  CGTAAAGCTCTGTTGTTAGAGAAGAACGTTGGTAGGAGTGGAAAATCTACCAAGTGACGG  420
            ||||||||||||||||||||||||||||||||||||||||||||||||||||||||||||
Sbjct  426  CGTAAAGCTCTGTTGTTAGAGAAGAACGTTGGTAGGAGTGGAAAATCTACCAAGTGACGG  485

Query  421  TAACTAACCAGAAAGGGACGGCTAACTACGTGCCAGCAGCCGCGGTAATACGTAGGTCCC  480
            ||||||||||||||||||||||||||||||||||||||||||||||||||||||||||||
Sbjct  486  TAACTAACCAGAAAGGGACGGCTAACTACGTGCCAGCAGCCGCGGTAATACGTAGGTCCC  545

Query  481  GAGCGTTGTCCGGATTTATTGGGCGTAAAGCGAGCGCAGGCGGTTCTTTAAGTCTGAAGT  540
            ||||||||||||||||||||||||||||||||||||||||||||||||||||||||||||
Sbjct  546  GAGCGTTGTCCGGATTTATTGGGCGTAAAGCGAGCGCAGGCGGTTCTTTAAGTCTGAAGT  605

Query  541  TAAAGGCAGTGGCTTAACCATTGTACGCTTTGGAAACTGGAGGACTTGAGTGCAGAAGGG  600
            ||||||||||||||||||||||||||||||||||||||||||||||||||||||||||||
Sbjct  606  TAAAGGCAGTGGCTTAACCATTGTACGCTTTGGAAACTGGAGGACTTGAGTGCAGAAGGG  665

Query  601  GAGAGTGGAATTCCATGTGTAGCGGTGAAATGCGTAGATATATGGAGGAACACCGGTGGC  660
            ||||||||||||||||||||||||||||||||||||||||||||||||||||||||||||
Sbjct  666  GAGAGTGGAATTCCATGTGTAGCGGTGAAATGCGTAGATATATGGAGGAACACCGGTGGC  725

Query  661  GAAAGCGGCTCTCTGGTCTGTAACTGACGCTGAGGCTCGAAAGCGTGGGGAGCAAACAGG  720
            ||||||||||||||||||||||||||||||||||||||||||||||||||||||||||||
Sbjct  726  GAAAGCGGCTCTCTGGTCTGTAACTGACGCTGAGGCTCGAAAGCGTGGGGAGCAAACAGG  785

Query  721  ATTAGATACCCTGGTAGTCCACGCCGTAAACGATGAGTGCTAGGTGTTAGGCCCTTTCCG  780
            ||||||||||||||||||||||||||||||||||||||||||||||||||||||||||||
Sbjct  786  ATTAGATACCCTGGTAGTCCACGCCGTAAACGATGAGTGCTAGGTGTTAGGCCCTTTCCG  845

Query  781  GGGCTTAGTGCCGCAGCTAACGCATTAAGC  810
            ||||||||||||||||||||||||||||||
Sbjct  846  GGGCTTAGTGCCGCAGCTAACGCATTAAGC  875
```

Streptococcus agalactiae strain NYD001 16S ribosomal RNA gene, partial sequence
Sequence ID: **MF113258.1** Length: 1510 Number of Matches: 1
Range 1: 66 to 875

Score	Expect	Identities	Gaps	Strand	Frame
1496 bits(810)	0.0()	810/810(100%)	0/810(0%)	Plus/Plus	

Features:

```
Query  1    TGTTTGGTGTTTACACTAGACTGATGAGTTGCGAACGGGTGAGTAACGCGTAGGTAACCT  60
            ||||||||||||||||||||||||||||||||||||||||||||||||||||||||||||
Sbjct  66   TGTTTGGTGTTTACACTAGACTGATGAGTTGCGAACGGGTGAGTAACGCGTAGGTAACCT  125

Query  61   GCCTCATAGCGGGGGATAACTATTGGAAACGATAGCTAATACCGCATAAGAGTAATTAAC  120
            ||||||||||||||||||||||||||||||||||||||||||||||||||||||||||||
Sbjct  126  GCCTCATAGCGGGGGATAACTATTGGAAACGATAGCTAATACCGCATAAGAGTAATTAAC  185
```

```
Query  121  ACATGTTAGTTATTTAAAAGGAGCAATTGCTTCACTGTGAGATGGACCTGCGTTGTATTA  180
            ||||||||||||||||||||||||||||||||||||||||||||||||||||||||||||
Sbjct  186  ACATGTTAGTTATTTAAAAGGAGCAATTGCTTCACTGTGAGATGGACCTGCGTTGTATTA  245
Query  181  GCTAGTTGGTGAGGTAAAGGCTCACCAAGGCGACGATACATAGCCGACCTGAGAGGGTGA  240
            ||||||||||||||||||||||||||||||||||||||||||||||||||||||||||||
Sbjct  246  GCTAGTTGGTGAGGTAAAGGCTCACCAAGGCGACGATACATAGCCGACCTGAGAGGGTGA  305
Query  241  TCGGCCACACTGGGACTGAGACACGGCCCAGACTCCTACGGGAGGCAGCAGTAGGGAATC  300
            ||||||||||||||||||||||||||||||||||||||||||||||||||||||||||||
Sbjct  306  TCGGCCACACTGGGACTGAGACACGGCCCAGACTCCTACGGGAGGCAGCAGTAGGGAATC  365
Query  301  TTCGGCAATGGACGGAAGTCTGACCGAGCAACGCCGCGTGAGTGAAGAAGGTTTTCGGAT  360
            ||||||||||||||||||||||||||||||||||||||||||||||||||||||||||||
Sbjct  366  TTCGGCAATGGACGGAAGTCTGACCGAGCAACGCCGCGTGAGTGAAGAAGGTTTTCGGAT  425
Query  361  CGTAAAGCTCTGTTGTTAGAGAAGAACGTTGGTAGGAGTGGAAAATCTACCAAGTGACGG  420
            ||||||||||||||||||||||||||||||||||||||||||||||||||||||||||||
Sbjct  426  CGTAAAGCTCTGTTGTTAGAGAAGAACGTTGGTAGGAGTGGAAAATCTACCAAGTGACGG  485
Query  421  TAACTAACCAGAAAGGGACGGCTAACTACGTGCCAGCAGCCGCGGTAATACGTAGGTCCC  480
            ||||||||||||||||||||||||||||||||||||||||||||||||||||||||||||
Sbjct  486  TAACTAACCAGAAAGGGACGGCTAACTACGTGCCAGCAGCCGCGGTAATACGTAGGTCCC  545
Query  481  GAGCGTTGTCCGGATTTATTGGGCGTAAAGCGAGCGCAGGCGGTTCTTTAAGTCTGAAGT  540
            ||||||||||||||||||||||||||||||||||||||||||||||||||||||||||||
Sbjct  546  GAGCGTTGTCCGGATTTATTGGGCGTAAAGCGAGCGCAGGCGGTTCTTTAAGTCTGAAGT  605
Query  541  TAAAGGCAGTGGCTTAACCATTGTACGCTTTGGAAACTGGAGGACTTGAGTGCAGAAGGG  600
            ||||||||||||||||||||||||||||||||||||||||||||||||||||||||||||
Sbjct  606  TAAAGGCAGTGGCTTAACCATTGTACGCTTTGGAAACTGGAGGACTTGAGTGCAGAAGGG  665
Query  601  GAGAGTGGAATTCCATGTGTAGCGGTGAAATGCGTAGATATATGGAGGAACACCGGTGGC  660
            ||||||||||||||||||||||||||||||||||||||||||||||||||||||||||||
Sbjct  666  GAGAGTGGAATTCCATGTGTAGCGGTGAAATGCGTAGATATATGGAGGAACACCGGTGGC  725
Query  661  GAAAGCGGCTCTCTGGTCTGTAACTGACGCTGAGGCTCGAAAGCGTGGGGAGCAAACAGG  720
            ||||||||||||||||||||||||||||||||||||||||||||||||||||||||||||
Sbjct  726  GAAAGCGGCTCTCTGGTCTGTAACTGACGCTGAGGCTCGAAAGCGTGGGGAGCAAACAGG  785
Query  721  ATTAGATACCCTGGTAGTCCACGCCGTAAACGATGAGTGCTAGGTGTTAGGCCCTTTCCG  780
            ||||||||||||||||||||||||||||||||||||||||||||||||||||||||||||
Sbjct  786  ATTAGATACCCTGGTAGTCCACGCCGTAAACGATGAGTGCTAGGTGTTAGGCCCTTTCCG  845
Query  781  GGGCTTAGTGCCGCAGCTAACGCATTAAGC  810
            ||||||||||||||||||||||||||||||
Sbjct  846  GGGCTTAGTGCCGCAGCTAACGCATTAAGC  875
```

Streptococcus agalactiae strain BJ1411 16S ribosomal RNA gene, partial sequence
Sequence ID: **MF113257.1** Length: 1510 Number of Matches: 1
Range 1: 66 to 875

Score	Expect	Identities	Gaps	Strand	Frame
1496 bits(810)	0.0()	810/810(100%)	0/810(0%)	Plus/Plus	

Features:

```
Query  1    TGTTTGGTGTTTACACTAGACTGATGAGTTGCGAACGGGTGAGTAACGCGTAGGTAACCT  60
            ||||||||||||||||||||||||||||||||||||||||||||||||||||||||||||
Sbjct  66   TGTTTGGTGTTTACACTAGACTGATGAGTTGCGAACGGGTGAGTAACGCGTAGGTAACCT  125
Query  61   GCCTCATAGCGGGGGATAACTATTGGAAACGATAGCTAATACCGCATAAGAGTAATTAAC  120
            ||||||||||||||||||||||||||||||||||||||||||||||||||||||||||||
Sbjct  126  GCCTCATAGCGGGGGATAACTATTGGAAACGATAGCTAATACCGCATAAGAGTAATTAAC  185
Query  121  ACATGTTAGTTATTTAAAAGGAGCAATTGCTTCACTGTGAGATGGACCTGCGTTGTATTA  180
            ||||||||||||||||||||||||||||||||||||||||||||||||||||||||||||
Sbjct  186  ACATGTTAGTTATTTAAAAGGAGCAATTGCTTCACTGTGAGATGGACCTGCGTTGTATTA  245
Query  181  GCTAGTTGGTGAGGTAAAGGCTCACCAAGGCGACGATACATAGCCGACCTGAGAGGGTGA  240
            ||||||||||||||||||||||||||||||||||||||||||||||||||||||||||||
Sbjct  246  GCTAGTTGGTGAGGTAAAGGCTCACCAAGGCGACGATACATAGCCGACCTGAGAGGGTGA  305
Query  241  TCGGCCACACTGGGACTGAGACACGGCCCAGACTCCTACGGGAGGCAGCAGTAGGGAATC  300
            ||||||||||||||||||||||||||||||||||||||||||||||||||||||||||||
Sbjct  306  TCGGCCACACTGGGACTGAGACACGGCCCAGACTCCTACGGGAGGCAGCAGTAGGGAATC  365
Query  301  TTCGGCAATGGACGGAAGTCTGACCGAGCAACGCCGCGTGAGTGAAGAAGGTTTTCGGAT  360
            ||||||||||||||||||||||||||||||||||||||||||||||||||||||||||||
Sbjct  366  TTCGGCAATGGACGGAAGTCTGACCGAGCAACGCCGCGTGAGTGAAGAAGGTTTTCGGAT  425
Query  361  CGTAAAGCTCTGTTGTTAGAGAAGAACGTTGGTAGGAGTGGAAAATCTACCAAGTGACGG  420
            ||||||||||||||||||||||||||||||||||||||||||||||||||||||||||||
Sbjct  426  CGTAAAGCTCTGTTGTTAGAGAAGAACGTTGGTAGGAGTGGAAAATCTACCAAGTGACGG  485
Query  421  TAACTAACCAGAAAGGGACGGCTAACTACGTGCCAGCAGCCGCGGTAATACGTAGGTCCC  480
            ||||||||||||||||||||||||||||||||||||||||||||||||||||||||||||
Sbjct  486  TAACTAACCAGAAAGGGACGGCTAACTACGTGCCAGCAGCCGCGGTAATACGTAGGTCCC  545
Query  481  GAGCGTTGTCCGGATTTATTGGGCGTAAAGCGAGCGCAGGCGGTTCTTTAAGTCTGAAGT  540
            ||||||||||||||||||||||||||||||||||||||||||||||||||||||||||||
Sbjct  546  GAGCGTTGTCCGGATTTATTGGGCGTAAAGCGAGCGCAGGCGGTTCTTTAAGTCTGAAGT  605
Query  541  TAAAGGCAGTGGCTTAACCATTGTACGCTTTGGAAACTGGAGGACTTGAGTGCAGAAGGG  600
            ||||||||||||||||||||||||||||||||||||||||||||||||||||||||||||
Sbjct  606  TAAAGGCAGTGGCTTAACCATTGTACGCTTTGGAAACTGGAGGACTTGAGTGCAGAAGGG  665
Query  601  GAGAGTGGAATTCCATGTGTAGCGGTGAAATGCGTAGATATATGGAGGAACACCGGTGGC  660
            ||||||||||||||||||||||||||||||||||||||||||||||||||||||||||||
Sbjct  666  GAGAGTGGAATTCCATGTGTAGCGGTGAAATGCGTAGATATATGGAGGAACACCGGTGGC  725
Query  661  GAAAGCGGCTCTCTGGTCTGTAACTGACGCTGAGGCTCGAAAGCGTGGGGAGCAAACAGG  720
            ||||||||||||||||||||||||||||||||||||||||||||||||||||||||||||
Sbjct  726  GAAAGCGGCTCTCTGGTCTGTAACTGACGCTGAGGCTCGAAAGCGTGGGGAGCAAACAGG  785
Query  721  ATTAGATACCCTGGTAGTCCACGCCGTAAACGATGAGTGCTAGGTGTTAGGCCCTTTCCG  780
            ||||||||||||||||||||||||||||||||||||||||||||||||||||||||||||
Sbjct  786  ATTAGATACCCTGGTAGTCCACGCCGTAAACGATGAGTGCTAGGTGTTAGGCCCTTTCCG  845
Query  781  GGGCTTAGTGCCGCAGCTAACGCATTAAGC  810
            ||||||||||||||||||||||||||||||
Sbjct  846  GGGCTTAGTGCCGCAGCTAACGCATTAAGC  875
```

Streptococcus agalactiae strain QX1501 16S ribosomal RNA gene, partial sequence
Sequence ID: **MF037804.1** Length: 1510 Number of Matches: 1
Range 1: 66 to 875

Score	Expect	Identities	Gaps	Strand	Frame
1496 bits(810)	0.0()	810/810(100%)	0/810(0%)	Plus/Plus	

Features:

```
Query  1    TGTTTGGTGTTTACACTAGACTGATGAGTTGCGAACGGGTGAGTAACGCGTAGGTAACCT  60
            ||||||||||||||||||||||||||||||||||||||||||||||||||||||||||||
Sbjct  66   TGTTTGGTGTTTACACTAGACTGATGAGTTGCGAACGGGTGAGTAACGCGTAGGTAACCT  125
Query  61   GCCTCATAGCGGGGGATAACTATTGGAAACGATAGCTAATACCGCATAAGAGTAATTAAC  120
            ||||||||||||||||||||||||||||||||||||||||||||||||||||||||||||
Sbjct  126  GCCTCATAGCGGGGGATAACTATTGGAAACGATAGCTAATACCGCATAAGAGTAATTAAC  185
Query  121  ACATGTTAGTTATTTAAAAGGAGCAATTGCTTCACTGTGAGATGGACCTGCGTTGTATTA  180
            ||||||||||||||||||||||||||||||||||||||||||||||||||||||||||||
Sbjct  186  ACATGTTAGTTATTTAAAAGGAGCAATTGCTTCACTGTGAGATGGACCTGCGTTGTATTA  245
Query  181  GCTAGTTGGTGAGGTAAAGGCTCACCAAGGCGACGATACATAGCCGACCTGAGAGGGTGA  240
            ||||||||||||||||||||||||||||||||||||||||||||||||||||||||||||
Sbjct  246  GCTAGTTGGTGAGGTAAAGGCTCACCAAGGCGACGATACATAGCCGACCTGAGAGGGTGA  305
Query  241  TCGGCCACACTGGGACTGAGACACGGCCCAGACTCCTACGGGAGGCAGCAGTAGGGAATC  300
            ||||||||||||||||||||||||||||||||||||||||||||||||||||||||||||
Sbjct  306  TCGGCCACACTGGGACTGAGACACGGCCCAGACTCCTACGGGAGGCAGCAGTAGGGAATC  365
Query  301  TTCGGCAATGGACGGAAGTCTGACCGAGCAACGCCGCGTGAGTGAAGAAGGTTTTCGGAT  360
            ||||||||||||||||||||||||||||||||||||||||||||||||||||||||||||
```

```
Sbjct  366   TTCGGCAATGGACGGAAGTCTGACCGAGCAACGCCGCGTGAGTGAAGAAGGTTTTCGGAT  425
Query  361   CGTAAAGCTCTGTTGTTAGAGAAGAACGTTGGTAGGAGTGGAAAATCTACCAAGTGACGG  420
             ||||||||||||||||||||||||||||||||||||||||||||||||||||||||||||
Sbjct  426   CGTAAAGCTCTGTTGTTAGAGAAGAACGTTGGTAGGAGTGGAAAATCTACCAAGTGACGG  485
Query  421   TAACTAACCAGAAAGGGACGGCTAACTACGTGCCAGCAGCCGCGGTAATACGTAGGTCCC  480
             ||||||||||||||||||||||||||||||||||||||||||||||||||||||||||||
Sbjct  486   TAACTAACCAGAAAGGGACGGCTAACTACGTGCCAGCAGCCGCGGTAATACGTAGGTCCC  545
Query  481   GAGCGTTGTCCGGATTTATTGGGCGTAAAGCGAGCGCAGGCGGTTCTTTAAGTCTGAAGT  540
             ||||||||||||||||||||||||||||||||||||||||||||||||||||||||||||
Sbjct  546   GAGCGTTGTCCGGATTTATTGGGCGTAAAGCGAGCGCAGGCGGTTCTTTAAGTCTGAAGT  605
Query  541   TAAAGGCAGTGGCTTAACCATTGTACGCTTTGGAAACTGGAGGACTTGAGTGCAGAAGGG  600
             ||||||||||||||||||||||||||||||||||||||||||||||||||||||||||||
Sbjct  606   TAAAGGCAGTGGCTTAACCATTGTACGCTTTGGAAACTGGAGGACTTGAGTGCAGAAGGG  665
Query  601   GAGAGTGGAATTCCATGTGTAGCGGTGAAATGCGTAGATATATGGAGGAACACCGGTGGC  660
             ||||||||||||||||||||||||||||||||||||||||||||||||||||||||||||
Sbjct  666   GAGAGTGGAATTCCATGTGTAGCGGTGAAATGCGTAGATATATGGAGGAACACCGGTGGC  725
Query  661   GAAAGCGGCTCTCTGGTCTGTAACTGACGCTGAGGCTCGAAAGCGTGGGGAGCAAACAGG  720
             ||||||||||||||||||||||||||||||||||||||||||||||||||||||||||||
Sbjct  726   GAAAGCGGCTCTCTGGTCTGTAACTGACGCTGAGGCTCGAAAGCGTGGGGAGCAAACAGG  785
Query  721   ATTAGATACCCTGGTAGTCCACGCCGTAAACGATGAGTGCTAGGTGTTAGGCCCTTTCCG  780
             ||||||||||||||||||||||||||||||||||||||||||||||||||||||||||||
Sbjct  786   ATTAGATACCCTGGTAGTCCACGCCGTAAACGATGAGTGCTAGGTGTTAGGCCCTTTCCG  845
Query  781   GGGCTTAGTGCCGCAGCTAACGCATTAAGC  810
             ||||||||||||||||||||||||||||||
Sbjct  846   GGGCTTAGTGCCGCAGCTAACGCATTAAGC  875
```

Streptococcus agalactiae strain PK1601 16S ribosomal RNA gene, partial sequence
Sequence ID: **MF037803.1** Length: 1510 Number of Matches: 1
Range 1: 66 to 875

Score	Expect	Identities	Gaps	Strand	Frame
1496 bits(810)	0.0()	810/810(100%)	0/810(0%)	Plus/Plus	

Features:

```
Query  1     TGTTTGGTGTTTACACTAGACTGATGAGTTGCGAACGGGTGAGTAACGCGTAGGTAACCT  60
             ||||||||||||||||||||||||||||||||||||||||||||||||||||||||||||
Sbjct  66    TGTTTGGTGTTTACACTAGACTGATGAGTTGCGAACGGGTGAGTAACGCGTAGGTAACCT  125
Query  61    GCCTCATAGCGGGGGATAACTATTGGAAACGATAGCTAATACCGCATAAGAGTAATTAAC  120
             ||||||||||||||||||||||||||||||||||||||||||||||||||||||||||||
Sbjct  126   GCCTCATAGCGGGGGATAACTATTGGAAACGATAGCTAATACCGCATAAGAGTAATTAAC  185
Query  121   ACATGTTAGTTATTTAAAAGGAGCAATTGCTTCACTGTGAGATGGACCTGCGTTGTATTA  180
             ||||||||||||||||||||||||||||||||||||||||||||||||||||||||||||
Sbjct  186   ACATGTTAGTTATTTAAAAGGAGCAATTGCTTCACTGTGAGATGGACCTGCGTTGTATTA  245
Query  181   GCTAGTTGGTGAGGTAAAGGCTCACCAAGGCGACGATACATAGCCGACCTGAGAGGGTGA  240
             ||||||||||||||||||||||||||||||||||||||||||||||||||||||||||||
Sbjct  246   GCTAGTTGGTGAGGTAAAGGCTCACCAAGGCGACGATACATAGCCGACCTGAGAGGGTGA  305
Query  241   TCGGCCACACTGGGACTGAGACACGGCCCAGACTCCTACGGGAGGCAGCAGTAGGGAATC  300
             ||||||||||||||||||||||||||||||||||||||||||||||||||||||||||||
Sbjct  306   TCGGCCACACTGGGACTGAGACACGGCCCAGACTCCTACGGGAGGCAGCAGTAGGGAATC  365
Query  301   TTCGGCAATGGACGGAAGTCTGACCGAGCAACGCCGCGTGAGTGAAGAAGGTTTTCGGAT  360
             ||||||||||||||||||||||||||||||||||||||||||||||||||||||||||||
Sbjct  366   TTCGGCAATGGACGGAAGTCTGACCGAGCAACGCCGCGTGAGTGAAGAAGGTTTTCGGAT  425
Query  361   CGTAAAGCTCTGTTGTTAGAGAAGAACGTTGGTAGGAGTGGAAAATCTACCAAGTGACGG  420
             ||||||||||||||||||||||||||||||||||||||||||||||||||||||||||||
Sbjct  426   CGTAAAGCTCTGTTGTTAGAGAAGAACGTTGGTAGGAGTGGAAAATCTACCAAGTGACGG  485
Query  421   TAACTAACCAGAAAGGGACGGCTAACTACGTGCCAGCAGCCGCGGTAATACGTAGGTCCC  480
             ||||||||||||||||||||||||||||||||||||||||||||||||||||||||||||
Sbjct  486   TAACTAACCAGAAAGGGACGGCTAACTACGTGCCAGCAGCCGCGGTAATACGTAGGTCCC  545
Query  481   GAGCGTTGTCCGGATTTATTGGGCGTAAAGCGAGCGCAGGCGGTTCTTTAAGTCTGAAGT  540
             ||||||||||||||||||||||||||||||||||||||||||||||||||||||||||||
Sbjct  546   GAGCGTTGTCCGGATTTATTGGGCGTAAAGCGAGCGCAGGCGGTTCTTTAAGTCTGAAGT  605
Query  541   TAAAGGCAGTGGCTTAACCATTGTACGCTTTGGAAACTGGAGGACTTGAGTGCAGAAGGG  600
             ||||||||||||||||||||||||||||||||||||||||||||||||||||||||||||
Sbjct  606   TAAAGGCAGTGGCTTAACCATTGTACGCTTTGGAAACTGGAGGACTTGAGTGCAGAAGGG  665
Query  601   GAGAGTGGAATTCCATGTGTAGCGGTGAAATGCGTAGATATATGGAGGAACACCGGTGGC  660
             ||||||||||||||||||||||||||||||||||||||||||||||||||||||||||||
Sbjct  666   GAGAGTGGAATTCCATGTGTAGCGGTGAAATGCGTAGATATATGGAGGAACACCGGTGGC  725
Query  661   GAAAGCGGCTCTCTGGTCTGTAACTGACGCTGAGGCTCGAAAGCGTGGGGAGCAAACAGG  720
             ||||||||||||||||||||||||||||||||||||||||||||||||||||||||||||
Sbjct  726   GAAAGCGGCTCTCTGGTCTGTAACTGACGCTGAGGCTCGAAAGCGTGGGGAGCAAACAGG  785
Query  721   ATTAGATACCCTGGTAGTCCACGCCGTAAACGATGAGTGCTAGGTGTTAGGCCCTTTCCG  780
             ||||||||||||||||||||||||||||||||||||||||||||||||||||||||||||
Sbjct  786   ATTAGATACCCTGGTAGTCCACGCCGTAAACGATGAGTGCTAGGTGTTAGGCCCTTTCCG  845
Query  781   GGGCTTAGTGCCGCAGCTAACGCATTAAGC  810
             ||||||||||||||||||||||||||||||
Sbjct  846   GGGCTTAGTGCCGCAGCTAACGCATTAAGC  875
```

BLAST is a registered trademark of the National Library of Medicine

Support center Mailing list YouTube

- National Library Of Medicine
- National Institutes Of Health
- U.S. Department of Health & Human Services
- USA.gov

NCBI
National Center for Biotechnology Information, U.S. National Library of Medicine 8600 Rockville Pike, Bethesda MD, 20894 USA
Policies and Guidelines | Contact
