# Supplementary material for: Isolation of Streptococcus agalactiae in a female llama (Lama glama) in South Tyrol (Italy)
Source: BMC Vet Res. 2018 Nov 13;14:343. doi: 10.1186/s12917-018-1676-9 (PMC6234556; doi:10.1186/s12917-018-1676-9)
Supplement: Supplementary file 3 — Clustal Omega allignment. (PDF 62 kb) [file 12917_2018_1676_MOESM3_ESM.pdf]

Clustal Omega

Results for job clustalo-I20180817-120426-0368-49877271-p2m

```
CLUSTAL O(1.2.4) multiple sequence alignment


18RS-1924-6      ------TGTTTGGTGTTTACACTAGACTGATGAGTTGCGAACGGGTGAGTAACGCGTAGG   54
18RS-1924-7      CGCTGATGTTTGGTGTTTACACTAGACTGATGAGTTGCGAACGGGTGAGTAACGCGTAGG   60
                       ******************************************************

18RS-1924-6      TAACCTGCCTCATAGCGGGGGATAACTATTGGAAACGATAGCTAATACCGCATAAGAGTA   114
18RS-1924-7      TAACCTGCCTCATAGCGGGGGATAACTATTGGAAACGATAGCTAATACCGCATAAGAGTA   120
                 ************************************************************

18RS-1924-6      ATTAACACATGTTAGTTATTTAAAAGGAGCAATTGCTTCACTGTGAGATGGACCTGCGTT   174
18RS-1924-7      ATTAACACATGTTAGTTATTTAAAAGGAGCAATTGCTTCACTGTGAGATGGACCTGCGTT   180
                 ************************************************************

18RS-1924-6      GTATTAGCTAGTTGGTGAGGTAAAGGCTCACCAAGGCGACGATACATAGCCGACCTGAGA   234
18RS-1924-7      GTATTAGCTAGTTGGTGAGGTAAAGGCTCACCAAGGCGACGATACATAGCCGACCTGAGA   240
                 ************************************************************

18RS-1924-6      GGGTGATCGGCCACACTGGGACTGAGACACGGCCCAGACTCCTACGGGAGGCAGCAGTAG   294
18RS-1924-7      GGGTGATCGGCCACACTGGGACTGAGACACGGCCCAGACTCCTACGGGAGGCAGCAGTAG   300
                 ************************************************************

18RS-1924-6      GGAATCTTCGGCAATGGACGGAAGTCTGACCGAGCAACGCCGCGTGAGTGAAGAAGGTTT   354
18RS-1924-7      GGAATCTTCGGCAATGGACGGAAGTCTGACCGAGCAACGCCGCGTGAGTGAAGAAGGTTT   360
                 ************************************************************

18RS-1924-6      TCGGATCGTAAAGCTCTGTTGTTAGAGAAGAACGTTGGTAGGAGTGGAAAATCTACCAAG   414
18RS-1924-7      TCGGATCGTAAAGCTCTGTTGTTAGAGAAGAACGTTGGTAGGAGTGGAAAATCTACCAAG   420
                 ************************************************************

18RS-1924-6      TGACGGTAACTAACCAGAAAGGGACGGCTAACTACGTGCCAGCAGCCGCGGTAATACGTA   474
18RS-1924-7      TGACGGTAACTAACCAGAAAGGGACGGCTAACTACGTGCCAGCAGCCGCGGTAATACGTA   480
                 ************************************************************

18RS-1924-6      GGTCCCGAGCGTTGTCCGGATTTATTGGGCGTAAAGCGAGCGCAGGCGGTTCTTTAAGTC   534
18RS-1924-7      GGTCCCGAGCGTTGTCCGGATTTATTGGGCGTAAAGCGAGCGCAGGCGGTTCTTTAAGTC   540
                 ************************************************************

18RS-1924-6      TGAAGTTAAAGGCAGTGGCTTAACCATTGTACGCTTTGGAAACTGGAGGACTTGAGTGCA   594
18RS-1924-7      TGAAGTTAAAGGCAGTGGCTTAACCATTGTACGCTTTGGAAACTGGAGGACTTGAGTGCA   600
                 ************************************************************

18RS-1924-6      GAAGGGGAGAGTGGAATTCCATGTGTAGCGGTGAAATGCGTAGATATATGGAGGAACACC   654
18RS-1924-7      GAAGGGGAGAGTGGAATTCCATGTGTAGCGGTGAAATGCGTAGATATATGGAGGAACACC   660
                 ************************************************************

18RS-1924-6      GGTGGCGAAAGCGGCTCTCTGGTCTGTAACTGACGCTGAGGCTCGAAAGCGTGGGGAGCA   714
18RS-1924-7      GGTGGCGAAAGCGGCTCTCTGGTCTGTAACTGACGCTGAGGCTCGAAAGCGTGGGGAGCA   720
                 ************************************************************

18RS-1924-6      AACAGGATTAGATACCCTGGTAGTCCACGCCGTAAACGATGAGTGCTAGGTGTTAGGCCC   774
18RS-1924-7      AACAGGATTAGATACCCTGGTAGTCCACGCCGTAAACGATGAGTGCTAGGTGTTAGGCCC   780
                 ************************************************************

18RS-1924-6      TTTCCGGGGCTTAGTGCCGCAGCTAACGCATTAAGC----       810
18RS-1924-7      TTTCCGGGGCTTAGTGCCGCAGCTAACGCATTAAGCACTC       820
                 ************************************
```

This website requires cookies, and the limited processing of your personal data in order to function. By using the site you are agreeing to this as outlined in our Privacy Notice (https://www.ebi.ac.uk/data-protection/privacy-notice/embl-ebi-public-website) and Terms of Use (https://www.ebi.ac.uk/about/terms-of-use).

I agree, dismiss this banner
